# Meta-analysis of plant growth-promoting rhizobacteria interaction with host plants: implications for drought stress response gene expression

**DOI:** 10.3389/fpls.2023.1282553

**Published:** 2024-01-15

**Authors:** Roberta Ferrante, Chiara Campagni, Cristina Vettori, Alice Checcucci, Cesare Garosi, Donatella Paffetti

**Affiliations:** ^1^ National Biodiversity Future Center (NBFC), Palermo, Italy; ^2^ Dipartimento di Scienze e Tecnologie Agrarie, Alimentari, Ambientali e Forestali (DAGRI), Università degli Studi di Firenze, Florence, Italy; ^3^ Istituto di Bioscienze e Biorisorse (IBBR), Consiglio Nazionale delle Ricerche (CNR), Sesto Fiorentino, Italy

**Keywords:** PGPR, plant gene regulation, transcriptional factors regulation, meta-analysis, abiotic stress, crops, drought stress

## Abstract

**Introduction:**

The molecular and physiological mechanisms activated in plants during drought stress tolerance are regulated by several key genes with both metabolic and regulatory roles. Studies focusing on crop gene expression following plant growth-promoting rhizobacteria (PGPR) inoculation may help understand which bioinoculant is closely related to the induction of abiotic stress responses.

**Methods:**

Here, we performed a meta-analysis following Preferred Reporting Items for Systematic Reviews and Meta-Analyses (PRISMA) guidelines to summarise information regarding plant-PGPR interactions, focusing on the regulation of nine genes involved in plant drought stress response. The literature research yielded 3,338 reports, of which only 41 were included in the meta-analysis based on the chosen inclusion criteria. The meta-analysis was performed on four genes (ACO, APX, ACS and *DREB2*); the other five genes (ERD15, MYB, MYC, acdS, WRKY) had an insufficient number of eligible articles.

**Results:**

Forest plots obtained through each meta-analysis showed that the overexpression of ACO, APX, ACS and *DREB2* genes was not statistically significant. Unlike the other genes, *DREB2* showed statistically significant results in both the presence and absence of PGPR. Considering I2>75 %, the results showed a high heterogeneity among the studies included, and the cause for this was examined using subgroup analysis. Moreover, the funnel plot and Egger’s test showed that the analyses were affected by strong publication bias.

**Discussion:**

This study argues that the presence of PGPR may not significantly influence the expression of drought stress response-related crop genes. This finding may be due to high heterogeneity, lack of data on the genes examined, and significant publication bias.

## 1 Introduction

Current agricultural practices aiming at maximizing targeting maximum yield has serious impacts on natural ecosystems, including the depletion of natural resources, a decline in ecosystem services, increased soil erosion, and a significant losses in biodiversity and soil organic matter (Struik et al., 2014; Watson et al., 2021). Therefore, scientists are focusing on maintaining crop productivity and sustainable agriculture. Accounting for the increasing demand for food and environmentally friendly and economically beneficial practices is necessary to preserve our planet. Climate change has impacted agricultural productivity alongside intensification. In fact, plant health is also affected by abiotic stresses, which limit the overall crop yield by more than 50% every year (Ojuederie et al., 2019). Drought is a significant abiotic stressor strongly impacting crop productivity (Golldack et al., 2014; Anjum et al., 2017; Hussain et al., 2018) and decreases the amount of arable land. The “agroecological practices” developed in the last century, which include livestock manure utilisation, cover crops and intercropping, agroforestry, biological control, and biodiversity conservation, remain the most used practices to realise a more “green” agriculture (Wezel et al., 2014). However, recognizing beneficial soil and plant microorganisms as crucial for enhancing plant nutrition and protection is not a recent development. Indeed, for several decades, the scientific community has deepened the study of complex networks established in the biosphere between plants and their associated microbiota, focusing on the role of microorganisms in plant physiology (Singh et al., 2016). Plants cannot be considered singular entities but are the result of a close relationship established with their microbiota, which can be beneficial, neutral, or pathogenic and is usually controlled by the plant’s genotype and environment (Meier et al., 2022; Wagner, 2022). Most bacteria that initially colonise the rhizosphere or plant surfaces as epiphytes can enter plants and proliferate as endophytes, thereby establishing a mutualistic association. Mutualists are often crucial for plant health and resilience (Vandenkoornhuyse et al., 2015), realising numerous mechanisms that enhance plant growth, such as plant growth-promoting (PGP) microorganisms. The main direct mechanisms involved are *i*) endogenous phytohormone modulation (auxins, gibberellins, cytokinins, and ethylene), *ii*) nutrient solubilisation, mainly phosphorus (P) and potassium (K), and *iii*) nutrient bioavailability (Çakmakçı, 2016) such as biological fixation of atmospheric nitrogen (N_2_). Among the most well-known indirect mechanisms are the regulation of plant-induced (ISR) and plant-acquired (ASR) systemic resistance (Alori et al., 2017; Enebe and Babalola, 2018), the production of secondary metabolites such as auxins and 1-aminocyclopropane-1-carboxylate (ACC) deaminase, and the general control of pathogenic diseases (Kamle et al., 2020) through the production of antibiotics, lytic enzymes, and siderophores. It is known that PGP microorganisms from the rhizosphere, named PGP rhizobacteria (PGPR), and exploiting their abilities to interact with the host could help plants in harsh environmental conditions. They can be used as bioinoculants in agricultural practices (Singh et al., 2011) to reduce the use of chemical fertilisers. For instance, *Andrographis paniculate* inoculated with *Pseudomonas* sp. showed numerous benefits, including enhanced growth, early flowering, and increased production of photosynthetic pigments (Thakur et al., 2023). Moreover, Thakur and Yadav (2023) demonstrated that AB-11, a strain of *Streptomyces* sp., hold promise as an agent for plant growth promotion, providing an environmentally friendly alternative to chemical fertilizers. The success of their utilisation depends on several factors, such as survival in the soil, ability to interact with the existing microbiota, genetic compatibility with the crop on which they are applied (Cangioli et al., 2021), and environmental factors. Some PGPR are characterised by specific PGP traits, such as heavy metal detoxifying activities, biological control, and abiotic stress tolerance (Egamberdieva and Lugtenberg, 2014), which allow them to be considered a resource for preserving agricultural productivity during adverse conditions (Chandra et al., 2021). It is essential to consider that the plant response to abiotic stresses is a complex phenomenon affecting both plant development and the metabolism and physiology of its microbiota (Omae and Tsuda, 2022). The regulatory mechanisms of plant stress responses include gene expression adjustments aimed at physiological and morphological adaptations (Kooyers, 2015). Several plant genes are involved in abiotic stress responses and drought tolerance (Gong et al., 2020), and many are secondary messengers and transcription factors (TFs) that participate in signalling pathways. A deeper understanding of the extreme complexity of such pathways should be the way to devise new and applicable strategies to improve plant tolerance to drought (Joshi et al., 2016), especially in light of some studies that report that plants inoculated with microbes show different patterns of gene expression compared to non-inoculated plants (Creus et al., 2005; Molina-Favero et al., 2008; Dimkpa C.O.et al., 2009; Lim and Kim, 2013; Sarma and Saikia, 2014; Vurukonda et al., 2016). Thus, studies focusing on plant gene expression in the presence of PGPR can play a significant role in understanding whether PGPR can be used as a resource for drought stress resistance. In this study, we provide a meta-analysis of peer-reviewed studies to verify whether the interaction established between PGPR and host plants may beneficially affect plant resilience to drought stress in a significant and detectable way.

## 2 Materials and methods

### 2.1 Protocol used to carry out systematic review and meta-analysis

**Figure 1:**
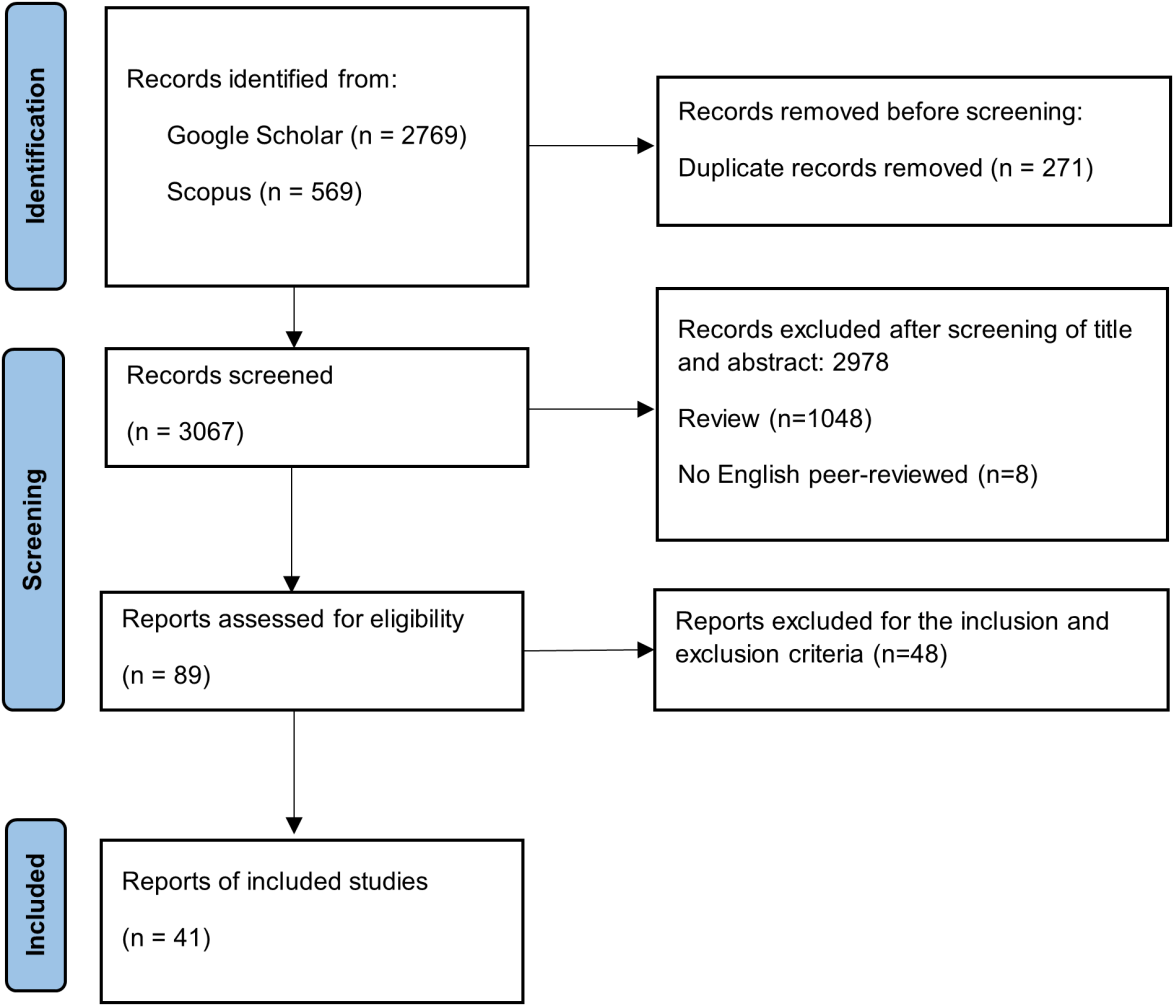
PRISMA workflow diagram to perform the systematic review and the meta-analysis (adapted from flow diagram proposed by Page et al., 2021). PRISMA, Preferred Reporting Items for Systematic Reviews and Meta-Analyses.

We performed a systematic review and meta-analysis of the influence of PGPR on plant genes that respond to drought stress following the guidelines of the Preferred Reporting Items for Systematic Reviews and Meta-Analyses (PRISMA) statement (Moher et al., 2009). The PRISMA protocol includes four steps for conducting a meta-analysis: *i*) identification of studies *via* bibliographic research, *ii*) screening of obtained studies *via* title–abstract analysis, *iii*) full-text screening to identify studies that meet the inclusion and exclusion criteria, and *iv*) detection of eligible studies that can be included in the meta-analysis (**Figure 1**) (Moher et al., 2009; Nakagawa et al., 2017).

### 2.2 Bibliographic research and search strategy

This study aimed to describe information from different studies on gene expression during water stress with/without the presence of PGPR. Therefore, we mainly focused on the following bacterial genes: *1-aminocyclopropane-1-carboxylate deaminase* (*acdS*) and on plant genes *aminocyclopropane-1-carboxylate synthetase* (*ACS*), *1-aminocyclopropane-1-carboxylate* (*ACO*), *ascorbate peroxidase* (*APX*), *early responsive to dehydration 15* (*ERD15*), *WRKY*, *myelocytomatosis* (*MYC*), *myeloblastosis* (*MYB*), and *dehydration-responsive element-binding protein 2* (*DREB2*). Firstly, a bibliographic search was conducted to collect studies regarding gene expression in crops during drought and in the presence of PGPR. Secondly, bibliographic research was performed to gather data on gene expression in crops during drought in the absence of PGPR. Google Scholar and Scopus were used as search engines. The keywords used for the first bibliographic research on Google Scholar were “gene AND expression AND drought AND stress AND plants AND PGPR”. The word selection used for all the genes in Scopus was ALL (“gene ID” AND “drought stress” AND “PGPR” OR “PGPB”). Only for *DREB2* was the bibliographic research regarding *DREB2* expression in plants subjected to drought stress and without PGPR yielded positive results. The search terms used were DREB2 AND expression AND drought AND stress AND plant”. All identified studies are listed in the **Supplementary Bibliography** (**Supplementary File 1**).

### 2.3 Study selection

After obtaining articles from Google Scholar and Scopus, a database was created for each analysed gene in order to simplify the preliminary screening of the studies, in particular, the presence of PGPR, *ACS* (**Table S1**), *APX* (**Table S2**), *ERD15* (**Table S3**), *MYB* (**Table S4**), *MYC* (**Table S5**), *acdS* (**Table S6**), *ACO* (**Table S7**), *WRKY* (**Table S8**), *DREB2* (**Table S9**), and *DREB2* without PGPR (**Table S10**). The tables report the keywords used to search the studies, the title of the study, the author’s first name, the abstract, the decision of rejection/acceptance, and the reasons for exclusion. Screening was performed by assessing titles and abstracts. After this step, a final screening of the studies based on the inclusion criteria was performed. The inclusion criteria were *i*) the focus on herbaceous/crop plants, *ii*) the number of individuals, *iii*) the presence of data regarding selected gene expression (i.e., log_2_ fold change (FC)) assessed by quantitative PCR, *iv*) the presence of dispersion indices, such as *standard error* (*SE*) or *standard deviation* (*SD*), and *v*) the presence of controls to compare gene expression between control and treated plants. Unfortunately, some studies did not report *SD* or *SE*, which are crucial for calculating the size effect. Therefore, a prognostic method was adopted to allocate an assigned *SD* (Ma et al., 2008; Weir et al., 2018). Two reviewers identified and examined eligible studies.

### 2.4 Data extraction

After eligible studies that met the inclusion criteria were identified, data extraction, such as *FC*, number of individuals/number of replicates, *SE*, and *SD*, was performed. These data were indispensable for calculating the effect size. In some studies, *FC* and *SD* values were obtained using Web Plot Digitiser (version 4.6), a web tool that allows data acquisition from plots and images (“WebPlotDigitizer - Extract data from plots, images, and maps”, n.d.) (Rohatgi, 2022). As mentioned previously, a prognostic method was adopted to determine the assigned *SD*. In particular, the SE values were obtained from another distribution consisting of data extracted from Borges et al. (2012); Feng et al. (2019); Le et al. (2012); Nabi et al. (2021), Neves-Borges et al. (2012); Song et al. (2016); Thirumalaikumar et al. (2018), and Wu et al. (2014). Thus, it was possible to predict the missing *SE* and the *SD* of our sampling distribution.

### 2.5 Effect size calculation

It is essential to select an appropriate effect size for a meta-analysis. In this case, continuous outputs were treated; therefore, Cohen’s *d* was used as the effect size. Cohen’s *d* estimates the standard mean difference of an effect (Lakens, 2013) between two groups, such as the control and treatment (Lakens, 2013; Nakagawa et al., 2017), and was calculated using Equation 1:


(1) $$d=\frac{\bar{FC_{1}}-\bar{FC_{2}}}{\sqrt{\frac{(n_{1}-1)SD_{1}^{2}+\left(n_{1}-1\right)\;SD_{2}^{2}}{n_{1}+n_{2}}}}$$

where $\bar{FC_{1}}$ is the log_2_ fold change in treated plants, $\bar{FC_{2}}$ is the log_2_ fold change in control plants, *n*_1_ is the number of treated plants, *n*_2_ is the number of control plants, *SD*_1_ is the standard deviation of treated plants, and *SD*_2_ is the standard deviation of control plants.

The numerator represented the difference between the means of *FC* ( $FC_{1\;})\;$ and *FC* $\left(FC_{2\;}\right)$ . The denominator represents the pooled *SD*. Because the number of samples in some studies included in the meta-analysis was<20, the correction factor, Hedges’s *g* (Lakens, 2013), was applied, as shown in Equation 2:


(2) $$Hedges^{\prime }s\;g=Cohen^{\prime }s\;d\;x\;\left(1-\frac{3}{4\left(n_{1}+n_{2}\right)-9}\right)$$

where $Cohen^{\prime }s\;d$ is the effect size calculated before applying the correction factor, *n*_1_ is the number of treated plants, and *n*_2_ is the number of control plants.

Moreover, the effect size error (*SE_ES_*) was determined (de Vries et al., 2022), as reported in Equation 3.


(3) $$SE_{ES}=\sqrt{\frac{n_{1}+n_{2}}{n_{1}\;n_{2}}+\frac{ES^{2}}{2\left(n_{1}+n_{2}\right)}}$$

where *ES* is the effect size, *n*_1_ is the number of treated plants, and *n*_2_ is the number of control plants.

### 2.6 Data analysis

A minimum of four articles were selected for each meta-analysis. The meta-analysis was performed using the software JASP 0.14 “Meta-analysis module” (JASP Team, 2023). A random-effects model considering the heterogeneity-assorted studies was chosen. The summary of results through forest and funnel plots using JASP was produced, and the heterogeneity of the included studies through forest plots and publication bias *via* funnel plots were assessed. The inconsistency or heterogeneity of the studies was estimated *via* an intraclass correlation index (*I* ^2^), which ranges from 0 to 1 (or 0 to 100%) and indicates how much the variation in effect size is due to the between-variance (*τ* ^2^) or, more generally, the proportion of variance not attributable to sampling (error) variance (Nakagawa et al., 2017). Publication bias was estimated using Egger’s test. Egger’s statistical test detects funnel plot asymmetry by determining whether the intercept deviates significantly from zero in a regression of standardised effect estimates *versus* precision (Hayashino et al., 2005). Bias can also be estimated using funnel plots (JASP Team, 2023), which are scatter plots of the treatment effects estimated from individual studies (horizontal axis) against the measure of study precision (vertical axis) (Sterne and Egger, 2001; Sterne et al., 2011).

## 3 Results

### 3.1 Study selection and data extraction

The individual bibliographic searches yielded 3,338 total reports through Google Scholar (2,769) and Scopus (569), following the PRISMA protocol, as shown in **Figure 1**. Among the records found, 271 duplicates were removed before the second screening phase. Then, title and abstract screening reduced records from 3,067 to 89, excluding reviews, non-original research articles, and studies regarding biotic stress. Subsequently, the remaining reports were analysed to assess their suitability for meta-analysis. The screening consisted of meeting the following inclusion criteria: *i*) studies on herbaceous plants, *ii*) number of individuals, *iii*) presence of data on selected gene expression in terms of log_2_ *FC* and assessed by quantitative PCR, *iv*) presence of dispersion indices such as *SE* or *SD*, and *v*) presence of a control to compare gene expression between control and treated plants. Finally, 41 studies were included in this meta-analysis.

After identifying the included studies, two types of databases were set up to organise the data extracted from the studies and calculate the effect size (**Table S11** and **Table S12**). **Tables S1–S9** show the organised data obtained from reports concerning studies on gene expression in crop plants inoculated with PGPR under drought conditions. **Table S10** shows the data on DREB2 expression in plants subjected to drought but not inoculated with PGPR. Initially, drought stress induced in plants by dehydration (lack of water) and polyethylene glycol (PEG) were considered for meta-analysis. Unfortunately, it was not possible to include *acdS*, *ERD15*, *WRKY*, *MYC*, and *MYB*, as there were insufficient eligible articles regarding the expression of these genes in plants inoculated with PGPR and exposed to water shortages.

### 3.2 Meta-analysis results

**Figure 2:**
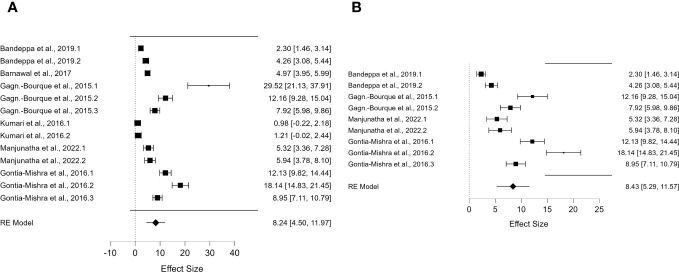
Forest plot of *DREB2* expression with PGPR in plants exposed to drought stress. **(A)** Forest plot of subgroups based on the drought stress treatment selected (dehydration). **(B)** Forest plot of subgroup analysis based on the drought stress treatment (dehydration) and the stressed plant organ (leaves). PGPR, plant growth-promoting rhizobacteria.

**Figure 3:**
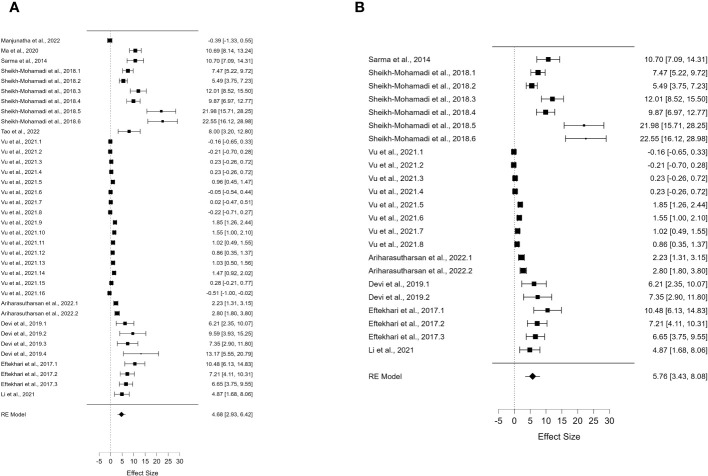
Forest plot of *DREB2* expression without PGPR in plants exposed to drought stress. **(A)** Forest plot of subgroups based on the drought stress treatment selected (dehydration). **(B)** Forest plot of subgroup analysis based on the drought stress treatment (dehydration) and the stressed plant organ (leaves). PGPR, plant growth-promoting rhizobacteria.

Our search strategy yielded 24 studies querying the expression of *ACS*, *ACO*, *APX*, and *DREB2* in plants subjected to drought stress and inoculated with PGPR and 17 studies querying *DREB2* expression in plants subjected to drought stress without PGPR. Firstly, an independent meta-analysis was performed using the software JASP 0.14 for each of the included genes (*ACS*, *ACO*, *APX*, and *DREB2*) that met the inclusion criteria. The analysis synthesised different information across studies on gene expression under water stress, with or without PGPR. **Table 1** summarises the results obtained from the meta-analysis for each gene, evidencing general pooled effect size, 95% confidence interval (CI), and pooled *I* ^2^ (%). Then, the effect size for each subgroup, 95% CI, and *I* ^2^ for each subgroup were obtained. Forest and funnel plots for *ACO* gene (**Figure S1**), *ACS* gene (**Figure S2**), *APX* gene (**Figure S3**), and *DREB2* gene (**Figure S4**) were obtained. Results for *ACO*, *ACS*, and *APX* genes were statistically insignificant since the 95% CI from the pooled estimated effect size crossed zero, and the heterogeneity was high. Therefore, a subgroup analysis based on specific and frequently analysed plant organs (i.e., leaves) was performed. Also, in this case, the results obtained for *ACO* (**Figure S5**), *ACS* (**Figure S6**), and *APX* (**Figure S7**) were statistically insignificant. In contrast, statistically significant results were obtained for *DREB2* with and without PGPR inoculation in plants subjected to drought stress (**Figures S4 and S8**). In these two analyses, the heterogeneity was considered very elevated because it was greater than 75% (Cohen, 1988). An *I* ^2^ value of 98.52% (**Table 1**) in *DREB2* expression in plants inoculated with PGPR during drought was obtained. The forest plot (**Figure S8**) and *I* ^2^ value of 99.248 (**Table 1**) for DREB2 expression in water-stressed plants and without PGPR showed high heterogeneity. Further subgroup analyses were performed to investigate the cause of this heterogeneity. The first was based on the specific method used for the stress treatment (dehydration) (**Figure 2A**) and on the plant organs analysed (leaves) (**Figure 2B**) in the presence of PGPR. The second method was based on the specific method used for stress treatment (dehydration) (**Figure 3A**) and on the plant organs analysed (leaves) (**Figure 3B**) but without PGPR.

**Table 1:** Summary of results obtained from the meta-analysis for each gene, namely, *1-aminocyclopropane-1-carboxylate (*ACO*)*, 1-*aminocyclopropane-1-carboxylate synthetase* (*ACS*), *ascorbate peroxidase (APX)*, and *dehydration-responsive element-binding protein 2 (DREB2)*.

Gene	Pooled *ES*	95% CI	Pooled *I* ^2^ (%)	Subgroups	*ES* for each subgroup	95% CI	*I* ^2^ for each subgroup
*ACO*	−0.27	[−1,33; 0.80]	90.968	Drought and leaf	−0.04	[−1.07; 0.99]	86.369
*ACS*	1.57	[−1.08;4.21]	97.838	Drought and leaf	0.41	[−1.01; 1.84]	92.202
*APX*	0.09	[−0.42; 0.60]	80.107	Drought and leaf	−0.11	[−0.58; 0.37]	76.636
*DREB2* *with PGPR*	9.14	[5.58; 12.70]	98.52	Dehydration	8.24	[4.50; 11.97]	98.66
				Dehydration and leaf	8.43	[5.29; 11.57]	96.681
*DREB2 without PGPR*	4.55	[3.57; 5.53]	99.25	Dehydration	4.68	[2.93; 6.47]	99.456
				Dehydration and leaf	5.76	[3.43;8.08]	99.380

The name of the gene, pooled effect size values, 95% confidence interval values, and *I* ^2^ index (also for the subgroup analysis) are listed. CI, confidence interval; *ES*, effect size; PGPR, plant growth-promoting rhizobacteria.

**Figure 4:**
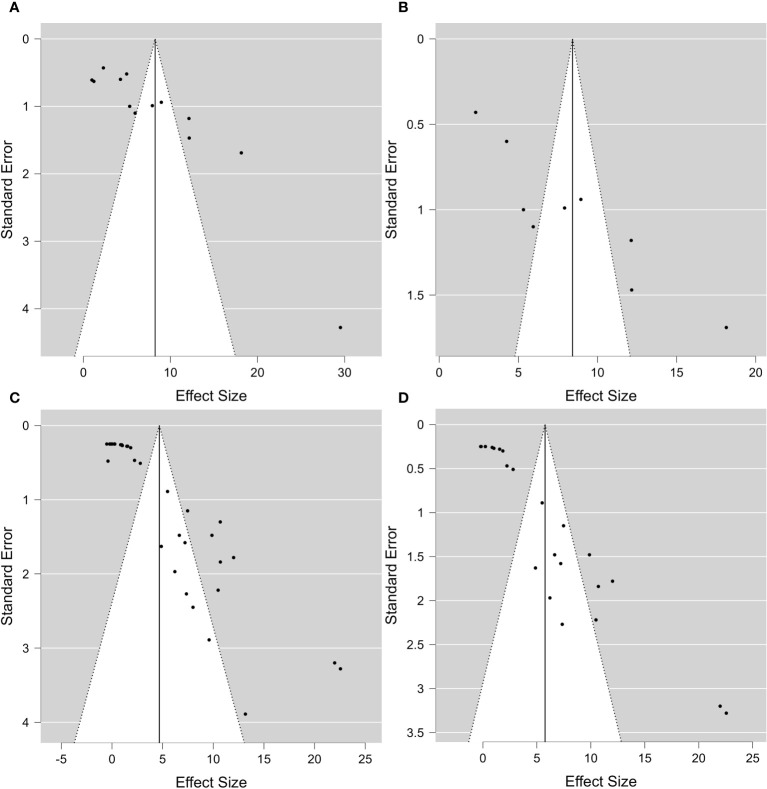
Funnel plot of *DREB2* expression with and without PGPR in plants exposed to drought stress. **(A)** Funnel plot of subgroup analysis based on the drought stress treatment selected (dehydration) and in the presence of PGPR. **(B)** Funnel plot of subgroup analysis based on drought stress treatment selected and the stressed plant organ (leaves) in the presence of PGPR. **(C)** Funnel plot of subgroup analysis based on the drought stress treatment selected (dehydration). **(D)** Funnel plot of subgroup analysis based on the drought stress treatment selected (dehydration) and the stressed plant organ (leaves). Analyses in panels **(C, D)** are independent of PGPR inoculation. PGPR, plant growth-promoting rhizobacteria.

For *DREB2*, thanks to the sufficient number of papers, it was possible to include further subgroup analyses. To allow the comparison of results and consistent assumptions, the minimum number of articles was considered to be at least four for each subgroup chosen. In particular, for *DREB2* with PGPR and *DREB2* without PGPR inoculation, the number of studies selected should be the same. In the first case, in the subgroup analysis of *DREB2* during dehydration of the entire plant or in different districts and the presence of PGPR, the *I* ^2^ increased to 98.665% (**Figure 2**). The subgroup analysis based on dehydration stress (**Figure 2A**) and drought stress applied to leaves (**Figure 2B**) in the presence of PGPR showed a decrease in heterogeneity of 96.68% but remained too high for consideration. Therefore, a subgroup analysis for *DREB2* expression without PGPR was performed (**Figure 3**). Both forest plots based on general dehydration (**Figure 3A**) and dehydration in leaves (**Figure 3B**) showed a decrease in heterogeneity; in particular, the *I* ^2^ value reached 98.66% for general dehydration and 96.68% for dehydration applied to leaves. In conclusion, significant heterogeneity was found in each summary effect, which could not be avoided by subgroup analysis. Furthermore, by observing the cumulative *ES* values, it can be assumed that the expression of the gene encoding *DREB2* was positively regulated during drought stress, in both the presence and absence of PGPR (**Table 1**). Nonetheless, the cumulative effect size values for *DREB2* in the presence of PGPR (*ES* = 9.14; 95% CI 5.58, 12.70) were higher than those of *DREB2* in the absence of PGPR inoculation (*ES* = 5.76; 95% CI 3.43, 8.08). Funnel plots of subgroup analysis of *DREB2* expression based on drought stress treatments and in the absence or presence of PGPR inoculation are shown in **Figure 4**. All funnel plots were asymmetrical along the vertical axis around the meta-analytical pooled effect size estimate. Moreover, almost all the points representing each study were external to the 95% confidence intervals. A significant publication bias in all analyses of *DREB2* expression with and without PGPR in plants exposed to drought stress and their subgroups was identified *via* Egger’s test (p< 0.01). Evident asymmetry, confirmed *via* Egger’s test, could be interpreted as evidence of publication bias and heterogeneity.

## 4 Discussion and conclusion

Climate change has exacerbated the water crisis in the Mediterranean region, affecting agricultural productivity and worsening land overexploitation. Therefore, scientists are challenged to develop new strategies for maintaining sustainable agriculture and coping with the current increase in drought stress. Several recent studies have shown that the association between plants and PGPR is important for resistance and adaptation to abiotic and biotic stresses. Among them, drought stress can be relieved by the presence of positive bacteria at the physio-morphological level, increasing root volume, enhancing nutrient mobilisation and up-taking, and over-regulating antioxidant enzymes, such as ascorbate peroxidase, catalase, superoxide dismutase, ascorbic acid, glutathione, α-tocopherol, and glutathione reductase (Bukhat et al., 2020; Goswami and Deka, 2020; Ahluwalia et al., 2021). Inter-kingdom communication causes the upregulation of drought-response marker genes such as *DREB1B-like*, *ERD15* (Naylor and Coleman-Derr, 2018), and *DREB2A* (Sarma and Saikia, 2014). The molecular mechanism underlying the plant–PGPR interactions has deepened thanks to advances in molecular techniques, such as the analysis of differential gene expression using cDNA microarrays (van Loon, 2007), qPCR (Ibort et al., 2018), and RNA-seq (Tao et al., 2022). Recent molecular studies have shown that PGPR affects the expression patterns of stress-responsive genes in plants, alleviating the damaging effects of drought stress (Vurukonda et al., 2016; Gupta et al., 2021; Manjunatha et al., 2022).

Saikia et al. (2018) studied *Vigna mungo* L. and *Pisum sativum* L. subjected to water stress and inoculated them with a combination of three ACC deaminase-producing rhizobacteria (*Ochrobactrum pseudogrignonense* RJ12, *Pseudomonas* sp. RJ15, and *Bacillus subtilis* RJ46) (Saikia et al., 2018). This study showed that inoculated plants showed a lower amount of *ACC* than the control due to the upregulation of *ACS* and the downregulation of *ACO* genes, consequently decreasing ethylene production (Saikia et al., 2018). Moreover, several studies have argued that the expression of genes encoding *MYC*, *MYB*, and *WRKY* is influenced by plant–microorganism interactions during abiotic stress (Dimkpa C. et al., 2009; Arora et al., 2020; Ha-Tran et al., 2021; Hoque et al., 2023; Rosier et al., 2018; Khan et al., 2020; Oleńska et al., 2020). Kasim et al. (2013) reported that wheat plants inoculated with strains of *Bacillus amyloliquefaciens* and *Azospirillum brasilense* and subjected to drought showed an upregulation of *APX1* and an increase in the activity of enzymes involved in the ascorbate-glutathione redox cycle, affecting reactive oxygen species (ROS) scavenging (Kasim et al., 2013). Similarly, *ERD15* gene expression was upregulated in *Arabidopsis thaliana* inoculated with *Paenibacillus polymyxa* strains under water shortage (Timmusk and Wagner, 1999). In addition, the upregulation of *DREB2* and dehydrins in *Vigna radiata* L. inoculated with *Pseudomonas aeruginosa* GGRJ21 was observed (Sarma and Saikia, 2014).

To further investigate the mechanisms described in these studies and explore the role of PGPR in plant drought stress-responsive genes, a meta-analysis was performed to quantitatively synthesise the data available in the literature. The focus was on the bacterial gene *acdS* and the plant genes *ACO*, *ACS*, *APX*, *WRKY*, *MYC*, *MYB*, *ERD15*, and *DREB2* to evaluate whether and how they are influenced by the presence of PGPR. These genes are specifically involved in metabolic pathways activated in plant responses to abiotic stress conditions, especially during water shortage. An independent meta-analysis was performed for each gene. Relevant articles were insufficient for *acdS*, *MYC*, *MYB*, *WRKY*, and *ERD15* to proceed with the analysis. The primary obstacle to conducting a meta-analysis for these genes stemmed from the insufficient number of studies providing the log_2_ fold change, a crucial parameter for our analysis. Additional challenges included the absence of standard deviation (*SD*) or standard error (*SE*) values for the relative transcript accumulation of drought-responsive genes. To address this, the prognostic method was employed for *DREB2*, *ACO*, *ACS*, and *APX* genes. It is essential to underscore that *SD* and *SE* are pivotal values in a study, playing a crucial role in determining the effect size in meta-analysis.

Despite the scarcity of data preventing a comprehensive meta-analysis for *acdS*, *MYC*, *MYB*, *WRKY*, and *ERD15* genes, we conducted a meticulous systematic review to gather insights into the expression and roles of these genes in response to drought stress in PGPR-treated plants. Although quantitative synthesis was impossible due to limited available studies, we excluded numerous *acdS* gene studies for lacking log_2_ fold change reporting. Many of these studies validated the presence of the ACC deaminase gene (*acdS*) through PCR in bacterial strains from the rhizosphere of plants like *Cyamopsis tetragonoloba* and *Pennisetum glaucum* L., showcasing their potential as PGPR in enhancing plant growth under water stress conditions (Goyal et al., 2022; Murali et al., 2021). It is widely recognized that microorganisms can reduce ethylene levels in the plant through the ACC deaminase enzyme. This hormone plays a crucial role in plant defence responses to abiotic stresses, limiting root and shoot growth (Selvakumar et al., 2012; Goswami and Deka, 2020). For instance, in *V. mungo* L. and *P. sativum* L. treated with a consortium of three ACC-deaminase-producing rhizobacteria, there was a reduction in deleterious stress ethylene accumulation during drought stress (Saikia et al., 2018). *MYB* transcription factors are known to be involved in plant drought responses, impacting development, metabolism, and stress regulation (Baldoni et al., 2015). However, the effect of drought on *MYB* gene regulation seems to vary based on plant species and organs (Baldoni et al., 2015). From our systematic review, only two articles regarding *MYB* expression in two different crop species inoculated with PGPR could have been included in the meta-analysis (Cho et al., 2013; Kakar et al., 2016). In *Oryza sativa* L. subjected to water stress and treated with a consortium of *B. amyloliquefaciens*–*Bacillus methylotrophicus*–*Brevibacillus laterosporus*, *OsMYB3R-*2 was upregulated, and plants showed improved seedling height and shoot number compared to control plants (Kakar et al., 2016). Conversely, in *A. thaliana* inoculated with *Pseudomonas chlororaphis* during water stress, *MYB* genes were downregulated (Cho et al., 2013). When water was suspended, *Arabidopsis* plants without inoculation showed a decrease in survival after 14 days of drought stress. The survival rate notably dropped in control plants on days 15 and 16 of the drought treatment. Conversely, plants inoculated with *P. chlororaphis* O6 did not display such a decline in viability when water was withheld (Cho et al., 2013). Only one study was suitable for meta-analysis regarding *MYC* gene, which is one of those found for *MYB* (Cho et al., 2013). Similarly, *MYC* genes, belonging to the basic helix-loop-helix (bHLH) transcription factor family, exhibited downregulation during water stress (Feng et al., 2023; Cho et al., 2013). *WRKY* transcription factors play a vital role in regulating plant stress responses. Overexpression of specific *WRKY* genes has shown increased tolerance to heat, drought, and salinity in various species (Tripathi et al., 2014). The only two *WRKY*-related studies that met the inclusion criteria for the meta-analysis showed that under drought stress conditions, in tomato inoculated with *Streptomyces* strains and *Bacillus megaterium*, *WRKY70*, *SlWRKY75*, and *SlWRKY45* were downregulated (Abbasi et al., 2020; Morcillo et al., 2021). This significantly impacted the fruit weight of tomato plants subjected to water stress and treated with these strains (Abbasi et al., 2020). *ERD15* was initially characterized as a rapidly drought-responsive gene in *Arabidopsis*; it has recently been reported as a negative regulator of ABA, which can also be induced by salicylic acid (SA), wounds, and pathogenic infections to mediate interactions between abiotic and biotic stress responses (Shao et al., 2014). Unfortunately, for ERD15, we did not find any articles that met the inclusion criteria.

The meta-analysis conducted on *ACO*, *ACS*, and *APX* showed non-significant results, considering that the confidence intervals of the summary effects overlapped by zero (Dong et al., 2017). *ACO* and *ACS* participate in the biosynthesis of ethylene, while *APX* gene codes for the ascorbate peroxide antioxidant enzyme, regulating the *ROS* defensive mechanism in plant cells in response to abiotic stress (Selvakumar et al., 2012; Nadeem et al., 2019; Goswami and Deka, 2020). From these results, it can be speculated that the lack of statistically significant differences in the expression of these genes, with or without PGPR, was due to the insignificant overall effect size. However, studies included in the meta-analysis for these genes highlighted a significant improvement in plants treated with PGPR during drought stress compared to the control (Kasim et al., 2013; Saikia et al., 2018). Saikia et al. (2018) conducted a study on *V. mungo* L. and *P. sativum* L. subjected to water stress and inoculated with a consortium of three ACC deaminase-producing rhizobacteria (*O. pseudogrignonense* RJ12, *Pseudomonas* sp. RJ15, and *B. subtilis* RJ46) (Saikia et al., 2018). This study demonstrated that inoculated plants showed a lower amount of *ACC* compared to the control due to the upregulation of *ACS* and the downregulation of *ACO* gene, consequently decreasing ethylene production (Saikia et al., 2018). Moreover, Kasim et al. (2013) reported that wheat plants inoculated with strains of *B. amyloliquefaciens* and *A. brasilense* during drought stress exhibited an upregulation of *APX1* and increased activity of enzymes involved in the ascorbate-glutathione redox cycle, impacting *ROS* scavenging (Kasim et al., 2013). Therefore, we also hypothesized that the statistically insignificant results might be attributed to heterogeneity, even if we could not investigate its sources through subgroup analysis (insufficient number of articles: four for *ACO* and *ACS* and five for *APX*). Variability can arise from mathematical–statistical causes (statistical heterogeneity) and also from the evaluation of plants with different characteristics, treatments, or their assessment (clinical heterogeneity) (Egger et al., 2022). We speculated that the main source of heterogeneity could be attributed to the different induction stress methods used in studies included in the meta-analysis. In fact, the included studies for *ACO* and *ACS* gene meta-analysis used different treatments: Saikia et al. (2018); Sandhya and Ali (2018), and Tiwari et al. (2016) employed PEG 6000, while SkZ et al. (2018) induced drought stress by discontinuing water after 14 days of planting. Similarly, studies analysing *APX* exhibited differences: Gururani et al. (2013) and Tiwari et al. (2016) used PEG to induce drought stress, while Murali et al. (2021) and Singh et al. (2020) induced drought stress by withholding water. The divergence in the stress induction method (using PEG or withholding water) leads to differences in stress precision and practicality, influencing the plants’ responses, and this could be considered a source of heterogeneity in our meta-analysis (Krizek, 1985). DREBs (dehydration-responsive element binding) belong to the ERF family and the ABA-independent signal transduction pathway (Akbudak et al., 2018). Specifically, *DREB2A* plays a key role in plants’ drought response, primarily functioning in ABA-independent water stress-induced gene expression (Liu et al., 1998). In the first analysis, the expression of *DREB2* in the absence of PGPR and stress versus the presence of PGPR under drought stress conditions was highlighted as a statistically significant result. The cumulative *ES* values in the forest plot clearly indicate *DREB2* overexpression. In the second analysis regarding the expression of *DREB2* in the absence of PGPR and stress versus the presence of drought stress, the absence of PGPR again highlighted a statistically significant result. Indeed, the cumulative *ES* values in the forest plot indicate *DREB2* overexpression. Subsequently, the results matched. This comparison showed a statistically insignificant overexpression of *DREB2* in the presence of PGPR compared to the results obtained for crops not treated with PGPR but still stressed due to drought. Indeed, by analysing the resulting forest plots (**Supplementary Figures** and **Supplementary File 2**), an overlap of the cumulative effect size was noticeable. Therefore, our data showed that *DREB2* was overexpressed in plants subjected to drought stress, regardless of PGPR inoculation. In the former case, the gene expression level seemed higher, highlighting the slight effect that the bioinoculation of PGPR had on *DREB2* expression in plants, but this was not statistically significant. However, studies included in the meta-analysis, which investigated the expression of *DREB2* in PGPR-inoculated and drought-stressed plants, demonstrated positive enhancements in terms of dry weight. For instance, *Brassica juncea* L. inoculated with osmotolerant strains like *Bacillus* sp. MR D17 and *Bacillus cereus* NA D17, *Glycine max* L. Merrill treated with *Pseudomonas simiae*, and *Triticum aestivum* inoculated with *Dietzia natronolimnaea* and *B. subtilis* LDR2 all showed substantial increases in dry weight in response to drought stress (Barnawal et al., 2017; Bandeppa et al., 2019; Vaishnav and Choudhary, 2019). Despite employing a random effects model and conducting subgroup analysis, we were unable to diminish the high heterogeneity. The sources of heterogeneity in this study were as follows:

- Treatments used for stressed plants. In some cases, water shortage or dehydration was caused by not administering water for a certain time interval (e.g., Gontia-Mishra et al., 2016; Sheikh-Mohamadi et al., 2018; Vu et al., 2021), while in other cases, PEG was utilised to provide the drought stress (DS) (e.g., Vaishnav and Choudhary, 2019; Zhang et al., 2022). In two out of eight articles included in the meta-analysis for *DREB2* in PGPR-inoculated plants (Sarma and Saikia, 2014; Vaishnav and Choudhary, 2019), the authors decided to induce DS with PEG. Therefore, a subgroup analysis based on the water suspension method used to induce DS was performed. Nevertheless, heterogeneity did not diminish sufficiently, allowing us to hypothesise dependence on other variables.
- Unavailability of a common protocol to stress the plant and measure the relative expression of genes. This reduces the number of data points subjected to comparison.
- Choice of native and non-native PGPR strains for plant inoculation. Indeed, in some studies, plant non-native PGPR (Gagné-Bourque et al., 2015; Barnawal et al., 2017; Bandeppa et al., 2019) were used. In other works, such as Sarma and Saikia (2014); Vaishnav and Choudhary (2019), and Gontia-Mishra et al. (2016), plant-native PGPR were inoculated. This aspect is relevant because plant-native microbiota originated through a specific selection process that occurred during evolution, overcoming issues in persistence in the plant and rhizosphere with an adaptation process (Banerjee et al., 2017). Thus, the utilisation of non-native PGPR may affect the expression of plant genes.
- Different plant organs are also subjected to stress. In some cases, the relative expression level of drought stress-related genes is derived from the seedling, whereas in others, it is derived from the root.
- Although all of the studies considered were conducted under controlled conditions, the biometric parameters used for plant growth evaluation and the soil used for cultivation may differ slightly.
- The limitations of the sample in this study may have overestimated the treatment effects and contributed to statistical heterogeneity (Zhang et al., 2013). In some cases, there were several hundred replicates, whereas in others, there were three replicates.
- Gene expression can be induced in different ways during drought stress. Studies did not measure the same time-lapse. *DREB2*, for instance, is expressed within a range of a few hours; it starts to increase 10 min after stress treatment and reaches its maximum after 10 h of stress (Nakashima and Yamaguchi-Shinozaki, 2006).

Finally, it is crucial to mention the publication bias evident in the asymmetry of the funnel plots for *DREB2*, *ACO*, *ACS*, and *APX*. Publication bias reflects the tendency to publish studies on the direction of results; namely, it is more probable that studies with statistically significant or positive results will be published and accepted by journals6 than studies showing statistically insignificant or negative results (Song et al., 2013; Olsson and Sundell, 2023), affecting a clear and exhaustive panel of the resulting data. Indeed, it is widely assumed that negative studies that appear to be conducted better than positive ones are much less likely to be accepted for publication (Thornton and Lee, 2000). Some relevant biases concerning scientific literature include time lag, outcome reporting, grey literature, full publication, language, citation, and media-attention biases (Song et al., 2013; Olsson and Sundell, 2023). According to Olsson and Sundell (2023), every type of bias negatively affects the effectiveness of research synthesis concerning biased literature. This can significantly alter the process of effect estimation in a meta-analysis (Thornton and Lee, 2000). In fact, as suggested in the guidelines of the PRISMA statement (Moher et al., 2009), we excluded all non-English-language articles and the “grey literature” such as dissertations, conference proceedings, and congresses. The results reported in the “grey literature” may have limited accessibility, thereby complicating their immediate use with respect to results from peer-reviewed journals (Song et al., 2013). The asymmetry of the funnel plots obtained from our analyses could be due to both the high heterogeneity and publication bias of the examined studies, generating asymmetries due to data irregularities, artefacts, and chances (Nakagawa et al., 2022).

In conclusion, meta-analysis is an enormously powerful and extremely useful tool in many scientific fields and serves as a synopsis of a research question that provides a quantitative assessment of the relationship between two target variables. The purpose is twofold: it can bring out a unique conclusion on a topic and open science to new and more specific research questions. Meta-analyses are also recommended to identify topics for which the available data are insufficient and further studies are required. In light of our findings, plants inoculated with PGPR did not show observable changes in the expression of the defence-related genes *acdS*, *ACS*, *ACO*, *APX*, and *DREB2*. These results could depend on the three pivotal challenges faced in this study: lack of data, heterogeneity of studies, and publication bias. However, in the case of any pathogenic attack, rhizobacteria-treated plants showed a strong and quick response compared to the control plants, as confirmed in another meta-analysis (Bukhat et al., 2020). Considering that our results did not show a clear ability of PGPR to influence plant gene expression and promote growth under water stress, further investigation is fundamental. It is important for future work to deepen the understanding of the aspects related to signal sensing, perception, and the metabolic pathways involved in *DREB2* expression and the expression of other plant-responsive genes under drought stress. Considering the limitations of the literature, our results emphasise the importance of establishing common and shared protocols and standardising treatments to facilitate reproducibility and statistical analysis.

## Data availability statement

The original contributions presented in the study are included in the article/**Supplementary Material**. Further inquiries can be directed to the corresponding author.

## Author contributions

RF: Data curation, Formal analysis, Investigation, Writing – original draft. CC: Data curation, Formal analysis, Investigation, Writing – original draft. CV: Funding acquisition, Project administration, Supervision, Writing – review & editing. AC: Writing – review & editing. CG: Writing – review & editing. DP: Conceptualization, Funding acquisition, Methodology, Project administration, Supervision, Writing – review & editing.
