# Supplementary figures and images for: Meta-analysis of plant growth-promoting rhizobacteria interaction with host plants: implications for drought stress response gene expression
Source: Front Plant Sci. 2024 Jan 15;14:1282553. doi: 10.3389/fpls.2023.1282553 (PMC10823023; doi:10.3389/fpls.2023.1282553)

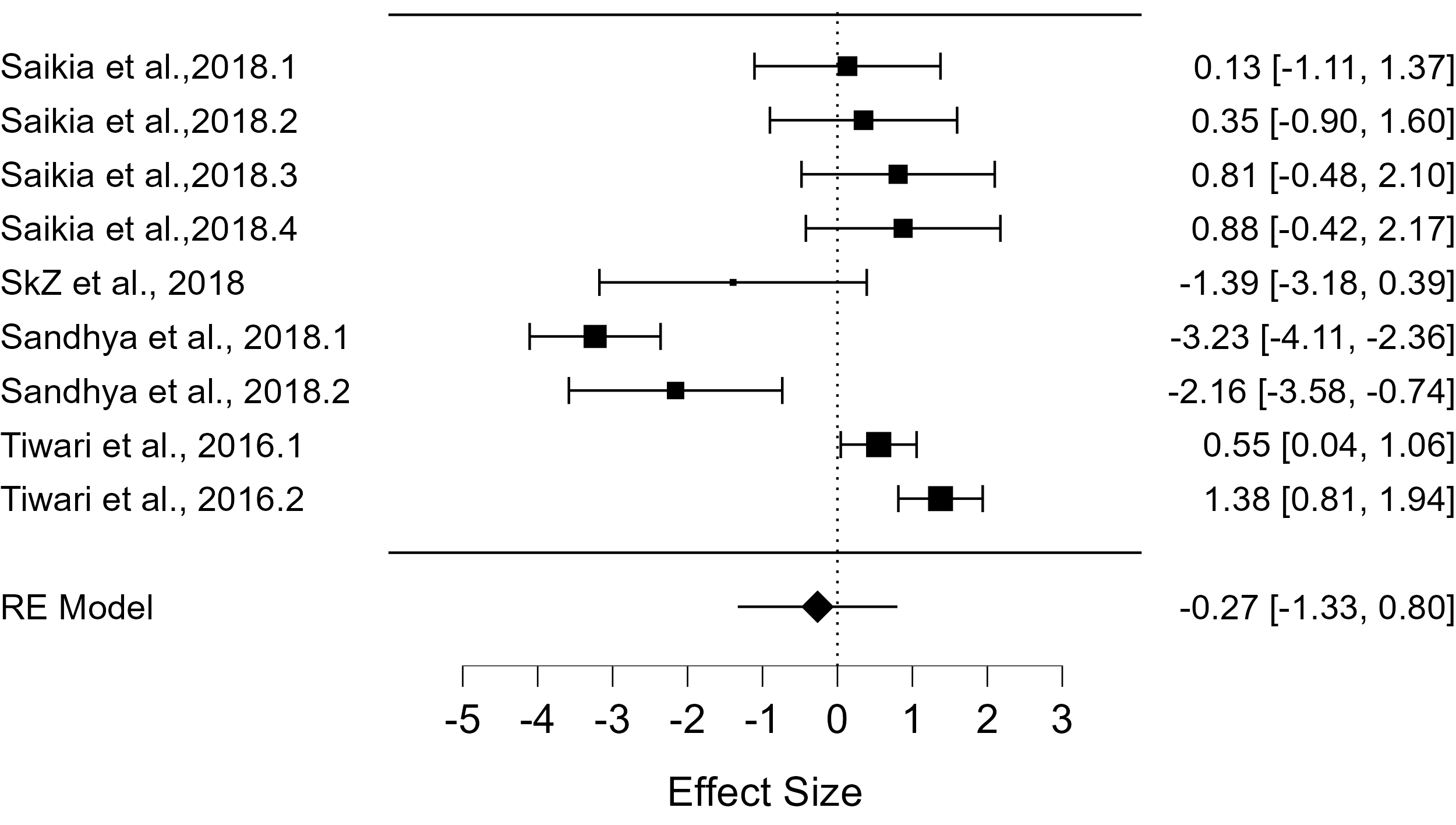

Supplement: Supplementary file 1 [file Image_1.tiff]

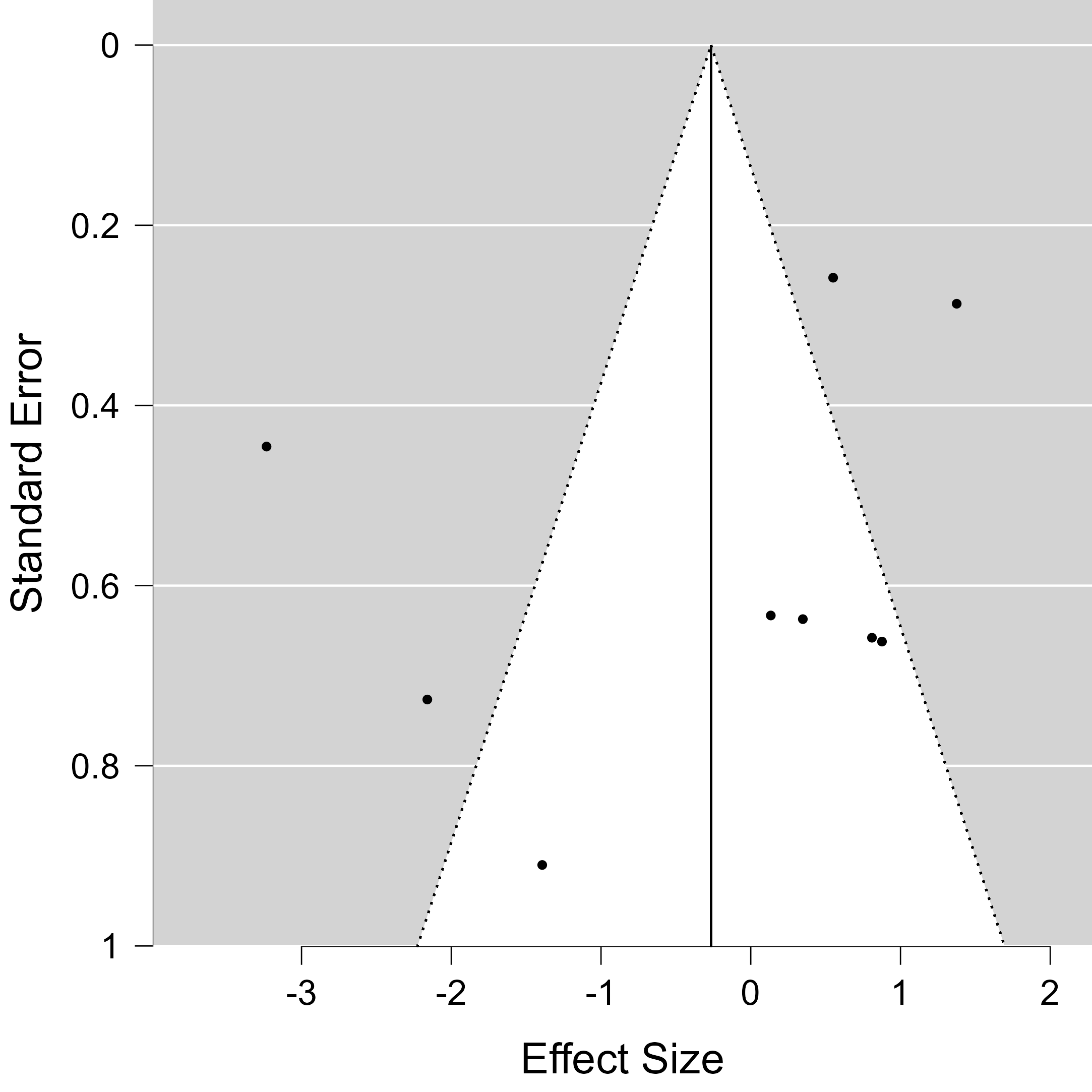

Supplement: Supplementary file 2 [file Image_2.tiff]

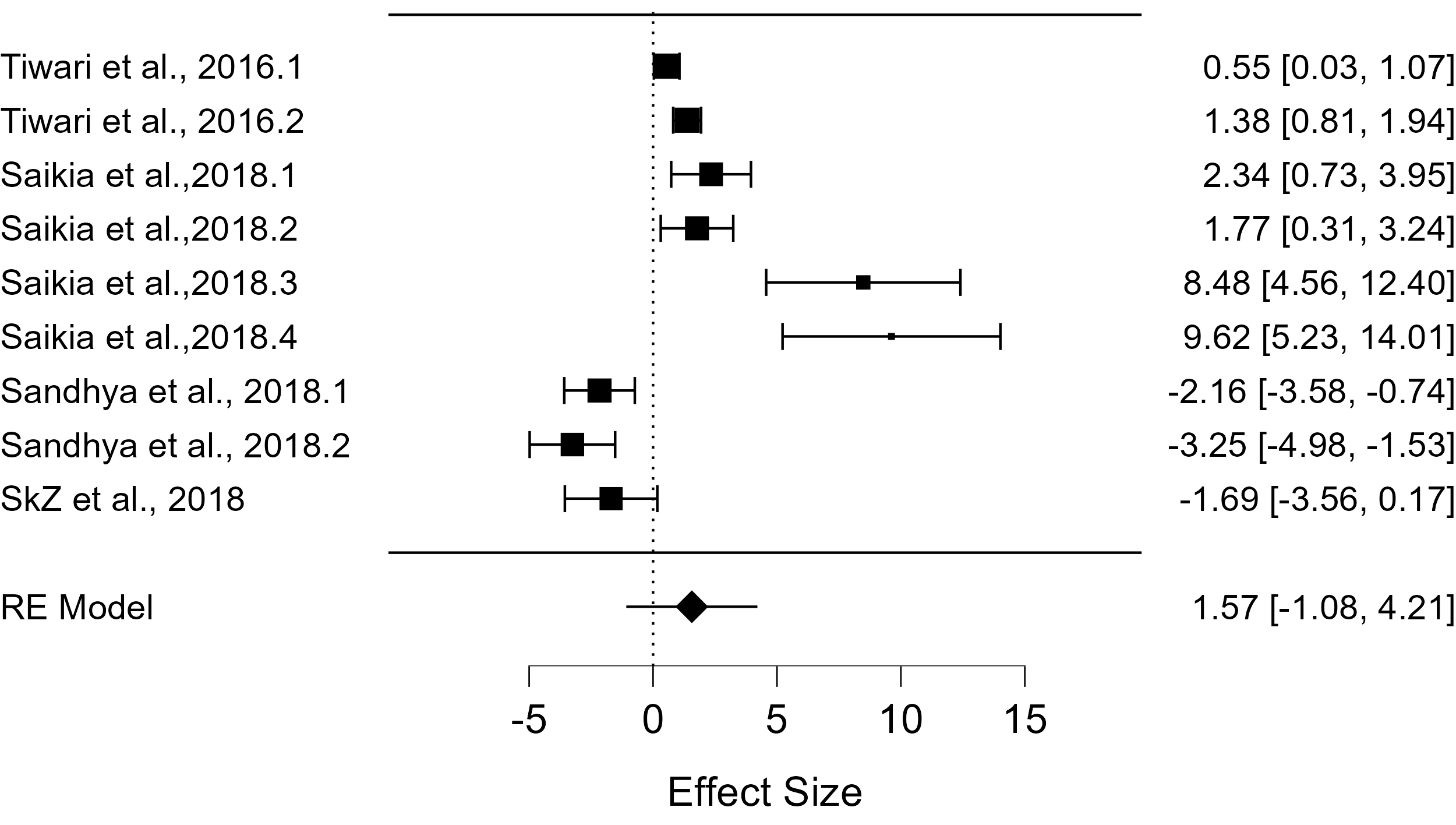

Supplement: Supplementary file 3 [file Image_3.tiff]

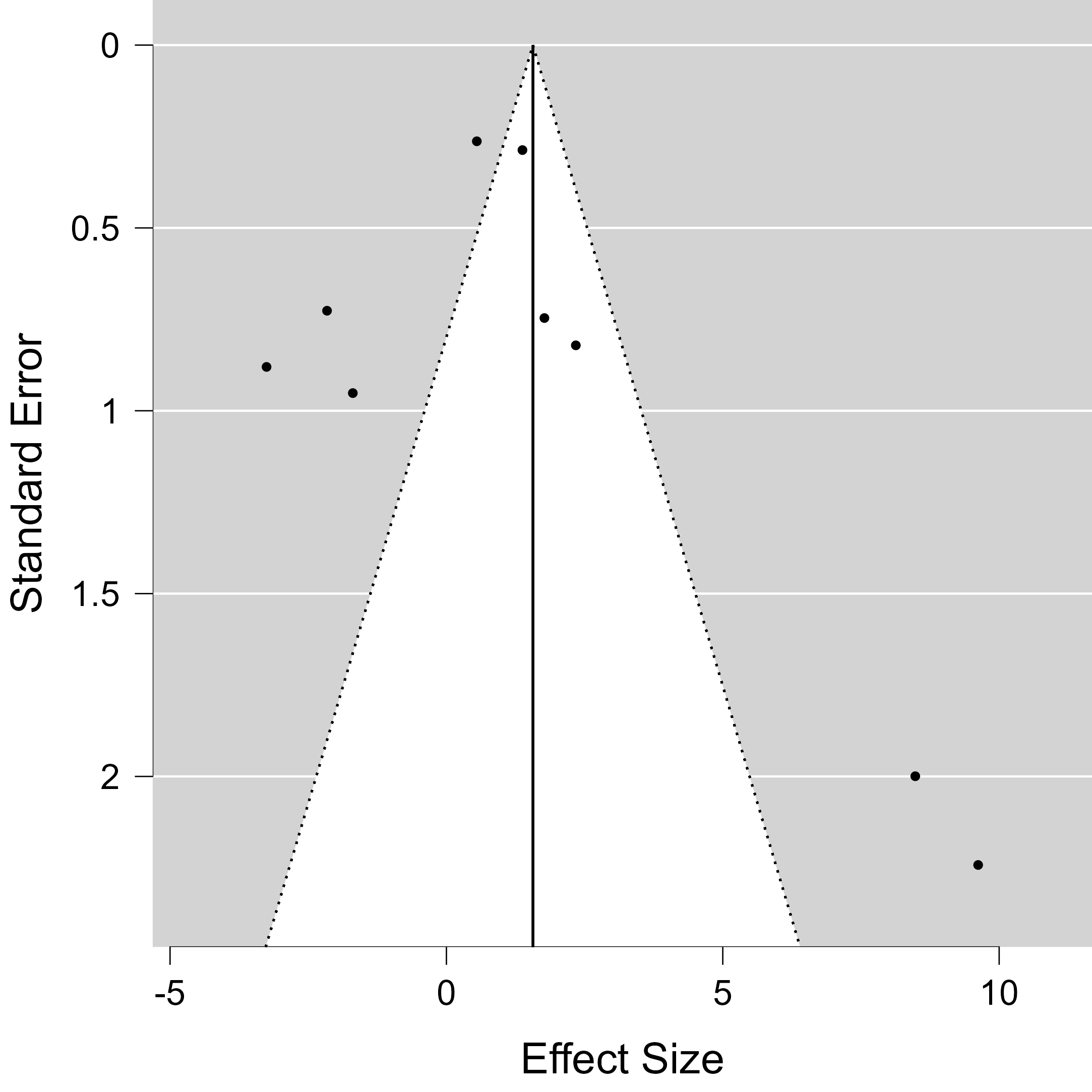

Supplement: Supplementary file 4 [file Image_4.tiff]

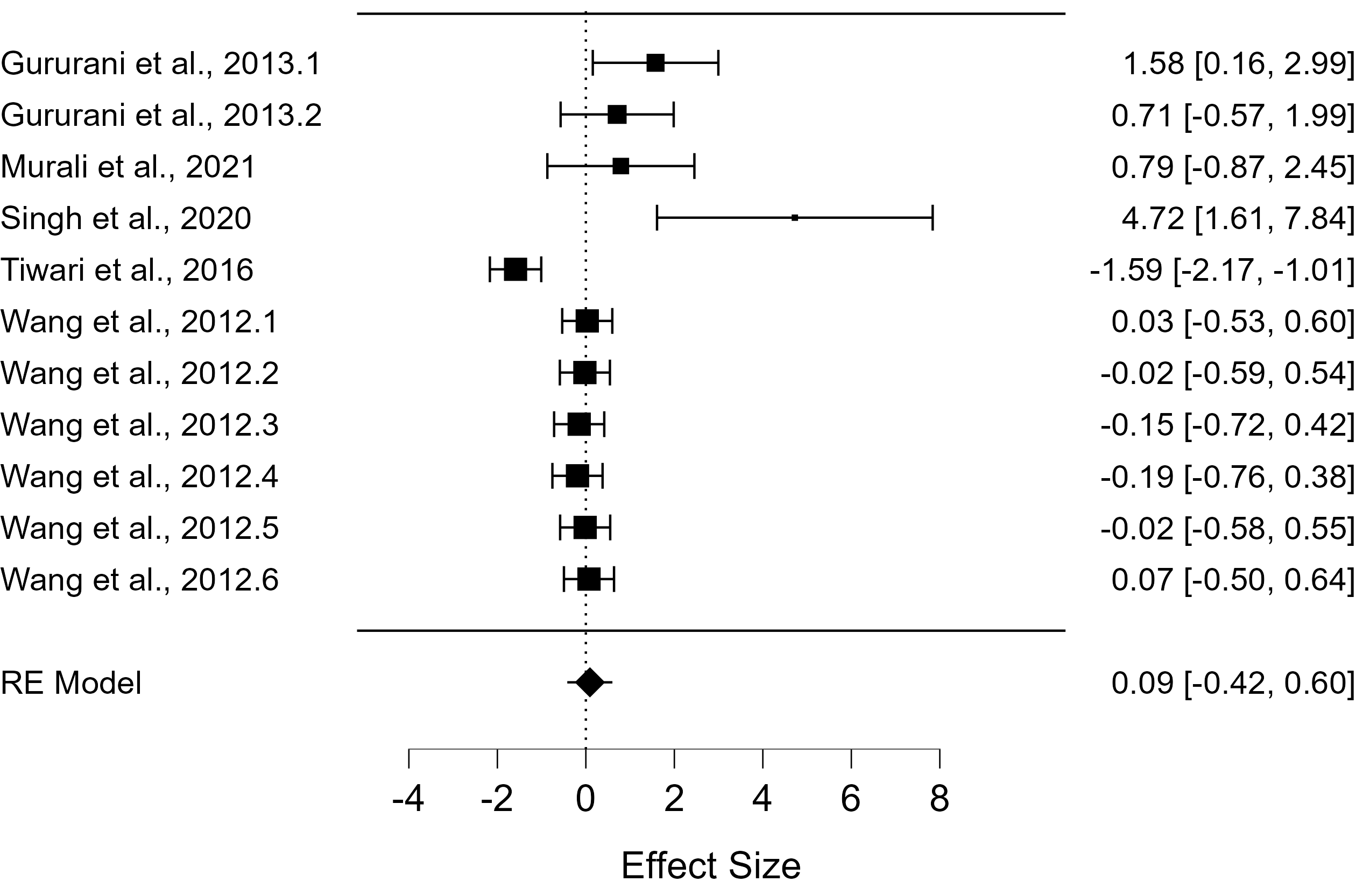

Supplement: Supplementary file 5 [file Image_5.tiff]

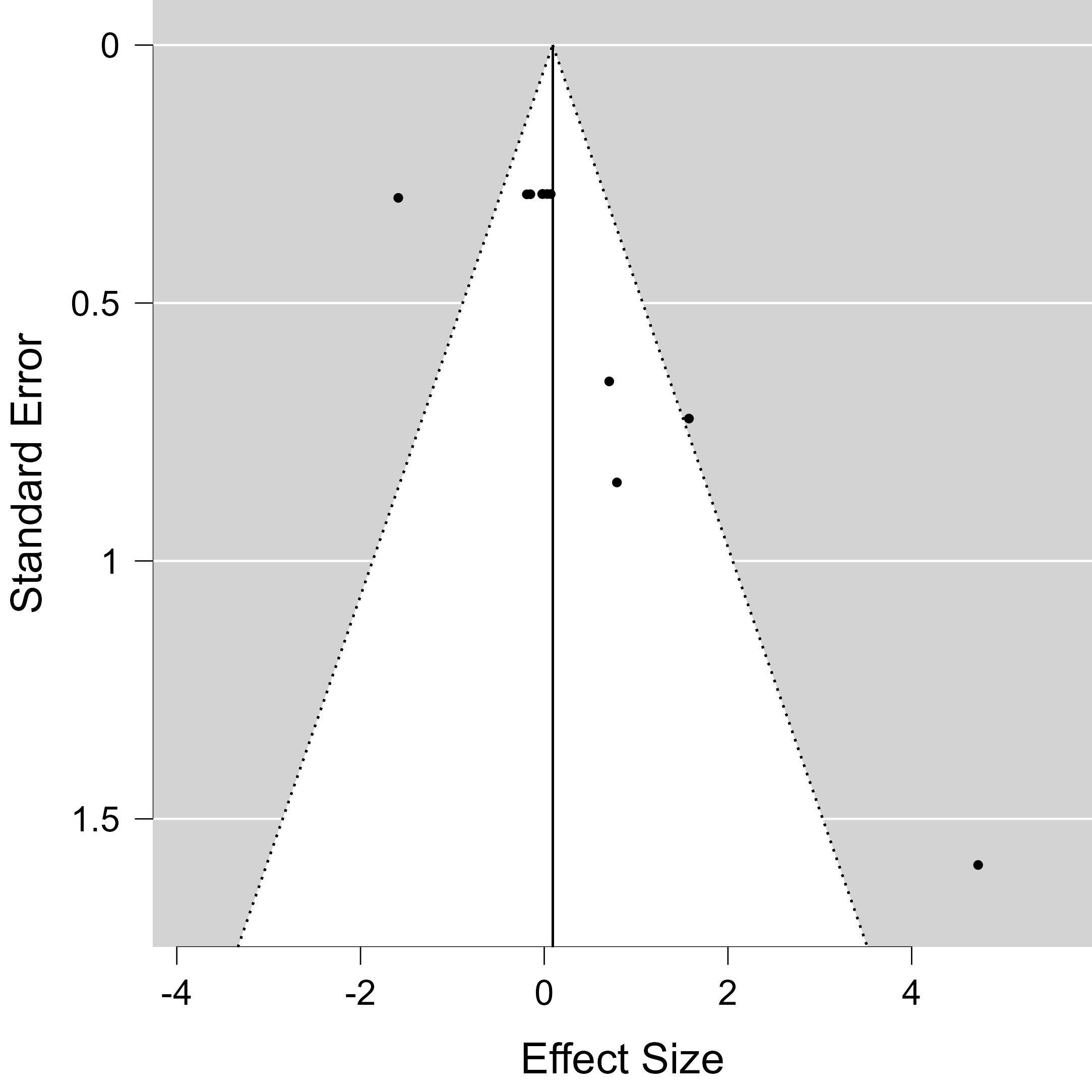

Supplement: Supplementary file 6 [file Image_6.tiff]

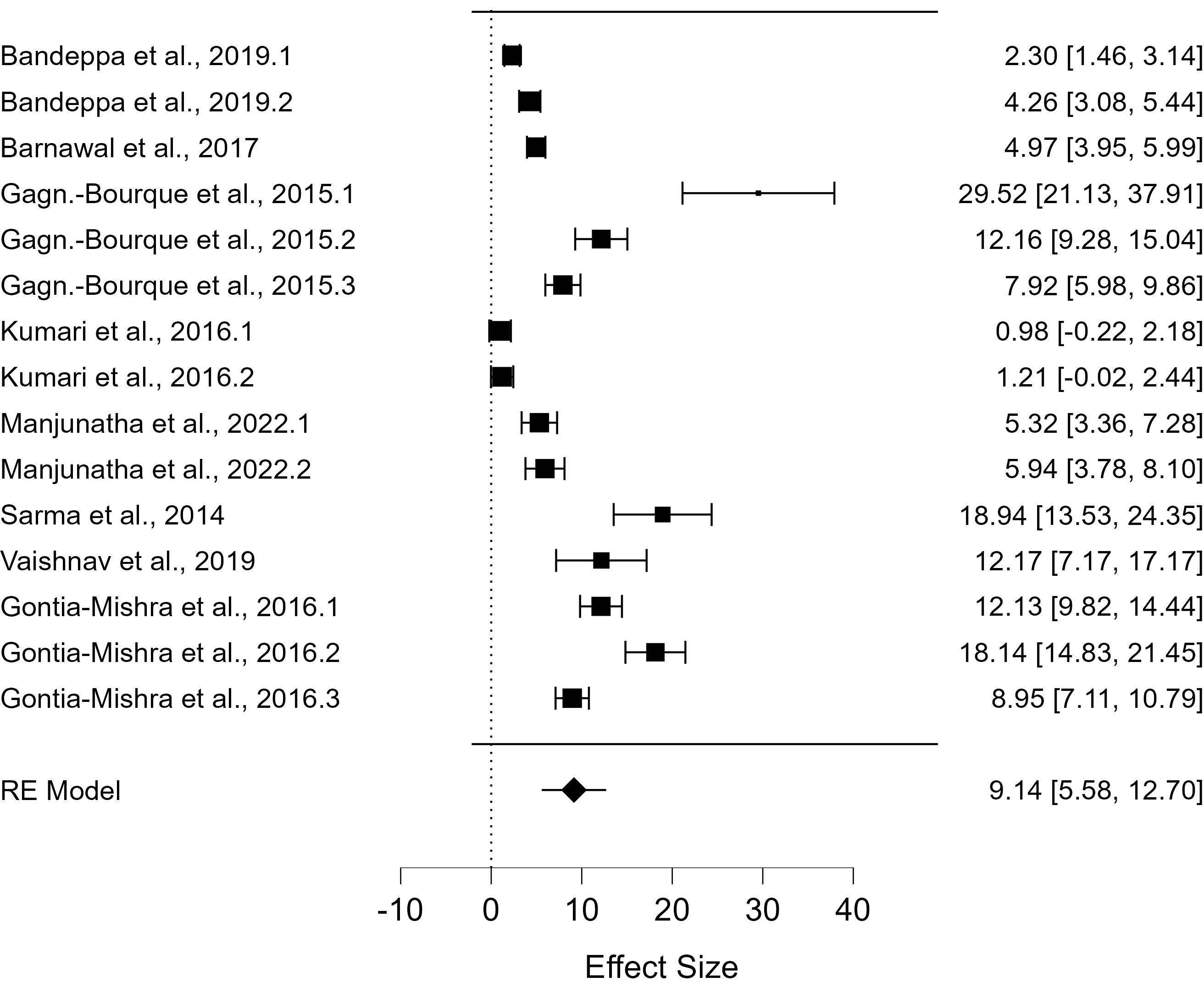

Supplement: Supplementary file 7 [file Image_7.tiff]

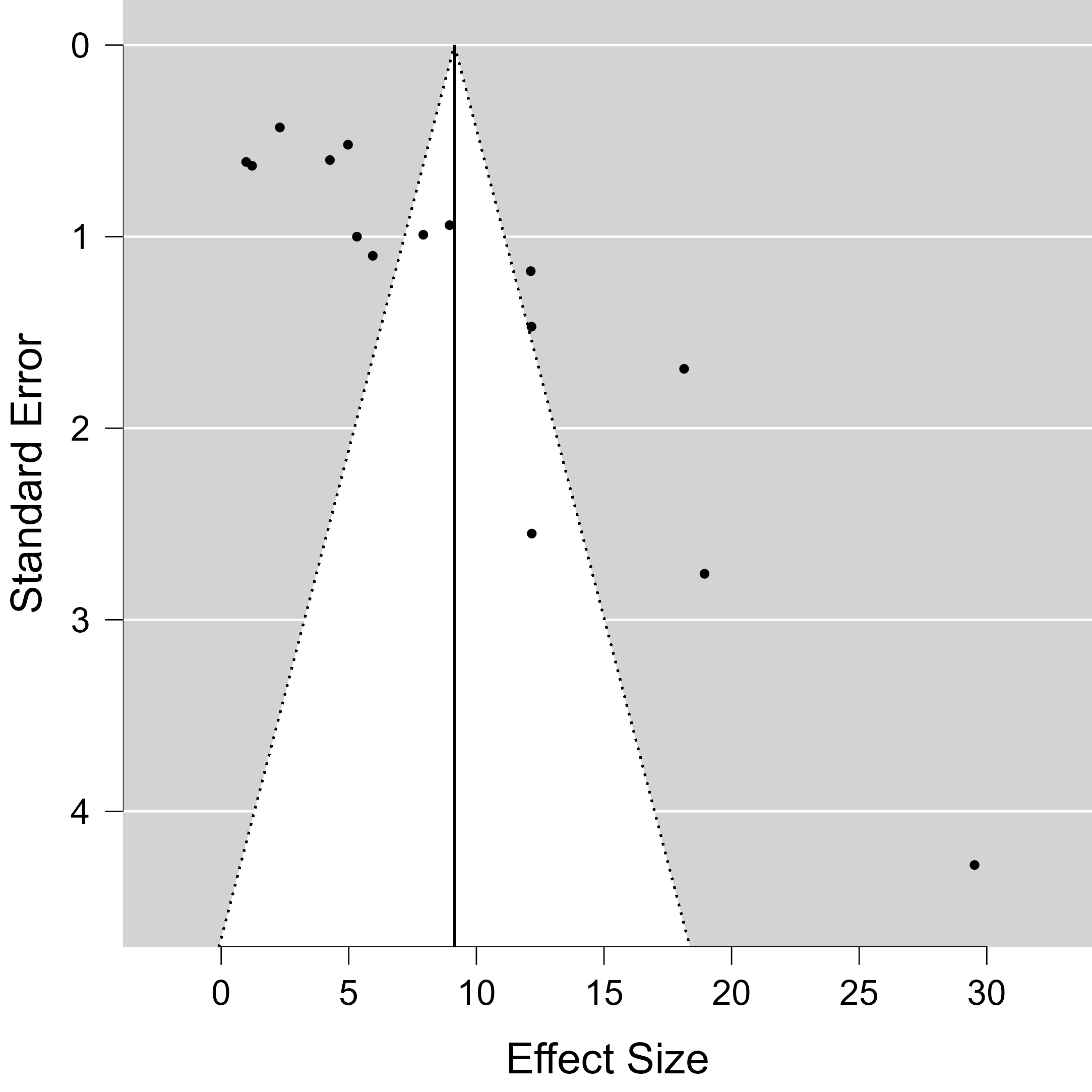

Supplement: Supplementary file 8 [file Image_8.tiff]

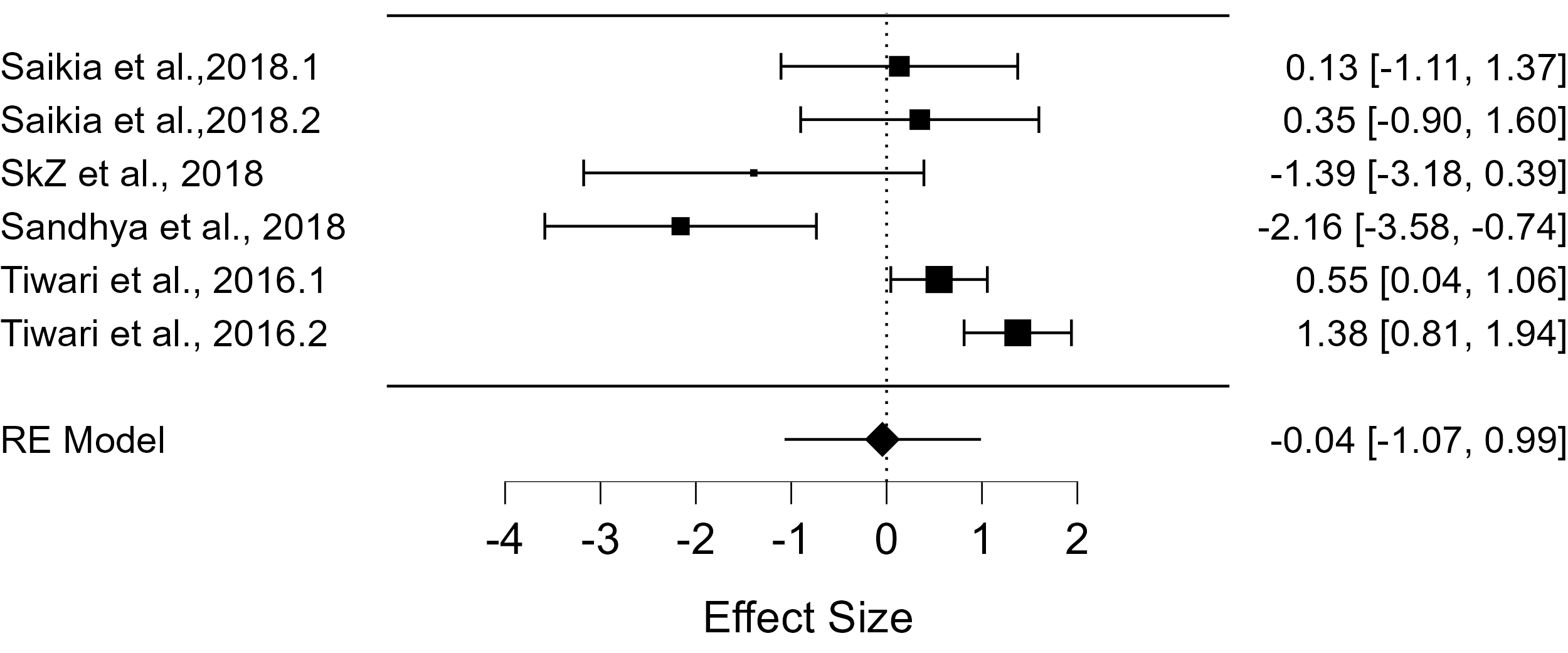

Supplement: Supplementary file 9 [file Image_9.tiff]

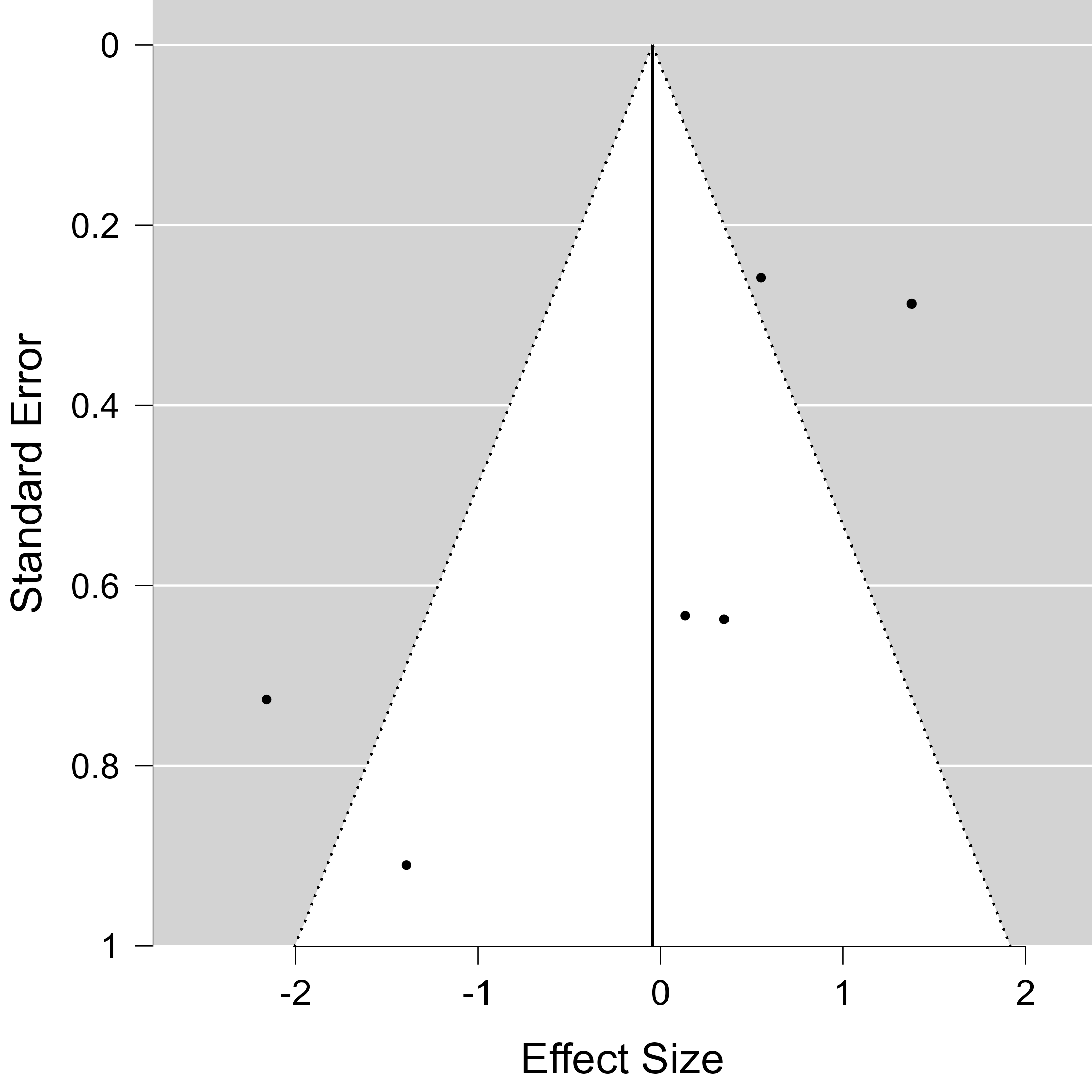

Supplement: Supplementary file 10 [file Image_10.tiff]

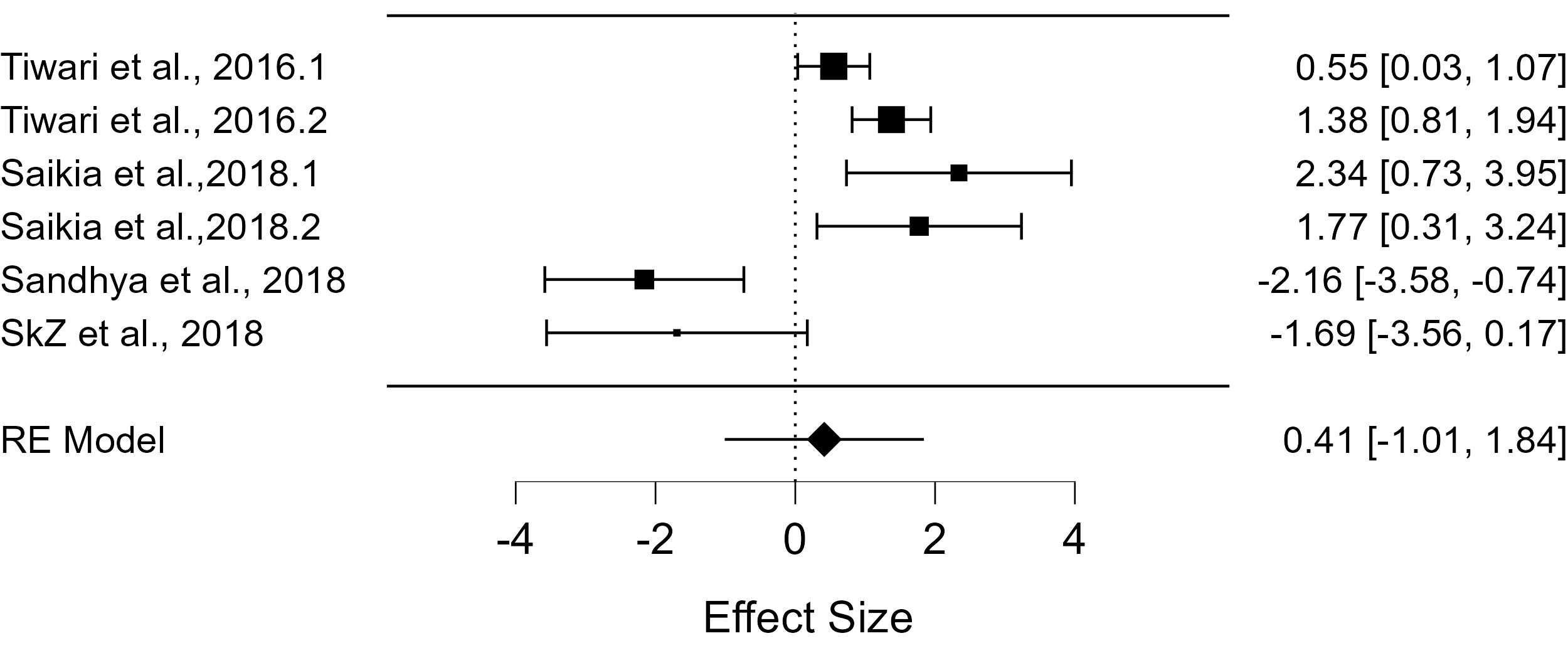

Supplement: Supplementary file 11 [file Image_11.tiff]

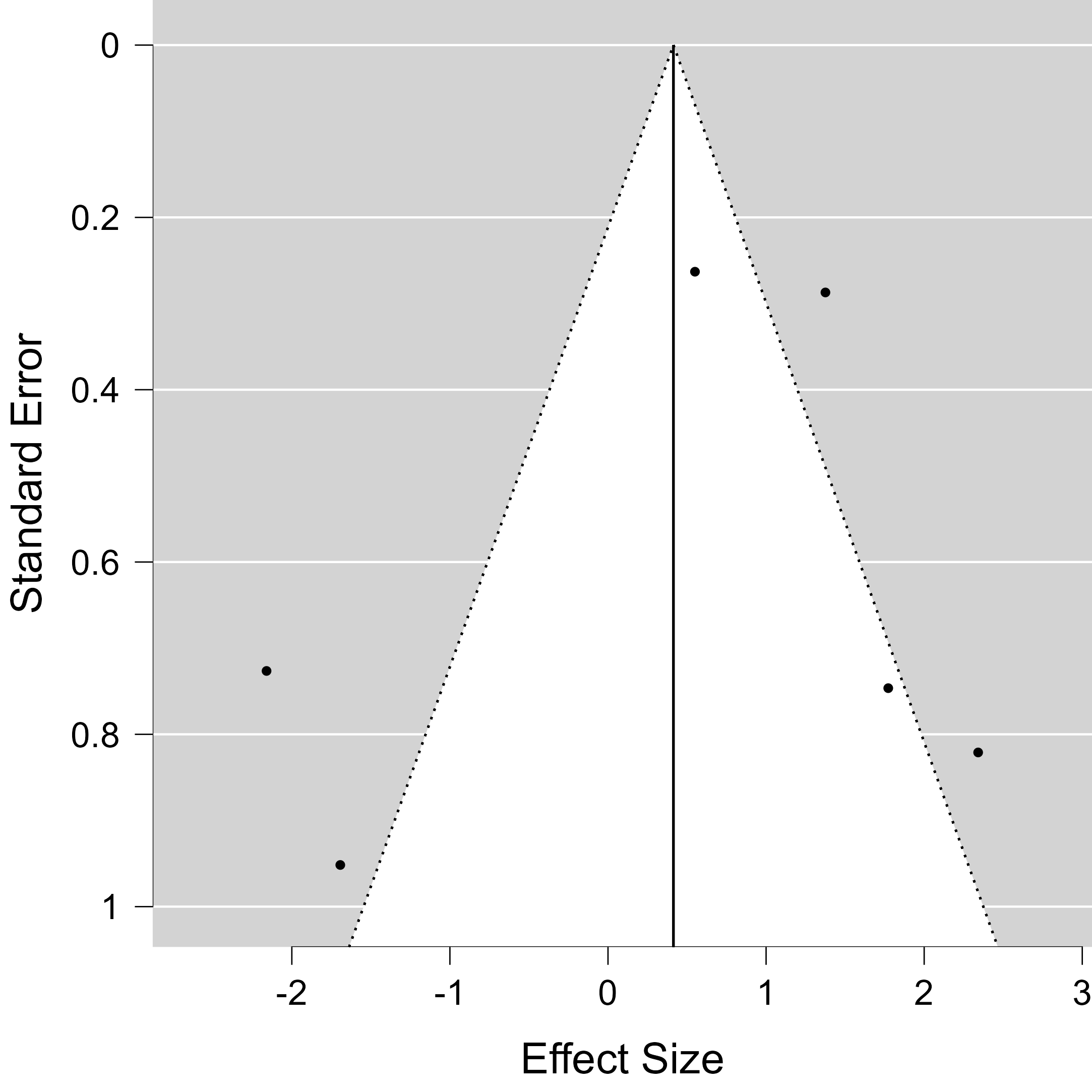

Supplement: Supplementary file 12 [file Image_12.tiff]

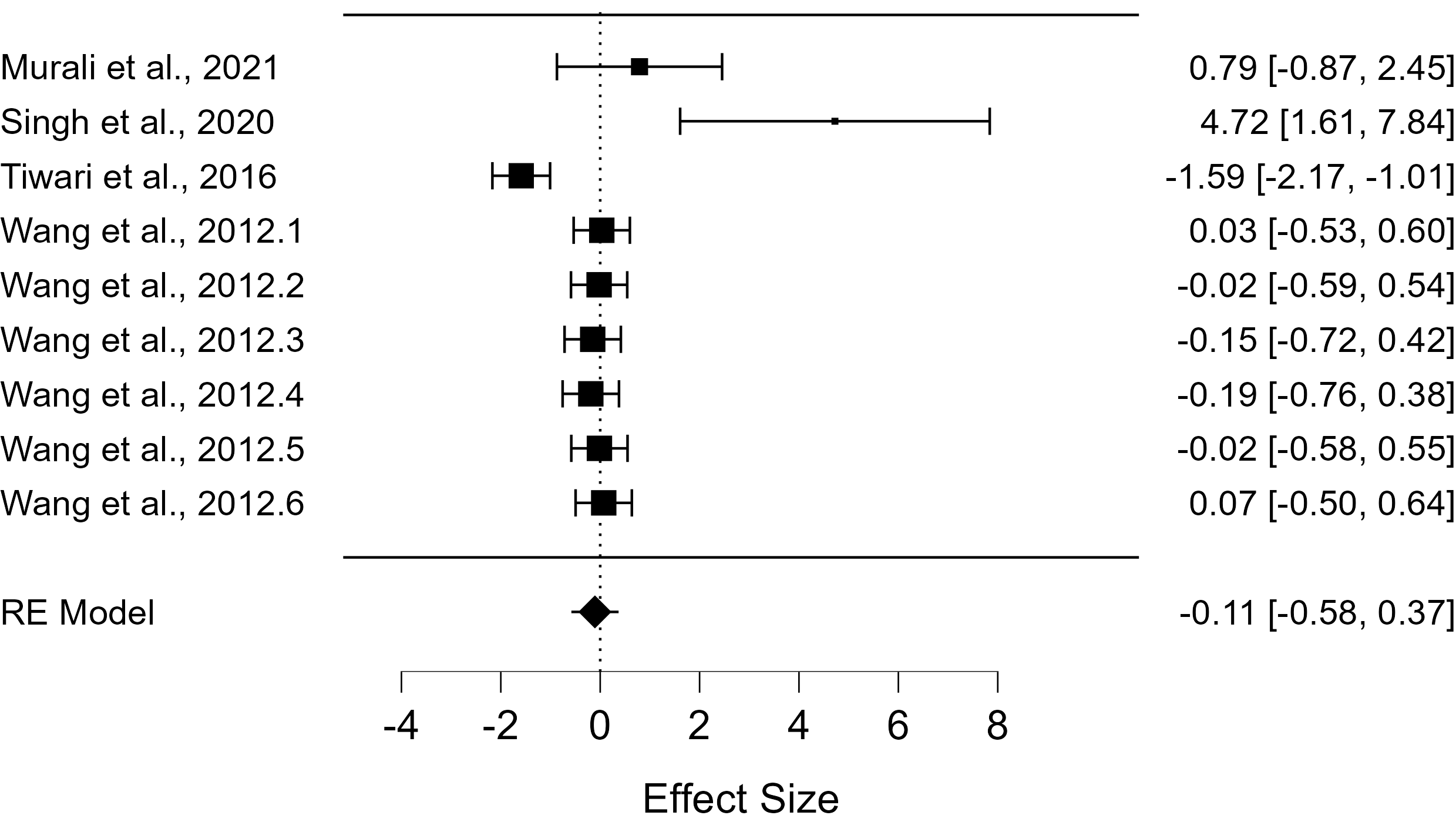

Supplement: Supplementary file 13 [file Image_13.tiff]

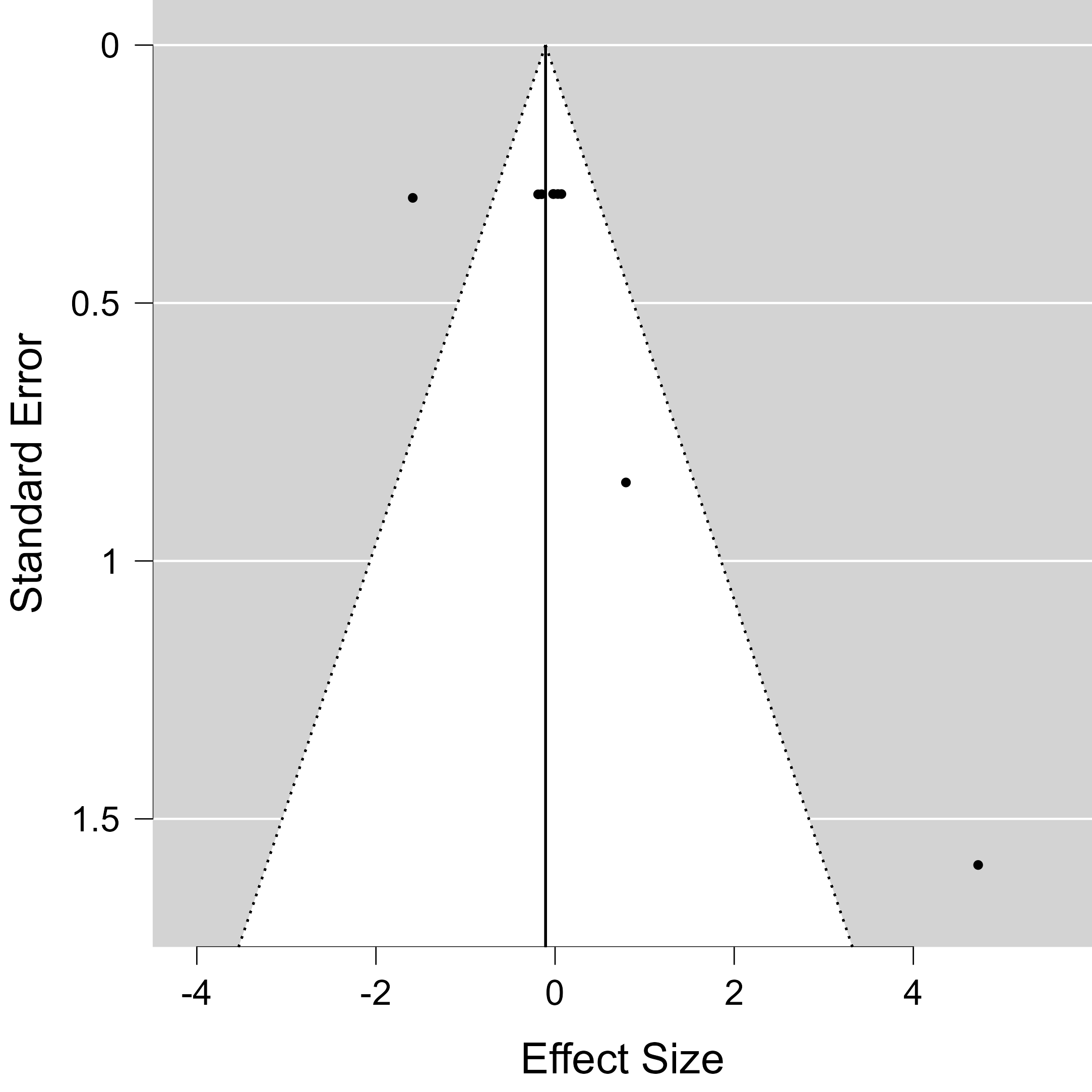

Supplement: Supplementary file 14 [file Image_14.tiff]

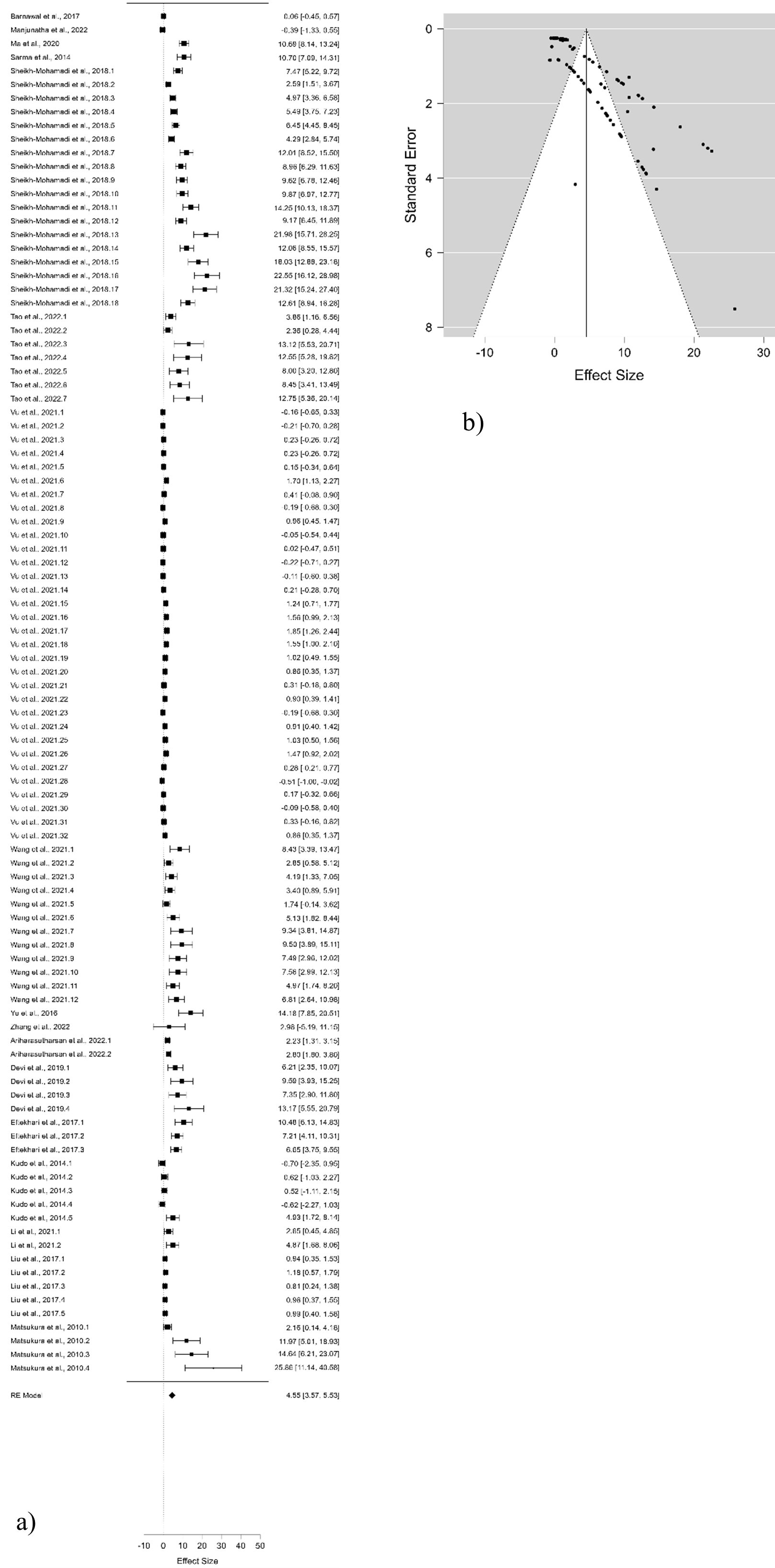

Supplement: Supplementary file 15 [file Image_15.tif]
