# Supplementary material for: Meta-analysis of plant growth-promoting rhizobacteria interaction with host plants: implications for drought stress response gene expression
Source: Front Plant Sci. 2024 Jan 15;14:1282553. doi: 10.3389/fpls.2023.1282553 (PMC10823023; doi:10.3389/fpls.2023.1282553)
Supplement: Supplementary file 28 [file DataSheet_1.docx]

List of articles found during the systematic review for:

***1-aminocyclopropane-1-carboxylic acid deaminase (acdS)***

1. Abbasi, S.; Sadeghi, A.; Safaie, N. Streptomyces Alleviate Drought Stress in Tomato Plants and Modulate the Expression of Transcription Factors ERF1 and WRKY70 Genes. *Scientia Horticulturae* **2020**, *265*, 109206, doi:10.1016/j.scienta.2020.109206.
2. Abd El-Daim, I.A.; Bejai, S.; Fridborg, I.; Meijer, J. Identifying Potential Molecular Factors Involved in *Bacillus Amyloliquefaciens* 5113 Mediated Abiotic Stress Tolerance in Wheat. *Plant Biol J* **2018**, *20*, 271–279, doi:10.1111/plb.12680.
3. Abd El-Daim, I.A.; Bejai, S.; Meijer, J. Bacillus Velezensis 5113 Induced Metabolic and Molecular Reprogramming during Abiotic Stress Tolerance in Wheat. *Sci Rep* **2019**, *9*, 16282, doi:10.1038/s41598-019-52567-x.
4. Abdelaal, K.; AlKahtani, M.; Attia, K.; Hafez, Y.; Király, L.; Künstler, A. The Role of Plant Growth-Promoting Bacteria in Alleviating the Adverse Effects of Drought on Plants. *Biology* **2021**, *10*, 520, doi:10.3390/biology10060520.
5. Afridi, M.S.; Amna; Sumaira; Mahmood, T.; Salam, A.; Mukhtar, T.; Mehmood, S.; Ali, J.; Khatoon, Z.; Bibi, M.; et al. Induction of Tolerance to Salinity in Wheat Genotypes by Plant Growth Promoting Endophytes: Involvement of ACC Deaminase and Antioxidant Enzymes. *Plant Physiology and Biochemistry* **2019**, *139*, 569–577, doi:10.1016/j.plaphy.2019.03.041.
6. Afridi, M.S.; Van Hamme, J. d.; Bundschuh, J.; Sumaira; Khan, M.N.; Salam, A.; Waqar, M.; Munis, M.F.H.; Chaudhary, H.J. Biotechnological Approaches in Agriculture and Environmental Management - Bacterium Kocuria Rhizophila 14ASP as Heavy Metal and Salt- Tolerant Plant Growth- Promoting Strain. *Biologia* **2021**, *76*, 3091–3105, doi:10.1007/s11756-021-00826-6.
7. Afzal, I.; Shinwari, Z.K.; Sikandar, S.; Shahzad, S. Plant Beneficial Endophytic Bacteria: Mechanisms, Diversity, Host Range and Genetic Determinants. *Microbiological Research* **2019**, *221*, 36–49, doi:10.1016/j.micres.2019.02.001.
8. Afzal, M.; Yousaf, S.; Reichenauer, T.G.; Kuffner, M.; Sessitsch, A. Soil Type Affects Plant Colonization, Activity and Catabolic Gene Expression of Inoculated Bacterial Strains during Phytoremediation of Diesel. *Journal of Hazardous Materials* **2011**, *186*, 1568–1575, doi:10.1016/j.jhazmat.2010.12.040.
9. Ahkami, A.H.; Allen White, R.; Handakumbura, P.P.; Jansson, C. Rhizosphere Engineering: Enhancing Sustainable Plant Ecosystem Productivity. *Rhizosphere* **2017**, *3*, 233–243, doi:10.1016/j.rhisph.2017.04.012.
10. Ahluwalia, O.; Singh, P.C.; Bhatia, R. A Review on Drought Stress in Plants: Implications, Mitigation and the Role of Plant Growth Promoting Rhizobacteria. *Resources, Environment and Sustainability* **2021**, *5*, 100032, doi:10.1016/j.resenv.2021.100032.
11. Akhgar, A.R.; Arzanlou, M.; Bakker, P.A.H.M.; Hamidpour, M. Characterization of 1-Aminocyclopropane-1-Carboxylate (ACC) Deaminase-Containing Pseudomonas Spp. in the Rhizosphere of Salt-Stressed Canola. *Pedosphere* **2014**, *24*, 461–468, doi:10.1016/S1002-0160(14)60032-1.
12. Alaylar, B.; Karadayi, M. Investigation of ACC-Deaminase and Indole Acetic Acid Producing Bacteria from Rhizospheric Soils in Ağrı Province. *Journal of the Institute of Science and Technology* **2021**, 933–942, doi:10.21597/jist.854685.
13. Albdaiwi, R.N.; Khyami-Horani, H.; Ayad, J.Y.; Alananbeh, K.M.; Al-Sayaydeh, R. Isolation and Characterization of Halotolerant Plant Growth Promoting Rhizobacteria From Durum Wheat (*Triticum turgidum* Subsp. *durum*) Cultivated in Saline Areas of the Dead Sea Region. *Front. Microbiol.* **2019**, *10*, 1639, doi:10.3389/fmicb.2019.01639.
14. Alcázar, R.; Marco, F.; Cuevas, J.C.; Patron, M.; Ferrando, A.; Carrasco, P.; Tiburcio, A.F.; Altabella, T. Involvement of Polyamines in Plant Response to Abiotic Stress. *Biotechnol Lett* **2006**, *28*, 1867–1876, doi:10.1007/s10529-006-9179-3.
15. Alemneh, A.A.; Zhou, Y.; Ryder, M.H.; Denton, M.D. Mechanisms in Plant Growth‐promoting Rhizobacteria That Enhance Legume–Rhizobial Symbioses. *J. Appl. Microbiol.* **2020**, *129*, 1133–1156, doi:10.1111/jam.14754.
16. Alexander, A.; Singh, V.K.; Mishra, A. Interaction of the Novel Bacterium Brachybacterium Saurashtrense JG06 with Arachis Hypogaea Leads to Changes in Physio-Biochemical Activity of Plants to Cope with Nitrogen Starvation Conditions. *Plant Physiology and Biochemistry* **2021**, *166*, 974–984, doi:10.1016/j.plaphy.2021.07.007.
17. Ali, B.; Wang, X.; Saleem, M.H.; Azeem, M.A.; Afridi, M.S.; Nadeem, M.; Ghazal, M.; Batool, T.; Qayyum, A.; Alatawi, A.; et al. Bacillus Mycoides PM35 Reinforces Photosynthetic Efficiency, Antioxidant Defense, Expression of Stress-Responsive Genes, and Ameliorates the Effects of Salinity Stress in Maize. *Life* **2022**, *12*, 219, doi:10.3390/life12020219.
18. Ali, B.; Wang, X.; Saleem, M.H.; Sumaira; Hafeez, A.; Afridi, M.S.; Khan, S.; Zaib-Un-Nisa; Ullah, I.; Amaral Júnior, A.T. do; et al. PGPR-Mediated Salt Tolerance in Maize by Modulating Plant Physiology, Antioxidant Defense, Compatible Solutes Accumulation and Bio-Surfactant Producing Genes. *Plants* **2022**, *11*, 345, doi:10.3390/plants11030345.
19. Ali, S.; Charles, T.C.; Glick, B.R. Amelioration of High Salinity Stress Damage by Plant Growth-Promoting Bacterial Endophytes That Contain ACC Deaminase. *Plant Physiology and Biochemistry* **2014**, *80*, 160–167, doi:10.1016/j.plaphy.2014.04.003.
20. Ali, S.; Charles, T.C.; Glick, B.R. Delay of Flower Senescence by Bacterial Endophytes Expressing 1-Aminocyclopropane-1-Carboxylate Deaminase. *J Appl Microbiol* **2012**, *113*, 1139–1144, doi:10.1111/j.1365-2672.2012.05409.x.
21. Ali, S.; Khan, M.A.; Kim, W.-C. Pseudomonas Veronii KJ Mitigates Flood Stress-Associated Damage in Sesamum Indicum L. *Appl Biol Chem* **2018**, *61*, 575–585, doi:10.1007/s13765-018-0392-2.
22. Ali, S.; Khan, N. Delineation of Mechanistic Approaches Employed by Plant Growth Promoting Microorganisms for Improving Drought Stress Tolerance in Plants. *Microbiological Research* **2021**, *249*, 126771, doi:10.1016/j.micres.2021.126771.
23. Ali, S.; Kim, W.-C. Plant Growth Promotion Under Water: Decrease of Waterlogging-Induced ACC and Ethylene Levels by ACC Deaminase-Producing Bacteria. *Front. Microbiol.* **2018**, *9*, 1096, doi:10.3389/fmicb.2018.01096.
24. Ali, S.; Xie, L. Plant Growth Promoting and Stress Mitigating Abilities of Soil Born Microorganisms. *FNA* **2020**, *11*, 96–104, doi:10.2174/2212798410666190515115548.
25. Al-Khayri, J.M., Jain, S.M., Johnson, D.V.*.The Date Palm Genome, Vol. 2: Omics and Molecular Breeding*; Eds.; Compendium of Plant Genomes; Springer International Publishing: Cham, 2021; ISBN 978-3-030-73749-8.
26. Amna; Ud Din, B.; Sarfraz, S.; Xia, Y.; Kamran, M.A.; Javed, M.T.; Sultan, T.; Hussain Munis, M.F.; Chaudhary, H.J. Mechanistic Elucidation of Germination Potential and Growth of Wheat Inoculated with Exopolysaccharide and ACC- Deaminase Producing Bacillus Strains under Induced Salinity Stress. *Ecotoxicology and Environmental Safety* **2019**, *183*, 109466, doi:10.1016/j.ecoenv.2019.109466.
27. Amna; Xia, Y.; Farooq, M.A.; Javed, M.T.; Kamran, M.A.; Mukhtar, T.; Ali, J.; Tabassum, T.; Rehman, S. ur; Hussain Munis, M.F.; et al. Multi-Stress Tolerant PGPR Bacillus Xiamenensis PM14 Activating Sugarcane (Saccharum Officinarum L.) Red Rot Disease Resistance. *Plant Physiology and Biochemistry* **2020**, *151*, 640–649, doi:10.1016/j.plaphy.2020.04.016.
28. Anand, G.; Bhattacharjee, A.; Shrivas, V.L.; Dubey, S.; Sharma, S. ACC Deaminase Positive Enterobacter-Mediated Mitigation of Salinity Stress, and Plant Growth Promotion of Cajanus Cajan: A Lab to Field Study. *Physiol Mol Biol Plants* **2021**, *27*, 1547–1557, doi:10.1007/s12298-021-01031-0.
29. Ansari, F.A.; Ahmad, I.; Pichtel, J. Growth Stimulation and Alleviation of Salinity Stress to Wheat by the Biofilm Forming Bacillus Pumilus Strain FAB10. *Applied Soil Ecology* **2019**, *143*, 45–54, doi:10.1016/j.apsoil.2019.05.023.
30. Ansari, F.A.; Jabeen, M.; Ahmad, I. *Pseudomonas azotoformans* FAP5, a Novel Biofilm-Forming PGPR Strain, Alleviates Drought Stress in Wheat Plant. *Int. J. Environ. Sci. Technol.* **2021**, *18*, 3855–3870, doi:10.1007/s13762-020-03045-9.
31. Ansari, M.W.; Trivedi, D.K.; Sahoo, R.K.; Gill, S.S.; Tuteja, N. A Critical Review on Fungi Mediated Plant Responses with Special Emphasis to Piriformospora Indica on Improved Production and Protection of Crops. *Plant Physiology and Biochemistry* **2013**, *70*, 403–410, doi:10.1016/j.plaphy.2013.06.005.
32. Argueso, C.T.; Hansen, M.; Kieber, J.J. Regulation of Ethylene Biosynthesis. *J Plant Growth Regul* **2007**, *26*, 92–105, doi:10.1007/s00344-007-0013-5.
33. Arif, I.; Batool, M.; Schenk, P.M. Plant Microbiome Engineering: Expected Benefits for Improved Crop Growth and Resilience. *Trends in Biotechnology* **2020**, *38*, 1385–1396, doi:10.1016/j.tibtech.2020.04.015.
34. Arraes, F.B.M.; Beneventi, M.A.; Lisei de Sa, M.E.; Paixao, J.F.R.; Albuquerque, E.V.S.; Marin, S.R.R.; Purgatto, E.; Nepomuceno, A.L.; Grossi-de-Sa, M.F. Implications of Ethylene Biosynthesis and Signaling in Soybean Drought Stress Tolerance. *BMC Plant Biol* **2015**, *15*, 213, doi:10.1186/s12870-015-0597-z.
35. Arun K., D.; Sabarinathan, K.G.; Gomathy, M.; Kannan, R.; Balachandar, D. Mitigation of Drought Stress in Rice Crop with Plant Growth-Promoting Abiotic Stress-Tolerant Rice Phyllosphere Bacteria. *J Basic Microbiol* **2020**, 60, 768-786, doi:10.1002/jobm.202000011.
36. Asaf, S.; Khan, A.L.; Khan, M.A.; Imran, Q.M.; Yun, B.-W.; Lee, I.-J. Osmoprotective Functions Conferred to Soybean Plants via Inoculation with *Sphingomonas* Sp. LK11 and Exogenous Trehalose. *Microbiological Research* **2017**, *205*, 135–145, doi:10.1016/j.micres.2017.08.009.
37. Ashwitha, K.; Rangeshwaran, R.; Vajid, N.V.; Sivakumar, G.; Jalali, S.K.; Rajalaxmi, K.; Manjunath, H. Characterization of Abiotic Stress Tolerant Pseudomonas Spp. Occurring in Indian Soils. *J Bio Control* **2013**,*27*, 25-48.
38. Asif, M.; Pervez, A.; Ahmad, R. Role of Melatonin and Plant‐Growth‐Promoting Rhizobacteria in the Growth and Development of Plants. *Clean – Soil, Air, Water* **2019**, *47*, 1800459, doi:10.1002/clen.201800459.
39. Aslam, F.; Ali, B. Halotolerant Bacterial Diversity Associated with Suaeda Fruticosa (L.) Forssk. Improved Growth of Maize under Salinity Stress. *Agronomy* **2018**, *8*, 131, doi:10.3390/agronomy8080131.
40. Azcón, R.; Medina, A.; Aroca, R.; Ruiz-Lozano, J.M. Abiotic Stress Remediation by the Arbuscular Mycorrhizal Symbiosis and Rhizosphere Bacteria/Yeast Interactions. In *Molecular Microbial Ecology of the Rhizosphere*; de Bruijn, F.J., Ed.; John Wiley & Sons, Inc.: Hoboken, NJ, USA, 2013; pp. 991–1002 ISBN 978-1-118-29767-4.
41. Backer, R.; Rokem, J.S.; Ilangumaran, G.; Lamont, J.; Praslickova, D.; Ricci, E.; Subramanian, S.; Smith, D.L. Plant Growth-Promoting Rhizobacteria: Context, Mechanisms of Action, and Roadmap to Commercialization of Biostimulants for Sustainable Agriculture. *Front. Plant Sci.* **2018**, *9*, 1473, doi:10.3389/fpls.2018.01473.
42. Bajpai, A.; Mahawar, H.; Dubey, G.; Atoliya, N.; Parmar, R.; Devi, M.H.; Kollah, B.; Mohanty, S.R. Prospect of Pink Pigmented Facultative Methylotrophs in Mitigating Abiotic Stress and Climate Change. *J Basic Microbiol* **2022**, jobm.202200087, doi:10.1002/jobm.202200087.
43. Bakshi, M.; Oelmüller, R. WRKY Transcription Factors: Jack of Many Trades in Plants. *Plant Signaling & Behavior* **2014**, *9*, e27700, doi:10.4161/psb.27700.
44. Bal, H.B.; Das, S.; Dangar, T.K.; Adhya, T.K. ACC Deaminase and IAA Producing Growth Promoting Bacteria from the Rhizosphere Soil of Tropical Rice Plants: Growth Promoting Rhizobacteria from Tropical Rice. *J. Basic Microbiol.* **2013**, *53*, 972–984, doi:10.1002/jobm.201200445.
45. Bal, H.B.; Nayak, L.; Das, S.; Adhya, T.K. Isolation of ACC Deaminase Producing PGPR from Rice Rhizosphere and Evaluating Their Plant Growth Promoting Activity under Salt Stress. *Plant Soil* **2013**, *366*, 93–105, doi:10.1007/s11104-012-1402-5.
46. Bangash, N.; Mahmood, S.; Akhtar, S.; Hayat, M.T.; Gulzar, S.; Khalid, A. Formulation of Biofertilizer for Improving Growth and Yield of Wheat in Rain Dependent Farming System. *Environmental Technology & Innovation* **2021**, *24*, 101806, doi:10.1016/j.eti.2021.101806.
47. Bardi, L.; Malusà, E. DROUGHT AND NUTRITIONAL STRESSES IN PLANT: ALLEVIATING ROLE OF RHIZOSPHERIC MICROORGANISMS. In *Abiotic Stress*: *New Research*; Nova Science Publisher, Inc.: Hauppauge, NY, USA, 2012; pp 1-57.
48. Barnawal, D.; Bharti, N.; Maji, D.; Chanotiya, C.S.; Kalra, A. ACC Deaminase-Containing Arthrobacter Protophormiae Induces NaCl Stress Tolerance through Reduced ACC Oxidase Activity and Ethylene Production Resulting in Improved Nodulation and Mycorrhization in Pisum Sativum. *Journal of Plant Physiology* **2014**, *171*, 884–894, doi:10.1016/j.jplph.2014.03.007.
49. Barnawal, D.; Bharti, N.; Pandey, S.S.; Pandey, A.; Chanotiya, C.S.; Kalra, A. Plant Growth-Promoting Rhizobacteria Enhance Wheat Salt and Drought Stress Tolerance by Altering Endogenous Phytohormone Levels and *TaCTR1* / *TaDREB2* Expression. *Physiol Plantarum* **2017**, *161*, 502–514, doi:10.1111/ppl.12614.
50. Barnawal, D.; Bharti, N.; Tripathi, A.; Pandey, S.S.; Chanotiya, C.S.; Kalra, A. ACC-Deaminase-Producing Endophyte Brachybacterium Paraconglomeratum Strain SMR20 Ameliorates Chlorophytum Salinity Stress via Altering Phytohormone Generation. *J Plant Growth Regul* **2016**, *35*, 553–564, doi:10.1007/s00344-015-9560-3.
51. Barnawal, D.; Maji, D.; Bharti, N.; Chanotiya, C.S.; Kalra, A. ACC Deaminase-Containing Bacillus Subtilis Reduces Stress Ethylene-Induced Damage and Improves Mycorrhizal Colonization and Rhizobial Nodulation in Trigonella Foenum-Graecum Under Drought Stress. *J Plant Growth Regul* **2013**, *32*, 809–822, doi:10.1007/s00344-013-9347-3.
52. Barnawal, D.; Pandey, S.S.; Bharti, N.; Pandey, A.; Ray, T.; Singh, S.; Chanotiya, C.S.; Kalra, A. ACC Deaminase-Containing Plant Growth-Promoting Rhizobacteria Protect *Papaver Somniferum* from Downy Mildew. *J Appl Microbiol* **2017**, *122*, 1286–1298, doi:10.1111/jam.13417.
53. Basu, S.; Rabara, R.; Negi, S. Towards a Better Greener Future - an Alternative Strategy Using Biofertilizers. I: Plant Growth Promoting Bacteria. *Plant Gene* **2017**, *12*, 43–49, doi:10.1016/j.plgene.2017.07.004.
54. Belimov, A.A.; Dodd, I.C.; Hontzeas, N.; Theobald, J.C.; Safronova, V.I.; Davies, W.J. Rhizosphere Bacteria Containing 1‐aminocyclopropane‐1‐carboxylate Deaminase Increase Yield of Plants Grown in Drying Soil via Both Local and Systemic Hormone Signalling. *New Phytologist* **2009**, *181*, 413–423, doi:10.1111/j.1469-8137.2008.02657.x.
55. Belimov, A.A.; Dodd, I.C.; Safronova, V.I.; Shaposhnikov, A.I.; Azarova, T.S.; Makarova, N.M.; Davies, W.J.; Tikhonovich, I.A. Rhizobacteria That Produce Auxins and Contain 1-Amino-Cyclopropane-1-Carboxylic Acid Deaminase Decrease Amino Acid Concentrations in the Rhizosphere and Improve Growth and Yield of Well-Watered and Water-Limited Potato ( *Solanum Tuberosum* ): Rhizobacterial Effects on Rhizosphere Amino Acid Concentrations and Potato Growth. *Ann Appl Biol* **2015**, *167*, 11–25, doi:10.1111/aab.12203.
56. Belimov, A.A.; Zinovkina, N.Y.; Safronova, V.I.; Litvinsky, V.A.; Nosikov, V.V.; Zavalin, A.A.; Tikhonovich, I.A. Rhizobial ACC Deaminase Contributes to Efficient Symbiosis with Pea (Pisum Sativum L.) under Single and Combined Cadmium and Water Deficit Stress. *Environmental and Experimental Botany* **2019**, *167*, 103859, doi:10.1016/j.envexpbot.2019.103859.
57. Betti, C., Della Rovere, F., Piacentini, D., Fattorini, L., Falasca, G., & Altamura, M. M. Jasmonates, ethylene and brassinosteroids control adventitious and lateral rooting as stress avoidance responses to heavy metals and metalloids. *Biomolecules* **2021**, *11*(1), 77.
58. Bhardwaj, D.; Ansari, M.W.; Sahoo, R.K.; Tuteja, N. Biofertilizers Function as Key Player in Sustainable Agriculture by Improving Soil Fertility, Plant Tolerance and Crop Productivity. *Microb Cell Fact* **2014**, *13*, 66, doi:10.1186/1475-2859-13-66.
59. Bharti, N.; Barnawal, D. Amelioration of Salinity Stress by PGPR. In *PGPR Amelioration in Sustainable Agriculture*; Elsevier, 2019; pp. 85–106 ISBN 978-0-12-815879-1.
60. Bhattacharyya, D.; Yu, S.-M.; Lee, Y.H. Volatile Compounds from *Alcaligenes Faecalis* JBCS1294 Confer Salt Tolerance in *Arabidopsis Thaliana* through the Auxin and Gibberellin Pathways and Differential Modulation of Gene Expression in Root and Shoot Tissues. *Plant Growth Regul* **2015**, *75*, 297–306, doi:10.1007/s10725-014-9953-5.
61. Bhattacharyya, P.N.; Jha, D.K. Plant Growth-Promoting Rhizobacteria (PGPR): Emergence in Agriculture. *World J Microbiol Biotechnol* **2012**, *28*, 1327–1350, doi:10.1007/s11274-011-0979-9.
62. Bhise, K.K.; Bhagwat, P.K.; Dandge, P.B. Plant Growth-Promoting Characteristics of Salt Tolerant Enterobacter Cloacae Strain KBPD and Its Efficacy in Amelioration of Salt Stress in Vigna Radiata L. *J Plant Growth Regul* **2017**, *36*, 215–226, doi:10.1007/s00344-016-9631-0.
63. Bhise, K.K.; Bhagwat, P.K.; Dandge, P.B. Synergistic Effect of Chryseobacterium Gleum Sp. SUK with ACC Deaminase Activity in Alleviation of Salt Stress and Plant Growth Promotion in Triticum Aestivum L. *3 Biotech* **2017**, *7*, 105, doi:10.1007/s13205-017-0739-0.
64. Bhise, K.K.; Dandge, P.B. Mitigation of Salinity Stress in Plants Using Plant Growth Promoting Bacteria. *Symbiosis* **2019**, *79*, 191–204, doi:10.1007/s13199-019-00638-y.
65. Bilal, S.; Shahzad, R.; Imran, M.; Jan, R.; Kim, K.M.; Lee, I.-J. Synergistic Association of Endophytic Fungi Enhances *Glycine Max* L. resilience to Combined Abiotic Stresses: Heavy Metals, High Temperature and Drought Stress. *Industrial Crops and Products* **2020**, *143*, 111931, doi:10.1016/j.indcrop.2019.111931.
66. Biodiversity and Biotechnological Applications of Halophilic Microbes for Sustainable Agriculture. *J App Biol Biotech* **2018**, doi:10.7324/JABB.2018.60109.
67. *Biofertilizers for Sustainable Agriculture and Environment*; Giri, B., Prasad, R., Wu, Q.-S., Varma, A., Eds.; Soil Biology; Springer International Publishing: Cham, 2019; Vol. 55; ISBN 978-3-030-18932-7.
68. Bomle, D.V.; Kiran, A.; Kumar, J.K.; Nagaraj, L.S.; Pradeep, C.K.; Ansari, M.A.; Alghamdi, S.; Kabrah, A.; Assaggaf, H.; Dablool, A.S.; et al. Plants Saline Environment in Perception with Rhizosphere Bacteria Containing 1-Aminocyclopropane-1-Carboxylate Deaminase. *IJMS* **2021**, *22*, 11461, doi:10.3390/ijms222111461.
69. Braga, R.M.; Dourado, M.N.; Araújo, W.L. Microbial Interactions: Ecology in a Molecular Perspective. *Brazilian Journal of Microbiology* **2016**, *47*, 86–98, doi:10.1016/j.bjm.2016.10.005.
70. Brígido, C.; Nascimento, F.X.; Duan, J.; Glick, B.R.; Oliveira, S. Expression of an Exogenous 1-Aminocyclopropane-1-Carboxylate Deaminase Gene in *Mesorhizobium* Spp. Reduces the Negative Effects of Salt Stress in Chickpea. *FEMS Microbiol Lett* **2013**, *343*, 46-53, doi:10.1111/1574-6968.12294.
71. Brijesh Singh, S.; Gowtham, H.G.; Murali, M.; Hariprasad, P.; Lakshmeesha, T.R.; Narasimha Murthy, K.; Amruthesh, K.N.; Niranjana, S.R. Plant Growth Promoting Ability of ACC Deaminase Producing Rhizobacteria Native to Sunflower (Helianthus Annuus L.). *Biocatalysis and Agricultural Biotechnology* **2019**, *18*, 101089, doi:10.1016/j.bcab.2019.101089.
72. Brotman, Y.; Landau, U.; Cuadros-Inostroza, Á.; Takayuki, T.; Fernie, A.R.; Chet, I.; Viterbo, A.; Willmitzer, L. Trichoderma-Plant Root Colonization: Escaping Early Plant Defense Responses and Activation of the Antioxidant Machinery for Saline Stress Tolerance. *PLoS Pathog* **2013**, *9*, e1003221, doi:10.1371/journal.ppat.1003221.
73. Brusamarello-Santos, L.C.C.; Pacheco, F.; Aljanabi, S.M.M.; Monteiro, R.A.; Cruz, L.M.; Baura, V.A.; Pedrosa, F.O.; Souza, E.M.; Wassem, R. Differential Gene Expression of Rice Roots Inoculated with the Diazotroph *Herbaspirillum Seropedicae*. *Plant Soil* **2012**, *356*, 113–125, doi:10.1007/s11104-011-1044-z.
74. Bukhat, S.; Imran, A.; Javaid, S.; Shahid, M.; Majeed, A.; Naqqash, T. Communication of Plants with Microbial World: Exploring the Regulatory Networks for PGPR Mediated Defense Signaling. *Microbiological Research* **2020**, *238*, 126486, doi:10.1016/j.micres.2020.126486.
75. Camaille, M.; Fabre, N.; Clément, C.; Ait Barka, E. Advances in Wheat Physiology in Response to Drought and the Role of Plant Growth Promoting Rhizobacteria to Trigger Drought Tolerance. *Microorganisms* **2021**, *9*, 687, doi:10.3390/microorganisms9040687.
76. Carlson, R.; Tugizimana, F.; Steenkamp, P.A.; Dubery, I.A.; Hassen, A.I.; Labuschagne, N. Rhizobacteria-Induced Systemic Tolerance against Drought Stress in *Sorghum bicolor* (L.) Moench. *Microbiological Research* **2020**, *232*, 126388, doi:10.1016/j.micres.2019.126388.
77. Chakraborti, S.; Bera, K.; Sadhukhan, S.; Dutta, P. Bio-Priming of Seeds: Plant Stress Management and Its Underlying Cellular, Biochemical and Molecular Mechanisms. *Plant Stress* **2022**, *3*, 100052, doi:10.1016/j.stress.2021.100052.
78. Chakraborty, U.; Chakraborty, B.; Dey, P.; Chakraborty, A.P. Role of Microorganisms in Alleviation of Abiotic Stresses for Sustainable Agriculture. In *Abiotic stresses in crop plants*; Eds.; CABI: Wallingford, 2015; pp. 232–253 ISBN 978-1-78064-373-1.
79. Chandna, R.; Augustine, R.; Bisht, N.C. Evaluation of Candidate Reference Genes for Gene Expression Normalization in Brassica Juncea Using Real Time Quantitative RT-PCR. *PLoS ONE* **2012**, *7*, e36918, doi:10.1371/journal.pone.0036918.
80. Chandra, D.; Srivastava, R.; Glick, B.R.; Sharma, A.K. Drought-Tolerant Pseudomonas Spp. Improve the Growth Performance of Finger Millet (Eleusine Coracana (L.) Gaertn.) Under Non-Stressed and Drought-Stressed Conditions. *Pedosphere* **2018**, *28*, 227–240, doi:10.1016/S1002-0160(18)60013-X.
81. Chandra, D.; Srivastava, R.; Gupta, V.V.S.R.; Franco, C.M.M.; Paasricha, N.; Saifi, S.K.; Tuteja, N.; Sharma, A.K. Field Performance of Bacterial Inoculants to Alleviate Water Stress Effects in Wheat (*Triticum Aestivum* L.). *Plant Soil* **2019**, *441*, 261–281, doi:10.1007/s11104-019-04115-9.
82. Chandrakala, C.; Voleti, S.R.; Bandeppa, S.; Sunil Kumar, N.; Latha, P.C. Silicate Solubilization and Plant Growth Promoting Potential of Rhizobium Sp. Isolated from Rice Rhizosphere. *Silicon* **2019**, *11*, 2895–2906, doi:10.1007/s12633-019-0079-2.
83. Chatterjee, A.; Shankar, A.; Singh, S.; Kesari, V.; Rai, R.; Patel, A.K.; Rai, L.C. Beneficial Microorganisms and Abiotic Stress Tolerance in Plants. In *Approaches for Enhancing Abiotic Stress Tolerance in Plants*; Hasanuzzaman, M., Nahar, K., Fujita, M., Oku, H., Islam, T., Eds.; CRC Press: Boca Raton, FL : CRC Press, Taylor & Francis Group, 2019., 2019; pp. 473–502 ISBN 978-1-351-10472-2.
84. Chatterjee, P.; Kanagendran, A.; Samaddar, S.; Pazouki, L.; Sa, T.-M.; Niinemets, Ü. Influence of Brevibacterium Linens RS16 on Foliage Photosynthetic and Volatile Emission Characteristics upon Heat Stress in Eucalyptus Grandis. *Science of The Total Environment* **2020**, *700*, 134453, doi:10.1016/j.scitotenv.2019.134453.
85. Chatterjee, P.; Kanagendran, A.; Samaddar, S.; Pazouki, L.; Sa, T.-M.; Niinemets, Ü. Inoculation of Brevibacterium Linens RS16 in Oryza Sativa Genotypes Enhanced Salinity Resistance: Impacts on Photosynthetic Traits and Foliar Volatile Emissions. *Science of The Total Environment* **2018**, *645*, 721–732, doi:10.1016/j.scitotenv.2018.07.187.
86. Chauhan, P.S.; Lata, C.; Tiwari, S.; Chauhan, A.S.; Mishra, S.K.; Agrawal, L.; Chakrabarty, D.; Nautiyal, C.S. Transcriptional Alterations Reveal Bacillus Amyloliquefaciens-Rice Cooperation under Salt Stress. *Sci Rep* **2019**, *9*, 11912, doi:10.1038/s41598-019-48309-8.
87. Chen, L.; Liu, Y.; Wu, G.; Veronican Njeri, K.; Shen, Q.; Zhang, N.; Zhang, R. Induced Maize Salt Tolerance by Rhizosphere Inoculation of *Bacillus Amyloliquefaciens* SQR9. *Physiol Plantarum* **2016**, *158*, 34–44, doi:10.1111/ppl.12441.
88. Cheng, Z.; Woody, O.Z.; McConkey, B.J.; Glick, B.R. Combined Effects of the Plant Growth-Promoting Bacterium Pseudomonas Putida UW4 and Salinity Stress on the Brassica Napus Proteome. *Applied Soil Ecology* **2012**, *61*, 255–263, doi:10.1016/j.apsoil.2011.10.006.
89. Cherif-Silini, H.; Thissera, B.; Bouket, A.C.; Saadaoui, N.; Silini, A.; Eshelli, M.; Alenezi, F.N.; Vallat, A.; Luptakova, L.; Yahiaoui, B.; et al. Durum Wheat Stress Tolerance Induced by Endophyte Pantoea Agglomerans with Genes Contributing to Plant Functions and Secondary Metabolite Arsenal. *IJMS* **2019**, *20*, 3989, doi:10.3390/ijms20163989.
90. Chiappero, J.; Cappellari, L. del R.; Sosa Alderete, L.G.; Palermo, T.B.; Banchio, E. Plant Growth Promoting Rhizobacteria Improve the Antioxidant Status in Mentha Piperita Grown under Drought Stress Leading to an Enhancement of Plant Growth and Total Phenolic Content. *Industrial Crops and Products* **2019**, *139*, 111553, doi:10.1016/j.indcrop.2019.111553.
91. Cho, S.-M.; Kang, B.R.; Kim, Y.C. Transcriptome Analysis of Induced Systemic Drought Tolerance Elicited by *Pseudomonas Chlororaphis* O6 in *Arabidopsis Thaliana*. *The Plant Pathology Journal* **2013**, *29*, 209–220, doi:10.5423/PPJ.SI.07.2012.0103.
92. Choi, J.; Roy Choudhury, A.; Walitang, D.I.; Lee, Y.; Sa, T. ACC Deaminase‐producing *Brevibacterium Linens* RS16 Enhances Heat‐stress Tolerance of Rice ( *Oryza Sativa* L.). *Physiologia Plantarum* **2022**, *174*, e13584, doi:10.1111/ppl.13584.
93. Choudhary, A.K.; Sultana, R.; Vales, M.I.; Saxena, K.B.; Kumar, R.R.; Ratnakumar, P. Integrated Physiological and Molecular Approaches to Improvement of Abiotic Stress Tolerance in Two Pulse Crops of the Semi-Arid Tropics. *The Crop Journal* **2018**, *6*, 99–114, doi:10.1016/j.cj.2017.11.002.
94. Choudhary, D.K., Varma, A., Tuteja, N. *Plant-Microbe Interaction: An Approach to Sustainable Agriculture*; Eds.; Springer Singapore: Singapore, 2016; ISBN 978-981-10-2853-3.
95. Choudhary, D.K.; Kasotia, A.; Jain, S.; Vaishnav, A.; Kumari, S.; Sharma, K.P.; Varma, A. Bacterial-Mediated Tolerance and Resistance to Plants Under Abiotic and Biotic Stresses. *J Plant Growth Regul* **2016**, *35*, 276–300, doi:10.1007/s00344-015-9521-x.
96. Choudhary, D.K.; Sharma, K.P.; Gaur, R.K. Biotechnological Perspectives of Microbes in Agro-Ecosystems. *Biotechnol Lett* **2011**, *33*, 1905–1910, doi:10.1007/s10529-011-0662-0.
97. Chu, T.N.; Tran, B.T.H.; Van Bui, L.; Hoang, M.T.T. Plant Growth-Promoting *Rhizobacterium Pseudomonas* PS01 Induces Salt Tolerance in *Arabidopsis Thaliana*. *BMC Res Notes* **2019**, *12*, 11, doi:10.1186/s13104-019-4046-1.
98. Correa-García, S.; Pande, P.; Séguin, A.; St-Arnaud, M.; Yergeau, E. Rhizoremediation of Petroleum Hydrocarbons: A Model System for Plant Microbiome Manipulation. *Microb. Biotechnol.* **2018**, *11*, 819–832, doi:10.1111/1751-7915.13303.
99. Creus, C.M.; Pereyra, M.A.; Casanovas, E.M.; Sueldo, R.J.; Barassi, C.A. Plant Growth-Promoting Effects of Rhizobacteria on Abiotic Stressed Plants. Azospirillum-Grasses Model. *The Amercas of Plants Science and Biotechnology* **2010,** *4*, 49-59.
100. Curá, J.; Franz, D.; Filosofía, J.; Balestrasse, K.; Burgueño, L. Inoculation with Azospirillum Sp. and Herbaspirillum Sp. Bacteria Increases the Tolerance of Maize to Drought Stress. *Microorganisms* **2017**, *5*, 41, doi:10.3390/microorganisms5030041.
101. Czarny, J.C.; Grichko, V.P.; Glick, B.R. Genetic Modulation of Ethylene Biosynthesis and Signaling in Plants. *Biotechnology Advances* **2006**, *24*, 410–419, doi:10.1016/j.biotechadv.2006.01.003.
102. Dakora, F.D., Chimphango, S.B.M., Valentine, A.J., Elmerich, C., Newton, W.E. *Biological Nitrogen Fixation: Towards Poverty Alleviation through Sustainable Agriculture: Proceedings of the 15th International Nitrogen Fixation Congress and the 12th International Conference of the African Association for Biological Nitrogen Fixation*; Eds.; Current Plant Science and Biotechnology in Agriculture; Springer Netherlands: Dordrecht, 2008; Vol. 42; ISBN 978-1-4020-8251-1.
103. Danish, S.; Kiran, S.; Fahad, S.; Ahmad, N.; Ali, M.A.; Tahir, F.A.; Rasheed, M.K.; Shahzad, K.; Li, X.; Wang, D.; et al. Alleviation of Chromium Toxicity in Maize by Fe Fortification and Chromium Tolerant ACC Deaminase Producing Plant Growth Promoting Rhizobacteria. *Ecotoxicology and Environmental Safety* **2019**, *185*, 109706, doi:10.1016/j.ecoenv.2019.109706.
104. Danish, S.; Zafar-ul-Hye, M. Co-Application of ACC-Deaminase Producing PGPR and Timber-Waste Biochar Improves Pigments Formation, Growth and Yield of Wheat under Drought Stress. *Sci Rep* **2019**, *9*, 5999, doi:10.1038/s41598-019-42374-9.
105. Danish, S.; Zafar-ul-Hye, M. Combined Role of ACC Deaminase Producing Bacteria and Biochar on Cereals Productivity under Drought. *Phyton* **2020**, *89*, 217–227, doi:10.32604/phyton.2020.08523.
106. Danish, S.; Zafar-ul-Hye, M.; Fahad, S.; Saud, S.; Brtnicky, M.; Hammerschmiedt, T.; Datta, R. Drought Stress Alleviation by ACC Deaminase Producing Achromobacter Xylosoxidans and Enterobacter Cloacae, with and without Timber Waste Biochar in Maize. *Sustainability* **2020**, *12*, 6286, doi:10.3390/su12156286.
107. Danish, S.; Zafar-ul-Hye, M.; Mohsin, F.; Hussain, M. ACC-Deaminase Producing Plant Growth Promoting Rhizobacteria and Biochar Mitigate Adverse Effects of Drought Stress on Maize Growth. *PLoS ONE* **2020**, *15*, e0230615, doi:10.1371/journal.pone.0230615.
108. de Souza, R.; Meyer, J.; Schoenfeld, R.; da Costa, P.B.; Passaglia, L.M.P. Characterization of Plant Growth-Promoting Bacteria Associated with Rice Cropped in Iron-Stressed Soils. *Ann Microbiol* **2015**, *65*, 951–964, doi:10.1007/s13213-014-0939-3.
109. de Zélicourt, A.; Synek, L.; Saad, M.M.; Alzubaidy, H.; Jalal, R.; Xie, Y.; Andrés-Barrao, C.; Rolli, E.; Guerard, F.; Mariappan, K.G.; et al. Ethylene Induced Plant Stress Tolerance by Enterobacter Sp. SA187 Is Mediated by 2‐keto‐4‐methylthiobutyric Acid Production. *PLoS Genet* **2018**, *14*, e1007273, doi:10.1371/journal.pgen.1007273.
110. Devi, N.S.A.; Kumutha, K.; Anandham, R.; Krishnamoorthy, R. Induction of Moisture Stress Tolerance by Bacillus and Paenibacillus in Pigeon Pea (Cajanus Cajan. L). *3 Biotech* **2021**, *11*, 355, doi:10.1007/s13205-021-02901-w.
111. Devi, N.S.A.; Kumutha, K.; Anandham, R.; Krishnamoorthy, R.; Babu, R. Plant Growth Promoting Traits of Firmibacteria under Drought Stress. *Res J Agric Sci* 9 **2018**, *9*, 1294-1299.
112. Dey, P.; Datta, D.; Saha, D.; Parida, S.; Panda, D. Plant-Endophyte Interaction and Its Application to Abiotic Stress Management of Crop Plants. *Int.J.Curr.Microbiol.App.Sci* **2019**, *8*, 2708–2716, doi:10.20546/ijcmas.2019.807.332.
113. DHULL, S., SHEORAN, H. S., KAKAR, R., & GERA, R. Screening and characterisation of ACC deaminase producing rhizobacteria from root nodules of clusterbean (Cyamopsis tetragonoloba). *Annals of Plant and Soil Research* **2018**, *20*(3), 254-257.
114. Dı́az, J.; ten Have, A.; van Kan, J.A.L. The Role of Ethylene and Wound Signaling in Resistance of Tomato to *Botrytis Cinerea*. *Plant Physiology* **2002**, *129*, 1341–1351, doi:10.1104/pp.001453.
115. Dimkpa, C.; Weinand, T.; Asch, F. Plant-Rhizobacteria Interactions Alleviate Abiotic Stress Conditions: Plant-Rhizobacteria Interactions. *Plant, Cell & Environment* **2009**, *32*, 1682–1694, doi:10.1111/j.1365-3040.2009.02028.x.
116. Dixit, R.; Agrawal, L.; Gupta, S.; Kumar, M.; Yadav, S.; Chauhan, P.S.; Nautiyal, C.S. Southern Blight Disease of Tomato Control by 1-Aminocyclopropane-1-Carboxylate (ACC) Deaminase Producing *Paenibacillus Lentimorbus* B-30488. *Plant Signaling & Behavior* **2016**, *11*, e1113363, doi:10.1080/15592324.2015.1113363.
117. Dixit, R.; Agrawal, L.; Srivastava, S.; Chauhan, P.S. *Paenibacillus Lentimorbus* Enhanced Abiotic Stress Tolerance Through Lateral Root Formation and Phytohormone Regulation. *J Plant Growth Regul* **2021**, doi:10.1007/s00344-021-10439-7.
118. Dodd, I.C.; Ruiz-Lozano, J.M. Microbial Enhancement of Crop Resource Use Efficiency. *Current Opinion in Biotechnology* **2012**, *23*, 236–242, doi:10.1016/j.copbio.2011.09.005.
119. Dudeja, S.S.; Giri, R.; Saini, R.; Suneja-Madan, P.; Kothe, E. Interaction of Endophytic Microbes with Legumes. *J. Basic Microbiol.* **2012**, *52*, 248–260, doi:10.1002/jobm.201100063.
120. Dudeja, S.S.; Suneja‐Madan, P.; Paul, M.; Maheswari, R.; Kothe, E. Bacterial Endophytes: Molecular Interactions with Their Hosts. *J Basic Microbiol* **2021**, *61*, 475–505, doi:10.1002/jobm.202000657.
121. Egamberdieva, D.; Wirth, S.J.; Alqarawi, A.A.; Abd_Allah, E.F.; Hashem, A. Phytohormones and Beneficial Microbes: Essential Components for Plants to Balance Stress and Fitness. *Front. Microbiol.* **2017**, *8*, 2104, doi:10.3389/fmicb.2017.02104.
122. Eichmann, R.; Richards, L.; Schäfer, P. Hormones as Go‐betweens in Plant Microbiome Assembly. *Plant J* **2021**, *105*, 518–541, doi:10.1111/tpj.15135.
123. Eida, A.A.; Alzubaidy, H.S.; de Zélicourt, A.; Synek, L.; Alsharif, W.; Lafi, F.F.; Hirt, H.; Saad, M.M. Phylogenetically Diverse Endophytic Bacteria from Desert Plants Induce Transcriptional Changes of Tissue-Specific Ion Transporters and Salinity Stress in Arabidopsis Thaliana. *Plant Science* **2019**, *280*, 228–240, doi:10.1016/j.plantsci.2018.12.002.
124. El-Esawi, M.; Alaraidh, I.; Alsahli, A.; Alzahrani, S.; Ali, H.; Alayafi, A.; Ahmad, M. *Serratia Liquefaciens* KM4 Improves Salt Stress Tolerance in Maize by Regulating Redox Potential, Ion Homeostasis, Leaf Gas Exchange and Stress-Related Gene Expression. *IJMS* **2018**, *19*, 3310, doi:10.3390/ijms19113310.
125. El-Esawi, M.A.; Al-Ghamdi, A.A.; Ali, H.M.; Alayafi, A.A. *Azospirillum Lipoferum* FK1 Confers Improved Salt Tolerance in Chickpea (*Cicer Arietinum* L.) by Modulating Osmolytes, Antioxidant Machinery and Stress-Related Genes Expression. *Environmental and Experimental Botany* **2019**, *159*, 55–65, doi:10.1016/j.envexpbot.2018.12.001.
126. Enagbonma, B.J.; Babalola, O.O. Unveiling Plant-Beneficial Function as Seen in Bacteria Genes from Termite Mound Soil. *J Soil Sci Plant Nutr* **2020**, *20*, 421–430, doi:10.1007/s42729-019-00124-w.
127. Enebe, M.C.; Babalola, O.O. The Influence of Plant Growth-Promoting Rhizobacteria in Plant Tolerance to Abiotic Stress: A Survival Strategy. *Appl Microbiol Biotechnol* **2018**, *102*, 7821–7835, doi:10.1007/s00253-018-9214-z.
128. Esmaeel, Q.; Miotto, L.; Rondeau, M.; Leclère, V.; Clément, C.; Jacquard, C.; Sanchez, L.; Barka, E.A. Paraburkholderia Phytofirmans PsJN-Plants Interaction: From Perception to the Induced Mechanisms. *Front. Microbiol.* **2018**, *9*, 2093, doi:10.3389/fmicb.2018.02093.
129. Etesami, H.; Glick, B.R. Halotolerant Plant Growth–Promoting Bacteria: Prospects for Alleviating Salinity Stress in Plants. *Environmental and Experimental Botany* **2020**, *178*, 104124, doi:10.1016/j.envexpbot.2020.104124.
130. Etesami, H.; Hosseini, H.M.; Alikhani, H.A.; Mohammadi, L. Bacterial Biosynthesis of 1-Aminocyclopropane-1-Carboxylate (ACC) Deaminase and Indole-3-Acetic Acid (IAA) as Endophytic Preferential Selection Traits by Rice Plant Seedlings. *J Plant Growth Regul* **2014**, *33*, 654–670, doi:10.1007/s00344-014-9415-3.
131. Eun, H.-D.; Ali, S.; Jung, H.; Kim, K.; Kim, W.-C. Profiling of ACC Synthase Gene (ACS11) Expression in Arabidopsis Induced by Abiotic Stresses. *Appl Biol Chem* **2019**, *62*, 42, doi:10.1186/s13765-019-0450-4.
132. Fahad, S.; Hussain, S.; Bano, A.; Saud, S.; Hassan, S.; Shan, D.; Khan, F.A.; Khan, F.; Chen, Y.; Wu, C.; et al. Potential Role of Phytohormones and Plant Growth-Promoting Rhizobacteria in Abiotic Stresses: Consequences for Changing Environment. *Environ Sci Pollut Res* **2015**, *22*, 4907–4921, doi:10.1007/s11356-014-3754-2.
133. Fan, D.; Smith, D.L. Characterization of Selected Plant Growth-Promoting Rhizobacteria and Their Non-Host Growth Promotion Effects. *Microbiol Spectr* **2021**, *9*, e00279-21, doi:10.1128/Spectrum.00279-21.
134. Farahat, M.G.; Mahmoud, M.K.; Youseif, S.H.; Saleh, S.A.; Kamel, Z. Alleviation of Salinity Stress in Wheat by ACC Deaminase-Producing Bacillus Aryabhattai EWR29 with Multifarious Plant Growth-Promoting Attributes. *Plant Archives,* **2020**, 20, 417-429.
135. Farrar, K.; Bryant, D.; Cope‐Selby, N. Understanding and Engineering Beneficial Plant–Microbe Interactions: Plant Growth Promotion in Energy Crops. *Plant Biotechnol J* **2014**, *12*, 1193–1206, doi:10.1111/pbi.12279.
136. Fathalla, A., & Sabry, S. Effect of 1-aminocyclopropane-1-carboxylic acid deaminase producing fluorescent pseudomonas on the growth of eggplant under drought stress. *Plant Archives* **2020**, *20*(1), 3389-3394.
137. Fernández-Llamosas, H.; Ibero, J.; Thijs, S.; Imperato, V.; Vangronsveld, J.; Díaz, E.; Carmona, M. Enhancing the Rice Seedlings Growth Promotion Abilities of Azoarcus Sp. CIB by Heterologous Expression of ACC Deaminase to Improve Performance of Plants Exposed to Cadmium Stress. *Microorganisms* **2020**, *8*, 1453, doi:10.3390/microorganisms8091453.
138. *Field Crops: Sustainable Management by PGPR*; Maheshwari, D.K., Dheeman, S., Eds.; Sustainable Development and Biodiversity; Springer International Publishing: Cham, 2019; Vol. 23; ISBN 978-3-030-30925-1.
139. Fiodor, A.; Singh, S.; Pranaw, K. The Contrivance of Plant Growth Promoting Microbes to Mitigate Climate Change Impact in Agriculture. *Microorganisms* **2021**, *9*, 1841, doi:10.3390/microorganisms9091841.
140. Fortt, J.; González, M.; Morales, P.; Araya, N.; Remonsellez, F.; Coba de la Peña, T.; Ostria-Gallardo, E.; Stoll, A. Bacterial Modulation of the Plant Ethylene Signaling Pathway Improves Tolerance to Salt Stress in Lettuce (*Lactuca Sativa* L.). *Front. Sustain. Food Syst.* **2022**, *6*, 768250, doi:10.3389/fsufs.2022.768250.
141. Fouda, A.; Hassan, S.E.D.; Eid, A.M.; El-Din Ewais, E. The Interaction Between Plants and Bacterial Endophytes Under Salinity Stress. In *Bioactive Molecules in Food*; Mérillon, J.-M., Ramawat, K.G., Eds.; Reference Series in Phytochemistry; Springer International Publishing: Cham, 2019; pp. 1–17 ISBN 978-3-319-54528-8.
142. Gamalero, E.; Berta, G.; Massa, N.; Glick, B.R.; Lingua, G. Synergistic Interactions between the ACC Deaminase-Producing Bacterium Pseudomonas Putida UW4 and the AM Fungus Gigaspora Rosea Positively Affect Cucumber Plant Growth: Synergistic Interactions between P. Putida UW4 and G. Rosea. *FEMS Microbiology Ecology* **2008**, *64*, 459–467, doi:10.1111/j.1574-6941.2008.00485.x.
143. Gamalero, E.; Bona, E.; Todeschini, V.; Lingua, G. Saline and Arid Soils: Impact on Bacteria, Plants, and Their Interaction. *Biology* **2020**, *9*, 116, doi:10.3390/biology9060116.
144. Gamalero, E.; Glick, B.R. Recent Advances in Bacterial Amelioration of Plant Drought and Salt Stress. *Biology* **2022**, *11*, 437, doi:10.3390/biology11030437.
145. Gamalero, E.; Lingua, G.; Berta, G.; Glick, B.R. Beneficial Role of Plant Growth Promoting Bacteria and Arbuscular Mycorrhizal Fungi on Plant Responses to Heavy Metal Stress. *Can. J. Microbiol.* **2009**, *55*, 501–514, doi:10.1139/W09-010.
146. Gebauer, L.; Bouffaud, M.-L.; Ganther, M.; Yim, B.; Vetterlein, D.; Smalla, K.; Buscot, F.; Heintz-Buschart, A.; Tarkka, M.T. Soil Texture, Sampling Depth and Root Hairs Shape the Structure of ACC Deaminase Bacterial Community Composition in Maize Rhizosphere. *Front. Microbiol.* **2021**, *12*, 616828, doi:10.3389/fmicb.2021.616828.
147. Gerszberg, A.; Hnatuszko-Konka, K. Tomato Tolerance to Abiotic Stress: A Review of Most Often Engineered Target Sequences. *Plant Growth Regul* **2017**, *83*, 175–198, doi:10.1007/s10725-017-0251-x.
148. Ghanem, M.E.; Hichri, I.; Smigocki, A.C.; Albacete, A.; Fauconnier, M.-L.; Diatloff, E.; Martinez-Andujar, C.; Lutts, S.; Dodd, I.C.; Pérez-Alfocea, F. Root-Targeted Biotechnology to Mediate Hormonal Signalling and Improve Crop Stress Tolerance. *Plant Cell Rep* **2011**, *30*, 807–823, doi:10.1007/s00299-011-1005-2.
149. Giri, B., Sharma, M.P. *Plant Stress Biology: Strategies and Trends*. Eds.; Springer Singapore: Singapore, 2020; ISBN 9789811593796.
150. Giri, B., Varma, A. *Microorganisms in Saline Environments: Strategies and Functions*; Eds.; Soil Biology; Springer International Publishing: Cham, 2019; Vol. 56; ISBN 978-3-030-18974-7.
151. Glick, B.R. Bacteria with ACC Deaminase Can Promote Plant Growth and Help to Feed the World. *Microbiological Research* **2014**, *169*, 30–39, doi:10.1016/j.micres.2013.09.009.
152. Glick, B.R. CHANGES IN PLANT GROWTH AND DEVELOPMENT BY RHIZOSPHERE BACTERIA THAT MODIFY PLANT ETHYLENE LEVELS. *Acta Hortic.* **2004**, 265–273, doi:10.17660/ActaHortic.2004.631.33.
153. Glick, B.R. Modulation of Plant Ethylene Levels by the Bacterial Enzyme ACC Deaminase. *FEMS Microbiology Letters* **2005**, *251*, 1–7, doi:10.1016/j.femsle.2005.07.030.
154. Glick, B.R. Plant Growth-Promoting Bacteria: Mechanisms and Applications. *Scientifica* **2012**, *2012*, 1–15, doi:10.6064/2012/963401.
155. Glick, B.R.; Cheng, Z.; Czarny, J.; Duan, J. Promotion of Plant Growth by ACC Deaminase-Producing Soil Bacteria. *Eur J Plant Pathol* **2007**, *119*, 329–339, doi:10.1007/s10658-007-9162-4.
156. Glick, B.R.; Nascimento, F.X. Pseudomonas 1-Aminocyclopropane-1-Carboxylate (ACC) Deaminase and Its Role in Beneficial Plant-Microbe Interactions. *Microorganisms* **2021**, *9*, 2467, doi:10.3390/microorganisms9122467.
157. Glick, B.R.; Penrose, D.M. The Use of ACC Deaminase-Containing Plant Growth-Promoting Bacteria to Protect Plants Against the Deleterious Effects of Ethylene. In *Plant Surface Microbiology*; Varma, A., Abbott, L., Werner, D., Hampp, R., Eds.; Springer Berlin Heidelberg: Berlin, Heidelberg, **2008**; pp. 133–144 ISBN 978-3-540-74050-6.
158. Glick, B.R.; Stearns, J.C. Making Phytoremediation Work Better: Maximizing a Plant’s Growth Potential in the Midst of Adversity. *International Journal of Phytoremediation* **2011**, *13*, 4–16, doi:10.1080/15226514.2011.568533.
159. Glick, B.R.; Todorovic, B.; Czarny, J.; Cheng, Z.; Duan, J.; McConkey, B. Promotion of Plant Growth by Bacterial ACC Deaminase. *Critical Reviews in Plant Sciences* **2007**, *26*, 227–242, doi:10.1080/07352680701572966.
160. Gokul, A.; Niekerk, L.-A.; Carelse, M.F.; Keyster, M. Transgenic Technology for Efficient Abiotic Stress Tolerance in Plants. In *Transgenic Technology Based Value Addition in Plant Biotechnology*; Elsevier, **2020**; pp. 95–122 ISBN 978-0-12-818632-9.
161. Gong, Y.; Chen, L.-J.; Pan, S.-Y.; Li, X.-W.; Xu, M.-J.; Zhang, C.-M.; Xing, K.; Qin, S. Antifungal Potential Evaluation and Alleviation of Salt Stress in Tomato Seedlings by a Halotolerant Plant Growth-Promoting Actinomycete Streptomyces Sp. KLBMP5084. *Rhizosphere* **2020**, *16*, 100262, doi:10.1016/j.rhisph.2020.100262.
162. Gontia-Mishra, I.; Sapre, S.; Kachare, S.; Tiwari, S. Molecular Diversity of 1-Aminocyclopropane-1-Carboxylate (ACC) Deaminase Producing PGPR from Wheat (Triticum Aestivum L.) Rhizosphere. *Plant Soil* **2017**, *414*, 213–227, doi:10.1007/s11104-016-3119-3.
163. Gontia-Mishra, I.; Sapre, S.; Sharma, A.; Tiwari, S. Amelioration of Drought Tolerance in Wheat by the Interaction of Plant Growth-Promoting Rhizobacteria. *Plant Biol J* **2016**, *18*, 992–1000, doi:10.1111/plb.12505.
164. Gontia-Mishra, I.; Sasidharan, S.; Tiwari, S. Recent Developments in Use of 1-Aminocyclopropane-1-Carboxylate (ACC) Deaminase for Conferring Tolerance to Biotic and Abiotic Stress. *Biotechnol Lett* **2014**, *36*, 889–898, doi:10.1007/s10529-014-1458-9.
165. Govindasamy, V.; Raina, S.K.; George, P.; Kumar, M.; Rane, J.; Minhas, P.S.; Vittal, K.P.R. Functional and Phylogenetic Diversity of Cultivable Rhizobacterial Endophytes of Sorghum [Sorghum Bicolor (L.) Moench]. *Antonie van Leeuwenhoek* **2017**, *110*, 925–943, doi:10.1007/s10482-017-0864-0.
166. Govindasamy, V.; Senthilkumar, M.; Annapurna, K. Effect of Mustard Rhizobacteria on Wheat Growth Promotion under Cadmium Stress: Characterization of *acdS* Gene Coding ACC Deaminase. *Ann Microbiol* **2015**, *65*, 1679–1687, doi:10.1007/s13213-014-1007-8.
167. Gowtham, H.G.; Singh, B.S.; Murali, M.; Shilpa, N.; Prasad, M.; Aiyaz, M.; Amruthesh, K.N.; Niranjana, S.R. Induction of Drought Tolerance in Tomato upon the Application of ACC Deaminase Producing Plant Growth Promoting Rhizobacterium Bacillus Subtilis Rhizo SF 48. *Microbiological Research* **2020**, *234*, 126422, doi:10.1016/j.micres.2020.126422.
168. Goyal, D.; Kumar, S.; Meena, D.; Solanki, S.S.; Swaroop, S.; Pandey, J. Selection of ACC Deaminase Positive, Thermohalotolerant and Drought Tolerance Enhancing Plant Growth‐promoting Bacteria from Rhizospheres of *Cyamopsis Tetragonoloba* Grown in Arid Regions. *Letters Applied Microbiology* **2021**, *74*, 519–535, doi:10.1111/lam.13633.
169. Goyal, D.; Prakash, O.; Pandey, J. Rhizospheric Microbial Diversity: An Important Component for Abiotic Stress Management in Crop Plants Toward Sustainable Agriculture. In *New and Future Developments in Microbial Biotechnology and Bioengineering*; Elsevier, 2019; pp. 115–134 ISBN 978-0-444-64191-5.
170. Grichko, V.P.; Glick, B.R. Amelioration of Flooding Stress by ACC Deaminase-Containingplant Growth-Promoting Bacteria. *Plant Physiology and Biochemistry* **2001**, *39*, 11–17, doi:10.1016/S0981-9428(00)01212-2.
171. Grichko, V.P.; Glick, B.R. Flooding Tolerance of Transgenic Tomato Plants Expressing the Bacterial Enzyme ACC Deaminase Controlledby the 35S, RolD or PRB-1b Promoter. *Plant Physiology and Biochemistry* **2001**, *39*, 19–25, doi:10.1016/S0981-9428(00)01217-1.
172. Grobelak, A.; Kokot, P.; Świątek, J.; Jaskulak, M.; Rorat, A. Bacterial ACC Deaminase Activity in Promoting Plant Growth on Areas Contaminated with Heavy Metals. *J. Ecol. Eng.* **2018**, *19*, 150–157, doi:10.12911/22998993/89818.
173. Gu, T.; Yu, H.; Li, F.; Zeng, W.; Wu, X.; Shen, L.; Yu, R.; Liu, Y.; Li, J. Antimony-Oxidizing Bacteria Alleviate Sb Stress in Arabidopsis by Attenuating Sb Toxicity and Reducing Sb Uptake. *Plant Soil* **2020**, *452*, 397–412, doi:10.1007/s11104-020-04569-2.
174. Guo, D.-J.; Singh, R.K.; Singh, P.; Li, D.-P.; Sharma, A.; Xing, Y.-X.; Song, X.-P.; Yang, L.-T.; Li, Y.-R. Complete Genome Sequence of *Enterobacter Roggenkampii* ED5, a Nitrogen Fixing Plant Growth Promoting Endophytic Bacterium With Biocontrol and Stress Tolerance Properties, Isolated From Sugarcane Root. *Front. Microbiol.* **2020**, *11*, 580081, doi:10.3389/fmicb.2020.580081.
175. Gupta, A.; Bano, A.; Rai, S.; Kumar, M.; Ali, J.; Sharma, S.; Pathak, N. ACC Deaminase Producing Plant Growth Promoting Rhizobacteria Enhance Salinity Stress Tolerance in Pisum Sativum. *3 Biotech* **2021**, *11*, 514, doi:10.1007/s13205-021-03047-5.
176. Gupta, A.; Bano, A.; Rai, S.; Mishra, R.; Singh, M.; Sharma, S.; Pathak, N. Mechanistic Insights of Plant-Microbe Interaction towards Drought and Salinity Stress in Plants for Enhancing the Agriculture Productivity. *Plant Stress* **2022**, *4*, 100073, doi:10.1016/j.stress.2022.100073.
177. Gupta, A.; Gopal, M.; Thomas, G.V.; Manikandan, V.; Gajewski, J.; Thomas, G.; Seshagiri, S.; Schuster, S.C.; Rajesh, P.; Gupta, R. Whole Genome Sequencing and Analysis of Plant Growth Promoting Bacteria Isolated from the Rhizosphere of Plantation Crops Coconut, Cocoa and Arecanut. *PLoS ONE* **2014**, *9*, e104259, doi:10.1371/journal.pone.0104259.
178. Gupta, A.; Singh, S.K.; Singh, M.K.; Singh, V.K.; Modi, A.; Singh, P.K.; Kumar, A. Plant Growth–Promoting Rhizobacteria and Their Functional Role in Salinity Stress Management. In *Abatement of Environmental Pollutants*; Elsevier, 2020; pp. 151–160 ISBN 978-0-12-818095-2.
179. Gupta, A.; Vandana, P. Effect of PGPR Isolates on Plant Growth Promotion in Relation to Salinity Stress. **2019**, *8*, 9.
180. Gupta, S.; Pandey, S. ACC Deaminase Producing Bacteria With Multifarious Plant Growth Promoting Traits Alleviates Salinity Stress in French Bean (Phaseolus Vulgaris) Plants. *Front. Microbiol.* **2019**, *10*, 1506, doi:10.3389/fmicb.2019.01506.
181. Gupta, S.; Pandey, S. Enhanced Salinity Tolerance in the Common Bean (Phaseolus Vulgaris) Plants Using Twin ACC Deaminase Producing Rhizobacterial Inoculation. *Rhizosphere* **2020**, *16*, 100241, doi:10.1016/j.rhisph.2020.100241.
182. Gupta, S.; Pandey, S. Unravelling the Biochemistry and Genetics of ACC Deaminase-An Enzyme Alleviating the Biotic and Abiotic Stress in Plants. *Plant Gene* **2019**, *18*, 100175, doi:10.1016/j.plgene.2019.100175.
183. Gururani, M.A.; Upadhyaya, C.P.; Baskar, V.; Venkatesh, J.; Nookaraju, A.; Park, S.W. Plant Growth-Promoting Rhizobacteria Enhance Abiotic Stress Tolerance in Solanum Tuberosum Through Inducing Changes in the Expression of ROS-Scavenging Enzymes and Improved Photosynthetic Performance. *J Plant Growth Regul* **2013**, *32*, 245–258, doi:10.1007/s00344-012-9292-6.
184. Habib, S.H.; Kausar, H.; Saud, H.M. Plant Growth-Promoting Rhizobacteria Enhance Salinity Stress Tolerance in Okra through ROS-Scavenging Enzymes. *BioMed Research International* **2016**, *2016*, 1–10, doi:10.1155/2016/6284547.
185. Haggag, W.M. Agriculture Biotechnology for Management of Multiple Biotic and Abiotic Environmental Stress in Crops. *J. Chem. Pharm. Res.* **2015**, *7*, 882-889.
186. Hahm, M.-S.; Son, J.-S.; Hwang, Y.-J.; Kwon, D.-K.; Ghim, S.-Y. Alleviation of Salt Stress in Pepper (Capsicum Annum L.) Plants by Plant Growth-Promoting Rhizobacteria. *Journal of Microbiology and Biotechnology* **2017**, *27*, 1790–1797, doi:10.4014/jmb.1609.09042.
187. Haidar, B.; Ferdous, M.; Fatema, B.; Ferdous, A.S.; Islam, M.R.; Khan, H. Population Diversity of Bacterial Endophytes from Jute (Corchorus Olitorius) and Evaluation of Their Potential Role as Bioinoculants. *Microbiological Research* **2018**, *208*, 43–53, doi:10.1016/j.micres.2018.01.008.
188. Haiquan, H.; Meijuan, H.; Guosheng, F.; Xuefei, L.; Jihua, W.; Qing, D.; Qixiang, Z. Isolation and Characterization of 1-Aminocyclopropane-1-Carboxylate (ACC) Deaminase-Containing Plant Growth-Promoting Rhizobacteria from Carnation Soil and Roots. *Afr. J. Microbiol. Res.* **2013**, *7*, 5664–5668, doi:10.5897/AJMR2013.1573.
189. Hale, L.; Luth, M.; Crowley, D. Biochar Characteristics Relate to Its Utility as an Alternative Soil Inoculum Carrier to Peat and Vermiculite. *Soil Biology and Biochemistry* **2015**, *81*, 228–235, doi:10.1016/j.soilbio.2014.11.023.
190. Hamid, B.; Zaman, M.; Farooq, S.; Fatima, S.; Sayyed, R.Z.; Baba, Z.A.; Sheikh, T.A.; Reddy, M.S.; El Enshasy, H.; Gafur, A.; et al. Bacterial Plant Biostimulants: A Sustainable Way towards Improving Growth, Productivity, and Health of Crops. *Sustainability* **2021**, *13*, 2856, doi:10.3390/su13052856.
191. Han, L.; Zhang, H.; Xu, Y.; Li, Y.; Zhou, J. Biological Characteristics and Salt-Tolerant Plant Growth-Promoting Effects of an ACC Deaminase-Producing Burkholderia Pyrrocinia Strain Isolated from the Tea Rhizosphere. *Arch Microbiol* **2021**, *203*, 2279–2290, doi:10.1007/s00203-021-02204-x.
192. Han, Y.; Wang, R.; Yang, Z.; Zhan, Y.; Ma, Y.; Ping, S.; Zhang, L.; Lin, M.; Yan, Y. 1-Aminocyclopropane-1-Carboxylate Deaminase from Pseudomonas Stutzeri A1501 Facilitates the Growth of Rice in the Presence of Salt or Heavy Metals. *Journal of Microbiology and Biotechnology* **2015**, *25*, 1119–1128, doi:10.4014/jmb.1412.12053.
193. Hao, Y.; Charles, T.C.; Glick, B.R. ACC Deaminase Activity in Avirulent *Agrobacterium Tumefaciens* D3. *Can. J. Microbiol.* **2011**, *57*, 278–286, doi:10.1139/w11-006.
194. Haque, M. M., Mosharaf, M. K., Khatun, M., Haque, M. A., Biswas, M. S., Islam, M. S., ... & Siddiquee, M. A. Biofilm producing rhizobacteria with multiple plant growth-promoting traits promote growth of tomato under water-deficit stress. *Frontiers in Microbiology* **2020**, *11*, 542053.
195. Haque, Md.M.; Biswas, Md.S.; Mosharaf, M.K.; Haque, Md.A.; Islam, Md.S.; Nahar, K.; Islam, Md.M.; Shozib, H.B.; Islam, Md.M.; Ferdous-E-Elahi Halotolerant Biofilm-Producing Rhizobacteria Mitigate Seawater-Induced Salt Stress and Promote Growth of Tomato. *Sci Rep* **2022**, *12*, 5599, doi:10.1038/s41598-022-09519-9.
196. Hardoim, P.R.; van Overbeek, L.S.; Elsas, J.D. van Properties of Bacterial Endophytes and Their Proposed Role in Plant Growth. *Trends in Microbiology* **2008**, *16*, 463–471, doi:10.1016/j.tim.2008.07.008.
197. Hariprasad, P.; Gowtham, H.G.; Gourav, C. Beneficial Plant-Associated Bacteria Modulate Host Hormonal System Enhancing Plant Resistance toward Abiotic Stress. In *Biocontrol Agents and Secondary Metabolites*; Elsevier, **2021**; pp. 113–151 ISBN 978-0-12-822919-4.
198. Harman, G.E.; Doni, F.; Khadka, R.B.; Uphoff, N. Endophytic Strains of *Trichoderma* Increase Plants’ Photosynthetic Capability. *J Appl Microbiol* **2021**, *130*, 529–546, doi:10.1111/jam.14368.
199. Haroon, U.; Khizar, M.; Liaquat, F.; Ali, M.; Akbar, M.; Tahir, K.; Batool, S.S.; Kamal, A.; Chaudhary, H.J.; Munis, M.F.H. Halotolerant Plant Growth-Promoting Rhizobacteria Induce Salinity Tolerance in Wheat by Enhancing the Expression of SOS Genes. *J Plant Growth Regul* **2021**, 1-14, doi:10.1007/s00344-021-10457-5.
200. Hartman, K.; Tringe, S.G. Interactions between Plants and Soil Shaping the Root Microbiome under Abiotic Stress. *Biochemical Journal* **2019**, *476*, 2705–2724, doi:10.1042/BCJ20180615.
201. Hartmann, A.; Klink, S.; Rothballer, M. Plant Growth Promotion and Induction of Systemic Tolerance to Drought and Salt Stress of Plants by Quorum Sensing Auto-Inducers of the N-Acyl-Homoserine Lactone Type: Recent Developments. *Front. Plant Sci.* **2021**, *12*, 683546, doi:10.3389/fpls.2021.683546.
202. Hasanuzzaman, M., Nahar, K., Hossain, Md.A*. Wheat Production in Changing Environments: Responses, Adaptation and Tolerance*; Eds.; Springer Singapore: Singapore, **2019**; ISBN 9789811368820.
203. Hasanuzzaman, M.,*.The Plant Family Brassicaceae: Biology and Physiological Responses to Environmental Stresses*; Ed.; Springer Singapore: Singapore, 2020; ISBN 9789811563447.
204. Hashem, A. Bacillus Subtilis: A Plant-Growth Promoting Rhizobacterium That Also Impacts Biotic Stress. *Saudi Journal of Biological Sciences* **2019**, *6*, 1291-1297, doi: 10.106/j.sbs.2019.05.004.
205. Hermosa, R.; Rubio, M.B. The Contribution of Trichoderma to Balancing the Costs of Plant Growth and Defense. *International Microbiology* **2013**, 69–80, doi:10.2436/20.1501.01.181.
206. Hermosa, R.; Viterbo, A.; Chet, I.; Monte, E. Plant-Beneficial Effects of Trichoderma and of Its Genes. *Microbiology* **2012**, *158*, 17–25, doi:10.1099/mic.0.052274-0.
207. Hernández, J.A.; Diaz‐Vivancos, P.; Acosta‐Motos, J.R.; Barba‐Espín, G. Where Biotic and Abiotic Stress Responses Converge: Common Patterns in Response to Salinity and  *Plum Pox Virus*  Infection in Pea and Peach Plants. *Ann Appl Biol* **2021**, *178*, 281–292, doi:10.1111/aab.12667.
208. Hernández-Canseco, J.; Bautista-Cruz, A.; Sánchez-Mendoza, S.; Aquino-Bolaños, T.; Sánchez-Medina, P.S. Plant Growth-Promoting Halobacteria and Their Ability to Protect Crops from Abiotic Stress: An Eco-Friendly Alternative for Saline Soils. *Agronomy* **2022**, *12*, 804, doi:10.3390/agronomy12040804.
209. Heydarian, Z.; Gruber, M.; Coutu, C.; Glick, B.R.; Hegedus, D.D. Gene Expression Patterns in Shoots of Camelina Sativa with Enhanced Salinity Tolerance Provided by Plant Growth Promoting Bacteria Producing 1-Aminocyclopropane-1-Carboxylate Deaminase or Expression of the Corresponding AcdS Gene. *Sci Rep* **2021**, *11*, 4260, doi:10.1038/s41598-021-83629-8.
210. Heydarian, Z.; Yu, M.; Gruber, M.; Coutu, C.; Robinson, S.J.; Hegedus, D.D. Changes in Gene Expression in Camelina Sativa Roots and Vegetative Tissues in Response to Salinity Stress. *Sci Rep* **2018**, *8*, 9804, doi:10.1038/s41598-018-28204-4.
211. Hontzeas, N.; Saleh, S.S.; Glick, B.R. Changes in Gene Expression in Canola Roots Induced by ACC-Deaminase-Containing Plant-Growth-Promoting Bacteria. *MPMI* **2004**, *17*, 865–871, doi:10.1094/MPMI.2004.17.8.865.
212. Hossain, A.; Ali, M.E.; Maitra, S.; Bhadra, P.; Rahman, Md.M.E.; Ali, S.; Aftab, T. The Role of Soil Microorganisms in Plant Adaptation to Abiotic Stresses: Current Scenario and Future Perspectives. In *Plant Perspectives to Global Climate Changes*; Elsevier, 2022; pp. 233–278 ISBN 978-0-323-85665-2.
213. Hua Guo, J.; Hao Jiang, C. The Plant Healthy and Safety Guards Plant Growth Promoting Rhizo Bacteria (PGPR). *Transcriptomics* **2015**, *3*, 109, doi:10.4172/2329-8936.1000109.
214. Hurst, C.J. *Microbes: The Foundation Stone of the Biosphere*; Ed.; Advances in Environmental Microbiology; Springer International Publishing: Cham, 2021; Vol. 8; ISBN 978-3-030-63511-4.
215. Husen, E.; Tri Wahyudi, A.; Suwanto, A.; Saraswati, R. Prospective Use of 1-Aminocyclopropane-1-Carboxylate Deaminase-Producing Bacteria for Plant Growth Promotion and Defense against Biotic and Abiotic Stresses in Peat-Soil-Agriculture. *MI* **2008**, *2*, 107–111, doi:10.5454/mi.2.3.2.
216. Hussain, S.; Huang, J.; Zhu, C.; Zhu, L.; Cao, X.; Hussain, S.; Ashraf, M.; Khaskheli, M.A.; Kong, Y.; Jin, Q.; et al. Pyridoxal 5′-Phosphate Enhances the Growth and Morpho-Physiological Characteristics of Rice Cultivars by Mitigating the Ethylene Accumulation under Salinity Stress. *Plant Physiology and Biochemistry* **2020**, *154*, 782–795, doi:10.1016/j.plaphy.2020.05.035.
217. Ilangumaran, G.; Smith, D.L. Plant Growth Promoting Rhizobacteria in Amelioration of Salinity Stress: A Systems Biology Perspective. *Front. Plant Sci.* **2017**, *8*, 1768, doi:10.3389/fpls.2017.01768.
218. Imperato, V.; Portillo-Estrada, M.; McAmmond, B.M.; Douwen, Y.; Van Hamme, J.D.; Gawronski, S.W.; Vangronsveld, J.; Thijs, S. Genomic Diversity of Two Hydrocarbon-Degrading and Plant Growth-Promoting Pseudomonas Species Isolated from the Oil Field of Bóbrka (Poland). *Genes* **2019**, *10*, 443, doi:10.3390/genes10060443.
219. Iqbal, A.; Mukherjee, M.; Rashid, J.; Khan, S.A.; Ali, M.A.; Arshad, M. Development of Plant-Microbe Phytoremediation System for Petroleum Hydrocarbon Degradation: An Insight from Alkb Gene Expression and Phytotoxicity Analysis. *Science of The Total Environment* **2019**, *671*, 696–704, doi:10.1016/j.scitotenv.2019.03.331.
220. Islam, F.; Yasmeen, T.; Ali, Q.; Ali, S.; Arif, M.S.; Hussain, S.; Rizvi, H. Influence of Pseudomonas Aeruginosa as PGPR on Oxidative Stress Tolerance in Wheat under Zn Stress. *Ecotoxicology and Environmental Safety* **2014**, *104*, 285–293, doi:10.1016/j.ecoenv.2014.03.008.
221. Islam, F.; Yasmeen, T.; Arif, M.S.; Ali, S.; Ali, B.; Hameed, S.; Zhou, W. Plant Growth Promoting Bacteria Confer Salt Tolerance in Vigna Radiata by Up-Regulating Antioxidant Defense and Biological Soil Fertility. *Plant Growth Regul* **2016**, *80*, 23–36, doi:10.1007/s10725-015-0142-y.
222. Islam, M.T., Rahman, M.M., Pandey, P., Boehme, M.H., Haesaert, G. *Bacilli and Agrobiotechnology: Phytostimulation and Biocontrol: Volume 2*; Eds.; Bacilli in Climate Resilient Agriculture and Bioprospecting; Springer International Publishing: Cham, 2019; ISBN 978-3-030-15174-4.
223. Jaemsaeng, R.; Jantasuriyarat, C.; Thamchaipenet, A. Molecular Interaction of 1-Aminocyclopropane-1-Carboxylate Deaminase (ACCD)-Producing Endophytic *Streptomyces* sp. GMKU 336 towards Salt-Stress Resistance of *Oryza sativa* L. cv. KDML105. *Sci Rep* **2018**, *8*, 1950, doi:10.1038/s41598-018-19799-9.
224. Jain, S.; Vaishnav, A.; Kasotia, A.; Kumari, S.; Gaur, R.K.; Choudhary, D.K. Rhizobacterium-Mediated Growth Promotion and Expression of Stress Enzymes in Glycine Max L. Merrill against Fusarium Wilt upon Challenge Inoculation. *World J Microbiol Biotechnol* **2014**, *30*, 399–406, doi:10.1007/s11274-013-1455-5.
225. Jaiswal, S.K.; Mohammed, M.; Ibny, F.Y.I.; Dakora, F.D. Rhizobia as a Source of Plant Growth-Promoting Molecules: Potential Applications and Possible Operational Mechanisms. *Front. Sustain. Food Syst.* **2021**, *4*, 619676, doi:10.3389/fsufs.2020.619676.
226. Jalil, S.U.; Ansari, M.I. Plant Microbiome and Its Functional Mechanism in Response to Environmental Stress. *Internation Journal of Green Pharmacy* **2018**, *12*, s81.
227. Jalili, F.; Khavazi, K.; Pazira, E.; Nejati, A.; Rahmani, H.A.; Sadaghiani, H.R.; Miransari, M. Isolation and Characterization of ACC Deaminase-Producing Fluorescent Pseudomonads, to Alleviate Salinity Stress on Canola (Brassica Napus L.) Growth. *Journal of Plant Physiology* **2009**, *166*, 667–674, doi:10.1016/j.jplph.2008.08.004.
228. Jamali, H.; Sharma, A.; Kashyap, P.L.; hi, R.; Srivastava, A.K. Exploitation of Multifarious Abiotic Stresses, Antagonistic Activity and Plant Growth Promoting Attributes of Bacillus Amyloliquefaciens AH53 for Sustainable Agriculture Production. *Int.J.Curr.Microbiol.App.Sci* **2018**, *7*, 751–763, doi:10.20546/ijcmas.2018.710.083.
229. Jamil, M. INDUCING DROUGHT TOLERANCE IN WHEAT THROUGH COMBINED USE OF L-TRYPTOPHAN AND Pseudomonas Fluorescens. *PAKJAS* **2018**, *55*, 331–337, doi:10.21162/PAKJAS/18.4980.
230. Jaroszuk-Ściseł, J.; Tyśkiewicz, R.; Nowak, A.; Ozimek, E.; Majewska, M.; Hanaka, A.; Tyśkiewicz, K.; Pawlik, A.; Janusz, G. Phytohormones (Auxin, Gibberellin) and ACC Deaminase In Vitro Synthesized by the Mycoparasitic Trichoderma DEMTkZ3A0 Strain and Changes in the Level of Auxin and Plant Resistance Markers in Wheat Seedlings Inoculated with This Strain Conidia. *IJMS* **2019**, *20*, 4923, doi:10.3390/ijms20194923.
231. Jatan, R.; Chauhan, P.S.; Lata, C. Pseudomonas Putida Modulates the Expression of MiRNAs and Their Target Genes in Response to Drought and Salt Stresses in Chickpea (Cicer Arietinum L.). *Genomics* **2019**, *111*, 509–519, doi:10.1016/j.ygeno.2018.01.007.
232. Jaya, D.K.; Giyanto, G.; Nurhidayat, N.; Antonius, S. Isolation, Identification, and Detection of ACC Deaminase Gene-Encoding Rhizobacteria from Rhizosphere of Stressed Pineapple. *Indones. J. Biotechnol.* **2019**, *24*, 17-25, doi:10.22146/ijbiotech.39018.
233. Jayakumar, A.; Nair, I.C.; Radhakrishnan, E.K. Environmental Adaptations of an Extremely Plant Beneficial Bacillus Subtilis Dcl1 Identified Through the Genomic and Metabolomic Analysis. *Microb Ecol* **2021**, *81*, 687–702, doi:10.1007/s00248-020-01605-7.
234. Jegan, S., et al. Rhizomicrobiome–a biological software to augment soil fertility and plant induced systemic tolerance under abiotic stress. *Microbes for plant stress management New India Publishing Agency* **2016***,* 25-53.
235. Jha, B.; Gontia, I.; Hartmann, A. The Roots of the Halophyte *Salicornia brachiata* Are a Source of New Halotolerant Diazotrophic Bacteria with Plant Growth-Promoting Potential. *Plant Soil* **2012**, *356*, 265–277, doi:10.1007/s11104-011-0877-9.
236. Jha, C. K., Sharma, P., Shukla, A., Parmar, P., Patel, R., Goswami, D., & Saraf, M.. Microbial enzyme, 1-aminocyclopropane-1-carboxylic acid (ACC) deaminase: an elixir for plant under stress. *Physiological and Molecular Plant Pathology* **2021**, *115*, 101664.
237. Jha, C.K.; Annapurna, K.; Saraf, M. Isolation of Rhizobacteria from Jatropha Curcas and Characterization of Produced ACC Deaminase. *J. Basic Microbiol.* **2012**, *52*, 285–295, doi:10.1002/jobm.201100113.
238. Jha, P.N.; Gupta, G.; Jha, P.; Mehrotra, R. Association of Rhizospheric/Endophytic Bacteria with Plants: A Potential Gateway to Sustainable Agriculture. *Greener Journal of Agricultural Sciences* **201*3***, *3*, 73-44.
239. Ji, J.; Yuan, D.; Jin, C.; Wang, G.; Li, X.; Guan, C. Enhancement of Growth and Salt Tolerance of Rice Seedlings (Oryza Sativa L.) by Regulating Ethylene Production with a Novel Halotolerant PGPR Strain Glutamicibacter Sp. YD01 Containing ACC Deaminase Activity. *Acta Physiol Plant* **2020**, *42*, 42, doi:10.1007/s11738-020-3034-3.
240. Jiroutova, P.; Oklestkova, J.; Strnad, M. Crosstalk between Brassinosteroids and Ethylene during Plant Growth and under Abiotic Stress Conditions. *IJMS* **2018**, *19*, 3283, doi:10.3390/ijms19103283.
241. Joshi, R.; Singla-Pareek, S.L.; Pareek, A. Engineering Abiotic Stress Response in Plants for Biomass Production. *Journal of Biological Chemistry* **2018**, *293*, 5035–5043, doi:10.1074/jbc.TM117.000232.
242. Kamala-Kannan, S.; Lee, K.-J.; Park, S.-M.; Chae, J.-C.; Yun, B.-S.; Lee, Y.H.; Park, Y.-J.; Oh, B.-T. Characterization of ACC Deaminase Gene in *Pseudomonas Entomophila* Strain PS-PJH Isolated from the Rhizosphere Soil. *J. Basic Microbiol.* **2010**, 200-5, doi:10.1002/jobm.200900171.
243. Kambiranda, D. M.; Vasanthhaiah, H. K. N.; Katam, R. et al. *Impact of Drought Stress on Peanut (Arachis Hypogaea L.) Productivity and Food Safety.*; INTECH Open Access Publisher, 2011; ISBN 978-953-307-779-6.
244. Kang, B.G.; Kim, W.T.; Yun, H.S.; Chang, S.C. Use of Plant Growth-Promoting Rhizobacteria to Control Stress Responses of Plant Roots. *Plant Biotechnol Rep* **2010**, *4*, 179–183, doi:10.1007/s11816-010-0136-1.
245. Kang, S.-M.; Radhakrishnan, R.; Khan, A.L.; Kim, M.-J.; Park, J.-M.; Kim, B.-R.; Shin, D.-H.; Lee, I.-J. Gibberellin Secreting Rhizobacterium, Pseudomonas Putida H-2-3 Modulates the Hormonal and Stress Physiology of Soybean to Improve the Plant Growth under Saline and Drought Conditions. *Plant Physiology and Biochemistry* **2014**, *84*, 115–124, doi:10.1016/j.plaphy.2014.09.001.
246. Kang, S.-M.; Shahzad, R.; Bilal, S.; Khan, A.L.; Park, Y.-G.; Lee, K.-E.; Asaf, S.; Khan, M.A.; Lee, I.-J. Indole-3-Acetic-Acid and ACC Deaminase Producing Leclercia Adecarboxylata MO1 Improves Solanum Lycopersicum L. Growth and Salinity Stress Tolerance by Endogenous Secondary Metabolites Regulation. *BMC Microbiol* **2019**, *19*, 80, doi:10.1186/s12866-019-1450-6.
247. Karnwal, A. Screening and Identification of Abiotic Stress-Responsive Efficient Antifungal Pseudomonas Spp. from Rice Rhizospheric Soil. *bta* **2021**, *102*, 5–19, doi:10.5114/bta.2021.103758.
248. Karnwal, A.; Bekir, T. Effect of plant hormones on the cambial activity of Cerasus vulgari Miller under stress conditions witn Zn. *Journal of Plant Protection Research* **2019**, *53*, 293-303, doi:10.24425/JPPR.2019.129747.
249. Karthikeyan, B.; Joe, M.M.; Islam, Md.R.; Sa, T. ACC Deaminase Containing Diazotrophic Endophytic Bacteria Ameliorate Salt Stress in Catharanthus Roseus through Reduced Ethylene Levels and Induction of Antioxidative Defense Systems. *Symbiosis* **2012**, *56*, 77–86, doi:10.1007/s13199-012-0162-6.
250. Karuppiah, V.; Sun, J.; Li, T.; Vallikkannu, M.; Chen, J. Co-Cultivation of Trichoderma Asperellum GDFS1009 and Bacillus Amyloliquefaciens 1841 Causes Differential Gene Expression and Improvement in the Wheat Growth and Biocontrol Activity. *Front. Microbiol.* **2019**, *10*, 1068, doi:10.3389/fmicb.2019.01068.
251. Kasim, W.A.; Osman, M.E.; Omar, M.N.; Abd El-Daim, I.A.; Bejai, S.; Meijer, J. Control of Drought Stress in Wheat Using Plant-Growth-Promoting Bacteria. *J Plant Growth Regul* **2013**, *32*, 122–130, doi:10.1007/s00344-012-9283-7.
252. Kasotia, A.; Varma, A.; Choudhary, D.K. Pseudomonas-Mediated Mitigation of Salt Stress and Growth Promotion in Glycine Max. *Agric Res* **2015**, *4*, 31–41, doi:10.1007/s40003-014-0139-1.
253. Katiyar, P.; Dubey, R.C.; Maheshwari, D.K. ACC Deaminase-Producing Ensifer Adhaerens KS23 Enhances Proximate Nutrient of Pisum Sativum L. Cultivated in High Altitude. *Arch Microbiol* **2021**, *203*, 2689–2698, doi:10.1007/s00203-021-02250-5.
254. Kaur, A.; Devi, S.R.; Vyas, P. Stress-tolerant antagonist plant growth-promoting rizhobacteria from Zea mays. *Journal of Plant Protection Research* **2018**, *58*, 115-123, doi:10.24425/119127.
255. Kaushal, M.; Wani, S.P. Plant-Growth-Promoting Rhizobacteria: Drought Stress Alleviators to Ameliorate Crop Production in Drylands. *Ann Microbiol* **2016**, *66*, 35–42, doi:10.1007/s13213-015-1112-3.
256. Kaushal, M.; Wani, S.P. Rhizobacterial-Plant Interactions: Strategies Ensuring Plant Growth Promotion under Drought and Salinity Stress. *Agriculture, Ecosystems & Environment* **2016**, *231*, 68–78, doi:10.1016/j.agee.2016.06.031.
257. Khalifa, A. ACC Deaminase-Containing Rhizobacteria from Rhizosphere of Zygophyllumcoccineum Alleviate Salt Stress Impact on Wheat (Triticum Aestivum L.). *Sci J King Faisal Univ Basic Appl Sci* **2020**, *21*, 89-102, doi:10.37575/b/agr/1988.
258. Khan, A.; Singh, A.V. Multifarious Effect of ACC Deaminase and EPS Producing Pseudomonas Sp. and Serratia Marcescens to Augment Drought Stress Tolerance and Nutrient Status of Wheat. *World J Microbiol Biotechnol* **2021**, *37*, 198, doi:10.1007/s11274-021-03166-4.
259. Khan, A.; Tan, D.K.Y.; Afridi, M.Z.; Luo, H.; Tung, S.A.; Ajab, M.; Fahad, S. Nitrogen Fertility and Abiotic Stresses Management in Cotton Crop: A Review. *Environ Sci Pollut Res* **2017**, *24*, 14551–14566, doi:10.1007/s11356-017-8920-x.
260. Khan, M.A.; Asaf, S.; Khan, A.L.; Ullah, I.; Ali, S.; Kang, S.-M.; Lee, I.-J. Alleviation of Salt Stress Response in Soybean Plants with the Endophytic Bacterial Isolate Curtobacterium Sp. SAK1. *Ann Microbiol* **2019**, *69*, 797–808, doi:10.1007/s13213-019-01470-x.
261. Khan, M.Y.; Zahir, Z.A.; Asghar, H.N.; Waraich, E.A. PRELIMINARY INVESTIGATIONS ON SELECTION OF SYNERGISTIC HALOTOLERANT PLANT GROWTH PROMOTING RHIZOBACTERIA FOR INDUCING SALINITY TOLERANCE IN WHEAT. *Pak J Bot*, *49*, 1541-1551.
262. Khan, N.; Ali, S.; Shahid, M.A.; Mustafa, A.; Sayyed, R.Z.; Curá, J.A. Insights into the Interactions among Roots, Rhizosphere, and Rhizobacteria for Improving Plant Growth and Tolerance to Abiotic Stresses: A Review. *Cells* **2021**, *10*, 1551, doi:10.3390/cells10061551.
263. Khan, N.; Ali, S.; Tariq, H.; Latif, S.; Yasmin, H.; Mehmood, A.; Shahid, M.A. Water Conservation and Plant Survival Strategies of Rhizobacteria under Drought Stress. *Agronomy* **2020**, *10*, 1683, doi:10.3390/agronomy10111683.
264. Khan, N.; Bano, A.; Ali, S.; Babar, Md.A. Crosstalk amongst Phytohormones from Planta and PGPR under Biotic and Abiotic Stresses. *Plant Growth Regul* **2020**, *90*, 189–203, doi:10.1007/s10725-020-00571-x.
265. Khan, S.; Afzal, M.; Iqbal, S.; Mirza, M.S.; Khan, Q.M. Inoculum Pretreatment Affects Bacterial Survival, Activity and Catabolic Gene Expression during Phytoremediation of Diesel Contaminated Soil. *Chemosphere* **2013**, *91*, 663–668, doi:10.1016/j.chemosphere.2013.01.025.
266. Khanna, K.; Ohri, P.; Bhardwaj, R.; Ahmad, P. Unsnarling Plausible Role of Plant Growth-Promoting Rhizobacteria for Mitigating Cd-Toxicity from Plants: An Environmental Safety Aspect. *J Plant Growth Regul* **2021**, doi:10.1007/s00344-021-10445-9.
267. Khare, E.; Mishra, J.; Arora, N.K. Multifaceted Interactions Between Endophytes and Plant: Developments and Prospects. *Front. Microbiol.* **2018**, *9*, 2732, doi:10.3389/fmicb.2018.02732.
268. Khoshru, B.; Mitra, D.; Khoshmanzar, E.; Myo, E.M.; Uniyal, N.; Mahakur, B.; Mohapatra, P.K.D.; Panneerselvam, P.; Boutaj, H.; Alizadeh, M.; et al. Current Scenario and Future Prospects of Plant Growth-Promoting Rhizobacteria: An Economic Valuable Resource for the Agriculture Revival under Stressful Conditions. *Journal of Plant Nutrition* **2020**, *43*, 3062–3092, doi:10.1080/01904167.2020.1799004.
269. Kim, J.; Woo, O.-G.; Bae, Y.; Keum, H.L.; Chung, S.; Sul, W.J.; Lee, J.-H. Enhanced Drought and Salt Stress Tolerance in *Arabidopsis* by *Flavobacterium Crocinum* HYN0056^T^. *J. Plant Biol.* **2020**, *63*, 63–71, doi:10.1007/s12374-020-09236-8.
270. Kim, K.; Jang, Y.-J.; Lee, S.-M.; Oh, B.-T.; Chae, J.-C.; Lee, K.-J. Alleviation of Salt Stress by *Enterobacter* Sp. EJ01 in Tomato and *Arabidopsis* Is Accompanied by Up-Regulation of Conserved Salinity Responsive Factors in Plants. *Molecules and Cells* **2014**, *37*, 109–117, doi:10.14348/molcells.2014.2239.
271. Kim, K.; Park, S.-H.; Chae, J.-C.; Soh, B.Y.; Lee, K.-J. Rapid Degradation of *Pseudomonas fluorescens* 1-Aminocyclopropane-1-Carboxylic Acid Deaminase Proteins Expressed in Transgenic *Arabidopsis*. *FEMS Microbiol Lett* **2014**, *355*, 193–200, doi:10.1111/1574-6968.12456.
272. Kim, Y.C.; Anderson, A.J. Rhizosphere Pseudomonads as Probiotics Improving Plant Health. *Molecular Plant Pathology* **2018**, *19*, 2349–2359, doi:10.1111/mpp.12693.
273. Klay, I.; Pirrello, J.; Riahi, L.; Bernadac, A.; Cherif, A.; Bouzayen, M.; Bouzid, S. Ethylene Response Factor *Sl-ERF.B.3* Is Responsive to Abiotic Stresses and Mediates Salt and Cold Stress Response Regulation in Tomato. *The Scientific World Journal* **2014**, *2014*, 1–12, doi:10.1155/2014/167681.
274. Kong, Z.; Glick, B.R.; Duan, J.; Ding, S.; Tian, J.; McConkey, B.J.; Wei, G. Effects of 1-Aminocyclopropane-1-Carboxylate (ACC) Deaminase-Overproducing Sinorhizobium Meliloti on Plant Growth and Copper Tolerance of Medicago Lupulina. *Plant Soil* **2015**, *391*, 383–398, doi:10.1007/s11104-015-2434-4.
275. Kothari Vishal, V., & Manuel, V. B. R. Effect of ACC-deaminase producing Bacillus cereus brm on the growth of Vigna radiata (Mung beans) under salinity stress. *Research Journal of Biotechnology Vol* **2015** *10*(11).
276. Kour, D.; Rana, K.L.; Kaur, T.; Sheikh, I.; Yadav, A.N.; Kumar, V.; Dhaliwal, H.S.; Saxena, A.K. Microbe-Mediated Alleviation of Drought Stress and Acquisition of Phosphorus in Great Millet (Sorghum Bicolour L.) by Drought-Adaptive and Phosphorus-Solubilizing Microbes. *Biocatalysis and Agricultural Biotechnology* **2020**, *23*, 101501, doi:10.1016/j.bcab.2020.101501.
277. Kour, D.; Rana, K.L.; Kaur, T.; Yadav, N.; Halder, S.K.; Yadav, A.N.; Sachan, S.G.; Saxena, A.K. Potassium Solubilizing and Mobilizing Microbes: Biodiversity, Mechanisms of Solubilization, and Biotechnological Implication for Alleviations of Abiotic Stress. In *New and Future Developments in Microbial Biotechnology and Bioengineering*; Elsevier, 2020; pp. 177–202 ISBN 978-0-12-820526-6.
278. Kour, D.; Rana, K.L.; Sheikh, I.; Kumar, V.; Yadav, A.N.; Dhaliwal, H.S.; Saxena, A.K. Alleviation of Drought Stress and Plant Growth Promotion by Pseudomonas Libanensis EU-LWNA-33, a Drought-Adaptive Phosphorus-Solubilizing Bacterium. *Proc. Natl. Acad. Sci., India, Sect. B Biol. Sci.* **2020**, *90*, 785–795, doi:10.1007/s40011-019-01151-4.
279. Kour, D.; Rana, K.L.; Yadav, A.N.; Sheikh, I.; Kumar, V.; Dhaliwal, H.S.; Saxena, A.K. Amelioration of Drought Stress in Foxtail Millet (Setaria Italica L.) by P-Solubilizing Drought-Tolerant Microbes with Multifarious Plant Growth Promoting Attributes. *Environmental Sustainability* **2020**, *3*, 23–34, doi:10.1007/s42398-020-00094-1.
280. Kruasuwan, W.; Thamchaipenet, A. 1-Aminocyclopropane-1-Carboxylate (ACC) Deaminase-Producing Endophytic Diazotrophic Enterobacter Sp. EN-21 Modulates Salt–Stress Response in Sugarcane. *J Plant Growth Regul* **2018**, *37*, 849–858, doi:10.1007/s00344-018-9780-4.
281. Kumar Arora, N.; Fatima, T.; Mishra, J.; Mishra, I.; Verma, S.; Verma, R.; Verma, M.; Bhattacharya, A.; Verma, P.; Mishra, P.; et al. Halo-Tolerant Plant Growth Promoting Rhizobacteria for Improving Productivity and Remediation of Saline Soils. *Journal of Advanced Research* **2020**, *26*, 69–82, doi:10.1016/j.jare.2020.07.003.
282. Kumar, A., Meena, V.S., *Plant Growth Promoting Rhizobacteria for Agricultural Sustainability: From Theory to Practices*.Eds.; Springer Singapore: Singapore, 2019; ISBN 9789811375521.
283. Kumar, A.; Patel, J.S.; Meena, V.S.; Srivastava, R. Recent Advances of PGPR Based Approaches for Stress Tolerance in Plants for Sustainable Agriculture. *Biocatalysis and Agricultural Biotechnology* **2019**, *20*, 101271, doi:10.1016/j.bcab.2019.101271.
284. Kumar, A.; Singh, S.; Gaurav, A.K.; Srivastava, S.; Verma, J.P. Plant Growth-Promoting Bacteria: Biological Tools for the Mitigation of Salinity Stress in Plants. *Front. Microbiol.* **2020**, *11*, 1216, doi:10.3389/fmicb.2020.01216.
285. Kumar, A.; Singh, S.; Mukherjee, A.; Rastogi, R.P.; Verma, J.P. Salt-Tolerant Plant Growth-Promoting Bacillus Pumilus Strain JPVS11 to Enhance Plant Growth Attributes of Rice and Improve Soil Health under Salinity Stress. *Microbiological Research* **2021**, *242*, 126616, doi:10.1016/j.micres.2020.126616.
286. Kumar, A.; Tripti; Maleva, M.; Bruno, L.B.; Rajkumar, M. Synergistic Effect of ACC Deaminase Producing Pseudomonas Sp. TR15a and Siderophore Producing Bacillus Aerophilus TR15c for Enhanced Growth and Copper Accumulation in Helianthus Annuus L. *Chemosphere* **2021**, *276*, 130038, doi:10.1016/j.chemosphere.2021.130038.
287. Kumar, A.; Tripti; Voropaeva, O.; Maleva, M.; Panikovskaya, K.; Borisova, G.; Rajkumar, M.; Bruno, L.B. Bioaugmentation with Copper Tolerant Endophyte Pseudomonas Lurida Strain EOO26 for Improved Plant Growth and Copper Phytoremediation by Helianthus Annuus. *Chemosphere* **2021**, *266*, 128983, doi:10.1016/j.chemosphere.2020.128983.
288. Kumar, M., Kumar, V., Prasad, R. *Phyto-Microbiome in Stress Regulation*; Eds.; Environmental and Microbial Biotechnology; Springer Singapore: Singapore, 2020; ISBN 9789811525759.
289. Kumar, M.; Mishra, S.; Dixit, V.; Kumar, M.; Agarwal, L.; Chauhan, P.S.; Nautiyal, C.S. Synergistic Effect of *Pseudomonas Putida* and *Bacillus Amyloliquefaciens* Ameliorates Drought Stress in Chickpea ( *Cicer Arietinum* L.). *Plant Signaling & Behavior* **2016**, *11*, e1071004, doi:10.1080/15592324.2015.1071004.
290. Kumar, P.A.; Sujatha, R.V.; Vennela, K. The Role of Rhizosphere Microorganisms Containing the Abiotic Stress Tolerance in Plants. *Chem Sci Rev Lett* **2018**, *7*, 214-221.
291. Kumar, S.; Kumar, M.; Wakchaure, G.C.; Bhushan, B.; Meena, K.K.; Singh, A.K.; Gurumurthy, S.; Rane, J. Microbial Management of Crop Abiotic Stresses: Current Trends and Prospects. In *Microbial Management of Plant Stresses*; Elsevier, 2021; pp. 251–260 ISBN 978-0-323-85193-0.
292. Kumar, S.A.; Sridar, R.; Sivakumar, U. Mitigation of Drought in Rice by a Phyllosphere Bacterium Bacillus Altitudinis FD48. *Afr. J. Microbiol. Res.* **2017**, *11*, 1614–1625, doi:10.5897/AJMR2017.8610.
293. Kumar, V., Kumar, M., Sharma, S., Prasad, R. *Probiotics and Plant Health*; Eds.; Springer Singapore: Singapore, 2017; ISBN 978-981-10-3472-5.
294. Kumar, V., Prasad, R., Kumar, M., Choudhary, D.K. *Microbiome in Plant Health and Disease: Challenges and Opportunities*; Eds.; Springer Singapore: Singapore, 2019; ISBN 9789811384943.
295. Kumari, P.; Khanna, V. ACC-Deaminase and EPS Production by Salt Tolerant Rhizobacteria Augment Growth in Chickpea under Salinity Stress. *Inter. Jour. of Bio-reso. Stress Manag.* **2015**, *6*, 558, doi:10.5958/0976-4038.2015.00084.6.
296. Kumari, S.; Vaishnav, A.; Jain, S.; Choudhary, D.K.; Sharma, K.P. In Vitro Screening for Salinity and Drought Stress Tolerance in Plant Growth Promoting Bacterial Strains. *International Journal of Agricultural and Life Sciences* **2016**, *2*, 7.
297. Kumari, S.; Vaishnav, A.; Jain, S.; Kumar Choudhary, D.; Sharma, K.P. Regulation of Ethylene Level in Mungbean (Vigna Radiata L.) by 1-Aminocyclopropane-1-Carboxylic Acid (ACC)-Deaminase Containing Bacterial Strain under Salt Stress. *Int.J.Curr.Microbiol.App.Sci* **2016**, *5*, 275–283, doi:10.20546/ijcmas.2016.511.030.
298. Kumari, S.; Vaishnav, A.; Jain, S.; Varma, A.; Choudhary, D.K. Bacterial-Mediated Induction of Systemic Tolerance to Salinity with Expression of Stress Alleviating Enzymes in Soybean (*Glycine Max* L. Merrill). *J Plant Growth Regul* **2015**, *34*, 558–573, doi:10.1007/s00344-015-9490-0.
299. Kumari, S.; Vaishnav, A.; Jain, S.; Varma, A.; Choudhary, D.K. Induced Drought Tolerance through Wild and Mutant Bacterial Strain *Pseudomonas* Simiae in Mung Bean (*Vigna Radiata* L.). *World J Microbiol Biotechnol* **2016**, *32*, 4, doi:10.1007/s11274-015-1974-3.
300. Kumawat, K. C., Sharma, P., Nagpal, S., Gupta, R. K., Sirari, A., Nair, R. M., ... & Singh, S. Dual microbial inoculation, a game changer?–bacterial biostimulants with multifunctional growth promoting traits to mitigate salinity stress in spring mungbean. *Frontiers in microbiology* **2021**, *11*, 600576.
301. Kurepin, L.V.; Ozga, J.A.; Zaman, M.; Pharis, R.P. The Physiology of Plant Hormones in Cereal, Oilseed and Pulse Crops. **2013**, *6*, 17.
302. Kurepin, L.V.; Zaman, M.; Pharis, R.P. Phytohormonal Basis for the Plant Growth Promoting Action of Naturally Occurring Biostimulators: Basis for the Action of Biostimulators. *J. Sci. Food Agric.* **2014**, *94*, 1715–1722, doi:10.1002/jsfa.6545.
303. Ledger, T.; Rojas, S.; Timmermann, T.; Pinedo, I.; Poupin, M.J.; Garrido, T.; Richter, P.; Tamayo, J.; Donoso, R. Volatile-Mediated Effects Predominate in Paraburkholderia Phytofirmans Growth Promotion and Salt Stress Tolerance of *Arabidopsis thaliana*. *Front. Microbiol.* **2016**, *7*, 1838, doi:10.3389/fmicb.2016.01838.
304. Leontidou, K.; Genitsaris, S.; Papadopoulou, A.; Kamou, N.; Bosmali, I.; Matsi, T.; Madesis, P.; Vokou, D.; Karamanoli, K.; Mellidou, I. Plant Growth Promoting Rhizobacteria Isolated from Halophytes and Drought-Tolerant Plants: Genomic Characterisation and Exploration of Phyto-Beneficial Traits. *Sci Rep* **2020**, *10*, 14857, doi:10.1038/s41598-020-71652-0.
305. Lephatsi, M.M.; Meyer, V.; Piater, L.A.; Dubery, I.A.; Tugizimana, F. Plant Responses to Abiotic Stresses and Rhizobacterial Biostimulants: Metabolomics and Epigenetics Perspectives. *Metabolites* **2021**, *11*, 457, doi:10.3390/metabo11070457.
306. Li, B.; Sang, T.; He, L.; Sun, J.; Li, J.; Guo, S. Exogenous Spermidine Inhibits Ethylene Production in Leaves of Cucumber Seedlings under NaCl Stress. *J. Amer. Soc. Hort. Sci.* **2013**, *138*, 108–113, doi:10.21273/JASHS.138.2.108.
307. Li, J.; McConkey, B.J.; Cheng, Z.; Guo, S.; Glick, B.R. Identification of Plant Growth-Promoting Bacteria-Responsive Proteins in Cucumber Roots under Hypoxic Stress Using a Proteomic Approach. *Journal of Proteomics* **2013**, *84*, 119–131, doi:10.1016/j.jprot.2013.03.011.
308. Li, X.; Sun, P.; Zhang, Y.; Jin, C.; Guan, C. A Novel PGPR Strain Kocuria Rhizophila Y1 Enhances Salt Stress Tolerance in Maize by Regulating Phytohormone Levels, Nutrient Acquisition, Redox Potential, Ion Homeostasis, Photosynthetic Capacity and Stress-Responsive Genes Expression. *Environmental and Experimental Botany* **2020**, *174*, 104023, doi:10.1016/j.envexpbot.2020.104023.
309. Li, Z.; Chang, S.; Ye, S.; Chen, M.; Lin, L.; Li, Y.; Li, S.; An, Q. Differentiation of 1-Aminocyclopropane-1-Carboxylate (ACC) Deaminase from Its Homologs Is the Key for Identifying Bacteria Containing ACC Deaminase. *FEMS Microbiology Ecology* **2015**, *91*, fiv112, doi:10.1093/femsec/fiv112.
310. Liu, C.-H.; Siew, W.; Hung, Y.-T.; Jiang, Y.-T.; Huang, C.-H. 1-Aminocyclopropane-1-Carboxylate (ACC) Deaminase Gene in *Pseudomonas Azotoformans* Is Associated with the Amelioration of Salinity Stress in Tomato. *J. Agric. Food Chem.* **2021**, *69*, 913–921, doi:10.1021/acs.jafc.0c05628.
311. Liu, H.; Carvalhais, L.C.; Crawford, M.; Singh, E.; Dennis, P.G.; Pieterse, C.M.J.; Schenk, P.M. Inner Plant Values: Diversity, Colonization and Benefits from Endophytic Bacteria. *Front. Microbiol.* **2017**, *8*, 2552, doi:10.3389/fmicb.2017.02552.
312. Liu, H.; Khan, M.Y.; Carvalhais, L.C.; Delgado-Baquerizo, M.; Yan, L.; Crawford, M.; Dennis, P.G.; Singh, B.; Schenk, P.M. Soil Amendments with Ethylene Precursor Alleviate Negative Impacts of Salinity on Soil Microbial Properties and Productivity. *Sci Rep* **2019**, *9*, 6892, doi:10.1038/s41598-019-43305-4.
313. Liu, Y.; Cao, L.; Tan, H.; Zhang, R. Surface Display of ACC Deaminase on Endophytic Enterobacteriaceae Strains to Increase Saline Resistance of Host Rice Sprouts by Regulating Plant Ethylene Synthesis. *Microb Cell Fact* **2017**, *16*, 214, doi:10.1186/s12934-017-0831-5.
314. Lo, K.-J.; Lin, S.-S.; Lu, C.-W.; Kuo, C.-H.; Liu, C.-T. Whole-Genome Sequencing and Comparative Analysis of Two Plant-Associated Strains of Rhodopseudomonas Palustris (PS3 and YSC3). *Sci Rep* **2018**, *8*, 12769, doi:10.1038/s41598-018-31128-8.
315. Loganathan, P.; Myung, H.; Muthusamy, G.; Lee, K.-J.; Seralathan, K.-K.; Oh, B.-T. Effect of Heavy Metals on *AcdS* Gene Expression in *Herbaspirillium* Sp. GW103 Isolated from Rhizosphere Soil: Effect of Heavy Metals on *AcdS* Gene Expression. *J. Basic Microbiol.* **2015**, *55*, 1232–1238, doi:10.1002/jobm.201500008.
316. Lopes, M.J. dos S.; Dias-Filho, M.B.; Gurgel, E.S.C. Successful Plant Growth-Promoting Microbes: Inoculation Methods and Abiotic Factors. *Front. Sustain. Food Syst.* **2021**, *5*, 606454, doi:10.3389/fsufs.2021.606454.
317. Lu, H.; Wei, T.; Lou, H.; Shu, X.; Chen, Q. A Critical Review on Communication Mechanism within Plant-Endophytic Fungi Interactions to Cope with Biotic and Abiotic Stresses. *JoF* **2021**, *7*, 719, doi:10.3390/jof7090719.
318. Lucas, J.A.; García-Cristobal, J.; Bonilla, A.; Ramos, B.; Gutierrez-Mañero, J. Beneficial Rhizobacteria from Rice Rhizosphere Confers High Protection against Biotic and Abiotic Stress Inducing Systemic Resistance in Rice Seedlings. *Plant Physiology and Biochemistry* **2014**, *82*, 44–53, doi:10.1016/j.plaphy.2014.05.007.
319. Lyngwi, N.A.; Nongkhlaw, M.; Kalita, D.; Joshi, S.R. Bioprospecting of Plant Growth Promoting Bacilli and Related Genera Prevalent in Soils of Pristine Sacred Groves: Biochemical and Molecular Approach. *PLoS ONE* **2016**, *11*, e0152951, doi:10.1371/journal.pone.0152951.
320. Ma, Y.; Dias, M.C.; Freitas, H. Drought and Salinity Stress Responses and Microbe-Induced Tolerance in Plants. *Front. Plant Sci.* **2020**, *11*, 591911, doi:10.3389/fpls.2020.591911.
321. Ma, Y.; Rajkumar, M.; Moreno, A.; Zhang, C.; Freitas, H. Serpentine Endophytic Bacterium Pseudomonas Azotoformans ASS1 Accelerates Phytoremediation of Soil Metals under Drought Stress. *Chemosphere* **2017**, *185*, 75–85, doi:10.1016/j.chemosphere.2017.06.135.
322. Magnucka, E.G.; Pietr, S.J. Various Effects of Fluorescent Bacteria of the Genus Pseudomonas Containing ACC Deaminase on Wheat Seedling Growth. *Microbiological Research* **2015**, *181*, 112–119, doi:10.1016/j.micres.2015.04.005.
323. Mahdi Dar, Z.; Masood, A.; Hussain Mughal, A.; Asif, M.; Ahamd Malik, M. Review on Drought Tolerance in Plants Induced by Plant Growth Promoting Rhizobacteria. *Int.J.Curr.Microbiol.App.Sci* **2018**, *7*, 412–422, doi:10.20546/ijcmas.2018.705.053.
324. Maheshwari, R.; Bhutani, N.; Suneja, P. Isolation and Characterization of ACC Deaminase Producing Endophytic Bacillus Mojavensis PRN2 from Pisum Sativum. *IRAN J BIOTCH* **2020**, *18*, doi:10.30498/ijb.2020.137279.2308.
325. Mahmud, K.K.; Khudhur, A.M. Effects Of The Transconjugant Pseudomonas Fluorescens Carrying 1-Aminocyclopropane-1-Carboxylate Deaminase Gene On Drought Tolerance Of Wheat. *Nat. Volatiles & Essent. Oils* **2022**, *9*, 1227-1242, .
326. Maqbool, S.; Amna, A.; Maqbool, A.; Mehmood, S.; Suhaib, M.; Sultan, T.; Munis, M.F.H.; Rehman, S.-U.; Chaudhary, H.J. Interaction of ACC Deaminase and Antioxidant Enzymes to Induce Drought Tolerance in Enterobacter Cloacae 2WC2 Inoculated Maize Genotypes. *PAK.J.BOT.* **2021**, *53*, 893-903, doi:10.30848/PJB2021-3(28).
327. Maqsood, A.; Shahid, M.; Hussain, S.; Mahmood, F.; Azeem, F.; Tahir, M.; Ahmed, T.; Noman, M.; Manzoor, I.; Basit, F. Root Colonizing *Burkholderia* Sp. AQ12 Enhanced Rice Growth and Upregulated Tillering-Responsive Genes in Rice. *Applied Soil Ecology* **2021**, *157*, 103769, doi:10.1016/j.apsoil.2020.103769.
328. Mathur, P.; Roy, S. Insights into the Plant Responses to Drought and Decoding the Potential of Root Associated Microbiome for Inducing Drought Tolerance. *Physiologia Plantarum* **2021**, *172*, 1016–1029, doi:10.1111/ppl.13338.
329. Matsuoka, H.; Ohwaki, Y.; Terakado-Tonooka, J.; Tanaka, F. Changes in Volatiles in Carrots Inoculated with ACC Deaminase-Producing Bacteria Isolated from Organic Crops. *Plant Soil* **2016**, *407*, 173–186, doi:10.1007/s11104-015-2769-x.
330. Maxton, A.; Singh, P.; Masih, S.A. ACC Deaminase-Producing Bacteria Mediated Drought and Salt Tolerance in *Capsicum Annuum*. *Journal of Plant Nutrition* **2018**, *41*, 574–583, doi:10.1080/01904167.2017.1392574.
331. Maxton, A.; Singh, P.; Prasad, S.M.; Andy, A.; Masih, S.A. Characterization of ACC Deaminase Producing B. Cepacia, C. Feurendii and S. Marcescens for Plant Growth Promoting Activity. *Int.J.Curr.Microbiol.App.Sci* **2017**, *6*, 883–897, doi:10.20546/ijcmas.2017.608.111.
332. Mayak, S.; Tirosh, T.; Glick, B.R. Plant Growth-Promoting Bacteria Confer Resistance in Tomato Plants to Salt Stress. *Plant Physiology and Biochemistry* **2004**, *42*, 565–572, doi:10.1016/j.plaphy.2004.05.009.
333. McMillan, M.; Kallenbach, C.M.; Whalen, J.K. Soybean Abiotic Stress Tolerance Is Improved by Beneficial Rhizobacteria in Biosolids-Amended Soil. *Applied Soil Ecology* **2022**, *174*, 104425, doi:10.1016/j.apsoil.2022.104425.
334. Mehmood, S.; Khan, A.A.; Shi, F.; Tahir, M.; Sultan, T.; Munis, M.F.H.; Kaushik, P.; Alyemeni, M.N.; Chaudhary, H.J. Alleviation of Salt Stress in Wheat Seedlings via Multifunctional *Bacillus Aryabhattai* PM34: An *In-Vitro* Study. *Sustainability* **2021**, *13*, 8030, doi:10.3390/su13148030.
335. Mehmood, S.; Muneer, M.A.; Tahir, M.; Javed, M.T.; Mahmood, T.; Afridi, M.S.; Pakar, N.P.; Abbasi, H.A.; Munis, M.F.H.; Chaudhary, H.J. Deciphering Distinct Biological Control and Growth Promoting Potential of Multi-Stress Tolerant Bacillus Subtilis PM32 for Potato Stem Canker. *Physiol Mol Biol Plants* **2021**, *27*, 2101–2114, doi:10.1007/s12298-021-01067-2.
336. Mellidou, I.; Ainalidou, A.; Papadopoulou, A.; Leontidou, K.; Genitsaris, S.; Karagiannis, E.; Van de Poel, B.; Karamanoli, K. Comparative Transcriptomics and Metabolomics Reveal an Intricate Priming Mechanism Involved in PGPR-Mediated Salt Tolerance in Tomato. *Front. Plant Sci.* **2021**, *12*, 713984, doi:10.3389/fpls.2021.713984.
337. *Microbial Metatranscriptomics Belowground*; Nath, M., Bhatt, D., Bhargava, P., Choudhary, D.K., Eds.; Springer Singapore: Singapore, 2021; ISBN 9789811597572.
338. Miliute, I.; Buzaite, O.; Baniulis, D.; Stanys, V. Bacterial Endophytes in Agricultural Crops and Their Role in Stress Tolerance: A Review. *Zemdirbyste-Agriculture* **2015**, *102*, 465–478, doi:10.13080/z-a.2015.102.060.
339. Mishra, P.; Mishra, J.; Arora, N.K. Plant Growth Promoting Bacteria for Combating Salinity Stress in Plants – Recent Developments and Prospects: A Review. *Microbiological Research* **2021**, *252*, 126861, doi:10.1016/j.micres.2021.126861.
340. Mishra, S.K.; Khan, M.H.; Misra, S.; Dixit, V.K.; Khare, P.; Srivastava, S.; Chauhan, P.S. Characterisation of Pseudomonas Spp. and Ochrobactrum Sp. Isolated from Volcanic Soil. *Antonie van Leeuwenhoek* **2017**, *110*, 253–270, doi:10.1007/s10482-016-0796-0.
341. Misra, S.; Chauhan, P.S. ACC Deaminase-Producing Rhizosphere Competent Bacillus Spp. Mitigate Salt Stress and Promote Zea Mays Growth by Modulating Ethylene Metabolism. *3 Biotech* **2020**, *10*, 119, doi:10.1007/s13205-020-2104-y.
342. Misra, S.; Dixit, V.K.; Khan, M.H.; Kumar Mishra, S.; Dviwedi, G.; Yadav, S.; Lehri, A.; Singh Chauhan, P. Exploitation of Agro-Climatic Environment for Selection of 1-Aminocyclopropane-1-Carboxylic Acid (ACC) Deaminase Producing Salt Tolerant Indigenous Plant Growth Promoting Rhizobacteria. *Microbiological Research* **2017**, *205*, 25–34, doi:10.1016/j.micres.2017.08.007.
343. Misra, S.; Dixit, V.K.; Mishra, S.K.; Chauhan, P.S. Demonstrating the Potential of Abiotic Stress-Tolerant Jeotgalicoccus Huakuii NBRI 13E for Plant Growth Promotion and Salt Stress Amelioration. *Ann Microbiol* **2019**, *69*, 419–434, doi:10.1007/s13213-018-1428-x.
344. Mitra, D.; Díaz Rodríguez, A.M.; Parra Cota, F.I.; Khoshru, B.; Panneerselvam, P.; Moradi, S.; Sagarika, M.S.; Anđelković, S.; Santos-Villalobos, S. de los; Das Mohapatra, P.K. Amelioration of Thermal Stress in Crops by Plant Growth-Promoting Rhizobacteria. *Physiological and Molecular Plant Pathology* **2021**, *115*, 101679, doi:10.1016/j.pmpp.2021.101679.
345. Molina, R.; Rivera, D.; Mora, V.; López, G.; Rosas, S.; Spaepen, S.; Vanderleyden, J.; Cassán, F. Regulation of IAA Biosynthesis in Azospirillum Brasilense Under Environmental Stress Conditions. *Curr Microbiol* **2018**, *75*, 1408–1418, doi:10.1007/s00284-018-1537-6.
346. Mommer, L.; Hinsinger, P.; Prigent-Combaret, C.; Visser, E.J.W. Advances in the Rhizosphere: Stretching the Interface of Life. *Plant Soil* **2016**, *407*, 1–8, doi:10.1007/s11104-016-3040-9.
347. Monteiro, P.H.R.; da Silva, F.B.; de Abreu, C.M.; da Silva, G.J. Plant Growth Promoting Rhizobacteria in Amelioration of Abiotic Stresses: A Functional Interplay and Prospective. In *Plant Growth-Promoting Microbes for Sustainable Biotic and Abiotic Stress Management*; Mohamed, H.I., El-Beltagi, H.E.-D.S., Abd-Elsalam, K.A., Eds.; Springer International Publishing: Cham, 2021; pp. 25–49 ISBN 978-3-030-66586-9.
348. Montes-Osuna, N., Gómez-Lama Cabanás, C., Valverde-Corredor, A., Legarda, G., Prieto, P., & Mercado-Blanco, J. Evaluation of indigenous olive biocontrol rhizobacteria as protectants against drought and salt stress. *Microorganisms* **2021**, *9*(6), 1209.
349. Moon, Y.-S.; Ali, S. A Fruitful Decade of Bacterial ACC Deaminase Biotechnology: A Pragmatic Approach towards Abiotic Stress Relief in Plants. *Theor. Exp. Plant Physiol.* **2022**, doi:10.1007/s40626-022-00237-1
350. Moon, Y.-S.; Ali, S. Isolation and Identification of Multi-Trait Plant Growth–Promoting Rhizobacteria from Coastal Sand Dune Plant Species of Pohang Beach. *Folia Microbiol* **2022**, *67*, 523–533, doi:10.1007/s12223-022-00959-4.
351. Morcillo, R.; Manzanera, M. The Effects of Plant-Associated Bacterial Exopolysaccharides on Plant Abiotic Stress Tolerance. *Metabolites* **2021**, *11*, 337, doi:10.3390/metabo11060337.
352. Mukhtar, T.; Ali, F.; Rafique, M.; Ali, J.; Afridi, M.S.; Smith, D.; Mehmood, S.; Amna; Souleimanov, A.; Jellani, G.; et al. Biochemical Characterization and Potential of Bacillus Safensis Strain SCAL1 to Mitigate Heat Stress in Solanum Lycopersicum L. *J Plant Growth Regul* **2022**, 1-6, doi:10.1007/s00344-021-10571-4.
353. Mukhtar, T.; Rehman, S. ur; Smith, D.; Sultan, T.; Seleiman, M.F.; Alsadon, A.A.; Amna; Ali, S.; Chaudhary, H.J.; Solieman, T.H.I.; et al. Mitigation of Heat Stress in Solanum Lycopersicum L. by ACC-Deaminase and Exopolysaccharide Producing Bacillus Cereus: Effects on Biochemical Profiling. *Sustainability* **2020**, *12*, 2159, doi:10.3390/su12062159.
354. Murali, M.; Gowtham, H.G.; Singh, S.B.; Shilpa, N.; Aiyaz, M.; Niranjana, S.R.; Amruthesh, K.N. Bio-Prospecting of ACC Deaminase Producing Rhizobacteria towards Sustainable Agriculture: A Special Emphasis on Abiotic Stress in Plants. *Applied Soil Ecology* **2021**, *168*, 104142, doi:10.1016/j.apsoil.2021.104142.
355. Murali, M.; Singh, S.B.; Gowtham, H.G.; Shilpa, N.; Prasad, M.; Aiyaz, M.; Amruthesh, K.N. Induction of Drought Tolerance in Pennisetum Glaucum by ACC Deaminase Producing PGPR- Bacillus Amyloliquefaciens through Antioxidant Defense System. *Microbiological Research* **2021**, *253*, 126891, doi:10.1016/j.micres.2021.126891.
356. Murset, V.; Hennecke, H.; Pessi, G. Disparate Role of Rhizobial ACC Deaminase in Root-Nodule Symbioses. *Symbiosis* **2012**, *57*, 43–50, doi:10.1007/s13199-012-0177-z.
357. Nadarajah, K.K. ROS Homeostasis in Abiotic Stress Tolerance in Plants. *IJMS* **2020**, *21*, 5208, doi:10.3390/ijms21155208.
358. Nadeem, S.M.; Ahmad, M.; Naveed, M.; Imran, M.; Zahir, Z.A.; Crowley, D.E. Relationship between in Vitro Characterization and Comparative Efficacy of Plant Growth-Promoting Rhizobacteria for Improving Cucumber Salt Tolerance. *Arch Microbiol* **2016**, *198*, 379–387, doi:10.1007/s00203-016-1197-5.
359. Nadeem, S.M.; Ahmad, M.; Tufail, M.A.; Asghar, H.N.; Nazli, F.; Zahir, Z.A. Appraising the Potential of EPS ‐producing Rhizobacteria with ACC ‐deaminase Activity to Improve Growth and Physiology of Maize under Drought Stress. *Physiologia Plantarum* **2021**, *172*, 463–476, doi:10.1111/ppl.13212.
360. Naing, A.H.; Campol, J.R.; Kang, H.; Xu, J.; Chung, M.Y.; Kim, C.K. Role of Ethylene Biosynthesis Genes in the Regulation of Salt Stress and Drought Stress Tolerance in Petunia. *Front. Plant Sci.* **2022**, *13*, 844449, doi:10.3389/fpls.2022.844449.
361. Naing, A.H.; Jeong, H.Y.; Jung, S.K.; Kim, C.K. Overexpression of 1-Aminocyclopropane-1-Carboxylic Acid Deaminase (*acdS*) Gene in *Petunia hybrida* Improves Tolerance to Abiotic Stresses. *Front. Plant Sci.* **2021**, *12*, 737490, doi:10.3389/fpls.2021.737490.
362. Naing, A.H.; Maung, T.; Kim, C.K. The ACC Deaminase‐producing Plant Growth‐promoting Bacteria: Influences of Bacterial Strains and ACC Deaminase Activities in Plant Tolerance to Abiotic Stress. *Physiologia Plantarum* **2021**, *173*, 1992–2012, doi:10.1111/ppl.13545.
363. Nascimento, F.; Brígido, C.; Alho, L.; Glick, B.R.; Oliveira, S. Enhanced Chickpea Growth-Promotion Ability of a *Mesorhizobium* Strain Expressing an Exogenous ACC Deaminase Gene. *Plant Soil* **2011**, *353*, 221–230, doi:10.1007/s11104-011-1025-2.
364. Nascimento, F.; Brìgido, C; Alho, L.; Glick, B.R.; Oliveira, S. Enhanced chicjpea Growth-Promotion Ability of a Mesorhizobium Strain Expressing an Exogenous ACC Deaminase Gene. *Plant Soil* **2012**, *353*, 221–230, doi:10.1007/s11104-011-1025-2.
365. Nascimento, F.X.; Brígido, C.; Glick, B.R.; Oliveira, S.; Alho, L. Mesorhizobium Ciceri LMS-1 Expressing an Exogenous 1-Aminocyclopropane-1-Carboxylate (ACC) Deaminase Increases Its Nodulation Abilities and Chickpea Plant Resistance to Soil Constraints: M. Ciceri (PRKACC) Increases Nodulation and Plant Growth. *Letters in Applied Microbiology* **2012**, *55*, 15–21, doi:10.1111/j.1472-765X.2012.03251.x.
366. Nascimento, F.X.; Rossi, M.J.; Soares, C.R.F.S.; McConkey, B.J.; Glick, B.R. New Insights into 1-Aminocyclopropane-1-Carboxylate (ACC) Deaminase Phylogeny, Evolution and Ecological Significance. *PLoS ONE* **2014**, *9*, e99168, doi:10.1371/journal.pone.0099168.
367. Nascimento, F.X.; Tavares, M.J.; Rossi, M.J.; Glick, B.R. The Modulation of Leguminous Plant Ethylene Levels by Symbiotic Rhizobia Played a Role in the Evolution of the Nodulation Process. *Heliyon* **2018**, *4*, e01068, doi:10.1016/j.heliyon.2018.e01068.
368. Nascimento, F.X.; Urón, P.; Glick, B.R.; Giachini, A.; Rossi, M.J. Genomic Analysis of the 1-Aminocyclopropane-1-Carboxylate Deaminase-Producing *Pseudomonas* *thivervalensis* SC5 Reveals Its Multifaceted Roles in Soil and in Beneficial Interactions With Plants. *Front. Microbiol.* **2021**, *12*, 752288, doi:10.3389/fmicb.2021.752288.
369. Nath Yadav, A. Plant Growth Promoting Bacteria: Biodiversity and Multifunctional Attributes for Sustainable Agriculture. *AIBM* **2017**, *5*, doi:10.19080/AIBM.2017.05.555671.
370. Nautiyal, C.S.; Srivastava, S.; Chauhan, P.S.; Seem, K.; Mishra, A.; Sopory, S.K. Plant Growth-Promoting Bacteria Bacillus Amyloliquefaciens NBRISN13 Modulates Gene Expression Profile of Leaf and Rhizosphere Community in Rice during Salt Stress. *Plant Physiology and Biochemistry* **2013**, *66*, 1–9, doi:10.1016/j.plaphy.2013.01.020.
371. Negacz, K.; Vellinga, P.; Barrett-Lennard, E.; Choukr-Allah, R.; Elzenga, T. *Future of Sustainable Agriculture in Saline Environments*; Negacz, K., Vellinga, P., Barrett-Lennard, E., Choukr-Allah, R., Elzenga, T., Eds.; 1st ed.; CRC Press, 2021; ISBN 978-1-00-311232-7.
372. Nikolic, B.; Schwab, H.; Sessitsch, A. Metagenomic Analysis of the 1-Aminocyclopropane-1-Carboxylate Deaminase Gene (AcdS) Operon of an Uncultured Bacterial Endophyte Colonizing Solanum Tuberosum L. *Arch Microbiol* **2011**, *193*, 665–676, doi:10.1007/s00203-011-0703-z.
373. Niu, X.; Song, L.; Xiao, Y.; Ge, W. Drought-Tolerant Plant Growth-Promoting Rhizobacteria Associated with Foxtail Millet in a Semi-Arid Agroecosystem and Their Potential in Alleviating Drought Stress. *Front. Microbiol.* **2018**, *8*, 2580, doi:10.3389/fmicb.2017.02580.
374. Nonaka, S.; Ezura, H. Agrobacterium Interaction Mediated by Ethylene and Super-Agrobacterium Conferring Efficient Gene Transfer. *Front. Plant Sci.* **2014**, *5*, 681, doi:10.3389/fpls.2014.00681.
375. Notununu, I.; Moleleki, L.; Roopnarain, A.; Adeleke, R. Effects of Plant Growth-Promoting Rhizobacteria on the Molecular Responses of Maize under Drought and Heat Stresses: A Review. *Pedosphere* **2022**, *32*, 90–106, doi:10.1016/S1002-0160(21)60051-6.
376. Nozari, R.M.; Ortolan, F.; Astarita, L.V.; Santarém, E.R. Streptomyces Spp. Enhance Vegetative Growth of Maize Plants under Saline Stress. *Braz J Microbiol* **2021**, *52*, 1371–1383, doi:10.1007/s42770-021-00480-9.
377. Ogbe, A.A.; Finnie, J.F.; Van Staden, J. The Role of Endophytes in Secondary Metabolites Accumulation in Medicinal Plants under Abiotic Stress. *South African Journal of Botany* **2020**, *134*, 126–134, doi:10.1016/j.sajb.2020.06.023.
378. Ojuederie, O.; Olanrewaju, O.; Babalola, O. Plant Growth Promoting Rhizobacterial Mitigation of Drought Stress in Crop Plants: Implications for Sustainable Agriculture. *Agronomy* **2019**, *9*, 712, doi:10.3390/agronomy9110712.
379. Oleńska, E.; Małek, W.; Wójcik, M.; Swiecicka, I.; Thijs, S.; Vangronsveld, J. Beneficial Features of Plant Growth-Promoting Rhizobacteria for Improving Plant Growth and Health in Challenging Conditions: A Methodical Review. *Science of The Total Environment* **2020**, *743*, 140682, doi:10.1016/j.scitotenv.2020.140682.
380. Omer, A.M.; Emara, H.M.; Zaghloul, R.A.; Abdel-Monem, M.O.; Dawwam, G.E. Effect of fungal inoculation and different levels of chemical fertilization on wheat growth and production( Triticum aestivum, L.). *Eco. Env. & Cons.* **2016**, *26*, 676-682.
381. Orozco-Mosqueda, Ma. del C.; Duan, J.; DiBernardo, M.; Zetter, E.; Campos-García, J.; Glick, B.R.; Santoyo, G. The Production of ACC Deaminase and Trehalose by the Plant Growth Promoting Bacterium Pseudomonas Sp. UW4 Synergistically Protect Tomato Plants Against Salt Stress. *Front. Microbiol.* **2019**, *10*, 1392, doi:10.3389/fmicb.2019.01392.
382. Ouzhand, B., Maleki, M., Soltani, M., & Shakeri, S. (2016). Molecular identification of the Iranian native ACC deaminase producing rhizobacteria using 16S rDNA sequence analysis.
383. Ozimek, E.; Jaroszuk-Ściseł, J.; Bohacz, J.; Korniłłowicz-Kowalska, T.; Tyśkiewicz, R.; Słomka, A.; Nowak, A.; Hanaka, A. Synthesis of Indoleacetic Acid, Gibberellic Acid and ACC-Deaminase by Mortierella Strains Promote Winter Wheat Seedlings Growth under Different Conditions. *IJMS* **2018**, *19*, 3218, doi:10.3390/ijms19103218.
384. Paço, A.; da-Silva, J.R.; Torres, D.P.; Glick, B.R.; Brígido, C. Exogenous ACC Deaminase Is Key to Improving the Performance of Pasture Legume-Rhizobial Symbioses in the Presence of a High Manganese Concentration. *Plants* **2020**, *9*, 1630, doi:10.3390/plants9121630.
385. Palacio-Rodríguez, R.; Coria-Arellano, J.L.; López-Bucio, J.; Sánchez-Salas, J.; Muro-Pérez, G.; Castañeda-Gaytán, G.; Sáenz-Mata, J. Halophilic Rhizobacteria from *Distichlis Spicata* Promote Growth and Improve Salt Tolerance in Heterologous Plant Hosts. *Symbiosis* **2017**, *73*, 179–189, doi:10.1007/s13199-017-0481-8.
386. Palaniyandi, S.A.; Damodharan, K.; Yang, S.H.; Suh, J.W. *Streptomyces* Sp. Strain PGPA39 Alleviates Salt Stress and Promotes Growth of ‘Micro Tom’ Tomato Plants. *J Appl Microbiol* **2014**, *117*, 766–773, doi:10.1111/jam.12563.
387. Palaniyandi, S.A.; Yang, S.H.; Zhang, L.; Suh, J.-W. Effects of Actinobacteria on Plant Disease Suppression and Growth Promotion. *Appl Microbiol Biotechnol* **2013**, *97*, 9621–9636, doi:10.1007/s00253-013-5206-1.
388. Parray, J.A.; Jan, S.; Kamili, A.N.; Qadri, R.A.; Egamberdieva, D.; Ahmad, P. Current Perspectives on Plant Growth-Promoting Rhizobacteria. *J Plant Growth Regul* **2016**, *35*, 877–902, doi:10.1007/s00344-016-9583-4.
389. Pasala, R.K.; Khan, M.I.R.; P.S., M.; M.A., F.; R, S.; Per, T.S.; P.P., D.; N.A., K.; Rane, J. Can Plant Bio-Regulators Minimize Crop Productivity Losses Caused by Drought, Heat and Salinity Stress? An Integrated Review. *Journal of Applied Botany and Food Quality* **2016**, *Vol 89*, p.113125, doi:10.5073/JABFQ.2016.089.014.
390. Patil, C.; Suryawanshi, R.; Koli, S.; Patil, S. Improved Method for Effective Screening of ACC (1-Aminocyclopropane-1-Carboxylate) Deaminase Producing Microorganisms. *Journal of Microbiological Methods* **2016**, *131*, 102–104, doi:10.1016/j.mimet.2016.10.009.
391. Pawlik, M.; Cania, B.; Thijs, S.; Vangronsveld, J.; Piotrowska-Seget, Z. Hydrocarbon Degradation Potential and Plant Growth-Promoting Activity of Culturable Endophytic Bacteria of *Lotus Corniculatus* and *Oenothera biennis* from a Long-Term Polluted Site. *Environ Sci Pollut Res* **2017**, *24*, 19640–19652, doi:10.1007/s11356-017-9496-1.
392. Pedrosa, F.O.; Monteiro, R.A.; Wassem, R.; Cruz, L.M.; Ayub, R.A.; Colauto, N.B.; Fernandez, M.A.; Fungaro, M.H.P.; Grisard, E.C.; Hungria, M.; et al. Genome of *Herbaspirillum* *seropedicae* Strain SmR1, a Specialized Diazotrophic Endophyte of Tropical Grasses. *PLoS Genet* **2011**, *7*, e1002064, doi:10.1371/journal.pgen.1002064.
393. Peng, J.; Wu, D.; Liang, Y.; Li, L.; Guo, Y. Disruption of *AcdS* Gene Reduces Plant Growth Promotion Activity and Maize Saline Stress Resistance by *Rahnella Aquatilis* HX2. *J Basic Microbiol* **2018**, *59*, 402–411, doi:10.1002/jobm.201800510.
394. Penrose, D.M.; Glick, B.R. Levels of ACC and Related Compounds in Exudate and Extracts of Canola Seeds Treated with ACC Deaminase-Containing Plant Growth-Promoting Bacteria. *Can. J. Microbiol.* **2001**, *47*, 368–372, doi:10.1139/w01-014.
395. Phurailatpam, L.; Goyal, D.; Mishra, S. Microbe-Mediated Amelioration Of Salinity Stress In Plants. *EBR* **2021**, *1*, 80–96, doi:10.47204/EBR.1.1.2021.80-96.
396. Pourbabaee, A.A.; Bahmani, E.; Alikhani, H.A.; Emami, S. Promotion of Wheat Growth under Salt Stress by Halotolerant Bacteria Containing ACC Deaminase. *J. Agr. Tech.* **2016**, *18*, 855-864.
397. Poveda, J. Trichoderma Parareesei Favors the Tolerance of Rapeseed (Brassica Napus L.) to Salinity and Drought Due to a Chorismate Mutase. *Agronomy* **2020**, *10*, 118, doi:10.3390/agronomy10010118.
398. Poveda, J.; Jiménez-Gómez, A.; Saati-Santamaría, Z.; Usategui-Martín, R.; Rivas, R.; García-Fraile, P. Mealworm Frass as a Potential Biofertilizer and Abiotic Stress Tolerance-Inductor in Plants. *Applied Soil Ecology* **2019**, *142*, 110–122, doi:10.1016/j.apsoil.2019.04.016.
399. Prakamhang, J.; Tittabutr, P.; Boonkerd, N.; Teamtisong, K.; Uchiumi, T.; Abe, M.; Teaumroong, N. Proposed Some Interactions at Molecular Level of PGPR Coinoculated with *Bradyrhizobium Diazoefficiens* USDA110 and *B. Japonicum* THA6 on Soybean Symbiosis and Its Potential of Field Application. *Applied Soil Ecology* **2015**, *85*, 38–49, doi:10.1016/j.apsoil.2014.08.009.
400. Pramanik, K., Soren, T., Mitra, S., & Maiti, T. K. In silico structural and functional analysis of Mesorhizobium ACC deaminase. *Computational Biology and Chemistry* **2017**, *68*, 12-21.
401. Pramanik, K.; Mandal, N.C. Structural Heterogeneity Assessment among the Isoforms of Fungal 1-Aminocyclopropane-1-Carboxylic Acid (ACC) Deaminase: A Comparative in Silico Perspective. *J Genet Eng Biotechnol* **2022**, *20*, 18, doi:10.1186/s43141-021-00294-0.
402. Pranaw, K.; Pidlisnyuk, V.; Trögl, J.; Malinská, H. Bioprospecting of a Novel Plant Growth-Promoting Bacterium Bacillus Altitudinis KP-14 for Enhancing Miscanthus × Giganteus Growth in Metals Contaminated Soil. *Biology* **2020**, *9*, 305, doi:10.3390/biology9090305.
403. Prudêncio de Araújo, V.L.V.; Lira Junior, M.A.; Souza Júnior, V.S. de; de Araújo Filho, J.C.; Cury Fracetto, F.J.; Andreote, F.D.; de Araujo Pereira, A.P.; Mendes Júnior, J.P.; Rêgo Barros, F.M. do; Monteiro Fracetto, G.G. Bacteria from Tropical Semiarid Temporary Ponds Promote Maize Growth under Hydric Stress. *Microbiological Research* **2020**, *240*, 126564, doi:10.1016/j.micres.2020.126564.
404. Qaderi, M.M.; Islam, M.A.; Reid, D.M.; Shah, S. Do Low-Ethylene-Producing Transgenic Canola ( *Brassica Napus* ) Plants Expressing the ACC Deaminase Gene Differ from Wild-Type Plants in Response to UVB Radiation? *Can. J. Bot.* **2007**, *85*, 148–159, doi:10.1139/b06-157.
405. Qi, W.; Zhao, L. Study of the Siderophore-Producing *Trichoderma Asperellum* Q1 on Cucumber Growth Promotion under Salt Stress: Genetic Immobilization of a Cellulase on Yeast Cell Surface. *J. Basic Microbiol* **2012**, *53*, 355–364, doi:10.1002/jobm.201200031.
406. Qin, S.; Miao, Q.; Feng, W.-W.; Wang, Y.; Zhu, X.; Xing, K.; Jiang, J.-H. Biodiversity and Plant Growth Promoting Traits of Culturable Endophytic Actinobacteria Associated with Jatropha Curcas L. Growing in Panxi Dry-Hot Valley Soil. *Applied Soil Ecology* **2015**, *93*, 47–55, doi:10.1016/j.apsoil.2015.04.004.
407. Qin, S.; Zhang, Y.-J.; Yuan, B.; Xu, P.-Y.; Xing, K.; Wang, J.; Jiang, J.-H. Isolation of ACC Deaminase-Producing Habitat-Adapted Symbiotic Bacteria Associated with Halophyte Limonium Sinense (Girard) Kuntze and Evaluating Their Plant Growth-Promoting Activity under Salt Stress. *Plant Soil* **2014**, *374*, 753–766, doi:10.1007/s11104-013-1918-3.
408. Qin, Y.; Druzhinina, I.S.; Pan, X.; Yuan, Z. Microbially Mediated Plant Salt Tolerance and Microbiome-Based Solutions for Saline Agriculture. *Biotechnology Advances* **2016**, *34*, 1245–1259, doi:10.1016/j.biotechadv.2016.08.005.
409. Qu, Q.; Zhang, Z.; Peijnenburg, W.J.G.M.; Liu, W.; Lu, T.; Hu, B.; Chen, J.; Chen, J.; Lin, Z.; Qian, H. Rhizosphere Microbiome Assembly and Its Impact on Plant Growth. *J. Agric. Food Chem.* **2020**, *68*, 5024–5038, doi:10.1021/acs.jafc.0c00073.
410. Radhakrishnan, R.; Hashem, A.; Abd_Allah, E.F. Bacillus: A Biological Tool for Crop Improvement through Bio-Molecular Changes in Adverse Environments. *Front. Physiol.* **2017**, *8*, 667, doi:10.3389/fphys.2017.00667.
411. Radwan, O. Genomics Improvement of Agronomic Crops to Abiotic Stress. *JIG* **2015**, *2*, 58-63, doi:10.15406/jig.2015.02.00026.
412. Rai, A.; Borpatragohain, B.; Sahoo, S. Role of Plant-Microbe Interactions on Abiotic Stress Tolerance in Plants: A Review. *Int. J Agric. Plant Sci.* **2019**, *1*, 25-31.
413. Rai, P.K.; Singh, M.; Anand, K.; Saurabh, S.; Kaur, T.; Kour, D.; Yadav, A.N.; Kumar, M. Role and Potential Applications of Plant Growth-Promoting Rhizobacteria for Sustainable Agriculture. In *New and Future Developments in Microbial Biotechnology and Bioengineering*; Elsevier, 2020; pp. 49–60 ISBN 978-0-12-820526-6.
414. Rajkumar, M.; Sandhya, S.; Prasad, M.N.V.; Freitas, H. Perspectives of Plant-Associated Microbes in Heavy Metal Phytoremediation. *Biotechnology Advances* **2012**, *30*, 1562–1574, doi:10.1016/j.biotechadv.2012.04.011.
415. Ramakrishna, W.; Rathore, P.; Kumari, R.; Yadav, R. Brown Gold of Marginal Soil: Plant Growth Promoting Bacteria to Overcome Plant Abiotic Stress for Agriculture, Biofuels and Carbon Sequestration. *Science of The Total Environment* **2020**, *711*, 135062, doi:10.1016/j.scitotenv.2019.135062.
416. Rani, S.; Kumar, P.; Suneja, P. Biotechnological Interventions for Inducing Abiotic Stress Tolerance in Crops. *Plant Gene* **2021**, *27*, 100315, doi:10.1016/j.plgene.2021.100315.
417. Rasche, F.; Marco-Noales, E.; Velvis, H.; van Overbeek, L.S.; López, M.M.; van Elsas, J.D.; Sessitsch, A. Structural Characteristics and Plant-Beneficial Effects of Bacteria Colonizing the Shoots of Field Grown Conventional and Genetically Modified T4-Lysozyme Producing Potatoes. *Plant Soil* **2006**, *289*, 123–140, doi:10.1007/s11104-006-9103-6.
418. Rashid, S.; Charles, T.C.; Glick, B.R. Isolation and Characterization of New Plant Growth-Promoting Bacterial Endophytes. *Applied Soil Ecology* **2012**, *61*, 217–224, doi:10.1016/j.apsoil.2011.09.011.
419. Rashid, U.; Yasmin, H.; Hassan, M.N.; Naz, R.; Nosheen, A.; Sajjad, M.; Ilyas, N.; Keyani, R.; Jabeen, Z.; Mumtaz, S.; et al. Drought-Tolerant Bacillus Megaterium Isolated from Semi-Arid Conditions Induces Systemic Tolerance of Wheat under Drought Conditions. *Plant Cell Rep* **2022**, *41*, 549–569, doi:10.1007/s00299-020-02640-x.
420. Rastogi, S.; Shah, S.; Kumar, R.; Vashisth, D.; Akhtar, M.Q.; Kumar, A.; Dwivedi, U.N.; Shasany, A.K. Ocimum Metabolomics in Response to Abiotic Stresses: Cold, Flood, Drought and Salinity. *PLoS ONE* **2019**, *14*, e0210903, doi:10.1371/journal.pone.0210903.
421. Ravanbakhsh, M.; Kowalchuk, G.A.; Jousset, A. Optimization of Plant Hormonal Balance by Microorganisms Prevents Plant Heavy Metal Accumulation. *Journal of Hazardous Materials* **2019**, *379*, 120787, doi:10.1016/j.jhazmat.2019.120787.
422. Ravanbakhsh, M.; Kowalchuk, G.A.; Jousset, A. Root-Associated Microorganisms Reprogram Plant Life History along the Growth–Stress Resistance Tradeoff. *ISME J* **2019**, *13*, 3093–3101, doi:10.1038/s41396-019-0501-1.
423. Ravanbakhsh, M.; Sasidharan, R.; Voesenek, L.A.C.J.; Kowalchuk, G.A.; Jousset, A. Microbial Modulation of Plant Ethylene Signaling: Ecological and Evolutionary Consequences. *Microbiome* **2018**, *6*, 52, doi:10.1186/s40168-018-0436-1.
424. Rehman, F.; Kalsoom, M.; Adnan, M.; Toor, M. D.; Zulfiqar, A. Plant Growth Promoting Rhizobacteria and Their Mechanisms Involved in Agricultural Crop Production: A Review. *SunText Rev Biotechnol* **2020**, *2*, 100, doi:10.51737/2766-5097.2020.010.
425. Riyazuddin, R.; Verma, R.; Singh, K.; Nisha, N.; Keisham, M.; Bhati, K.K.; Kim, S.T.; Gupta, R. Ethylene: A Master Regulator of Salinity Stress Tolerance in Plants. *Biomolecules* **2020**, *10*, 959, doi:10.3390/biom10060959.
426. Rizvi, A.; Khan, Mohd.S. Heavy Metal Induced Oxidative Damage and Root Morphology Alterations of Maize (Zea Mays L.) Plants and Stress Mitigation by Metal Tolerant Nitrogen Fixing Azotobacter Chroococcum. *Ecotoxicology and Environmental Safety* **2018**, *157*, 9–20, doi:10.1016/j.ecoenv.2018.03.063.
427. Roy Choudhury, A.; Roy, S.K.; Trivedi, P.; Choi, J.; Cho, K.; Yun, S.H.; Walitang, D.I.; Park, J.; Kim, K.; Sa, T. Label‐free Proteomics Approach Reveals Candidate Proteins in Rice ( *Oryza Sativa* L.) Important for ACC Deaminase Producing Bacteria‐mediated Tolerance against Salt Stress. *Environmental Microbiology* **2022**, 1462-2920.15937, doi:10.1111/1462-2920.15937.
428. Ruzzi, M.; Aroca, R. Plant Growth-Promoting Rhizobacteria Act as Biostimulants in Horticulture. *Scientia Horticulturae* **2015**, *196*, 124–134, doi:10.1016/j.scienta.2015.08.042.
429. Saakre, M.; Baburao, T.M.; Salim, A.P.; Ffancies, R.M.; Achuthan, V.P.; Thomas, G.; Sivarajan, S.R. Identification and Characterization of Genes Responsible for Drought Tolerance in Rice Mediated by *Pseudomonas Fluorescens*. *Rice Science* **2017**, *24*, 291–298, doi:10.1016/j.rsci.2017.04.005.
430. Saberi Riseh, R.; Ebrahimi-Zarandi, M.; Tamanadar, E.; Moradi Pour, M.; Thakur, V.K. Salinity Stress: Toward Sustainable Plant Strategies and Using Plant Growth-Promoting Rhizobacteria Encapsulation for Reducing It. *Sustainability* **2021**, *13*, 12758, doi:10.3390/su132212758.
431. Safari, D.; Jamali, F.; Nooryazdan, H. R. Evaluation of ACC Deaminase Producing Pseudomonas Fluorescens Strains for Their Effects on Seed Germination and Early Growth of Wheat under Salt Stress. *Aust J Crop Sci* **2018**, *12*, 413–421, doi:10.21475/ajcs.18.12.03.pne801.
432. Safdarian, M.; Askari, H.; Nematzadeh, G.; Sofo, A. Halophile Plant Growth-Promoting Rhizobacteria Induce Salt Tolerance Traits in Wheat Seedlings (Triticum Aestivum L.). *Pedosphere* **2020**, *30*, 684–693, doi:10.1016/S1002-0160(19)60835-0.
433. Safdarian, M.; Askari, H.; Shariati J., V.; Nematzadeh, G. Transcriptional Responses of Wheat Roots Inoculated with *Arthrobacter Nitroguajacolicus* to Salt Stress. *Sci Rep* **2019**, *9*, 1792, doi:10.1038/s41598-018-38398-2.
434. Sagar, A.; Rathore, P.; Ramteke, P.W.; Ramakrishna, W.; Reddy, M.S.; Pecoraro, L. Plant Growth Promoting Rhizobacteria, Arbuscular Mycorrhizal Fungi and Their Synergistic Interactions to Counteract the Negative Effects of Saline Soil on Agriculture: Key Macromolecules and Mechanisms. *Microorganisms* **2021**, *9*, 1491, doi:10.3390/microorganisms9071491.
435. Saghafi, D.; Delangiz, N.; Lajayer, B.A.; Ghorbanpour, M. An Overview on Improvement of Crop Productivity in Saline Soils by Halotolerant and Halophilic PGPRs. *3 Biotech* **2019**, *9*, 261, doi:10.1007/s13205-019-1799-0.
436. Saikia, J.; Sarma, R.K.; Dhandia, R.; Yadav, A.; Bharali, R.; Gupta, V.K.; Saikia, R. Alleviation of Drought Stress in Pulse Crops with ACC Deaminase Producing Rhizobacteria Isolated from Acidic Soil of Northeast India. *Sci Rep* **2018**, *8*, 3560, doi:10.1038/s41598-018-21921-w.
437. Saleem, A.R.; Brunetti, C.; Khalid, A.; Della Rocca, G.; Raio, A.; Emiliani, G.; De Carlo, A.; Mahmood, T.; Centritto, M. Drought Response of Mucuna Pruriens (L.) DC. Inoculated with ACC Deaminase and IAA Producing Rhizobacteria. *PLoS ONE* **2018**, *13*, e0191218, doi:10.1371/journal.pone.0191218.
438. Saleem, M.; Arshad, M.; Hussain, S.; Bhatti, A.S. Perspective of Plant Growth Promoting Rhizobacteria (PGPR) Containing ACC Deaminase in Stress Agriculture. *J Ind Microbiol Biotechnol* **2007**, *34*, 635–648, doi:10.1007/s10295-007-0240-6.
439. Saleem, S.; Ul Mushtaq, N.; Hafiz Shah, W.; Rasool, A.; Rehman Hakeem, K.; Ul Rehman, R. Morpho-Physiological, Biochemical and Molecular Adaptation of Millets to Abiotic Stresses: A Review. *Phyton* **2021**, *90*, 1363–1385, doi:10.32604/phyton.2021.014826.
440. Salem, G.; Stromberger, M.E.; Byrne, P.F.; Manter, D.K.; El-Feki, W.; Weir, T.L. Genotype-Specific Response of Winter Wheat (Triticum Aestivum L.) to Irrigation and Inoculation with ACC Deaminase Bacteria. *Rhizosphere* **2018**, *8*, 1–7, doi:10.1016/j.rhisph.2018.08.001.
441. Salwan, R.; Sharma, A.; Sharma, V. Microbes Mediated Plant Stress Tolerance in Saline Agricultural Ecosystem. *Plant Soil* **2019**, *442*, 1–22, doi:10.1007/s11104-019-04202-x.
442. Samaddar, S.; Chatterjee, P.; Roy Choudhury, A.; Ahmed, S.; Sa, T. Interactions between Pseudomonas Spp. and Their Role in Improving the Red Pepper Plant Growth under Salinity Stress. *Microbiological Research* **2019**, *219*, 66–73, doi:10.1016/j.micres.2018.11.005.
443. Sandhya, V.; Shrivastava, M.; Ali, Sk.Z.; Sai Shiva Krishna Prasad, V. Endophytes from Maize with Plant Growth Promotion and Biocontrol Activity under Drought Stress. *Russ. Agricult. Sci.* **2017**, *43*, 22–34, doi:10.3103/S1068367417010165.
444. Sangiorgio, D.; Cellini, A.; Donati, I.; Pastore, C.; Onofrietti, C.; Spinelli, F. Facing Climate Change: Application of Microbial Biostimulants to Mitigate Stress in Horticultural Crops. *Agronomy* **2020**, *10*, 794, doi:10.3390/agronomy10060794.
445. Santos, A.P.; Belfiore, C.; Úrbez, C.; Ferrando, A.; Blázquez, M.A.; Farías, M.E. Extremophiles as Plant Probiotics to Promote Germination and Alleviate Salt Stress in Soybean. *J Plant Growth Regul* **2022**, 1-14, doi:10.1007/s00344-022-10605-5.
446. Santoyo, G.; Guzmán-Guzmán, P.; Parra-Cota, F.I.; Santos-Villalobos, S. de los; Orozco-Mosqueda, Ma. del C.; Glick, B.R. Plant Growth Stimulation by Microbial Consortia. *Agronomy* **2021**, *11*, 219, doi:10.3390/agronomy11020219.
447. Sapre, S.; Gontia-Mishra, I.; Tiwari, S. Klebsiella Sp. Confers Enhanced Tolerance to Salinity and Plant Growth Promotion in Oat Seedlings (Avena Sativa). *Microbiological Research* **2018**, *206*, 25–32, doi:10.1016/j.micres.2017.09.009.
448. Saraf, M.; Jha, C.K.; Patel, D. The Role of ACC Deaminase Producing PGPR in Sustainable Agriculture. In *Plant Growth and Health Promoting Bacteria*; Maheshwari, D.K., Ed.; Microbiology Monographs; Springer Berlin Heidelberg: Berlin, Heidelberg, 2010; Vol. 18, pp. 365–385 ISBN 978-3-642-13611-5.
449. Sarapat, S.; Longtonglang, A.; Umnajkitikorn, K.; Girdthai, T.; Boonkerd, N.; Tittabutr, P.; Teaumroong, N. Application of Rice Endophytic *Bradyrhizobium* Strain SUTN9-2 Containing Modified ACC Deaminase to Rice Cultivation under Water Deficit Conditions. *Journal of Plant Interactions* **2020**, *15*, 322–334, doi:10.1080/17429145.2020.1824028.
450. Saravanakumar, D.; Samiyappan, R. ACC Deaminase from Pseudomonas Fluorescens Mediated Saline Resistance in Groundnut (Arachis Hypogea) Plants. *J Appl Microbiol* **2007**, *102*, 1283–1292, doi:10.1111/j.1365-2672.2006.03179.x.
451. Sarkar, A.; Ghosh, P.K.; Pramanik, K.; Mitra, S.; Soren, T.; Pandey, S.; Mondal, M.H.; Maiti, T.K. A Halotolerant *Enterobacter* Sp. Displaying ACC Deaminase Activity Promotes Rice Seedling Growth under Salt Stress. *Research in Microbiology* **2018**, *169*, 20–32, doi:10.1016/j.resmic.2017.08.005.
452. Sarkar, A.; Pramanik, K.; Mitra, S.; Soren, T.; Maiti, T.K. Enhancement of Growth and Salt Tolerance of Rice Seedlings by ACC Deaminase-Producing Burkholderia Sp. MTCC 12259. *Journal of Plant Physiology* **2018**, *231*, 434–442, doi:10.1016/j.jplph.2018.10.010.
453. Sarma, R.K.; Saikia, R. Alleviation of Drought Stress in Mung Bean by Strain Pseudomonas Aeruginosa GGRJ21. *Plant Soil* **2014**, *377*, 111–126, doi:10.1007/s11104-013-1981-9.
454. Sathya, A.; Vijayabharathi, R.; Gopalakrishnan, S. Plant Growth-Promoting Actinobacteria: A New Strategy for Enhancing Sustainable Production and Protection of Grain Legumes. *3 Biotech* **2017**, *7*, 102, doi:10.1007/s13205-017-0736-3.
455. Satyanarayana, T., Das, S.K., Johri, B.N. *Microbial Diversity in Ecosystem Sustainability and Biotechnological Applications: Volume 2. Soil & Agroecosystems*; Eds.; Springer Singapore: Singapore, 2019; ISBN 9789811384868.
456. Saxena, A.K.; Kumar, M.; Chakdar, H.; Anuroopa, N.; Bagyaraj, D.J. *Bacillus* Species in Soil as a Natural Resource for Plant Health and Nutrition. *J Appl Microbiol* **2020**, *128*, 1583–1594, doi:10.1111/jam.14506.
457. Saxena, R.; Kumar, M.; Tomar, R.S. Plant–Rhizobacteria Interactions to Induce Biotic and Abiotic Stress Tolerance in Plants. In *Plant, Soil and Microbes in Tropical Ecosystems*; Dubey, S.K., Verma, S.K., Eds.; Rhizosphere Biology; Springer Singapore: Singapore, 2021; pp. 1–18 ISBN 9789811633638.
458. Sayyed, R.Z., Arora, N.K., Reddy, M.S. *Plant Growth Promoting Rhizobacteria for Sustainable Stress Management: Volume 1: Rhizobacteria in Abiotic Stress Management*; Eds.; Microorganisms for Sustainability; Springer Singapore: Singapore, 2019; Vol. 12; ISBN 9789811365355.
459. Sayyed, R.Z.; Reddy, M.S.; Al-Turki, A.I. Recent Trends in PGPR Research for Sustainable Crop Productivity.; Scientific publishers: Jodhpur, India, 2016, pp. 258. ISBN 978-81-7233-990-6.
460. Sayyed, Riyazali Zafarali, ed. *Plant Growth Promoting Rhizobacteria for Sustainable Stress Management: Volume 2: Rhizobacteria in Biotic Stress Management*. Vol. 13. Springer Nature, 2019.
461. Schellingen, K.; Van Der Straeten, D.; Vandenbussche, F.; Prinsen, E.; Remans, T.; Vangronsveld, J.; Cuypers, A. Cadmium-Induced Ethylene Production and Responses in *Arabidopsis Thaliana* Rely on *ACS2* and *ACS6* Gene Expression. *BMC Plant Biol* **2014**, *14*, 214, doi:10.1186/s12870-014-0214-6.
462. Sepúlveda-Caamaño, M.; Gerding, M.; Vargas, M.; Moya-Elizondo, E.; Oyarzúa, P.; Campos, J. Lentil ( *Lens Culinaris* L.) Growth Promoting Rhizobacteria and Their Effect on Nodulation in Coinoculation with Rhizobia. *Archives of Agronomy and Soil Science* **2018**, *64*, 244–256, doi:10.1080/03650340.2017.1342034.
463. Sgroy, V.; Cassán, F.; Masciarelli, O.; Del Papa, M.F.; Lagares, A.; Luna, V. Isolation and Characterization of Endophytic Plant Growth-Promoting (PGPB) or Stress Homeostasis-Regulating (PSHB) Bacteria Associated to the Halophyte Prosopis Strombulifera. *Appl Microbiol Biotechnol* **2009**, *85*, 371–381, doi:10.1007/s00253-009-2116-3.
464. Shabanamol, S.; Divya, K.; George, T.K.; Rishad, K.S.; Sreekumar, T.S.; Jisha, M.S. Characterization and in Planta Nitrogen Fixation of Plant Growth Promoting Endophytic Diazotrophic Lysinibacillus Sphaericus Isolated from Rice (Oryza Sativa). *Physiological and Molecular Plant Pathology* **2018**, *102*, 46–54, doi:10.1016/j.pmpp.2017.11.003.
465. Shah, S.; Li, J.; Moffatt, B.A.; Glick, B.R. Isolation and Characterization of ACC Deaminase Genes from Two Different Plant Growth-Promoting Rhizobacteria. **1998**, *44*, 11.
466. Shaharoona, B., Jamro, G. M., Zahir, Z. A., Arshad, M., & Memon, K. S. Effectiveness of various Pseudomonas spp. and Burkholderia caryophylli containing ACC-deaminase for improving growth and yield of wheat (Triticum aestivum L.). *Journal of microbiology and biotechnology* **2007**, *17*(8), 1300-1307.
467. Shaharoona, B.; Bibi, R.; Arshad, M.; Ahmed, Z. 1-AMINOCYLOPROPANE-1-CARBOXYLATE (ACC)- DEAMINASE RHIZOBACTERIA EXTENUATES ACC-INDUCED CLASSICAL TRIPLE RESPONSE IN ETIOLATED PEA SEEDLINGS. *Park J Bot* **2006**, 38, 1491-1499.
468. Shaharoona, B.; Imran, M.; Arshad, M.; Khalid, A. Manipulation of Ethylene Synthesis in Roots Through Bacterial ACC Deaminase for Improving Nodulation in Legumes. *Critical Reviews in Plant Sciences* **2011**, *30*, 279–291, doi:10.1080/07352689.2011.572058.
469. Shahid, M.; Ahmed, T.; Noman, M.; Javed, M.T.; Javed, M.R.; Tahir, M.; Shah, S.M. Non-Pathogenic Staphylococcus Strains Augmented the Maize Growth through Oxidative Stress Management and Nutrient Supply under Induced Salt Stress. *Ann Microbiol* **2019**, *69*, 727–739, doi:10.1007/s13213-019-01464-9.
470. Shahid, M.; Javed, M.T.; Mushtaq, A.; Akram, M.S.; Mahmood, F.; Ahmed, T.; Noman, M.; Azeem, M. Microbe-Mediated Mitigation of Cadmium Toxicity in Plants. In *Cadmium Toxicity and Tolerance in Plants*; Elsevier, 2019; pp. 427–449 ISBN 978-0-12-814864-8.
471. Shahid, M.; Mahmood, F.; Hussain, S.; Shahzad, T.; Haider, M.Z.; Noman, M.; Mushtaq, A.; Fatima, Q.; Ahmed, T.; Mustafa, G. Enzymatic Detoxification of Azo Dyes by a Multifarious Bacillus Sp. Strain MR-1/2-Bearing Plant Growth-Promoting Characteristics. *3 Biotech* **2018**, *8*, 425, doi:10.1007/s13205-018-1442-5.
472. Shahid, M.; Shah, A.A.; Basit, F.; Noman, M.; Zubair, M.; Ahmed, T.; Naqqash, T.; Manzoor, I.; Maqsood, A. Achromobacter Sp. FB-14 Harboring ACC Deaminase Activity Augmented Rice Growth by Upregulating the Expression of Stress-Responsive CIPK Genes under Salinity Stress. *Braz J Microbiol* **2020**, *51*, 719–728, doi:10.1007/s42770-019-00199-8.
473. Sharma, A.; Kashyap, P.L.; Srivastava, A.K.; Bansal, Y.K.; Kaushik, R. Isolation and Characterization of Halotolerant Bacilli from Chickpea (Cicer Arietinum L.) Rhizosphere for Plant Growth Promotion and Biocontrol Traits. *Eur J Plant Pathol* **2019**, *153*, 787–800, doi:10.1007/s10658-018-1592-7.
474. Sharma, A.; Singh, R.K.; Singh, P.; Vaishnav, A.; Guo, D.-J.; Verma, K.K.; Li, D.-P.; Song, X.-P.; Malviya, M.K.; Khan, N.; et al. Insights into the Bacterial and Nitric Oxide-Induced Salt Tolerance in Sugarcane and Their Growth-Promoting Abilities. *Microorganisms* **2021**, *9*, 2203, doi:10.3390/microorganisms9112203.
475. Sharp, R.G.; Chen, L.; Davies, W.J. Inoculation of Growing Media with the Rhizobacterium Variovorax Paradoxus 5C-2 Reduces Unwanted Stress Responses in Hardy Ornamental Species. *Scientia Horticulturae* **2011**, *129*, 804–811, doi:10.1016/j.scienta.2011.03.016.
476. Shilev, S. Plant-Growth-Promoting Bacteria Mitigating Soil Salinity Stress in Plants. *Applied Sciences* **2020**, *10*, 7326, doi:10.3390/app10207326.
477. Shin, W.; Siddikee, Md.A.; Joe, M.M.; Benson, A.; Kim, K.; Selvakumar, G.; Kang, Y.; Jeon, S.; Samaddar, S.; Chatterjee, P.; et al. Halotolerant Plant Growth Promoting Bacteria Mediated Salinity Stress Amelioration in Plants. *Korean Journal of Soil Science and Fertilizer* **2016**, *49*, 355–367, doi:10.7745/KJSSF.2016.49.4.355.
478. Shrivastava, P.; Kumar, R. Soil Salinity: A Serious Environmental Issue and Plant Growth Promoting Bacteria as One of the Tools for Its Alleviation. *Saudi Journal of Biological Sciences* **2015**, *22*, 123–131, doi:10.1016/j.sjbs.2014.12.001.
479. Siddikee, M.A.; Zereen, M.I.; Wu, M.; Zhang, W.; Dai, C.-C. Phomopsis Liquidambaris Reduces Ethylene Biosynthesis in Rice under Salt Stress via Inhibiting the Activity of 1-Aminocyclopropane-1-Carboxylate Deaminase. *Arch Microbiol* **2021**, *203*, 6215–6229, doi:10.1007/s00203-021-02588-w.
480. Siddikee, Md.A.; Chauhan, P.S.; Sa, T. Regulation of Ethylene Biosynthesis Under Salt Stress in Red Pepper (Capsicum Annuum L.) by 1-Aminocyclopropane-1-Carboxylic Acid (ACC) Deaminase-Producing Halotolerant Bacteria. *J Plant Growth Regul* **2012**, *31*, 265–272, doi:10.1007/s00344-011-9236-6.
481. Siddikee, Md.A.; Chauhan, Puneet.S.; Anandham, R.; Han, G.-H.; Sa, T. Isolation, Characterization, and Use for Plant Growth Promotion Under Salt Stress, of ACC Deaminase-Producing Halotolerant Bacteria Derived from Coastal Soil. *J. Microbiol. Biotechnol.* **2010**, *20*, 1577–1584, doi:10.4014/jmb.1007.07011.
482. Siddique, M.S.; Qadir, G.; Gill, S.M.; Sultan, T.; Hayyat, R. Evaluation of PGPR Strains Having ACC Deaminase Activity for Their Efficacy to Induce Water Stress Tolerance in Sunflower under Varied Moisture Regimes. *Internation Journal of Bioscience* **2019**, *14,* 419-429.
483. Silambarasan, S.; Logeswari, P.; Cornejo, P.; Abraham, J.; Valentine, A. Simultaneous Mitigation of Aluminum, Salinity and Drought Stress in Lactuca Sativa Growth via Formulated Plant Growth Promoting Rhodotorula Mucilaginosa CAM4. *Ecotoxicology and Environmental Safety* **2019**, *180*, 63–72, doi:10.1016/j.ecoenv.2019.05.006.
484. Silambarasan, S.; Logeswari, P.; Cornejo, P.; Kannan, V.R. Evaluation of the Production of Exopolysaccharide by Plant Growth Promoting Yeast Rhodotorula Sp. Strain CAH2 under Abiotic Stress Conditions. *International Journal of Biological Macromolecules* **2019**, *121*, 55–62, doi:10.1016/j.ijbiomac.2018.10.016.
485. Silambarasan, S.; Logeswari, P.; Cornejo, P.; Kannan, V.R. Role of Plant Growth–Promoting Rhizobacterial Consortium in Improving the Vigna Radiata Growth and Alleviation of Aluminum and Drought Stresses. *Environ Sci Pollut Res* **2019**, *26*, 27647–27659, doi:10.1007/s11356-019-05939-9.
486. Sindhu, S.; Dahiya, A.; Gera, R.; Sindhu, S.S. Mitigation of Abiotic Stress in Legume-Nodulating Rhizobia for Sustainable Crop Production. *Agric Res* **2020**, *9*, 444–459, doi:10.1007/s40003-020-00474-3.
487. Singh, B., Gowtham, H. G., & Niranjana, S. R. ACC deaminase producing PGPR invoke changes in antioxidant systems to minimize the adverse effects of salt in sunflower. RJLBPCS **2019**, 5, 1-15.
488. Singh, D.P., Gupta, V.K., Prabha, R. *Microbial Interventions in Agriculture and Environment: Volume 2: Rhizosphere, Microbiome and Agro-Ecology*; Eds.; Springer Singapore: Singapore, 2019; ISBN 9789811383823.
489. Singh, D.P.; Singh, V.; Gupta, V.K.; Shukla, R.; Prabha, R.; Sarma, B.K.; Patel, J.S. Microbial Inoculation in Rice Regulates Antioxidative Reactions and Defense Related Genes to Mitigate Drought Stress. *Sci Rep* **2020**, *10*, 4818, doi:10.1038/s41598-020-61140-w.
490. Singh, H.B., Vaishnav, A., Sayyed, R.Z. *Antioxidants in Plant-Microbe Interaction*; Eds.; Springer Singapore: Singapore, 2021; ISBN 9789811613494.
491. Singh, P., Singh, R. K., Guo, D. J., Sharma, A., Singh, R. N., Li, D. P., ... & Li, Y. R. Whole genome analysis of sugarcane root-associated endophyte Pseudomonas aeruginosa B18—a plant growth-promoting bacterium with antagonistic potential against Sporisorium scitamineum. *Frontiers in Microbiology* **2021**, *12*, 628376.
492. Singh, P.; Pandey, S.S.; Dubey, B.K.; Raj, R.; Barnawal, D.; Chandran, A.; Rahman, L. ur Salt and Drought Stress Tolerance with Increased Biomass in Transgenic Pelargonium Graveolens through Heterologous Expression of ACC Deaminase Gene from Achromobacter Xylosoxidans. *Plant Cell Tiss Organ Cult* **2021**, *147*, 297–311, doi:10.1007/s11240-021-02124-0.
493. Singh, P.; Singh, R.K.; Li, H.-B.; Guo, D.-J.; Sharma, A.; Lakshmanan, P.; Malviya, M.K.; Song, X.-P.; Solanki, M.K.; Verma, K.K.; et al. Diazotrophic Bacteria *Pantoea Dispersa* and *Enterobacter Asburiae* Promote Sugarcane Growth by Inducing Nitrogen Uptake and Defense-Related Gene Expression. *Front. Microbiol.* **2021**, *11*, 600417, doi:10.3389/fmicb.2020.600417.
494. Singh, R. K., Singh, P., Guo, D. J., Sharma, A., Li, D. P., Li, X., ... & Li, Y. R. Root-derived endophytic diazotrophic bacteria Pantoea cypripedii AF1 and Kosakonia arachidis EF1 promote nitrogen assimilation and growth in sugarcane. *Frontiers in Microbiology* **2021**, 3768.
495. Singh, R.P., Kothari, R., Koringa, P.G., Singh, S.P. *Understanding Host-Microbiome Interactions - An Omics Approach*; Eds.; Springer Singapore: Singapore, 2017; ISBN 978-981-10-5049-7.
496. Singh, R.P.; Jha, P.; Jha, P.N. Bio-Inoculation of Plant Growth-Promoting Rhizobacterium Enterobacter Cloacae ZNP-3 Increased Resistance Against Salt and Temperature Stresses in Wheat Plant (Triticum Aestivum L.). *J Plant Growth Regul* **2017**, *36*, 783–798, doi:10.1007/s00344-017-9683-9.
497. Singh, R.P.; Jha, P.; Jha, P.N. The Plant-Growth-Promoting Bacterium Klebsiella Sp. SBP-8 Confers Induced Systemic Tolerance in Wheat (Triticum Aestivum) under Salt Stress. *Journal of Plant Physiology* **2015**, *184*, 57–67, doi:10.1016/j.jplph.2015.07.002.
498. Singh, R.P.; Jha, P.N. A Halotolerant Bacterium Bacillus Licheniformis HSW-16 Augments Induced Systemic Tolerance to Salt Stress in Wheat Plant (*Triticum Aestivum*). *Front. Plant Sci.* **2016**, *7*, 1890 ,doi:10.3389/fpls.2016.01890.
499. Singh, R.P.; Jha, P.N. Alleviation of Salinity-Induced Damage on Wheat Plant by an ACC Deaminase-Producing Halophilic Bacterium Serratia Sp. SL- 12 Isolated from a Salt Lake. *Symbiosis* **2016**, *69*, 101–111, doi:10.1007/s13199-016-0387-x.
500. Singh, R.P.; Jha, P.N. Mitigation of Salt Stress in Wheat Plant (Triticum Aestivum) by ACC Deaminase Bacterium Enterobacter Sp. SBP-6 Isolated from Sorghum Bicolor. *Acta Physiol Plant* **2016**, *38*, 110, doi:10.1007/s11738-016-2123-9.
501. Singh, R.P.; Jha, P.N. The Multifarious PGPR Serratia Marcescens CDP-13 Augments Induced Systemic Resistance and Enhanced Salinity Tolerance of Wheat (Triticum Aestivum L.). *PLoS ONE* **2016**, *11*, e0155026, doi:10.1371/journal.pone.0155026.
502. Singh, R.P.; Jha, P.N. The PGPR Stenotrophomonas Maltophilia SBP-9 Augments Resistance against Biotic and Abiotic Stress in Wheat Plants. *Front. Microbiol.* **2017**, *8*, 1945, doi:10.3389/fmicb.2017.01945.
503. Singh, R.P.; Jha, P.N. Transposon Mutagenesis of ACC Deamination Gene Alters the Proteomic Analysis of Wheat Plant under Non-Saline and Saline Stress. *J Proteins Proteom* **2022**, *13*, 39–53, doi:10.1007/s42485-022-00083-4.
504. Singh, R.P.; Runthala, A.; Khan, S.; Jha, P.N. Quantitative Proteomics Analysis Reveals the Tolerance of Wheat to Salt Stress in Response to Enterobacter Cloacae SBP-8. *PLoS ONE* **2017**, *12*, e0183513, doi:10.1371/journal.pone.0183513.
505. Singh, R.P.; Shelke, G.M.; Kumar, A.; Jha, P.N. Biochemistry and Genetics of ACC Deaminase: A Weapon to “Stress Ethylene” Produced in Plants. *Front. Microbiol.* **2015**, *6*, doi:10.3389/fmicb.2015.00937.
506. Singh, S.; Tripathi, A.; Chanotiya, C.S.; Barnawal, D.; Singh, P.; Patel, V.K.; Vajpayee, P.; Kalra, A. Cold Stress Alleviation Using Individual and Combined Inoculation of ACC Deaminase Producing Microbes in Ocimum Sanctum. *Environmental Sustainability* **2020**, *3*, 289–301, doi:10.1007/s42398-020-00118-w.
507. Singh, S.; Yadav, S.K.; Mishra, P.; Maurya, R.; Rana, V.; Yadav, A.K.; Singh, A.; Ram, G.; Ramteke, P.W. Comparative Analysis of 1-Aminocyclopropane-1-Carboxylate (ACC) Deaminase in Selected Plant Growth Promoting Rhizobacteria (PGPR). *JOURNAL OF PURE AND APPLIED MICROBIOLOGY* **2015**, *9,* 1587-1596.
508. Singh, U.B., Sahu, P.K., Singh, H.V., Sharma, P.K. *Rhizosphere Microbes: Soil and Plant Functions*. Eds.; Microorganisms for Sustainability; Springer Singapore: Singapore, 2020; Vol. 23; ISBN 9789811591532.
509. Singh, V.K.; Singh, A.K.; Singh, P.P.; Kumar, A. Interaction of Plant Growth Promoting Bacteria with Tomato under Abiotic Stress: A Review. *Agriculture, Ecosystems & Environment* **2018**, *267*, 129–140, doi:10.1016/j.agee.2018.08.020.
510. Singha, L.P.; Sinha, N.; Pandey, P. Rhizoremediation Prospects of Polyaromatic Hydrocarbon Degrading Rhizobacteria, That Facilitate Glutathione and Glutathione-S-Transferase Mediated Stress Response, and Enhance Growth of Rice Plants in Pyrene Contaminated Soil. *Ecotoxicology and Environmental Safety* **2018**, *164*, 579–588, doi:10.1016/j.ecoenv.2018.08.069.
511. SkZ, A.; Vardharajula, S.; Vurukonda, S.S.K.P. Transcriptomic Profiling of Maize (*Zea Mays* L.) Seedlings in Response to *Pseudomonas Putida* Stain FBKV2 Inoculation under Drought Stress. *Ann Microbiol* **2018**, *68*, 331–349, doi:10.1007/s13213-018-1341-3.
512. Sofy, M.R.; Aboseidah, A.A.; Heneidak, S.A.; Ahmed, H.R. ACC Deaminase Containing Endophytic Bacteria Ameliorate Salt Stress in Pisum Sativum through Reduced Oxidative Damage and Induction of Antioxidative Defense Systems. *Environ Sci Pollut Res* **2021**, *28*, 40971–40991, doi:10.1007/s11356-021-13585-3.
513. Souza, R. de; Ambrosini, A.; Passaglia, L.M.P. Plant Growth-Promoting Bacteria as Inoculants in Agricultural Soils. *Genet. Mol. Biol.* **2015**, *38*, 401–419, doi:10.1590/S1415-475738420150053.
514. Srinivasan, R.; Subramanian, P.; Tirumani, S.; Gothandam, K.M.; Ramya, M. Ectopic Expression of Bacterial 1-Aminocyclopropane 1-Carboxylate Deaminase in *Chlamydomonas Reinhardtii* Enhances Algal Biomass and Lipid Content under Nitrogen Deficit Condition. *Bioresource Technology* **2021**, *341*, 125830, doi:10.1016/j.biortech.2021.125830.
515. Srivastava, S.; Chaudhry, V.; Mishra, A.; Chauhan, P.S.; Rehman, A.; Yadav, A.; Tuteja, N.; Nautiyal, C.S. Gene Expression Profiling through Microarray Analysis in *Arabidopsis Thaliana* Colonized by *Pseudomonas Putida* MTCC5279, a Plant Growth Promoting Rhizobacterium. *Plant Signaling & Behavior* **2012**, *7*, 235–245, doi:10.4161/psb.18957.
516. Srivastava, S.; Patel, J.S.; Singh, H.B.; Sinha, A.; Sarma, B.K. *Streptomyces Rochei* SM3 Induces Stress Tolerance in Chickpea Against *Sclerotinia Sclerotiorum* and NaCl. *J Phytopathol* **2015**, *163*, 583–592, doi:10.1111/jph.12358.
517. Stearns, J.C.; Woody, O.Z.; McConkey, B.J.; Glick, B.R. Effects of Bacterial ACC Deaminase on *Brassica Napus* Gene Expression. *MPMI* **2012**, *25*, 668–676, doi:10.1094/MPMI-08-11-0213.
518. Stromberger, M.E.; Abduelafez, I.; Byrne, P.; Canela deceased, M.M.; Elamari, A.A.; Manter, D.K.; Weir, T. Genotype-Specific Enrichment of 1-Aminocyclopropane-1-Carboxylic Acid Deaminase-Positive Bacteria in Winter Wheat Rhizospheres. *Soil Science Society of America Journal* **2017**, *81*, 796–805, doi:10.2136/sssaj2016.12.0437.
519. Suarez, C.; Cardinale, M.; Ratering, S.; Steffens, D.; Jung, S.; Montoya, A.M.Z.; Geissler-Plaum, R.; Schnell, S. Plant Growth-Promoting Effects of Hartmannibacter Diazotrophicus on Summer Barley (Hordeum Vulgare L.) under Salt Stress. *Applied Soil Ecology* **2015**, *95*, 23–30, doi:10.1016/j.apsoil.2015.04.017.
520. Subramanian, P.; Krishnamoorthy, R.; Chanratana, M.; Kim, K.; Sa, T. Expression of an Exogenous 1-Aminocyclopropane-1-Carboxylate Deaminase Gene in Psychrotolerant Bacteria Modulates Ethylene Metabolism and Cold Induced Genes in Tomato under Chilling Stress. *Plant Physiology and Biochemistry* **2015**, *89*, 18–23, doi:10.1016/j.plaphy.2015.02.003.
521. Sun, Y.; Cheng, Z.; Glick, B.R. The Presence of a 1-Aminocyclopropane-1-Carboxylate (ACC) Deaminase Deletion Mutation Alters the Physiology of the Endophytic Plant Growth-Promoting Bacterium *Burkholderia Phytofirmans* PsJN. *FEMS Microbiology Letters* **2009**, *296*, 131–136, doi:10.1111/j.1574-6968.2009.01625.x.
522. Tahir, M.; Ahmad, I.; Shahid, M.; Shah, G.M.; Farooq, A.B.U.; Akram, M.; Tabassum, S.A.; Naeem, M.A.; Khalid, U.; Ahmad, S.; et al. Regulation of Antioxidant Production, Ion Uptake and Productivity in Potato (Solanum Tuberosum L.) Plant Inoculated with Growth Promoting Salt Tolerant Bacillus Strains. *Ecotoxicology and Environmental Safety* **2019**, *178*, 33–42, doi:10.1016/j.ecoenv.2019.04.027.
523. Tamot, B.K.; Pauls, K.P.; Glick, B.R. Regulation of Expression of the Prb-1b I ACC Deaminase Gene by UV-8 in Transgenic Tomatoes. *J. Plant Biochemistry & Biotechnology,* **2003**,12, 25-29.
524. Tanpure, R.S.; Ghuge, S.A.; Dawkar, V.V.; Kumar, A. Signaling Responses and Their Role in the Mitigation of Abiotic Stresses. In *Stress Tolerance in Horticultural Crops*; Elsevier, 2021; pp. 327–346 ISBN 978-0-12-822849-4.
525. Timmusk, S.; Abd El-Daim, I.A.; Copolovici, L.; Tanilas, T.; Kännaste, A.; Behers, L.; Nevo, E.; Seisenbaeva, G.; Stenström, E.; Niinemets, Ü. Drought-Tolerance of Wheat Improved by Rhizosphere Bacteria from Harsh Environments: Enhanced Biomass Production and Reduced Emissions of Stress Volatiles. *PLoS ONE* **2014**, *9*, e96086, doi:10.1371/journal.pone.0096086.
526. Timmusk, S.; Paalme, V.; Pavlicek, T.; Bergquist, J.; Vangala, A.; Danilas, T.; Nevo, E. Bacterial Distribution in the Rhizosphere of Wild Barley under Contrasting Microclimates. *PLoS ONE* **2011**, *6*, e17968, doi:10.1371/journal.pone.0017968.
527. Tiryaki, D.; Aydın, İ.; Atıcı, Ö. Psychrotolerant Bacteria Isolated from the Leaf Apoplast of Cold-Adapted Wild Plants Improve the Cold Resistance of Bean (Phaseolus Vulgaris L.) under Low Temperature. *Cryobiology* **2019**, *86*, 111–119, doi:10.1016/j.cryobiol.2018.11.001.
528. Tiwari, G.; Duraivadivel, P.; Sharma, S.; P., H. 1-Aminocyclopropane-1-Carboxylic Acid Deaminase Producing Beneficial Rhizobacteria Ameliorate the Biomass Characters of Panicum Maximum Jacq. by Mitigating Drought and Salt Stress. *Sci Rep* **2018**, *8*, 17513, doi:10.1038/s41598-018-35565-3.
529. Tiwari, S.; Lata, C.; Chauhan, P.S.; Nautiyal, C.S. Pseudomonas Putida Attunes Morphophysiological, Biochemical and Molecular Responses in Cicer Arietinum L. during Drought Stress and Recovery. *Plant Physiology and Biochemistry* **2016**, *99*, 108–117, doi:10.1016/j.plaphy.2015.11.001.
530. Tiwari, S.; Prasad, V.; Chauhan, P.S.; Lata, C. Bacillus Amyloliquefaciens Confers Tolerance to Various Abiotic Stresses and Modulates Plant Response to Phytohormones through Osmoprotection and Gene Expression Regulation in Rice. *Front. Plant Sci.* **2017**, *8*, 1510, doi:10.3389/fpls.2017.01510.
531. Truyens, S.; Weyens, N.; Cuypers, A.; Vangronsveld, J. Bacterial Seed Endophytes: Genera, Vertical Transmission and Interaction with Plants: Bacterial Seed Endophytes. *Environmental Microbiology Reports* **2015**, *7*, 40–50, doi:10.1111/1758-2229.12181.
532. Ullah, A.; Nisar, M.; Ali, H.; Hazrat, A.; Hayat, K.; Keerio, A.A.; Ihsan, M.; Laiq, M.; Ullah, S.; Fahad, S.; et al. Drought Tolerance Improvement in Plants: An Endophytic Bacterial Approach. *Appl Microbiol Biotechnol* **2019**, *103*, 7385–7397, doi:10.1007/s00253-019-10045-4.
533. Ullah, U.; Ashraf, M.; Shahzad, S.M.; Siddiqui, A.R.; Piracha, M.A.; Suleman, M. Growth Behavior of Tomato (Solanum Lycopersicum L.) under Drought Stress in the Presence of Silicon and Plant Growth Promoting Rhizobacteria. *Soil & Enviroment* **2016**, *35,* 65-75.
534. Upadhyay, R.K. Plant-Rhizobacteria Interaction: Physiological Implication for Heavy Metal Stress in Plants—A Review. *Israel Journal of Plant Sciences* **2011**, *59*, 249–254, doi:10.1560/IJPS.59.2-4.249.
535. Upadhyay, S.K.; Singh, G.; Singh, D.P. Mechanism and Understanding of PGPR: An Approach for Sustainable Agriculture Under Abiotic Stresses. In book: Microbes and Enviromental Management (pp. 225-254). Ed.: first, Champter 11, Publisher: Studium Press (India) Pvt. Ltd, Editors: Jay Shankar D. P. Singh.
536. Vacheron, J.; Combes-Meynet, E.; Walker, V.; Gouesnard, B.; Muller, D.; Moënne-Loccoz, Y.; Prigent-Combaret, C. Expression on Roots and Contribution to Maize Phytostimulation of 1-Aminocyclopropane-1-Decarboxylate Deaminase Gene AcdS in Pseudomonas Fluorescens F113. *Plant Soil* **2016**, *407*, 187–202, doi:10.1007/s11104-016-2907-0.
537. Vaishnav, A.; Choudhary, D.K. Regulation of Drought-Responsive Gene Expression in *Glycine Max* L. Merrill Is Mediated Through *Pseudomonas Simiae* Strain AU. *J Plant Growth Regul* **2019**, *38*, 333–342, doi:10.1007/s00344-018-9846-3.
538. Vaishnav, A.; Kasotia, A.; Choudhary, D.K. Role of Functional Bacterial Phylum Proteobacteria in Glycine Max Growth Promotion Under Abiotic Stress: A Glimpse on Case Study. In *In Silico Approach for Sustainable Agriculture*;., Eds.; Springer Singapore: Singapore, 2018; pp. 17–49 ISBN 9789811303463.
539. Vaishnav, A.; Kumari, S.; Jain, S.; Varma, A.; Choudhary, D.K. Putative Bacterial Volatile-Mediated Growth in Soybean ( *Glycine Max* L. Merrill) and Expression of Induced Proteins under Salt Stress. *J Appl Microbiol* **2015**, *119*, 539–551, doi:10.1111/jam.12866.
540. Vaishnav, A.; Shukla, A.K.; Sharma, A.; Kumar, R.; Choudhary, D.K. Endophytic Bacteria in Plant Salt Stress Tolerance: Current and Future Prospects. *J Plant Growth Regul* **2019**, *38*, 650–668, doi:10.1007/s00344-018-9880-1.
541. Valliere, J.M.; Wong, W.S.; Nevill, P.G.; Zhong, H.; Dixon, K.W. Preparing for the Worst: Utilizing Stress‐tolerant Soil Microbial Communities to Aid Ecological Restoration in the Anthropocene. *Ecological Solutions and Evidence* **2020**, *1*, doi:10.1002/2688-8319.12027.
542. van Loon, L.C. Plant Responses to Plant Growth-Promoting Rhizobacteria. *Eur J Plant Pathol* **2007**, *119*, 243–254, doi:10.1007/s10658-007-9165-1.
543. Van Oosten, M.J.; Di Stasio, E.; Cirillo, V.; Silletti, S.; Ventorino, V.; Pepe, O.; Raimondi, G.; Maggio, A. Root Inoculation with *Azotobacter Chroococcum* 76A Enhances Tomato Plants Adaptation to Salt Stress under Low N Conditions. *BMC Plant Biol* **2018**, *18*, 205, doi:10.1186/s12870-018-1411-5.
544. Van Oosten, M.J.; Pepe, O.; De Pascale, S.; Silletti, S.; Maggio, A. The Role of Biostimulants and Bioeffectors as Alleviators of Abiotic Stress in Crop Plants. *Chem. Biol. Technol. Agric.* **2017**, *4*, 5, doi:10.1186/s40538-017-0089-5.
545. Vandana, U.K.; Singha, B.; Gulzar, A.B.M.; Mazumder, P.B. Molecular Mechanisms in Plant Growth Promoting Bacteria (PGPR) to Resist Environmental Stress in Plants. In *Molecular Aspects of Plant Beneficial Microbes in Agriculture*; Elsevier, 2020; pp. 221–233 ISBN 978-0-12-818469-1.
546. Vanderstraeten, L.; Van Der Straeten, D. Accumulation and Transport of 1-Aminocyclopropane-1-Carboxylic Acid (ACC) in Plants: Current Status, Considerations for Future Research and Agronomic Applications. *Front. Plant Sci.* **2017**, *8*, 38, doi:10.3389/fpls.2017.00038.
547. Varma, A., Tripathi, S., Prasad, R. *Plant Microbe Symbiosis*; Eds.; Springer International Publishing: Cham, 2020; ISBN 978-3-030-36247-8.
548. Varma, A.; Tripathi, S., Prasad, R., *Plant Biotic Interactions: State of the Art*. Eds.; Springer International Publishing: Cham, 2019; ISBN 978-3-030-26656-1.
549. Vega-Celedón, P.; Bravo, G.; Velásquez, A.; Cid, F.P.; Valenzuela, M.; Ramírez, I.; Vasconez, I.-N.; Álvarez, I.; Jorquera, M.A.; Seeger, M. Microbial Diversity of Psychrotolerant Bacteria Isolated from Wild Flora of Andes Mountains and Patagonia of Chile towards the Selection of Plant Growth-Promoting Bacterial Consortia to Alleviate Cold Stress in Plants. *Microorganisms* **2021**, *9*, 538, doi:10.3390/microorganisms9030538.
550. Verma, H.; Kumar, D.; Kumar, V.; Kumari, M.; Singh, S.K.; Sharma, V.K.; Droby, S.; Santoyo, G.; White, J.F.; Kumar, A. The Potential Application of Endophytes in Management of Stress from Drought and Salinity in Crop Plants. *Microorganisms* **2021**, *9*, 1729, doi:10.3390/microorganisms9081729.
551. Verma, K.K.; Song, X.-P.; Li, D.-M.; Singh, M.; Rajput, V.D.; Malviya, M.K.; Minkina, T.; Singh, R.K.; Singh, P.; Li, Y.-R. Interactive Role of Silicon and Plant–Rhizobacteria Mitigating Abiotic Stresses: A New Approach for Sustainable Agriculture and Climate Change. *Plants* **2020**, *9*, 1055, doi:10.3390/plants9091055.
552. Verma, P.; Saxena, R.; Tomar, R.S. Rhizobacteria: A Promising Tool for Drought Tolerance in Crop Plants. *Internationa Journal of Pahrma and Bio Science (int-BIONANO-2016)*, 116-125.
553. Verma, S.K., White, Jr, J.F.,*.Seed Endophytes: Biology and Biotechnology*; Eds.; Springer International Publishing: Cham, 2019; ISBN 978-3-030-10503-7.
554. Vimal, S.R.; Patel, V.K.; Singh, J.S. Plant Growth Promoting Curtobacterium Albidum Strain SRV4: An Agriculturally Important Microbe to Alleviate Salinity Stress in Paddy Plants. *Ecological Indicators* **2019**, *105*, 553–562, doi:10.1016/j.ecolind.2018.05.014.
555. Viterbo, A.; Landau, U.; Kim, S.; Chernin, L.; Chet, I. Characterization of ACC Deaminase from the Biocontrol and Plant Growth-Promoting Agent Trichoderma Asperellum T203: ACC Deaminase Characterization in Trichoderma. *FEMS Microbiology Letters* **2010**, *305*, 42–48, doi:10.1111/j.1574-6968.2010.01910.x.
556. Vurukonda, S.S.K.P.; Vardharajula, S.; Shrivastava, M.; SkZ, A. Enhancement of Drought Stress Tolerance in Crops by Plant Growth Promoting Rhizobacteria. *Microbiological Research* **2016**, *184*, 13–24, doi:10.1016/j.micres.2015.12.003.
557. Wang, C.; Knill, E.; Glick, B.R.; Défago, G. Effect of Transferring 1-Aminocyclopropane-1- Carboxylic Acid (ACC) Deaminase Genes into Pseudomonas Fluorescens Strain CHA0 and Its GacA Derivative CHA96 on Their Growth-Promoting and Disease-Suppressive Capacities. *Can J Microbiol.* **2000**, *46*, 698-907, doi:10.1139/w00-071.
558. Wang, G.; Li, B.; Peng, D.; Zhao, H.; Lu, M.; Zhang, L.; Li, J.; Zhang, S.; Guan, C.; Ji, J. Combined Application of H2S and a Plant Growth Promoting Strain JIL321 Regulates Photosynthetic Efficacy, Soil Enzyme Activity and Growth-Promotion in Rice under Salt Stress. *Microbiological Research* **2022**, *256*, 126943, doi:10.1016/j.micres.2021.126943.
559. Wang, K.L.-C.; Li, H.; Ecker, J.R. Ethylene Biosynthesis and Signaling Networks. *Plant Cell* **2002**, *14*, S131–S151, doi:10.1105/tpc.001768.
560. Wang, N.; Chen, H.; Nonaka, S.; Sato-Izawa, K.; Kusano, M.; Ezura, H. Ethylene Biosynthesis Controlled by NON-RIPENING: A Regulatory Conflict between Wounding and Ripening. *Plant Physiology and Biochemistry* **2018**, *132*, 720–726, doi:10.1016/j.plaphy.2018.07.034.
561. Wang, Q.; Dodd, I.C.; Belimov, A.A.; Jiang, F. Rhizosphere Bacteria Containing 1-Aminocyclopropane-1- Carboxylate Deaminase Increase Growth and Photosynthesis of Pea Plants under Salt Stress by Limiting Na+ Accumulation. *Functional Plant Biol.* **2016**, *43*, 161, doi:10.1071/FP15200.
562. Wang, Z.; Solanki, M.K.; Yu, Z.-X.; Yang, L.-T.; An, Q.-L.; Dong, D.-F.; Li, Y.-R. Draft Genome Analysis Offers Insights Into the Mechanism by Which Streptomyces Chartreusis WZS021 Increases Drought Tolerance in Sugarcane. *Front. Microbiol.* **2019**, *9*, 3262, doi:10.3389/fmicb.2018.03262.
563. Wdowiak-Wróbel, S.; Małek, W. Properties of Astragalus Sp. Microsymbionts and Their Putative Role in Plant Growth Promotion. *Arch Microbiol* **2016**, *198*, 793–801, doi:10.1007/s00203-016-1243-3.
564. Wi, S.J.; Kim, W.T.; Park, K.Y. Overexpression of Carnation S-Adenosylmethionine Decarboxylase Gene Generates a Broad-Spectrum Tolerance to Abiotic Stresses in Transgenic Tobacco Plants. *Plant Cell Rep* **2006**, *25*, 1111–1121, doi:10.1007/s00299-006-0160-3.
565. Win, K.T.; Tanaka, F.; Okazaki, K.; Ohwaki, Y. The ACC Deaminase Expressing Endophyte Pseudomonas Spp. Enhances NaCl Stress Tolerance by Reducing Stress-Related Ethylene Production, Resulting in Improved Growth, Photosynthetic Performance, and Ionic Balance in Tomato Plants. *Plant Physiology and Biochemistry* **2018**, *127*, 599–607, doi:10.1016/j.plaphy.2018.04.038.
566. Wong, C.E.; Li, Y.; Labbe, A.; Guevara, D.; Nuin, P.; Whitty, B.; Diaz, C.; Golding, G.B.; Gray, G.R.; Weretilnyk, E.A.; et al. Transcriptional Profiling Implicates Novel Interactions between Abiotic Stress and Hormonal Responses in Thellungiella, a Close Relative of Arabidopsis. *Plant Physiology* **2006**, *140*, 1437–1450, doi:10.1104/pp.105.070508.
567. Woo, O.-G.; Kim, H.; Kim, J.-S.; Keum, H.L.; Lee, K.-C.; Sul, W.J.; Lee, J.-H. *Bacillus Subtilis* Strain GOT9 Confers Enhanced Tolerance to Drought and Salt Stresses in *Arabidopsis* *Thaliana* and *Brassica Campestris*. *Plant Physiology and Biochemistry* **2020**, *148*, 359–367, doi:10.1016/j.plaphy.2020.01.032.
568. Xu, M.; Sheng, J.; Chen, L.; Men, Y.; Gan, L.; Guo, S.; Shen, L. Bacterial Community Compositions of Tomato (Lycopersicum Esculentum Mill.) Seeds and Plant Growth Promoting Activity of ACC Deaminase Producing Bacillus Subtilis (HYT-12-1) on Tomato Seedlings. *World J Microbiol Biotechnol* **2014**, *30*, 835–845, doi:10.1007/s11274-013-1486-y.
569. Yadav, A.N. *Soil Microbiomes for Sustainable Agriculture: Functional Annotation*; Ed.; Sustainable Development and Biodiversity; Springer International Publishing: Cham, 2021; Vol. 27; ISBN 978-3-030-73506-7.
570. Yadav, A.N., Rastegari, A.A., Yadav, N., Kour, D. *Advances in Plant Microbiome and Sustainable Agriculture: Functional Annotation and Future Challenges*; Eds.; Microorganisms for Sustainability; Springer Singapore: Singapore, 2020; Vol. 20; ISBN 9789811532030.
571. Yadav, V.K.; Raghav, M., Sharma, S. K.; Bhagat, N. Rhizobacteriome: Promising Candidate for Conferring Drought Tolerance in Crops. *J. Pure Appl. Microbiol.* **2020**, *14*, 73–92, doi:10.22207/JPAM.14.1.10.
572. Yaish, M.W.; Antony, I.; Glick, B.R. Isolation and Characterization of Endophytic Plant Growth-Promoting Bacteria from Date Palm Tree (Phoenix Dactylifera L.) and Their Potential Role in Salinity Tolerance. *Antonie van Leeuwenhoek* **2015**, *107*, 1519–1532, doi:10.1007/s10482-015-0445-z.
573. Yan, J.; Smith, M.D.; Glick, B.R.; Liang, Y. Effects of ACC Deaminase Containing Rhizobacteria on Plant Growth and Expression of Toc GTPases in Tomato ( *Solanum Lycopersicum* ) under Salt Stress. *Botany* **2014**, *92*, 775–781, doi:10.1139/cjb-2014-0038.
574. Yankey, R.; Omoor, I.N.A.; Karanja, J.K.; Wang, L.; Urga, R.T.; Fang, C.H.; Dongmei, L.; Lin, H.; Okal, J.E.; Datti, I.L.; et al. Metabolic Properties, Gene Functions, and Biosafety Analysis Reveal the Action of Three Rhizospheric Plant Growth-Promoting Bacteria of Jujuncao (Pennisetum Giganteum). *Environ Sci Pollut Res* **2022**, *29*, 38435-38449, doi:10.1007/s11356-021-17854-z.
575. Yasin, N.A.; Akram, W.; Khan, W.U.; Ahmad, S.R.; Ahmad, A.; Ali, A. Halotolerant Plant-Growth Promoting Rhizobacteria Modulate Gene Expression and Osmolyte Production to Improve Salinity Tolerance and Growth in *Capsicum Annum* L. *Environ Sci Pollut Res* **2018**, *25*, 23236–23250, doi:10.1007/s11356-018-2381-8.
576. Yasmeen, T.; Ahmad, A.; Arif, M.S.; Mubin, M.; Rehman, K.; Shahzad, S.M.; Iqbal, S.; Rizwan, M.; Ali, S.; Alyemeni, M.N.; et al. Biofilm Forming Rhizobacteria Enhance Growth and Salt Tolerance in Sunflower Plants by Stimulating Antioxidant Enzymes Activity. *Plant Physiology and Biochemistry* **2020**, *156*, 242–256, doi:10.1016/j.plaphy.2020.09.016.
577. Yasmin, H.; Bano, A.; Wilson, N.L.; Nosheen, A.; Naz, R.; Hassan, M.N.; Ilyas, N.; Saleem, M.H.; Noureldeen, A.; Ahmad, P.; et al. Drought‐tolerant *Pseudomonas* Sp. Showed Differential Expression of Stress‐responsive Genes and Induced Drought Tolerance in *Arabidopsis thaliana* . *Physiologia Plantarum* **2022**, *174*, doi:10.1111/ppl.13497.
578. Yasmin, H.; Naeem, S.; Bakhtawar, M.; Jabeen, Z.; Nosheen, A.; Naz, R.; Keyani, R.; Mumtaz, S.; Hassan, M.N. Halotolerant Rhizobacteria Pseudomonas Pseudoalcaligenes and Bacillus Subtilis Mediate Systemic Tolerance in Hydroponically Grown Soybean (Glycine Max L.) against Salinity Stress. *PLoS ONE* **2020**, *15*, e0231348, doi:10.1371/journal.pone.0231348.
579. Yim, W.-J.; Woo, S.-M.; Kim, K.-Y.; Sa, T.-M. Regulation of Ethylene Emission in Tomato (Lycopersicon Esculentum Mill.) and Red Pepper (Capsicum Annuum L.) Inoculated with ACC Deaminase Producing Methylobacterium Spp. *Korean Journal of Soil Science and Fertilizer* **2012**, *45*, 37–42, doi:10.7745/KJSSF.2012.45.1.037.
580. Yoolong, S.; Kruasuwan, W.; Thanh Phạm, H.T.; Jaemsaeng, R.; Jantasuriyarat, C.; Thamchaipenet, A. Modulation of Salt Tolerance in Thai Jasmine Rice (*Oryza sativa* L. cv. KDML105) by *Streptomyces Venezuelae* ATCC 10712 Expressing ACC Deaminase. *Sci Rep* **2019**, *9*, 1275, doi:10.1038/s41598-018-37987-5.
581. Yuan, Y.; Zu, M.; Sun, L.; Zuo, J.; Tao, J. Isolation and Screening of 1-Aminocyclopropane-1-Carboxylic Acid (ACC) Deaminase Producing PGPR from Paeonia Lactiflora Rhizosphere and Enhancement of Plant Growth. *Scientia Horticulturae* **2022**, *297*, 110956, doi:10.1016/j.scienta.2022.110956.
582. Yue; Shen; Chen; Liang; Chu; Chen; Sun Microbiological Insights into the Stress-Alleviating Property of an Endophytic Bacillus Altitudinis WR10 in Wheat under Low-Phosphorus and High-Salinity Stresses. *Microorganisms* **2019**, *7*, 508, doi:10.3390/microorganisms7110508.
583. Zafar-ul-Hye, M.; Danish, S.; Abbas, M.; Ahmad, M.; Munir, T.M. ACC Deaminase Producing PGPR Bacillus Amyloliquefaciens and Agrobacterium Fabrum along with Biochar Improve Wheat Productivity under Drought Stress. *Agronomy* **2019**, *9*, 343, doi:10.3390/agronomy9070343.
584. Zafar-Ul-Hye, M.; Shahjahan, A.; Danish, S.; Abid, M.; Qayyum, M.F. MITIGATION OF CADMIUM TOXICITY INDUCED STRESS IN WHEAT BY ACC-DEAMINASE CONTAINING PGPR ISOLATED FROM CADMIUM POLLUTED WHEAT RHIZOSPHERE. *Pak. J. Bot* **2018**, *50*, 1727-1734.
585. Zafar-ul-Hye, M.; Tahzeeb-ul-Hassan, M.; Wahid, A.; Danish, S.; Khan, M.J.; Fahad, S.; Brtnicky, M.; Hussain, G.S.; Battaglia, M.L.; Datta, R. Compost Mixed Fruits and Vegetable Waste Biochar with ACC Deaminase Rhizobacteria Can Minimize Lead Stress in Mint Plants. *Sci Rep* **2021**, *11*, 6606, doi:10.1038/s41598-021-86082-9.
586. Zahedi, H. Toward the Mitigation of Biotic and Abiotic Stresses through Plant Growth Promoting Rhizobacteria. In *Advances in Organic Farming*; Elsevier, **2021**; pp. 161–172 ISBN 978-0-12-822358-1.
587. Zaidi, A., Khan, M.S. *Microbial Strategies for Vegetable Production,* Eds.; Springer International Publishing: Cham, 2017; ISBN 978-3-319-54400-7.
588. Zainab, N.; Amna; Din, B.U.; Javed, M.T.; Afridi, M.S.; Mukhtar, T.; Kamran, M.A.; Qurat ul ain; Khan, A.A.; Ali, J.; et al. Deciphering Metal Toxicity Responses of Flax (Linum Usitatissimum L.) with Exopolysaccharide and ACC-Deaminase Producing Bacteria in Industrially Contaminated Soils. *Plant Physiology and Biochemistry* **2020**, *152*, 90–99, doi:10.1016/j.plaphy.2020.04.039.
589. Zerrouk, I.Z.; Rahmoune, B.; Khelifi, L.; Mounir, K.; Baluska, F.; Ludwig-Müller, J. Algerian Sahara PGPR Confers Maize Root Tolerance to Salt and Aluminum Toxicity via ACC Deaminase and IAA. *Acta Physiol Plant* **2019**, *41*, 91, doi:10.1007/s11738-019-2881-2.
590. Zhang, F.; Zhang, J.; Chen, L.; Shi, X.; Lui, Z.; Li, C. Heterologous Expression of ACC Deaminase from Trichoderma Asperellum Improves the Growth Performance of Arabidopsis Thaliana under Normal and Salt Stress Conditions. *Plant Physiology and Biochemistry* **2015**, *94*, 41–47, doi:10.1016/j.plaphy.2015.05.007.
591. Zhang, G.; Sun, Y.; Sheng, H.; Li, H.; Liu, X. Effects of the Inoculations Using Bacteria Producing ACC Deaminase on Ethylene Metabolism and Growth of Wheat Grown under Different Soil Water Contents. *Plant Physiology and Biochemistry* **2018**, *125*, 178–184, doi:10.1016/j.plaphy.2018.02.005.
592. Zhang, H.; Sun, X.; Dai, M. Improving Crop Drought Resistance with Plant Growth Regulators and Rhizobacteria: Mechanisms, Applications, and Perspectives. *Plant Communications* **2022**, *3*, 100228, doi:10.1016/j.xplc.2021.100228.
593. Zhang, M.; Yang, L.; Hao, R.; Bai, X.; Wang, Y.; Yu, X. Drought-Tolerant Plant Growth-Promoting Rhizobacteria Isolated from Jujube (*Ziziphus Jujuba*) and Their Potential to Enhance Drought Tolerance. *Plant Soil* **2020**, *452*, 423–440, doi:10.1007/s11104-020-04582-5.
594. Zhang, S., Gan, Y., & Xu, B. Mechanisms of the IAA and ACC-deaminase producing strain of Trichoderma longibrachiatum T6 in enhancing wheat seedling tolerance to NaCl stress. *BMC plant biology* **2019**, *19*(1), 1-18.
595. Zhang, S.; Gan, Y.; Xu, B. Application of Plant-Growth-Promoting Fungi Trichoderma Longibrachiatum T6 Enhances Tolerance of Wheat to Salt Stress through Improvement of Antioxidative Defense System and Gene Expression. *Front. Plant Sci.* **2016**, *7*, 1405, doi:10.3389/fpls.2016.01405.
596. Zhang, Y.; He, L.; Chen, Z.; Wang, Q.; Qian, M.; Sheng, X. Characterization of ACC Deaminase-Producing Endophytic Bacteria Isolated from Copper-Tolerant Plants and Their Potential in Promoting the Growth and Copper Accumulation of Brassica Napus. *Chemosphere* **2011**, *83*, 57–62, doi:10.1016/j.chemosphere.2011.01.041.
597. Zhou, J.; Ahmed, N.; Cheng, Y.; Qin, C.; Chen, P.; Zhang, C.; Zhang, L. Effect of Inoculation of Strains with ACC Deaminase Isolated from Vermicompost on Seed Germination and Some Physiological Attributes in Maize (Zea Mays L.) Exposed to Salt Stress. *PAK.J.BOT.* **2019**, *51*, 1169-1177, doi:10.30848/PJB2019-4(34).
598. Zia, R.; Nawaz, M.S.; Siddique, M.J.; Hakim, S.; Imran, A. Plant Survival under Drought Stress: Implications, Adaptive Responses, and Integrated Rhizosphere Management Strategy for Stress Mitigation. *Microbiological Research* **2021**, *242*, 126626, doi:10.1016/j.micres.2020.126626.
599. Zia, R.; Nawaz, M.S.; Yousaf, S.; Amin, I.; Hakim, S.; Mirza, M.S.; Imran, A. Seed Inoculation of desert‐ Plant Growth‐promoting Rhizobacteria Induce Biochemical Alterations and Develop Resistance against Water Stress in Wheat. *Physiologia Plantarum* **2021**, *172*, 990–1006, doi:10.1111/ppl.13362.
600. Zolla, G.; Badri, D.V.; Bakker, M.G.; Manter, D.K.; Vivanco, J.M. Soil Microbiomes Vary in Their Ability to Confer Drought Tolerance to Arabidopsis. *Applied Soil Ecology* **2013**, *68*, 1–9, doi:10.1016/j.apsoil.2013.03.007.
601. Zubair; Hanif; Farzand; Sheikh; Khan; Suleman; Ayaz; Gao Genetic Screening and Expression Analysis of Psychrophilic *Bacillus* Spp. Reveal Their Potential to Alleviate Cold Stress and Modulate Phytohormones in Wheat. *Microorganisms* **2019**, *7*, 337, doi:10.3390/microorganisms7090337.

***1-aminocyclopropane-1-carboxylic acid oxidase (ACO)***

1. AbdElgawad, H.; El-Sawah, A.M.; Mohammed, A.E.; Alotaibi, M.O.; Yehia, R.S.; Selim, S.; Saleh, A.M.; Beemster, G.T.S.; Sheteiwy, M.S. Increasing Atmospheric CO_2_ Differentially Supports Arsenite Stress Mitigating Impact of Arbuscular Mycorrhizal Fungi in Wheat and Soybean Plants. *Chemosphere* **2022**, *296*, 134044, doi:10.1016/j.chemosphere.2022.134044.
2. Abdelsamad, N. Factors Affecting Expression of Soybean Sudden Death Syndrome: Flooding, Oxygen Level, and Ethylene Hormone. Doctor of Philosophy, Iowa State University, Digital Repository: Ames, 2016, p. 11169146.
3. Adak, M. K.; Saha, I.; Dolui, D.; Hasanuzzaman, M. An Updated Overview of the Physiological and Molecular Responses of Rice to Anoxia. *Front Biosci (Landmark Ed)* **2021**, *26*, 1240-1255, doi:10.52586/5021.
4. Adeleke, B.; Ayangbenro, A.; Babalola, O. Genomic Analysis of Endophytic Bacillus Cereus T4S and Its Plant Growth-Promoting Traits. *Plants* **2021**, *10*, 1776, doi:10.3390/plants10091776.
5. *Advances in Plant Microbiome and Sustainable Agriculture: Functional Annotation and Future Challenges*; Yadav, A.N., Rastegari, A.A., Yadav, N., Kour, D., Eds.; Microorganisms for Sustainability; Springer Singapore: Singapore, 2020; Vol. 20; ISBN 9789811532030.
6. Agaras, B.C.; Iriarte, A.; Valverde, C.F. Genomic Insights into the Broad Antifungal Activity, Plant-Probiotic Properties, and Their Regulation, in Pseudomonas Donghuensis Strain SVBP6. *PLoS ONE* **2018**, *13*, e0194088, doi:10.1371/journal.pone.0194088.
7. Ahammed, G.J., Yu, J.-Q. *Plant Hormones under Challenging Environmental Factors*; Eds.; Springer Netherlands: Dordrecht, 2016; ISBN 978-94-017-7756-8.
8. Ajeng, A.A.; Abdullah, R.; Ling, T.C.; Ismail, S.; Lau, B.F.; Ong, H.C.; Chew, K.W.; Show, P.L.; Chang, J.-S. Bioformulation of Biochar as a Potential Inoculant Carrier for Sustainable Agriculture. *Environmental Technology & Innovation* **2020**, *20*, 101168, doi:10.1016/j.eti.2020.101168.
9. Alberton, D.; Valdameri, G.; Moure, V.R.; Monteiro, R.A.; Pedrosa, F. de O.; Müller-Santos, M.; de Souza, E.M. What Did We Learn From Plant Growth-Promoting Rhizobacteria (PGPR)-Grass Associations Studies Through Proteomic and Metabolomic Approaches? *Front. Sustain. Food Syst.* **2020**, *4*, 607343, doi:10.3389/fsufs.2020.607343.
10. Ali, S.; Kim, W.-C. Plant Growth Promotion Under Water: Decrease of Waterlogging-Induced ACC and Ethylene Levels by ACC Deaminase-Producing Bacteria. *Front. Microbiol.* **2018**, *9*, 1096, doi:10.3389/fmicb.2018.01096.
11. Andrés-Barrao, C.; Alzubaidy, H.; Jalal, R.; Mariappan, K.G.; de Zélicourt, A.; Bokhari, A.; Artyukh, O.; Alwutayd, K.; Rawat, A.; Shekhawat, K.; et al. Coordinated Bacterial and Plant Sulfur Metabolism in *Enterobacter* Sp. SA187–Induced Plant Salt Stress Tolerance. *Proc. Natl. Acad. Sci. U.S.A.* **2021**, *118*, e2107417118, doi:10.1073/pnas.2107417118.
12. Ayaz, M.; Ali, Q.; Farzand, A.; Khan, A.R.; Ling, H.; Gao, X. Nematicidal Volatiles from *Bacillus Atrophaeus* GBSC56 Promote Growth and Stimulate Induced Systemic Resistance in Tomato against Meloidogyne Incognita. *IJMS* **2021**, *22*, 5049, doi:10.3390/ijms22095049.
13. Backer, R.; Rokem, J.S.; Ilangumaran, G.; Lamont, J.; Praslickova, D.; Ricci, E.; Subramanian, S.; Smith, D.L. Plant Growth-Promoting Rhizobacteria: Context, Mechanisms of Action, and Roadmap to Commercialization of Biostimulants for Sustainable Agriculture. *Front. Plant Sci.* **2018**, *9*, 1473, doi:10.3389/fpls.2018.01473.
14. Bal, H.B.; Adhya, T.K. Alleviation of Submergence Stress in Rice Seedlings by Plant Growth-Promoting Rhizobacteria With ACC Deaminase Activity. *Front. Sustain. Food Syst.* **2021**, *5*, 606158, doi:10.3389/fsufs.2021.606158.
15. Bao, Y.; Zhang, X.; Sun, X.; Bao, M.; Wang, Y. Morphological and Molecular Analyses of the Interaction between *Rosa Multiflora* and *Podosphaera Pannosa*. *Genes* **2022**, *13*, 1003, doi:10.3390/genes13061003.
16. Barickman, T.C.; Adhikari, B.; Sehgal, A.; Walne, C.H.; Reddy, K.R.; Gao, W. Drought and Elevated CO2 Impacts Photosynthesis and Biochemicals of Basil (*Ocimum Basilicum* L.). *Stresses* **2021**, *1*, 223–237, doi:10.3390/stresses1040016.
17. Bellido, E.; de la Haba, P.; Agüera, E. Physiological Alteration in Sunflower Plants (*Helianthus Annuus* L.) Exposed to High CO_2_ and Arbuscular Mycorrhizal Fungi. *Plants* **2021**, *10*, 937, doi:10.3390/plants10050937.
18. Bhanse, P.; Kumar, M.; Singh, L., Kumar Awasthi, M.; Qureshi, A.; Role of plant growth-promoting rhizobacteria in boosting the phytoremediation of stressed soils: Opportunities, challenges, and prospects. *Chemosphere* **2022**, Volume 303, Part 1, 134954, doi:10.1016/j.chemosphere.2022.134954.
19. Bharti, N.; Barnawal, D. Amelioration of Salinity Stress by PGPR. In *PGPR Amelioration in Sustainable Agriculture*; Elsevier, 2019; pp. 85–106 ISBN 978-0-12-815879-1.
20. Bisht, N.; Tiwari, S.; Singh, P.C.; Niranjan, A.; Singh Chauhan, P. A Multifaceted Rhizobacterium Paenibacillus Lentimorbus Alleviates Nutrient Deficiency-Induced Stress in Cicer Arietinum L. *Microbiological Research* **2019**, *223–225*, 110–119, doi:10.1016/j.micres.2019.04.007.
21. Bomle, D.V.; Kiran, A.; Kumar, J.K.; Nagaraj, L.S.; Pradeep, C.K.; Ansari, M.A.; Alghamdi, S.; Kabrah, A.; Assaggaf, H.; Dablool, A.S.; et al. Plants Saline Environment in Perception with Rhizosphere Bacteria Containing 1-Aminocyclopropane-1-Carboxylate Deaminase. *IJMS* **2021**, *22*, 11461, doi:10.3390/ijms222111461.
22. Borbély, P.; Poór, P.; Tari, I. Changes in Physiological and Photosynthetic Parameters in Tomato of Different Ethylene Status under Salt Stress: Effects of Exogenous 1-Aminocyclopropane-1-Carboxylic Acid Treatment and the Inhibition of Ethylene Signalling. *Plant Physiology and Biochemistry* **2020**, *156*, 345–356, doi:10.1016/j.plaphy.2020.09.019.
23. Brunetti, C.; Saleem, A.R.; Della Rocca, G.; Emiliani, G.; De Carlo, A.; Balestrini, R.; Khalid, A.; Mahmood, T.; Centritto, M. Effects of Plant Growth-Promoting Rhizobacteria Strains Producing ACC Deaminase on Photosynthesis, Isoprene Emission, Ethylene Formation and Growth of *Mucuna Pruriens* (L.) DC. in Response to Water Deficit. *Journal of Biotechnology* **2021**, *331*, 53–62, doi:10.1016/j.jbiotec.2021.03.008.
24. Cabanás, C.G.-L.; Sesmero, R.; Valverde-Corredor, A.; Javier López-Escudero, F.; Mercado-Blanco, J. A Split-Root System to Assess Biocontrol Effectiveness and Defense-Related Genetic Responses in above-Ground Tissues during the Tripartite Interaction Verticillium Dahliae-Olive-Pseudomonas Fluorescens PICF7 in Roots. *Plant Soil* **2017**, *417*, 433–452, doi:10.1007/s11104-017-3269-y.
25. Çakır, B.; Gül, A.; Yolageldi, L.; Özaktan, H. Response to Fusarium Oxysporum f.Sp. Radicis-Lycopersici in Tomato Roots Involves Regulation of SA- and ET-Responsive Gene Expressions. *Eur J Plant Pathol* **2014**, *139*, 379–391, doi:10.1007/s10658-014-0394-9.
26. Canellas, L.P.; Olivares, F.L. Physiological Responses to Humic Substances as Plant Growth Promoter. *Chem Biol Techn Agric* **2014**, *1*, 3, doi:10.1186/2196-5641-1-3.
27. Chatterjee, P.; Kanagendran, A.; Samaddar, S.; Pazouki, L.; Sa, T.-M.; Niinemets, Ü. Inoculation of Brevibacterium Linens RS16 in Oryza Sativa Genotypes Enhanced Salinity Resistance: Impacts on Photosynthetic Traits and Foliar Volatile Emissions. *Science of The Total Environment* **2018**, *645*, 721–732, doi:10.1016/j.scitotenv.2018.07.187.
28. Chavan, S.; Nadanathangam, V. Effects of Nanoparticles on Plant Growth-Promoting Bacteria in Indian Agricultural Soil. *Agronomy* **2019**, *9*, 140, doi:10.3390/agronomy9030140.
29. Chen, W.; Tang, L.; Wang, J.; Zhu, H.; Jin, J.; Yang, J.; Fan, W. Research Advances in the Mutual Mechanisms Regulating Response of Plant Roots to Phosphate Deficiency and Aluminum Toxicity. *IJMS* **2022**, *23*, 1137, doi:10.3390/ijms23031137.
30. Chen, X.; Sun, M.; Chong, S.; Si, J.; Wu, L. Transcriptomic and Metabolomic Approaches Deepen Our Knowledge of Plant–Endophyte Interactions. *Front. Plant Sci.* **2022**, *12*, 700200, doi:10.3389/fpls.2021.700200.
31. Choudhary, P.; Pramitha, L.; Rana, S.; Verma, S.; Aggarwal, P.R.; Muthamilarasan, M. Hormonal Crosstalk in Regulating Salinity Stress Tolerance in Graminaceous Crops. *Physiologia Plantarum* **2021**, *173*, 1587–1596, doi:10.1111/ppl.13558.
32. Conrath, U. Chapter 9 Priming of Induced Plant Defense Responses. In *Advances in Botanical Research*; Elsevier, 2009; Vol. 51, pp. 361–395 ISBN 978-0-12-374834-8.
33. Contesto, C.; Desbrosses, G.; Lefoulon, C.; Béna, G.; Borel, F.; Galland, M.; Gamet, L.; Varoquaux, F.; Touraine, B. Effects of Rhizobacterial ACC Deaminase Activity on Arabidopsis Indicate That Ethylene Mediates Local Root Responses to Plant Growth-Promoting Rhizobacteria. *Plant Science* **2008**, *175*, 178–189, doi:10.1016/j.plantsci.2008.01.020.
34. Cook, J.; Degon, Z.; Ruiz, D.; Pope, J.; Rahmatallah, Y.; Mukherjee, A. The Plant Growth-Promoting Bacteria, *Azospirillum Brasilense*, Induce a Diverse Array of Genes in Rice Shoots and Promote Their Growth. *Plant Growth Regul* **2022**, *97*, 143–155, doi:10.1007/s10725-022-00813-0.
35. Correa-García, S.; Pande, P.; Séguin, A.; St-Arnaud, M.; Yergeau, E. Rhizoremediation of Petroleum Hydrocarbons: A Model System for Plant Microbiome Manipulation. *Microb. Biotechnol.* **2018**, *11*, 819–832, doi:10.1111/1751-7915.13303.
36. Darriaut, R.; Lailheugue, V.; Masneuf-Pomarède, I.; Marguerit, E.; Martins, G.; Compant, S.; Ballestra, P.; Upton, S.; Ollat, N.; Lauvergeat, V. Grapevine Rootstock and Soil Microbiome Interactions: Keys for a Resilient Viticulture. *Horticulture Research* **2022**, *9*, uhac019, doi:10.1093/hr/uhac019.
37. De La Torre-Ruiz, N.; Ruiz-Valdiviezo, V.M.; Rincón-Molina, C.I.; Rodríguez-Mendiola, M.; Arias-Castro, C.; Gutiérrez-Miceli, F.A.; Palomeque-Dominguez, H.; Rincón-Rosales, R. Effect of Plant Growth-Promoting Bacteria on the Growth and Fructan Production of *Agave Americana* L. *Brazilian Journal of Microbiology* **2016**, *47*, 587–596, doi:10.1016/j.bjm.2016.04.010.
38. Debouza, N.E.; Babu Thruppoyil, S.; Gopi, K.; Zain, S.; Ksiksi, T. Plant and Seed Germination Responses to Global Change, with a Focus on CO2: A Review. *OE* **2021**, *6*, e74260, doi:10.3897/oneeco.6.e74260.
39. Dhanushkodi, R. Drought Stress Responses of the Medicago Truncatula - Ensifer Meliloti Symbiosis on Nodule Senescence and Nitrogen Fixation. Doctoral dissertation 2018.
40. Dimopoulou, A.; Theologidis, I.; Liebmann, B.; Kalantidis, K.; Vassilakos, N.; Skandalis, N. *Bacillus Amyloliquefaciens* MBI600 Differentially Induces Tomato Defense Signaling Pathways Depending on Plant Part and Dose of Application. *Sci Rep* **2019**, *9*, 19120, doi:10.1038/s41598-019-55645-2.
41. Dixit, R.; Agrawal, L.; Gupta, S.; Kumar, M.; Yadav, S.; Chauhan, P.S.; Nautiyal, C.S. Southern Blight Disease of Tomato Control by 1-Aminocyclopropane-1-Carboxylate (ACC) Deaminase Producing *Paenibacillus Lentimorbus* B-30488. *Plant Signaling & Behavior* **2016**, *11*, e1113363, doi:10.1080/15592324.2015.1113363.
42. Du, G.; Wang, L.; Li, H.; Sun, P.; Fu, J.; Suo, Y.; Han, W.; Diao, S.; Mai, Y.; Li, F. Selection and Validation of Reference Genes for Quantitative Gene Expression Analyses in Persimmon (*Diospyros Kaki* Thunb.) Using Real-Time Quantitative PCR. *BioFut* **2020**, *70*, 261–267, doi:10.1556/019.70.2019.24.
43. Du, N.; Shi, L.; Yuan, Y.; Li, B.; Shu, S.; Sun, J.; Guo, S. Proteomic Analysis Reveals the Positive Roles of the Plant-Growth-Promoting Rhizobacterium NSY50 in the Response of Cucumber Roots to *Fusarium oxysporum* f. Sp. Cucumerinum Inoculation. *Front. Plant Sci.* **2016**, *7*, doi:10.3389/fpls.2016.01859.
44. Dubey, S.K., Verma, S.K., *Plant, Soil and Microbes in Tropical Ecosystems*; Eds.; Rhizosphere Biology; Springer Singapore: Singapore, 2021; ISBN 9789811633638.
45. Dunwell, J.M.; Gibbings, J.G.; Mahmood, T.; Saqlan Naqvi, S.M. Germin and Germin-like Proteins: Evolution, Structure, and Function. *Critical Reviews in Plant Sciences* **2008**, *27*, 342–375, doi:10.1080/07352680802333938.
46. El-Awadi, M.E.; Dawood, M.G.; Abdel-Baky, Y.R.; Hassan, E.A. Physiological Effect of Melatonin, IAA and Their Precursor on Quality and Quantity of Chickpea Plants Grown under Sandy Soil Conditions. **2017**, 10.
47. Farahat, M.G.; Mahmoud, M.K.; Youseif, S.H.; Saleh, S.A.; Kamel, Z. Alleviation of Salinity Stress in Wheat by ACC Deaminase-Producing *Bacillus aryabhattai* EWR29 with Multifarious Plant Growth-Promoting Attributes. *Plant Archieves* **2020,** *20,* 417-429.
48. Fazal, A.; Ur, U. R.; Abdul. R., Nisar, A.; Mohammad, A.; Akhtar, R. The Role o Phytohormones in Combating Biotic Stress. In: *Plant Growth Regulators for Climate.Smart Agricolture* **2021**, 187-206.
49. Feki, K.; Tounsi, S.; Mrabet, M.; Mhadhbi, H.; Brini, F. Recent Advances in Physiological and Molecular Mechanisms of Heavy Metal Accumulation in Plants. *Environ Sci Pollut Res* **2021**, *28*, 64967–64986, doi:10.1007/s11356-021-16805-y.
50. Garcia Teijeiro, R.; Belimov, A.A.; Dodd, I.C. Microbial Inoculum Development for Ameliorating Crop Drought Stress: A Case Study of Variovorax Paradoxus 5C-2. *New Biotechnology* **2020**, *56*, 103–113, doi:10.1016/j.nbt.2019.12.006.
51. Glick, B.R. *Beneficial Plant-Bacterial Interactions*; Springer International Publishing: Cham, 2020; ISBN 978-3-030-44367-2.
52. Goel, R., Soni, R., Suyal, D.C. *Microbiological Advancements for Higher Altitude Agro-Ecosystems & Sustainability*; Eds.; Rhizosphere Biology; Springer Singapore: Singapore, 2020; ISBN 9789811519017.
53. Gómez-Lama Cabanás, C.; Schilirò, E.; Valverde-Corredor, A.; Mercado-Blanco, J. The Biocontrol Endophytic Bacterium Pseudomonas Fluorescens PICF7 Induces Systemic Defense Responses in Aerial Tissues upon Colonization of Olive Roots. *Front. Microbiol.* **2014**, *5*, doi:10.3389/fmicb.2014.00427.
54. González-Morales, S.; Solís-Gaona, S.; Valdés-Caballero, M.V.; Juárez-Maldonado, A.; Loredo-Treviño, A.; Benavides-Mendoza, A. Transcriptomics of Biostimulation of Plants Under Abiotic Stress. *Front. Genet.* **2021**, *12*, 583888, doi:10.3389/fgene.2021.583888.
55. Groen, S.C.; Whiteman, N.K. The Evolution of Ethylene Signaling in Plant Chemical Ecology. *J Chem Ecol* **2014**, *40*, 700–716, doi:10.1007/s10886-014-0474-5.
56. Gulzar, A.B.M.; Mazumder, P.B. Helping Plants to Deal with Heavy Metal Stress: The Role of Nanotechnology and Plant Growth Promoting Rhizobacteria in the Process of Phytoremediation. *Environ Sci Pollut Res* **2022**, *29*, 40319–40341, doi:10.1007/s11356-022-19756-0.
57. Gupta, S.; Pandey, S. Unravelling the Biochemistry and Genetics of ACC Deaminase-An Enzyme Alleviating the Biotic and Abiotic Stress in Plants. *Plant Gene* **2019**, *18*, 100175, doi:10.1016/j.plgene.2019.100175.
58. Gupta, V.V.S.R., Sharma, A.K. *Rhizosphere Biology: Interactions Between Microbes and Plants*; Eds.; Rhizosphere Biology; Springer Singapore: Singapore, 2021; ISBN 9789811561245.
59. Hao, L.; Zhang, Z.; Hao, B.; Diao, F.; Zhang, J.; Bao, Z.; Guo, W. Arbuscular Mycorrhizal Fungi Alter Microbiome Structure of Rhizosphere Soil to Enhance Maize Tolerance to La. *Ecotoxicology and Environmental Safety* **2021**, *212*, 111996, doi:10.1016/j.ecoenv.2021.111996.
60. Hardoim, P.R.; van Overbeek, L.S.; Elsas, J.D. van Properties of Bacterial Endophytes and Their Proposed Role in Plant Growth. *Trends in Microbiology* **2008**, *16*, 463–471, doi:10.1016/j.tim.2008.07.008.
61. Heo, A.Y.; Koo, Y.M.; Choi, H.W. Biological Control Activity of Plant Growth Promoting Rhizobacteria Burkholderia Contaminans AY001 against Tomato Fusarium Wilt and Bacterial Speck Diseases. *Biology* **2022**, *11*, 619, doi:10.3390/biology11040619.
62. Hu, D.; Wei, L.; Liao, W. Brassinosteroids in Plants: Crosstalk with Small-Molecule Compounds. *Biomolecules* **2021**, *11*, 1800, doi:10.3390/biom11121800.
63. Huang, C.-N.; Lin, C.-P.; Hsieh, F.-C.; Lee, S.-K.; Cheng, K.-C.; Liu, C.-T. Characterization and Evaluation of *Bacillus amyloliquefaciens* Strain WF02 Regarding Its Biocontrol Activities and Genetic Responses against Bacterial Wilt in Two Different Resistant Tomato Cultivars. *World J Microbiol Biotechnol* **2016**, *32*, 183, doi:10.1007/s11274-016-2143-z.
64. Huang, N.; Ling, H.; Su, Y.; Liu, F.; Xu, L.; Su, W.; Wu, Q.; Guo, J.; Gao, S.; Que, Y. Transcriptional Analysis Identifies Major Pathways as Response Components to *Sporisorium scitamineum* Stress in Sugarcane. *Gene* **2018**, *678*, 207–218, doi:10.1016/j.gene.2018.08.043.
65. Hussain, S.; Zhu, C.; Huang, J.; Huang, J.; Zhu, L.; Cao, X.; Nanda, S.; Khaskheli, M.A.; Liang, Q.; Kong, Y.; et al. Ethylene Response of Salt Stressed Rice Seedlings Following Ethephon and 1-Methylcyclopropene Seed Priming. *Plant Growth Regul* **2020**, *92*, 219–231, doi:10.1007/s10725-020-00632-1.
66. Ilangumaran, G.; Subramanian, S.; Smith, D.L. Soybean Leaf Proteomic Profile Influenced by Rhizobacteria Under Optimal and Salt Stress Conditions. *Front. Plant Sci.* **2022**, *13*, 809906, doi:10.3389/fpls.2022.809906.
67. Ilyas, M.; Nisar, M.; Khan, N.; Hazrat, A.; Khan, A.H.; Hayat, K.; Fahad, S.; Khan, A.; Ullah, A. Drought Tolerance Strategies in Plants: A Mechanistic Approach. *J Plant Growth Regul* **2021**, *40*, 926–944, doi:10.1007/s00344-020-10174-5.
68. Islam, M.T., Rahman, M.M., Pandey, P., Boehme, M.H., Haesaert, G. *Bacilli and Agrobiotechnology: Phytostimulation and Biocontrol: Volume 2*; Eds.; Bacilli in Climate Resilient Agriculture and Bioprospecting; Springer International Publishing: Cham, 2019; ISBN 978-3-030-15174-4.
69. Islam, W.; Noman, A.; Naveed, H.; Huang, Z.; Chen, H.Y.H. Role of Environmental Factors in Shaping the Soil Microbiome. *Environ Sci Pollut Res* **2020**, *27*, 41225–41247, doi:10.1007/s11356-020-10471-2.
70. Izaguirre-Mayoral, M.L.; Lazarovits, G.; Baral, B. Ureide Metabolism in Plant-Associated Bacteria: Purine Plant-Bacteria Interactive Scenarios under Nitrogen Deficiency. *Plant Soil* **2018**, *428*, 1–34, doi:10.1007/s11104-018-3674-x.
71. Jaiswal, A.K.; Alkan, N.; Elad, Y.; Sela, N.; Philosoph, A.M.; Graber, E.R.; Frenkel, O. Molecular Insights into Biochar-Mediated Plant Growth Promotion and Systemic Resistance in Tomato against Fusarium Crown and Root Rot Disease. *Sci Rep* **2020**, *10*, 13934, doi:10.1038/s41598-020-70882-6.
72. Jaiswal, S.K.; Mohammed, M.; Ibny, F.Y.I.; Dakora, F.D. Rhizobia as a Source of Plant Growth-Promoting Molecules: Potential Applications and Possible Operational Mechanisms. *Front. Sustain. Food Syst.* **2021**, *4*, 619676, doi:10.3389/fsufs.2020.619676.
73. Jalmi, S.K.; Sinha, A.K. Ambiguities of PGPR-Induced Plant Signaling and Stress Management. *Front. Microbiol.* **2022**, *13*, 899563, doi:10.3389/fmicb.2022.899563.
74. Jan, M.; Liu, Z.; Guo, C.; Sun, X. Molecular Regulation of Cotton Fiber Development: A Review. *IJMS* **2022**, *23*, 5004, doi:10.3390/ijms23095004.
75. Jat, S.L.; Suby, S.B.; Parihar, C.M.; Gambhir, G.; Kumar, N.; Rakshit, S. Microbiome for Sustainable Agriculture: A Review with Special Reference to the Corn Production System. *Arch Microbiol* **2021**, *203*, 2771–2793, doi:10.1007/s00203-021-02320-8.
76. Jha, C.K.; Sharma, P.; Shukla, A.; Parmar, P.; Patel, R.; Goswami, D.; Saraf, M. Microbial Enzyme, 1-Aminocyclopropane-1-Carboxylic Acid (ACC) Deaminase: An Elixir for Plant under Stress. *Physiological and Molecular Plant Pathology* **2021**, *115*, 101664, doi:10.1016/j.pmpp.2021.101664.
77. Ji, J.; Yuan, D.; Jin, C.; Wang, G.; Li, X.; Guan, C. Enhancement of Growth and Salt Tolerance of Rice Seedlings (Oryza Sativa L.) by Regulating Ethylene Production with a Novel Halotolerant PGPR Strain Glutamicibacter Sp. YD01 Containing ACC Deaminase Activity. *Acta Physiol Plant* **2020**, *42*, 42, doi:10.1007/s11738-020-3034-3.
78. Kaleem, F.; Shabir, G.; Aslam, K.; Rasul, S.; Manzoor, H.; Shah, S.M.; Khan, A.R. An Overview of the Genetics of Plant Response to Salt Stress: Present Status and the Way Forward. *Appl Biochem Biotechnol* **2018**, *186*, 306–334, doi:10.1007/s12010-018-2738-y.
79. Kang, W.; Zhu, X.; Wang, Y.; Chen, L.; Duan, Y. Transcriptomic and Metabolomic Analyses Reveal That Bacteria Promote Plant Defense during Infection of Soybean Cyst Nematode in Soybean. *BMC Plant Biol* **2018**, *18*, 86, doi:10.1186/s12870-018-1302-9.
80. Kaushal, M. Microbes in Cahoots with Plants: MIST to Hit the Jackpot of Agricultural Productivity during Drought. *IJMS* **2019**, *20*, 1769, doi:10.3390/ijms20071769.
81. Khan, M.I.R.; Ashfaque, F.; Chhillar, H.; Irfan, M.; Khan, N.A. The Intricacy of Silicon, Plant Growth Regulators and Other Signaling Molecules for Abiotic Stress Tolerance: An Entrancing Crosstalk between Stress Alleviators. *Plant Physiology and Biochemistry* **2021**, *162*, 36–47, doi:10.1016/j.plaphy.2021.02.024.
82. Khan, N.; Ali, S.; Tariq, H.; Latif, S.; Yasmin, H.; Mehmood, A.; Shahid, M.A. Water Conservation and Plant Survival Strategies of Rhizobacteria under Drought Stress. *Agronomy* **2020**, *10*, 1683, doi:10.3390/agronomy10111683.
83. King, E.; Wallner, A.; Rimbault, I.; Barrachina, C.; Klonowska, A.; Moulin, L.; Czernic, P. Monitoring of Rice Transcriptional Responses to Contrasted Colonizing Patterns of *Phytobeneficial burkholderia* s.l. Reveals a Temporal Shift in JA Systemic Response. *Front. Plant Sci.* **2019**, *10*, 1141, doi:10.3389/fpls.2019.01141.
84. Kokila, V.; Prasanna, R.; Kumar, A.; Nishanth, S.; Shukla, J.; Gulia, U.; Nain, L.; Shivay, Y.S.; Singh, A.K. Cyanobacterial Inoculation in Elevated CO_2_ Environment Stimulates Soil C Enrichment and Plant Growth of Tomato. *Environmental Technology & Innovation* **2022**, *26*, 102234, doi:10.1016/j.eti.2021.102234.
85. Kumar, S.P.J.; Chintagunta, A.D.; Reddy, Y.M.; Rajjou, L.; Garlapati, V.K.; Agarwal, D.K.; Prasad, S.R.; Simal-Gandara, J. Implications of Reactive Oxygen and Nitrogen Species in Seed Physiology for Sustainable Crop Productivity under Changing Climate Conditions. *Current Plant Biology* **2021**, *26*, 100197, doi:10.1016/j.cpb.2021.100197.
86. Kumar, V., Prasad, R., Kumar, M., Choudhary, D.K. *Microbiome in Plant Health and Disease: Challenges and Opportunities*; Eds.; Springer Singapore: Singapore, 2019; ISBN 9789811384943.
87. Kumari, S.; Amit; Jamwal, R.; Mishra, N.; Singh, D.K. Recent Developments in Environmental Mercury Bioremediation and Its Toxicity: A Review. *Environmental Nanotechnology, Monitoring & Management* **2020**, *13*, 100283, doi:10.1016/j.enmm.2020.100283.
88. Lahlali, R., Ezrari, S., Radouane, N., Kenfaoui, J., Esmaeel, Q., El Hamss, H., et al. Biological control of plant pathogens: A global perspective. *Microorganisms* **2022**, *10*, 596.
89. Lahlali, R.; Ezrari, S.; Radouane, N.; Kenfaoui, J.; Esmaeel, Q.; El Hamss, H.; Belabess, Z.; Barka, E.A. Biological Control of Plant Pathogens: A Global Perspective. *Microorganisms* **2022**, *10*, 596, doi:10.3390/microorganisms10030596.
90. Lang, H.; He, Y.; Li, F.; Ma, D.; Sun, J. Integrative Hormone and Transcriptome Analysis Underline the Role of Abscisic Acid in Seed Shattering of Weedy Rice. *Plant Growth Regul* **2021**, *94*, 261–273, doi:10.1007/s10725-021-00714-8.
91. Leal de Castilho, C.; Longoni, L.; Sampaio, J.; Lisboa, B.B.; Vargas, L.K.; Beneduzi, A. The Rhizosphere Microbiome and Growth-Promoting Rhizobacteria of the Brazilian Juçara Palm. *Rhizosphere* **2020**, *15*, 100233, doi:10.1016/j.rhisph.2020.100233.
92. Li, B.; Sang, T.; He, L.; Sun, J.; Li, J.; Guo, S. Exogenous Spermidine Inhibits Ethylene Production in Leaves of Cucumber Seedlings under NaCl Stress. *J. Amer. Soc. Hort. Sci.* **2013**, *138*, 108–113, doi:10.21273/JASHS.138.2.108.
93. Li, L.; Zeng, Y.; Cheng, X.; Shen, W. The Applications of Molecular Hydrogen in Horticulture. *Horticulturae* **2021**, *7*, 513, doi:10.3390/horticulturae7110513.
94. Li, T.; Mann, R.; Kaur, J.; Spangenberg, G.; Sawbridge, T. Transcriptome Analyses of Barley Roots Inoculated with Novel *Paenibacillus* Sp. and *Erwinia Gerundensis* Strains Reveal Beneficial Early-Stage Plant–Bacteria Interactions. *Plants* **2021**, *10*, 1802, doi:10.3390/plants10091802.
95. Li, T.; Zhang, J.; Shen, C.; Li, H.; Qiu, L. 1-Aminocyclopropane-1-Carboxylate: A Novel and Strong Chemoattractant for the Plant Beneficial Rhizobacterium *Pseudomonas putida* UW4. *MPMI* **2019**, *32*, 750–759, doi:10.1094/MPMI-11-18-0317-R.
96. Li, X.; Peng, D.; Zhang, Y.; Ju, D.; Guan, C. Klebsiella Sp. PD3, a Phenanthrene (PHE)-Degrading Strain with Plant Growth Promoting Properties Enhances the PHE Degradation and Stress Tolerance in Rice Plants. *Ecotoxicology and Environmental Safety* **2020**, *201*, 110804, doi:10.1016/j.ecoenv.2020.110804.
97. Li, Y.; Shi, H.; Zhang, H.; Chen, S. Amelioration of Drought Effects in Wheat and Cucumber by the Combined Application of Super Absorbent Polymer and Potential Biofertilizer. *PeerJ* **2019**, *7*, e6073, doi:10.7717/peerj.6073.
98. Liu, Y.; Ji, D.; Turgeon, R.; Chen, J.; Lin, T.; Huang, J.; Luo, J.; Zhu, Y.; Zhang, C.; Lv, Z. Physiological and Proteomic Responses of Mulberry Trees (Morus Alba. L.) to Combined Salt and Drought Stress. *IJMS* **2019**, *20*, 2486, doi:10.3390/ijms20102486.
99. Llanes, A., Reginato, M., Devinar, G., & Luna, V. What is known about phytohormones in halophytes? A review. *Biologia* **2018**, *73*, 727-742.
100. Loeschcke, A., Dienst, D., Wewer, V., Hage-Hülsmann, J., Dietsch, M., Kranz-Finger, S., et al. The photosynthetic bacteria Rhodobacter capsulatus and Synechocystis sp. PCC 6803 as new hosts for cyclic plant triterpene biosynthesis. *PLoS One* **2017**, *12*(12), e0189816, doi: 10.1371/journal.pone.0189816.
101. Loper, J. E., Hassan, K. A., Mavrodi, D. V., Davis, E. W., Lim, C. K., Shaffer, B. T., et al. Comparative genomics of plant-associated Pseudomonas spp.: insights into diversity and inheritance of traits involved in multitrophic interactions. *PLoS genetics* **2012**, *8*(7), e1002784, doi: 10.1371/journal.pgen.1002784.
102. Lorito, M., Woo, S. L., Harman, G. E., & Monte, E. Translational research on Trichoderma: from'omics to the field. *Annual review of phytopathology* **2010**, *48*, 395-417.
103. Maag, D., Kandula, D. R., Müller, C., Mendoza-Mendoza, A., Wratten, S. D., Stewart, A., & Rostás, M. Trichoderma atroviride LU132 promotes plant growth but not induced systemic resistance to Plutella xylostella in oilseed rape. *BioControl* **2014**, *59*, 241-252.
104. Mahdi, S.S., Singh, R. *Innovative Approaches for Sustainable Development: Theories and Practices in Agriculture*; Eds.; Springer International Publishing: Cham, 2022; ISBN 978-3-030-90548-4.
105. Maheshwari, D.K. *Bacteria in Agrobiology: Plant Probiotics*; Ed.; Springer Berlin Heidelberg: Berlin, Heidelberg, 2012; ISBN 978-3-642-27514-2.
106. Maheshwari, H.S.; Bharti, A.; Agnihotri, R.; Dukare, A.; Prabina, B.J.; Gangola, S.; Sharma, M.P. Combating the Abiotic Stress Through Phytomicrobiome Studies. In *Phytomicrobiome Interactions and Sustainable Agriculture*; Verma, A., Saini, J.K., Hesham, A.E., Singh, H.B., Eds.; Wiley, 2021; pp. 45–65 ISBN 978-1-119-64462-0.
107. Malambane, G., Nonaka, S., Shiba, H., Ezura, H., Tsujimoto, H., & Akashi, K. Comparative effects of ethylene inhibitors on Agrobacterium-mediated transformation of drought-tolerant wild watermelon. *Bioscience, Biotechnology, and Biochemistry* **2018**, *82*(3), 433-441, doi: 10.1080/09168451.2018.1431516.
108. Maquia, I. S. A., Fareleira, P., Videira e. Castro, I., Soares, R., Brito, D. R., Mbanze, A. A.,et al. The nexus between fire and soil bacterial diversity in the african miombo woodlands of niassa special reserve, mozambique. *Microorganisms* **2021**, *9*(8), 1562, doi: 10.3390/microorganisms9081562.
109. Menéndez, E., Pérez-Yépez, J., Hernández, M., Rodríguez-Pérez, A., Velázquez, E., & León-Barrios, M. Plant growth promotion abilities of phylogenetically diverse Mesorhizobium strains: effect in the root colonization and development of tomato seedlings. *Microorganisms* **2020**, *8*(3), 412.
110. Mishra, A. K., Sharma, K., & Misra, R. S.. Elicitor recognition, signal transduction and induced resistance in plants. *Journal of Plant Interactions* **2012**, *7*(2), 95-120.
111. Mishra, A., Singh, S. P., Mahfooz, S., Shukla, R., Mishra, N., Pandey, S., et al. External supplement of impulsive micromanager Trichoderma helps in combating CO 2 stress in rice grown under FACE. *Plant Molecular Biology Reporter* **2019**, *37*, 1-13.
112. Misra, S., & Chauhan, P. S. ACC deaminase-producing rhizosphere competent Bacillus spp. mitigate salt stress and promote Zea mays growth by modulating ethylene metabolism. *3 Biotech* **2020**, *10*(3), 119.
113. Mohamed, H.I., El-Beltagi, H.E.-D.S., Abd-Elsalam, K.A. *Plant Growth-Promoting Microbes for Sustainable Biotic and Abiotic Stress Management*; Eds.; Springer International Publishing: Cham, 2021; ISBN 978-3-030-66586-9.
114. Mohd Afandi, N. S., Habib, M. A. H., & Ismail, M. N. Recent insights on gene expression studies on Hevea Brasiliensis fatal leaf fall diseases. *Physiology and Molecular Biology of Plants* **2022**, *28*(2), 471-484.
115. Mokrani, S.; Nabti, E.; Cruz, C. Current Advances in Plant Growth Promoting Bacteria Alleviating Salt Stress for Sustainable Agriculture. *Applied Sciences* **2020**, *10*, 7025, doi:10.3390/app10207025.
116. Molina, L., Udaondo, Z., Montero-Curiel, M., Wittich, R. M., García-Puente, A., & Segura, A. Clover Root Exudates Favor Novosphingobium sp. HR1a Establishment in the Rhizosphere and Promote Phenanthrene Rhizoremediation. *Msphere* **2021**, *6*(4), e00412-21, doi: 10.1128/mSphere.00412-21.
117. Molina, L.; Segura, A. Biochemical and Metabolic Plant Responses toward Polycyclic Aromatic Hydrocarbons and Heavy Metals Present in Atmospheric Pollution. *Plants* **2021**, *10*, 2305, doi:10.3390/plants10112305.
118. Molinari, S., & Leonetti, P. Bio-control agents activate plant immune response and prime susceptible tomato against root-knot nematodes. *PloS one* **2019**, *14*(12), e0213230, doi: 10.1371/journal.pone.0213230.
119. Molinari, S., & Leonetti, P. Molecular signaling involved in immune system activation against root-knot nematodes by bio-control agents in tomato plants. *bioRxiv* **2019** , 556175.
120. Moreno, C. A., Castillo, F., González, A., Bernal, D., Jaimes, Y., Chaparro, M., et al. Biological and molecular characterization of the response of tomato plants treated with Trichoderma koningiopsis. *Physiological and Molecular Plant Pathology* **2009**, *74*(2), 111-120.
121. Mukherjee, P., Mitra, A., & Roy, M. Halomonas rhizobacteria of Avicennia marina of Indian Sundarbans promote rice growth under saline and heavy metal stresses through exopolysaccharide production. *Frontiers in Microbiology* **2019**, *10*, 1207, doi: 10.3389/fmicb.2019.01207.
122. Murali, M.; Gowtham, H.G.; Singh, S.B.; Shilpa, N.; Aiyaz, M.; Niranjana, S.R.; Amruthesh, K.N. Bio-Prospecting of ACC Deaminase Producing Rhizobacteria towards Sustainable Agriculture: A Special Emphasis on Abiotic Stress in Plants. *Applied Soil Ecology* **2021**, *168*, 104142, doi:10.1016/j.apsoil.2021.104142.
123. Nadarajah, K.; Abdul Rahman, N.S.N. Plant–Microbe Interaction: Aboveground to Belowground, from the Good to the Bad. *IJMS* **2021**, *22*, 10388, doi:10.3390/ijms221910388.
124. Nadeem, S.M.; Zahir, Z.A.; Naveed, M.; Ashraf, M. Microbial ACC-Deaminase: Prospects and Applications for Inducing Salt Tolerance in Plants. *Critical Reviews in Plant Sciences* **2010**, *29*, 360–393, doi:10.1080/07352689.2010.524518.
125. Nagarajan, V., Tsai, H. C., Chen, J. S., Hussain, B., Koner, S., Hseu, Z. Y., & Hsu, B. M. Comparison of bacterial communities and their functional profiling using 16S rRNA gene sequencing between the inherent serpentine-associated sites, hyper-accumulator, downgradient agricultural farmlands, and distal non-serpentine soils. *Journal of Hazardous Materials* **2022**, *431*, 128557, doi:10.1016/j.jhazmat.2022.128557.
126. Naing, A. H., Campol, J. R., Kang, H., Xu, J., Chung, M. Y., & Kim, C. K. Role of ethylene biosynthesis genes in the regulation of salt stress and drought stress tolerance in petunia. *Frontiers in plant science* **2022**, *13*, doi: 10.3389/fpls.2022.844449.
127. Naing, A.H.; Jeong, H.Y.; Jung, S.K.; Kim, C.K. Overexpression of 1-Aminocyclopropane-1-Carboxylic Acid Deaminase (AcdS) Gene in *Petunia Hybrida* Improves Tolerance to Abiotic Stresses. *Front. Plant Sci.* **2021**, *12*, 737490, doi:10.3389/fpls.2021.737490.
128. Neifar, M., Chouchane, H., Najjari, A., El Hidri, D., Mahjoubi, M., Ghedira, K., et al. Genome analysis provides insights into crude oil degradation and biosurfactant production by extremely halotolerant Halomonas desertis G11 isolated from Chott El-Djerid salt-lake in Tunisian desert. *Genomics* **2019**, *111*(6), 1802-1814, doi: 10.1016/j.ygeno.2018.12.003.
129. Niu, X., Song, L., Xiao, Y., & Ge, W. Drought-tolerant plant growth-promoting rhizobacteria associated with foxtail millet in a semi-arid agroecosystem and their potential in alleviating drought stress. *Frontiers in microbiology* **2018**, *8*, 2580, doi: 10.3389/fmicb.2017.02580.
130. Nonaka, S.; Ezura, H. Plant-*Agrobacterium* Interaction Mediated by Ethylene and Super-Agrobacterium Conferring Efficient Gene Transfer. *Front. Plant Sci.* **2014**, *5*, doi:10.3389/fpls.2014.00681.
131. Otlewska, A.; Migliore, M.; Dybka-Stępień, K.; Manfredini, A.; Struszczyk-Świta, K.; Napoli, R.; Białkowska, A.; Canfora, L.; Pinzari, F. When Salt Meddles Between Plant, Soil, and Microorganisms. *Front. Plant Sci.* **2020**, *11*, 553087, doi:10.3389/fpls.2020.553087.
132. Paliyath, G., Murr, D. P., Handa, A. K., & Lurie, S. *Postharvest biology and technology of fruits, vegetables, and flowers*;John Wiley & Sons; 2009.
133. Paniagua-López, M., Vela-Cano, M., Correa-Galeote, D., Martín-Peinado, F., Garzón, F. M., Pozo, C., et al. Soil remediation approach and bacterial community structure in a long-term contaminated soil by a mining spill (Aznalcóllar, Spain). *Science of The Total Environment* **2021**, *777*, 145128, doi:10.1016/j.scitotenv.2021.145128.
134. Polko, J.K.; Kieber, J.J. 1-Aminocyclopropane 1-Carboxylic Acid and Its Emerging Role as an Ethylene-Independent Growth Regulator. *Front. Plant Sci.* **2019**, *10*, 1602, doi:10.3389/fpls.2019.01602.
135. Poupin, M.J.; Greve, M.; Carmona, V.; Pinedo, I. A Complex Molecular Interplay of Auxin and Ethylene Signaling Pathways Is Involved in Arabidopsis Growth Promotion by Burkholderia Phytofirmans PsJN. *Front. Plant Sci.* **2016**, *7*, doi:10.3389/fpls.2016.00492.
136. Qian, L., Song, F., Xia, J., & Wang, R. A Glucuronic Acid-Producing Endophyte Pseudomonas sp. MCS15 Reduces Cadmium Uptake in Rice by Inhibition of Ethylene Biosynthesis. *Frontiers in Plant Science* **2022**, 1180, doi: 10.3389/fpls.2022.876545.
137. Rachappanavar, V.; Padiyal, A.; Sharma, J.K.; Gupta, S.K.; Negi, N. *Efficient Exploration of Silicon Derived Benefits to Combat Biotic and Abiotic Stresses in Fruit Crops*; In Review, 2021;
138. Ranjan, A., Sinha, R., Bala, M., Pareek, A., Singla-Pareek, S. L., & Singh, A. K. Silicon-mediated abiotic and biotic stress mitigation in plants: Underlying mechanisms and potential for stress resilient agriculture. *Plant Physiology and Biochemistry* **2021**, *163*, 15-25.
139. Rasheed, R.; Ashraf, M.A.; Ali, S.; Iqbal, M.; Zafar, S.; Akbar, A.; Banik, A. Role of NO in Plants. In *Nitric Oxide in Plant Biology*; Elsevier, 2022; pp. 139–168 ISBN 978-0-12-818797-5.
140. Rauf, M., Awais, M., Ud-Din, A., Ali, K., Gul, H., Rahman, M. M, et al. Molecular mechanisms of the 1-aminocyclopropane-1-carboxylic acid (ACC) deaminase producing Trichoderma asperellum MAP1 in enhancing wheat tolerance to waterlogging stress. *Frontiers in Plant Science* **2021**, *11*, 614971.
141. Ravanbakhsh, M.; Sasidharan, R.; Voesenek, L.A.C.J.; Kowalchuk, G.A.; Jousset, A. Microbial Modulation of Plant Ethylene Signaling: Ecological and Evolutionary Consequences. *Microbiome* **2018**, *6*, 52, doi:10.1186/s40168-018-0436-1.
142. Ribaudo, C. M., Riva, D. S., Gori, J. I., Zaballa, J. I., & Molina, C.. Identification of endophytic bacteria and their characterization as biocontrol agents against tomato southern blight disease. *Appli Micro Open Access* **2016**, *2*(1000123), 2.
143. Riyazuddin, R.; Verma, R.; Singh, K.; Nisha, N.; Keisham, M.; Bhati, K.K.; Kim, S.T.; Gupta, R. Ethylene: A Master Regulator of Salinity Stress Tolerance in Plants. *Biomolecules* **2020**, *10*, 959, doi:10.3390/biom10060959.
144. Rodríguez-Sifuentes, L., Marszalek, J. E., Chuck-Hernández, C., & Serna-Saldívar, S. O. Legumes protease inhibitors as biopesticides and their defense mechanisms against biotic factors. *International Journal of Molecular Sciences* **2020**, *21*(9), 3322.
145. Román-Ponce, B., Ramos-Garza, J., Arroyo-Herrera, I., Maldonado-Hernández, J., Bahena-Osorio, Y., Vásquez-Murrieta, M. S., & Wang, E. T. Mechanism of arsenic resistance in endophytic bacteria isolated from endemic plant of mine tailings and their arsenophore production. *Archives of microbiology* **2018**, *200*, 883-895, doi:10.1007/s00203-018-1495-1.
146. Romera, F.J.; García, M.J.; Lucena, C.; Martínez-Medina, A.; Aparicio, M.A.; Ramos, J.; Alcántara, E.; Angulo, M.; Pérez-Vicente, R. Induced Systemic Resistance (ISR) and Fe Deficiency Responses in Dicot Plants. *Front. Plant Sci.* **2019**, *10*, 287, doi:10.3389/fpls.2019.00287.
147. Saberi Riseh, R., Gholizadeh Vazvani, M., Ebrahimi-Zarandi, M., & Skorik, Y. A. Alginate-induced disease resistance in plants. *Polymers* **2022**, *14*, 661.
148. Saberi Riseh, R., Gholizadeh Vazvani, M., Ebrahimi-Zarandi, M., & Skorik, Y. A. Alginate-induced disease resistance in plants. *Polymers* **2022**, *14*,661.
149. Saikia, J.; Sarma, R.K.; Dhandia, R.; Yadav, A.; Bharali, R.; Gupta, V.K.; Saikia, R. Alleviation of Drought Stress in Pulse Crops with ACC Deaminase Producing Rhizobacteria Isolated from Acidic Soil of Northeast India. *Sci Rep* **2018**, *8*, 3560, doi:10.1038/s41598-018-21921-w.
150. Saleem, A. R., Brunetti, C., Khalid, A., Della Rocca, G., Raio, A., Emiliani, G., et al. Drought response of Mucuna pruriens (L.) DC. inoculated with ACC deaminase and IAA producing rhizobacteria. *PLoS One* **2018**, *13*(2), e0191218, doi: 10.1371/journal.pone.0191218.
151. Sarwat, M., Ahmad, A., Abdin, M.Z., Ibrahim, M.M.*Stress Signaling in Plants: Genomics and Proteomics Perspective, Volume 2*, Eds.; Springer International Publishing: Cham, 2017; ISBN 978-3-319-42182-7.
152. Scotti, R., D’Agostino, N., & Zaccardelli, M. Gene expression profiling of tomato roots interacting with Pseudomonas fluorescens unravels the molecular reprogramming that occurs during the early phases of colonization. *Symbiosis* **2019**, *78*(2), 177-192, doi:10.1007/s13199-019-00611-9.
153. Sharma, A. K., & Sharma, P.*Trichoderma: Host Pathogen Interactions and Applications*; Eds.; Springer Nature; 2020.
154. Shi, L., Du, N., Yuan, Y., Shu, S., Sun, J., & Guo, S. Vinegar residue compost as a growth substrate enhances cucumber resistance against the Fusarium wilt pathogen Fusarium oxysporum by regulating physiological and biochemical responses. *Environmental Science and Pollution Research* **2016**, *23*, 18277-18287, doi: 10.1007/s11356-016-6798-7.
155. Shilev, S.; Kartalska, Y.; Dimitrova, K. Bacterial Alleviation of Drought Stress in Plants: Potential Mechanisms and Challenges. In *Microbial Management of Plant Stresses*; Elsevier, 2021; pp. 55–71 ISBN 978-0-323-85193-0.
156. Shin, W.; Siddikee, Md.A.; Joe, M.M.; Benson, A.; Kim, K.; Selvakumar, G.; Kang, Y.; Jeon, S.; Samaddar, S.; Chatterjee, P.; et al. Halotolerant Plant Growth Promoting Bacteria Mediated Salinity Stress Amelioration in Plants. *Korean Journal of Soil Science and Fertilizer* **2016**, *49*, 355–367, doi:10.7745/KJSSF.2016.49.4.355.
157. Shokri-Gharelo, R., Bandehagh, A., & Hossain, M. A. Proteomic Profiling and Protein-Protein Interaction Network Reveal the Molecular Mechanisms of Susceptibility to Drought Stress in Canola (Brassica napus L.). *Phyton* **2022**, *91*(7), 1403.
158. Siddikee, M. A., Chauhan, P. S., & Sa, T. Regulation of ethylene biosynthesis under salt stress in red pepper (Capsicum annuum L.) by 1-aminocyclopropane-1-carboxylic acid (ACC) deaminase-producing halotolerant bacteria. *Journal of Plant Growth Regulation* **2012**, *31*, 265-272, doi:10.1007/s00344-011-9236-6.
159. Siddikee, M. A., Zereen, M. I., Wu, M., Zhang, W., & Dai, C. C. Phomopsis liquidambaris reduces ethylene biosynthesis in rice under salt stress via inhibiting the activity of 1-aminocyclopropane-1-carboxylate deaminase. *Archives of Microbiology* **2021**, *203*, 6215-6229.
160. Simm, S., Scharf, K. D., Jegadeesan, S., Chiusano, M. L., Firon, N., & Schleiff, E. Survey of genes involved in biosynthesis, transport, and signaling of phytohormones with focus on Solanum lycopersicum. *Bioinformatics and Biology insights* **2016**, *10*, BBI-S38425, doi: 10.4137/BBI.S38425.
161. Singh, D., Kumar, D., Satapathy, L., Pathak, J., Chandra, S., Riaz, A, et al. Insights of Lr28 mediated wheat leaf rust resistance: transcriptomic approach. *Gene* **2017**, *637*, 72-89, doi: 10.1016/j.gene.2017.09.028.
162. Singh, P., Singh, R. K., Guo, D. J., Sharma, A., Singh, R. N., Li, D. P., ... & Li, Y. R. Whole genome analysis of sugarcane root-associated endophyte Pseudomonas aeruginosa B18—a plant growth-promoting bacterium with antagonistic potential against Sporisorium scitamineum. *Frontiers in Microbiology* **2021**, *12*, 628376, doi: 10.3389/fmicb.2021.628376.
163. SkZ, A.; Vardharajula, S.; Vurukonda, S.S.K.P. Transcriptomic Profiling of Maize (*Zea Mays* L.) Seedlings in Response to Pseudomonas Putida Stain FBKV2 Inoculation under Drought Stress. *Ann Microbiol* **2018**, *68*, 331–349, doi:10.1007/s13213-018-1341-3.
164. Sofy, M.R.; Aboseidah, A.A.; Heneidak, S.A.; Ahmed, H.R. ACC Deaminase Containing Endophytic Bacteria Ameliorate Salt Stress in Pisum Sativum through Reduced Oxidative Damage and Induction of Antioxidative Defense Systems. *Environ Sci Pollut Res* **2021**, *28*, 40971–40991, doi:10.1007/s11356-021-13585-3.
165. Stearns, J.C.; Woody, O.Z.; McConkey, B.J.; Glick, B.R. Effects of Bacterial ACC Deaminase on *Brassica Napus* Gene Expression. *MPMI* **2012**, *25*, 668–676, doi:10.1094/MPMI-08-11-0213.
166. Subramanian, P.; Krishnamoorthy, R.; Chanratana, M.; Kim, K.; Sa, T. Expression of an Exogenous 1-Aminocyclopropane-1-Carboxylate Deaminase Gene in Psychrotolerant Bacteria Modulates Ethylene Metabolism and Cold Induced Genes in Tomato under Chilling Stress. *Plant Physiology and Biochemistry* **2015**, *89*, 18–23, doi:10.1016/j.plaphy.2015.02.003.
167. Surovy, M. Z., & Islam, T. Principle, diversity, mechanism, and potential of practical application of plant probiotic bacteria for the biocontrol of phytopathogens by induced systemic resistance. *Food Security and Plant Disease Management* **2021**, 75-94, doi:10.1016/B978-0-12-821843-3.00004-0.
168. Svoboda, T., Parich, A., Güldener, U., Schöfbeck, D., Twaruschek, K., Václavíková, M., et al. Biochemical characterization of the Fusarium graminearum candidate ACC-deaminases and virulence testing of knockout mutant strains. *Frontiers in Plant Science* **2019**, *10*, 1072, doi: 10.3389/fpls.2019.01072.
169. Tao, J.-J.; Chen, H.-W.; Ma, B.; Zhang, W.-K.; Chen, S.-Y.; Zhang, J.-S. The Role of Ethylene in Plants Under Salinity Stress. *Front. Plant Sci.* **2015**, *6*, doi:10.3389/fpls.2015.01059.
170. Thankappan, S.; Narayanasamy, S.; Sridharan, A.P.; Binodh, A.K.; Nirmala Kumari, A.; Parasuraman, P.; Uthandi, S. Rhizospheric Volatilome in Modulating Induced Systemic Resistance against Biotic Stress: A New Paradigm for Future Food Security. *Physiological and Molecular Plant Pathology* **2022**, *120*, 101852, doi:10.1016/j.pmpp.2022.101852.
171. Tiwari, S.; Lata, C.; Chauhan, P.S.; Nautiyal, C.S. Pseudomonas Putida Attunes Morphophysiological, Biochemical and Molecular Responses in *Cicer Arietinum* L. during Drought Stress and Recovery. *Plant Physiology and Biochemistry* **2016**, *99*, 108–117, doi:10.1016/j.plaphy.2015.11.001.
172. Toscano, S.; Ferrante, A.; Romano, D. Response of Mediterranean Ornamental Plants to Drought Stress. *Horticulturae* **2019**, *5*, 6, doi:10.3390/horticulturae5010006.
173. Tsukanova, K.A.; Сhеbоtаr, V.К.; Meyer, J.J.M.; Bibikova, T.N. Effect of Plant Growth-Promoting Rhizobacteria on Plant Hormone Homeostasis. *South African Journal of Botany* **2017**, *113*, 91–102, doi:10.1016/j.sajb.2017.07.007.
174. van der Ent, S.; Pieterse, C.M.J. Ethylene: Multi-Tasker in Plant-Attacker Interactions. In *Annual Plant Reviews Volume 44*; McManus, M.T., Ed.; Wiley-Blackwell: Oxford, UK, 2012; pp. 343–377 ISBN 978-1-118-22308-6.
175. van Loon, L. C., Geraats, B. P., & Linthorst, H. J. Ethylene as a modulator of disease resistance in plants. *Trends in plant science* **2006**, *11*, 184-191.
176. Vanderstraeten, L.; Van Der Straeten, D. Accumulation and Transport of 1-Aminocyclopropane-1-Carboxylic Acid (ACC) in Plants: Current Status, Considerations for Future Research and Agronomic Applications. *Front. Plant Sci.* **2017**, *8*, doi:10.3389/fpls.2017.00038.
177. Varma, A., Tripathi, S., & Prasad, R. *Plant biotic interactions*; Springer, 2019; doi: 10.1007/978-3-030-26657-8.
178. Vílchez, J.I.; García-Fontana, C.; Román-Naranjo, D.; González-López, J.; Manzanera, M. Plant Drought Tolerance Enhancement by Trehalose Production of Desiccation-Tolerant Microorganisms. *Front. Microbiol.* **2016**, *7*, doi:10.3389/fmicb.2016.01577.
179. Vwioko, E., Adinkwu, O., & El-Esawi, M. A. Comparative physiological, biochemical, and genetic responses to prolonged waterlogging stress in okra and maize given exogenous ethylene priming. *Frontiers in Physiology* **2017**, *8*, 632, doi: 10.3389/fphys.2017.00632.
180. Waigi, M. G., Sun, K., & Gao, Y. Sphingomonads in microbe-assisted phytoremediation: tackling soil pollution. *Trends in biotechnology* **2017**, *35*, 883-899.
181. Wang, M., Sun, Y., Gu, Z., Wang, R., Sun, G., Zhu, C., et al. Nitrate protects cucumber plants against Fusarium oxysporum by regulating citrate exudation. *Plant and Cell Physiology* **2016**, *57*(9), 2001-2012.
182. Wang, Z., Chen, Z., Kowalchuk, G. A., Xu, Z., Fu, X., & Kuramae, E. E. Succession of the resident soil microbial community in response to periodic inoculations. *Applied and Environmental Microbiology* **2021**, *87*(9), e00046-21, doi: 10.1128/AEM.00046-21.
183. Wang, Z., Li, N., Yu, Q., & Wang, H. Genome-wide characterization of salt-responsive miRNAs, circRNAs and associated ceRNA networks in tomatoes. *International Journal of Molecular Science* **2021**, *22*(22), 12238.
184. Wiggins, G.; Thomas, J.; Rahmatallah, Y.; Deen, C.; Haynes, A.; Degon, Z.; Glazko, G.; Mukherjee, A. Common Gene Expression Patterns Are Observed in Rice Roots during Associations with Plant Growth-Promoting Bacteria, Herbaspirillum Seropedicae and Azospirillum Brasilense. *Sci Rep* **2022**, *12*, 8827, doi:10.1038/s41598-022-12285-3.
185. Williams, A., Pétriacq, P., Beerling, D. J., Cotton, T. A., & Ton, J. Impacts of atmospheric CO2 and soil nutritional value on plant responses to rhizosphere colonization by soil bacteria. *Frontiers in plant science* **2018**, *9*, 1493, doi: 10.3389/fpls.2018.01493.
186. Yacoubou, A. M., Wallis, N. Z., Salami, H. A., Yaoitcha, A. S., Menkir, A., Tayo, O., & Agre, P. A. Agronomic Performance Of S1 Maize Lines Derived From A Bi-Parental Cross Under Infested And Striga Free Environments. European Scientific Journal **2021,** 17(25), 306, doi:10.19044/esj.2021.v17n25p306.
187. Yadav, A. N., Rastegari, A. A., Yadav, N., & Kour, D. *Advances in plant microbiome and sustainable agriculture: functional annotation and future challenges* (Vol. 2); Eds.; Singapore: Springer, 2020.
188. Yadav, B.; Chhaya; Dubey, R.; Gnanasekaran, P.; Narayan, O.P. OMICS Approaches towards Understanding Plant’s Responses to Counterattack Heavy Metal Stress: An Insight into Molecular Mechanisms of Plant Defense. *Plant Gene* **2021**, *28*, 100333, doi:10.1016/j.plgene.2021.100333.
189. Yadav, V. K., Raghav, M., Sharma, S. K., & Bhagat, N.. Rhizobacteriome: promising candidate for conferring drought tolerance in crops. *J. Pure Appl. Microbiol* **2020** , *14*, 73-92.
190. Yim, W. J., Kim, K. Y., Lee, Y. W., Sundaram, S. P., Lee, Y., & Sa, T. M. Real time expression of ACC oxidase and PR-protein genes mediated by Methylobacterium spp. in tomato plants challenged with Xanthomonas campestris pv. vesicatoria. *Journal of plant physiology* **2014**, *171*(12), 1064-1075, doi: 10.1016/j.jplph.2014.03.009.
191. Yim, W., Seshadri, S., Kim, K., Lee, G., & Sa, T. Ethylene emission and PR protein synthesis in ACC deaminase producing Methylobacterium spp. inoculated tomato plants (Lycopersicon esculentum Mill.) challenged with Ralstonia solanacearum under greenhouse conditions. *Plant physiology and biochemistry* **2013**, *67*, 95-104, doi: 10.1016/j.plaphy.2013.03.002.
192. Yoo, S. J., Kim, S. T., Weon, H. Y., Song, J., & Sang, M. K. Biocontrol activity of anti-salinity Bacillus mesonae H20-5 against Bacterial wilt in different tomato cultivars. *Biological Control* **2022** , *169*, 104869, doi:0.1016/j.biocontrol.2022.104869.
193. Yu, X., Shen, T., Kang, X., Cui, Y., Chen, Q., Shoaib, M., et al. Long-term phytoremediation using the symbiotic Pongamia pinnata reshaped soil micro-ecological environment. *Science of the Total Environment* **2021**, *774*, 145112, doi:10.1016/j.scitotenv.2021.145112.
194. Zaib, S.; Ahmad, I.; Shakeel, S.N. MODULATION OF BARLEY (*Hordeum Vulgare*) DEFENSE AND HORMONAL. *Pak. J. Agri. Sci.* **2021**, *57*, 1469-1481*,* doi: 10.21162/PAKJAS/20.9373 http://www.pakjas.com.pk
195. Zhang, C., Zhang, G., Wen, Y., Li, T., Gao, Y., Meng, F., et al. Pseudomonas sp. UW4 acdS gene promotes primordium initiation and fruiting body development of Agaricus bisporus. *World Journal of Microbiology and Biotechnology* **2019**, *35*, 1-10, doi: 10.1007/s11274-019-2741-7.
196. Zhang, S., Gan, Y., & Xu, B. Mechanisms of the IAA and ACC-deaminase producing strain of Trichoderma longibrachiatum T6 in enhancing wheat seedling tolerance to NaCl stress. *BMC plant biology* **2019**, *19*(1), 1-18.
197. Zhao, H., Zhong, S., Sang, L., Zhang, X., Chen, Z., Wei’s, Q. et al. PaACL silencing accelerates flower senescence and changes the proteome to maintain metabolic homeostasis in Petunia hybrida. *Journal of Experimental Botany* **2020**, *71*(16), 4858-4876, doi: 10.1093/jxb/eraa208.
198. Zhu, Y.-X.; Gong, H.-J.; Yin, J.-L. Role of Silicon in Mediating Salt Tolerance in Plants: A Review. *Plants* **2019**, *8*, 147, doi:10.3390/plants8060147.
199. Zulfiqar, F., & Hancock, J. T. Hydrogen sulfide in horticulture: Emerging roles in the era of climate change. *Plant Physiology and Biochemistry* **2020**, *155*, 667-675.

***acetyl-CoA synthetase (ACS)***

1. , S.K. Rhizosphere Signaling Nurturing Phyto-Microbiome Niche. *Trop Plant Res* **2020**, *7*, 522–528, doi:10.22271/tpr.2020.v7.i2.064.
2. 153. Groen, S.C.; Whiteman, N.K. The Evolution of Ethylene Signaling in Plant Chemical Ecology. *J Chem Ecol* **2014**, *40*, 700–716, doi:10.1007/s10886-014-0474-5.
3. Abou-Sreea, A.I.B.; Azzam, C.R.; Al-Taweel, S.K.; Abdel-Aziz, R.M.; Belal, H.E.E.; Rady, M.M.; Abdel-Kader, A.A.S.; Majrashi, A.; Khaled, K.A.M. Natural Biostimulant Attenuates Salinity Stress Effects in Chili Pepper by Remodeling Antioxidant, Ion, and Phytohormone Balances, and Augments Gene Expression. **2021**, *10*, 2316, doi: 10.3390/plants10112316.
4. Adeleke, B.S.; Babalola, O.O. Meta-Omics of Endophytic Microbes in Agricultural Biotechnology. *Biocatalysis and Agricultural Biotechnology* **2022**, *42*, 102332, doi:10.1016/j.bcab.2022.102332.
5. Adeleke, B.S.; Babalola, O.O.; Glick, B.R. Plant Growth-Promoting Root-Colonizing Bacterial Endophytes. *Rhizosphere* **2021**, *20*, 100433, doi:10.1016/j.rhisph.2021.100433.
6. Afandi, N.S.M. Recent Insights on Gene Expression Studies on Hevea Brasiliensis Fatal Leaf Fall Diseases. **2022**, *Physiol Mol Biol Plants* **2022**, *28*, 471-484, doi: 10.007/s12298-022-01145-z.
7. Afridi, M.S.; Van Hamme, J. d.; Bundschuh, J.; Sumaira; Khan, M.N.; Salam, A.; Waqar, M.; Munis, M.F.H.; Chaudhary, H.J. Biotechnological Approaches in Agriculture and Environmental Management - Bacterium Kocuria Rhizophila 14ASP as Heavy Metal and Salt- Tolerant Plant Growth- Promoting Strain. *Biologia* **2021**, *76*, 3091–3105, doi:10.1007/s11756-021-00826-6.
8. Aftab, T., Hakeem, K.R. *Plant Growth Regulators: Signalling under Stress Conditions*, Eds.; Springer: Cham, 2021; ISBN 978-3-030-61153-8.
9. Ahammed, G.J., Yu, J.-Q. *Plant Hormones under Challenging Environmental Factors*, Eds.; Springer Netherlands: Dordrecht, 2016; ISBN 978-94-017-7756-8.
10. Ahluwalia, O.; Singh, P.C.; Bhatia, R. A Review on Drought Stress in Plants: Implications, Mitigation and the Role of Plant Growth Promoting Rhizobacteria. *Resources, Environment and Sustainability* **2021**, *5*, 100032, doi:10.1016/j.resenv.2021.100032.
11. Ahmad, M.; Imtiaz, M.; Shoib Nawaz, M.; Mubeen, F.; Imran, A. What Did We Learn From Current Progress in Heat Stress Tolerance in Plants? Can Microbes Be a Solution? *Front. Plant Sci.* **2022**, *13*, 794782, doi:10.3389/fpls.2022.794782.
12. Ahmad, P., Wani, M.R. *Physiological Mechanisms and Adaptation Strategies in Plants Under Changing Environment*; Eds. Springer New York: New York, NY, 2014; ISBN 978-1-4614-8599-5.
13. Ahmed, B.; Shahid, M.; Syed, A.; Rajput, V.D.; Elgorban, A.M.; Minkina, T.; Bahkali, A.H.; Lee, J. Drought Tolerant Enterobacter Sp./Leclercia Adecarboxylata Secretes Indole-3-Acetic Acid and Other Biomolecules and Enhances the Biological Attributes of Vigna Radiata (L.) R. Wilczek in Water Deficit Conditions. *Biology* **2021**, *10*, 1149, doi:10.3390/biology10111149.
14. Ajeng, A.A.; Abdullah, R.; Ling, T.C.; Ismail, S.; Lau, B.F.; Ong, H.C.; Chew, K.W.; Show, P.L.; Chang, J.-S. Bioformulation of Biochar as a Potential Inoculant Carrier for Sustainable Agriculture. *Environmental Technology & Innovation* **2020**, *20*, 101168, doi:10.1016/j.eti.2020.101168.
15. Akhtar, N.; Ilyas, N.; Meraj, T.A.; Pour-Aboughadareh, A.; Sayyed, R.Z.; Mashwani, Z.-R.; Poczai, P. Improvement of Plant Responses by Nanobiofertilizer: A Step towards Sustainable Agriculture. **2022**, 21.
16. Alawiye, T.T.; Babalola, O.O. Metabolomics: Current Application and Prospects in Crop Production. *Biologia* **2021**, *76*, 227–239, doi:10.2478/s11756-020-00574-z.
17. Alberton, D.; Mu, M.; Valdameri, G.; Cordeiro, F.A.; Geo, M.; de Souza, E.M. Comparative Proteomics Analysis of the Rice Roots Colonized by Herbaspirillum Seropedicae Strain SmR1 Reveals Induction of the Methionine Recycling in the Plant Host. *J. Proteome Res.* **2013**, *12*, 1757-68, doi: 10.1021/pr400425f.
18. Alberton, D.; Valdameri, G.; Moure, V.R.; Monteiro, R.A.; Pedrosa, F. de O.; Müller-Santos, M.; de Souza, E.M. What Did We Learn From Plant Growth-Promoting Rhizobacteria (PGPR)-Grass Associations Studies Through Proteomic and Metabolomic Approaches? *Front. Sustain. Food Syst.* **2020**, *4*, 607343, doi:10.3389/fsufs.2020.607343.
19. Alenezi, F.N.; Slama, H.B.; Bouket, A.C.; Cherif-Silini, H.; Silini, A.; Luptakova, L.; Nowakowska, J.A.; Oszako, T.; Belbahri, L. Bacillus Velezensis: A Treasure House of Bioactive Compounds of Medicinal, Biocontrol and Environmental Importance. *Forests* **2021**, *12*, 1714, doi:10.3390/f12121714.
20. Al-Farsi, S.M.; Nawaz, A. Effects, Tolerance Mechanisms and Management of Salt Stress in Lucerne (Medicago Sativa). *Crop and Pasture Science* **2020**, *71,* 411.428, doi: 10.1071/CP20033.
21. Ali, M.A.; Naveed, M.; Mustafa, A.; Abbas, A. The Good, the Bad, and the Ugly of Rhizosphere Microbiome. In *Probiotics and Plant Health*; Kumar, V., Kumar, M., Sharma, S., Prasad, R., Eds.; Springer Singapore: Singapore, 2017; pp. 253–290 ISBN 978-981-10-3472-5.
22. Ali, S.; Khan, N. Delineation of Mechanistic Approaches Employed by Plant Growth Promoting Microorganisms for Improving Drought Stress Tolerance in Plants. *Microbiological Research* **2021**, *249*, 126771, doi:10.1016/j.micres.2021.126771.
23. Ali, S.; Kim, W.-C. Plant Growth Promotion Under Water: Decrease of Waterlogging-Induced ACC and Ethylene Levels by ACC Deaminase-Producing Bacteria. *Front. Microbiol.* **2018**, *9*, 1096, doi:10.3389/fmicb.2018.01096.
24. Ali, S.; Tyagi, A.; Mushtaq, M.; Al-Mahmoudi, H.; Bae, H. Harnessing Plant Microbiome for Mitigating Arsenic Toxicity in Sustainable Agriculture. *Environmental Pollution* **2022**, *300*, 118940, doi:10.1016/j.envpol.2022.118940.
25. Alkharabsheh, H.M.; Seleiman, M.F.; Hewedy, O.A.; Battaglia, M.L.; Jalal, R.S.; Alhammad, B.A.; Schillaci, C.; Ali, N.; Al-Doss, A. Field Crop Responses and Management Strategies to Mitigate Soil Salinity in Modern Agriculture: A Review. *Agronomy* **2021**, *11*, 2299, doi: 10.3390/agronomy11112299.
26. AL-Shwaiman, H.A. Beijerinckia Fluminensis BFC-33, a Novel Multi-Stress-Tolerant Soil Bacterium: Deciphering the Stress Amelioration, Phytopathogenic Inhibition and Growth Promotion in Triticum Aestivum (L.). *Chemosphere* **2022**, *295*, 133843, doi: 10.1016/j.chemosphere.2022.133843.
27. Alzubaidy, H. Induction of Salt Tolerance by Enterobacter Sp. SA187 in the Model Organism Arabidopsis Thaliana. *PloS Genet* **2018**, *14*, e1007273, doi: 10.1371/journal.pgen.1007273.
28. Ameen, F.; Alsamhary, K.; Alabdullatif, J.A.; ALNadhari, S. A Review on Metal-Based Nanoparticles and Their Toxicity to Beneficial Soil Bacteria and Fungi. *Ecotoxicology and Environmental Safety* **2021**, *213*, 112027, doi:10.1016/j.ecoenv.2021.112027.
29. Amrani, A.E.; Dumas, A.-S.; Wick, L.Y.; Yergeau, E.; Berthome, R. “Omics” Insights into PAH Degradation toward Improved Green Remediation Biotechnologies. *Environ. Sci. Technol.* **2015**, *49*, 11281-91, doi: 10.1021/acs.est5b01740.
30. Andrés-Barrao, C.; Alzubaidy, H.; Jalal, R.; Mariappan, K.G.; de Zélicourt, A.; Bokhari, A.; Artyukh, O.; Alwutayd, K.; Rawat, A.; Shekhawat, K.; et al. Coordinated Bacterial and Plant Sulfur Metabolism in *Enterobacter* Sp. SA187–Induced Plant Salt Stress Tolerance. *Proc. Natl. Acad. Sci. U.S.A.* **2021**, *118*, e2107417118, doi:10.1073/pnas.2107417118.
31. Ankati, S.; Rani, T.S.; Podile, A.R. Partner-Triggered Proteome Changes in the Cell Wall of Bacillus Sonorensis and Roots of Groundnut Benefit Each Other. *Microbiological Research* **2018**, *217*, 91–100, doi:10.1016/j.micres.2018.10.003.
32. Annapurna, K.; Kumar, A.; Kumar, L.V.; Govindasamy, V.; Bose, P.; Ramadoss, D. PGPR-Induced Systemic Resistance (ISR) in Plant Disease Management. In: Maheshwari. Eds. Bacteria in Agrobiology: Diseas Management. Springer, Berlin, Heidelberg, doi: 10.1007/978-3-642-33639-3_15.
33. Anshu, A., P. Agarwal, K. Mishra, U. Yadav, I. Verma, S. Chauhan, P. K. Srivastava, and P. C. Singh. Synergistic Action of Trichoderma Koningiopsis and T. Asperellum Mitigates Salt Stress in Paddy. *Physiology and Molecular Biology of Plants* **2022** 28 (5): 987-1004. doi:10.1007/s12298-022-01192-6.
34. *Approaches to Plant Stress and Their Management*; Gaur, R.K., Sharma, P., Eds.; Springer India: New Delhi, 2014; ISBN 978-81-322-1619-3.
35. Argueso, C.T.; Hansen, M.; Kieber, J.J. Regulation of Ethylene Biosynthesis. *Journal of Plant Growth Regulation* **2007**, *26*, 92-105.
36. Arora, N.K.; Fatima, T.; Mishra, I.; Verma, M.; Mishra, J.; Mishra, V. Environmental Sustainability: Challenges and Viable Solutions. *Environmental Sustainability* **2018**, *1*, 309–340, doi:10.1007/s42398-018-00038-w.
37. Assaf, M. Effect of Plant Growth Regulators and Salt Stress on Secondary Metabolite Composition in Lamiaceae Species. *South African Journal of Botany* **2022**, *144*, 480-493, doi: 10.1016/j.sajb.2021.10.030.
38. Azizoglu, U. Bacillus Thuringiensis as a Biofertilizer and Biostimulator: A Mini-Review of the Little-Known Plant Growth-Promoting Properties of Bt. *Curr Microbiol* **2019**, *76*, 1379–1385, doi:10.1007/s00284-019-01705-9.
39. Azmat, A.; Tanveer, Y.; Yasmin, H.; Hassan, M.N.; Shahzad, A.; Reddy, M.; Ahmad, A. Coactive Role of Zinc Oxide Nanoparticles and Plant Growth Promoting Rhizobacteria for Mitigation of Synchronized Effects of Heat and Drought Stress in Wheat Plants. *Chemosphere* **2022**, *297*, 133982, doi:10.1016/j.chemosphere.2022.133982.
40. Babu, S., R. Singh, D. Yadav, S. S. Rathore, R. Raj, R. Avasthe, S. K. Yadav, et al. Nanofertilizers for Agricultural and Environmental Sustainability. *Chemosphere* **2022**, 292. doi:10.1016/j.chemosphere.2021.133451.
41. Backer, R.; Rokem, J.S.; Ilangumaran, G.; Lamont, J.; Praslickova, D.; Ricci, E.; Subramanian, S.; Smith, D.L. Plant Growth-Promoting Rhizobacteria: Context, Mechanisms of Action, and Roadmap to Commercialization of Biostimulants for Sustainable Agriculture. *Front. Plant Sci.* **2018**, *9*, 1473, doi:10.3389/fpls.2018.01473.
42. Backes, A.; Charton, S.; Planchon, S.; Esmaeel, Q.; Sergeant, K.; Hausman, J.-F.; Renaut, J.; Barka, E.A.; Jacquard, C.; Guerriero, G. Gene Expression and Metabolite Analysis in Barley Inoculated with Net Blotch Fungus and Plant Growth-Promoting Rhizobacteria. *Plant Physiology and Biochemistry* **2021**, *168*, 488–500, doi:10.1016/j.plaphy.2021.10.027.
43. Bali, A.S.; Sidhu, G.P.S. Arsenic Acquisition, Toxicity and Tolerance in Plants - From Physiology to Remediation: A Review. *Chemosphere* **2021**, *283*, 131050, doi:10.1016/j.chemosphere.2021.131050.
44. Balthazar, C.; Joly, D.L.; Filion, M. Exploiting Beneficial Pseudomonas Spp. for Cannabis Production. *Front. Microbiol.* **2022**, *12*, 833172, doi:10.3389/fmicb.2021.833172.
45. Barnawal, D.; Bharti, N.; Maji, D.; Chanotiya, C. S.; Kalra, A. ACC Deaminase-Containing Arthrobacter Protophormiae Induces NaCl Stress Tolerance through Reduced ACC Oxidase Activity and Ethylene Production Resulting in Improved Nodulation and Mycorrhization in Pisum Sativum. *Journal of Plant Physiology* **2014**, *171*, 884-94, doi: 10.1016/j.jplph.2014.03.007.
46. Barnawal, D.; Bharti, N.; Tripathi, A. et al. ACC-Deaminase-Producing Endophyte Brachybacterium Paraconglomeratum Strain SMR20 Ameliorates Chlorophytum Salinity Stress via Altering Phytohormone Generation. *J Plant Growth Regul* **2016**, *35*, 553-564, doi:10.007/s00344-015-9560-3.
47. Bashandy, S.R.; Abd-Alla, M.H.; Dawood, M.F.A. Alleviation of the Toxicity of Oily Wastewater to Canola Plants by the N2-Fixing, Aromatic Hydrocarbon Biodegrading Bacterium Stenotrophomonas Maltophilia-SR1. *Applied Soil Ecology* **2020**, *154*, 103654, doi:10.1016/j.apsoil.2020.103654.
48. Bechtaoui, N.; Rabiu, M.K.; Raklami, A.; Oufdou, K.; Hafidi, M.; Jemo, M. Phosphate-Dependent Regulation of Growth and Stresses Management in Plants. *Front. Plant Sci.* **2021**, *12*, 679916, doi:10.3389/fpls.2021.679916.
49. Bellabarba, A.; Fagorzi, C.; diCenzo, G.C.; Pini, F.; Viti, C.; Checcucci, A. Deciphering the Symbiotic Plant Microbiome: Translating the Most Recent Discoveries on Rhizobia for the Improvement of Agricultural Practices in Metal-Contaminated and High Saline Lands. *Agronomy* **2019**, *9*, 529, doi:10.3390/agronomy9090529.
50. Berni, R.; Hausman, J.-F.; Villas-Boas, S.; Guerriero, G. Impact of Pseudomonas Sp. SVB-B33 on Stress- and Cell Wall-Related Genes in Roots and Leaves of Hemp under Salinity. *Horticulturae* **2022**, *8*, 336, doi: 10.3390/horticulturae8040336.
51. Bhanse, P.; Kumar, M.; Singh, L.; Awasthi, M. K.; Qureshi, A. Role of Plant Growth-Promoting Rhizobacteria in Boosting the Phytoremediation of Stressed Soils: Opportunities, Challenges, and Prospects. *Chemosphere* **2022**, *303*, 13494, doi: 10.1016/j.chemosphere.2022.134954.
52. Bhattacharyya, P.N.; Jha, D.K. Plant Growth-Promoting Rhizobacteria (PGPR): Emergence in Agriculture. *World J Microbiol Biotechnol* **2012**, *28*, 1327-50, doi: 10.007/s11274-011-0979-9.
53. Bist, V.; Anand, V.; Srivastava, S.; Kaur, J.; Naseem, M.; Mishra, S.; Srivastava, P.K.; Tripathi, R.D.; Srivastava, S. Alleviative Mechanisms of Silicon Solubilizing Bacillus Amyloliquefaciens Mediated Diminution of Arsenic Toxicity in Rice. *Journal of Hazardous Materials* **2022**, *428*, 128170, doi:10.1016/j.jhazmat.2021.128170.
54. Bomle, D.V.; Kiran, A.; Kumar, J.K.; Nagaraj, L.S.; Pradeep, C.K.; Ansari, M.A.; Alghamdi, S.; Kabrah, A.; Assaggaf, H.; Dablool, A.S.; et al. Plants Saline Environment in Perception with Rhizosphere Bacteria Containing 1-Aminocyclopropane-1-Carboxylate Deaminase. *IJMS* **2021**, *22*, 11461, doi:10.3390/ijms222111461.
55. Bonini, P.; Rouphael, Y.; Miras-Moreno, B.; Lee, B.; Cardarelli, M.; Erice, G.; Cirino, V.; Lucini, L.; Colla, G. A Microbial-Based Biostimulant Enhances Sweet Pepper Performance by Metabolic Reprogramming of Phytohormone Profile and Secondary Metabolism. *Front. Plant Sci.* **2020**, *11*, 567388, doi:10.3389/fpls.2020.567388.
56. Boorboori, M.R.; Zhang, H.-Y. Arbuscular Mycorrhizal Fungi Are an Influential Factor in Improving the Phytoremediation of Arsenic, Cadmium, Lead, and Chromium. *JoF* **2022**, *8*, 176, doi:10.3390/jof8020176.
57. Boto, A.; Jiménez-Arias, D. Pure Organic Active Compounds Against Abiotic Stress: A Biostimulant Overview. *Frontiers in Plant Science* **2020**, *11*, 575829. Doi: 10.3389/fpls.2020.575829.
58. Brandt, S. Spectrometric and In-Silico Assessment of the Adenylyl Cyclase Activity of a Recombinant Clathrin Assembly Protein from Arabidopsis Thaliana. Dissertation 2020.
59. Brooks, S.M.; Alper, H.S. Applications, Challenges, and Needs for Employing Synthetic Biology beyond the Lab. *Nat Commun* **2021**, *12*, 1390, doi:10.1038/s41467-021-21740-0.
60. Brunetti, C., Saleem A. R.; Della Rocca, G.; Emiliani, G. et al. Effects of Plant Growth-Promoting Rhizobacteria Strains Producing ACC Deaminase on Photosynthesis, Isoprene Emission, Ethylene Formation and Growth of Mucuna Pruriens (L.) DC. in Response to Water Deficit. *Journal of Biotechnology* **2021**, *331*, 53-62, doi: 10.1016/j.jbotec.2021.03.008.
61. Buffagni, V.; Vurro, F.; Janni, M.; Gullì, M.; Keller, A.A.; Marmiroli, N. Shaping Durum Wheat for the Future: Gene Expression Analyses and Metabolites Profiling Support the Contribution of BCAT Genes to Drought Stress Response. *Front. Plant Sci.* **2020**, *11*, 891, doi:10.3389/fpls.2020.00891.
62. Caradonia, F.; Buti, M.; Flore, A.; Morcia, C.; Ronga, D.; Gatti, R.; Moulin, L.; Terzi, V.; Francia, E.; Milc, J.A. *Characterization of Leaf Transcriptome in Grafted Tomato Seedlings Inoculated with Beneficial Rhizobacteria Azospirillum Brasiliensis and Paraburkholderia Graminis*; In Review, 2022;
63. Cardarelli, M.; Woo, S.L.; Rouphael, Y.; Colla, G. Seed Treatments with Microorganisms Can Have a Biostimulant Effect by Influencing Germination and Seedling Growth of Crops. *Plants* **2022**, *11*, 259, doi:10.3390/plants11030259.
64. Castañeda-Murillo, C.C.; Rojas-Ortiz, J.G.; Sánchez-Reinoso, A.D.; Chávez-Arias, C.C.; Restrepo-Díaz, H. Foliar Brassinosteroid Analogue (DI-31) Sprays Increase Drought Tolerance by Improving Plant Growth and Photosynthetic Efficiency in Lulo Plants. *Heliyon* **2022**, *8*, e08977, doi:10.1016/j.heliyon.2022.e08977.
65. Cataldo, E.; Fucile, M.; Mattii, G.B. Biostimulants in Viticulture: A Sustainable Approach against Biotic and Abiotic Stresses. *Plants* **2022**, *11*, 162, doi:10.3390/plants11020162.
66. Cellini, A.; Spinelli, F.; Donati, I.; Ryu, C.-M.; Kloepper, J.W. Bacterial Volatile Compound-Based Tools for Crop Management and Quality. *Trends in Plant Science* **2021**, *26*, 968–983, doi:10.1016/j.tplants.2021.05.006.
67. Chamkhi, I.; Benali, T.; Aanniz, T.; El Menyiy, N.; Guaouguaou, F.-E.; El Omari, N.; El-Shazly, M.; Zengin, G.; Bouyahya, A. Plant-Microbial Interaction: The Mechanism and the Application of Microbial Elicitor Induced Secondary Metabolites Biosynthesis in Medicinal Plants. *Plant Physiology and Biochemistry* **2021**, *167*, 269–295, doi:10.1016/j.plaphy.2021.08.001.
68. Chamkhi, I.; Cheto, S.; Geistlinger, J.; Zeroual, Y.; Kouisni, L.; Bargaz, A.; Ghoulam, C. Legume-Based Intercropping Systems Promote Beneficial Rhizobacterial Community and Crop Yield under Stressing Conditions. *Industrial Crops and Products* **2022**, *183*, 114958, doi:10.1016/j.indcrop.2022.114958.
69. Chandran, V.; Shaji, H.; Mathew, L. Endophytic Microbial Influence on Plant Stress Responses. In *Microbial Endophytes*; Elsevier, 2020; pp. 161–193 ISBN 978-0-12-819654-0.
70. Chaudhary, T.; Dixit, M.; Gera, R.; Shukla, A.K.; Prakash, A.; Gupta, G.; Shukla, P. Techniques for Improving Formulations of Bioinoculants. *3 Biotech* **2020**, *10*, 199, doi:10.1007/s13205-020-02182-9.
71. Chaudhary, T.; Gera, R.; Shukla1, P. Emerging Molecular Tools for Engineering Phytomicrobiome. *Indian J Microbiol* **2021**, *61*, 116–124, doi:10.1007/s12088-020-00915-1.
72. Chaudhry, S. Climate Change Regulated Abiotic Stress Mechanisms in Plants: A Comprehensive Review. *Plant Cell Reports* **2022**, *41* ,1-31, doi: 10.007/s00299-021-02759-5.
73. Chen, C.; Unrine, J.M.; Judy, J.D.; Lewis, R.W.; Guo, J.; McNear, D.H.; Tsyusko, O.V. Toxicogenomic Responses of the Model Legume Medicago Truncatula to Aged Biosolids Containing a Mixture of Nanomaterials (TiO2, Ag, and ZnO) from a Pilot Wastewater Treatment Plant. *Environ. Sci. Technol.* **2015**, *49*, 8759-68, doi: 10.1021/acs.est.5b01211.
74. Chen, W.; Tang, L.; Wang, J.; Zhu, H.; Jin, J.; Yang, J.; Fan, W. Research Advances in the Mutual Mechanisms Regulating Response of Plant Roots to Phosphate Deficiency and Aluminum Toxicity. *IJMS* **2022**, *23*, 1137, doi:10.3390/ijms23031137.
75. Cheng, C.; Wang, J.; Hou, W.; Malik, K.; Zhao, C.; Niu, X.; Liu, Y.; Huang, R.; Li, C.; Nan, Z. Elucidating the Molecular Mechanisms by Which Seed-Borne Endophytic Fungi, Epichloë Gansuensis, Increases the Tolerance of Achnatherum Inebrians to NaCl Stress. *IJMS* **2021**, *22*, 13191, doi:10.3390/ijms222413191.
76. Cheng, F.; Cheng, Z. Research Progress on the Use of Plant Allelopathy in Agriculture and the Physiological and Ecological Mechanisms of Allelopathy. *Front. Plant Sci.* **2015**, *6*, 1020, doi:10.3389/fpls.2015.01020.
77. Cheng, X., L. Sheng, S. Peng, E. Thorley, H. Cao, and K. Li. Integrated Mechanism of Heavy Metal Bioremediation from Soil to Rice (Oryza Sativa L.) Mediated by Enterococcus Faecium. *Plant Growth Regulation* **2022** 97 (3): 523-535. doi:10.1007/s10725-022-00811-2.
78. Choudhary, D.K.; Kasotia, A.; Jain, S.; Vaishnav, A.; Kumari, S.; Sharma, K.P.; Varma, A. Bacterial-Mediated Tolerance and Resistance to Plants Under Abiotic and Biotic Stresses. *J Plant Growth Regul* **2016**, *35*, 276–300, doi:10.1007/s00344-015-9521-x.
79. *Climate Change and the Microbiome: Sustenance of the Ecosphere*; Choudhary, D.K., Mishra, A., Varma, A., Eds.; Soil Biology; Springer International Publishing: Cham, 2021; Vol. 63; ISBN 978-3-030-76862-1.
80. Contesto, C.; Desbrosses, G.; Lefoulon, C.; Béna, G.; Borel, F.; Galland, M.; Gamet, L.; Varoquaux, F.; Touraine, B. Effects of Rhizobacterial ACC Deaminase Activity on Arabidopsis Indicate That Ethylene Mediates Local Root Responses to Plant Growth-Promoting Rhizobacteria. *Plant Science* **2008**, *175*, 178–189, doi:10.1016/j.plantsci.2008.01.020.
81. Cota-Ruiz, K. Toxicity of Copper Hydroxide Nanoparticles, Bulk Copper Hydroxide, and Ionic Copper to Alfalfa Plants: A Spectroscopic and Gene Expression Study. *Environmental Pollution* **2018**, *243*, 703-712, doi: 10.10167/J.envpol.2018.09.028.
82. Crialesi, P.C.B.; Thuler, R.T.; Iost Filho, F.H.; Thuler, A.M.G.; Lemos, M.V.F.; De Bortoli, S.A. Plant Growth Promoting Rhizobacteria (PGPR) and Plutella Xylostella (L.) (Lepidoptera: Plutellidae) Interaction as a Resistance Inductor Factor in Brassica Oleracea Var. Capitata. *Plant Sci. Today* **2017**, *4*, 121–132, doi:10.14719/pst.2017.4.3.305.
83. da Silva, M.S.R. de A.; dos Santos, B. de M.S.; da Silva, C.S.R. de A.; da Silva, C.S.R. de A.; Antunes, L.F. de S.; dos Santos, R.M.; Santos, C.H.B.; Rigobelo, E.C. Humic Substances in Combination With Plant Growth-Promoting Bacteria as an Alternative for Sustainable Agriculture. *Front. Microbiol.* **2021**, *12*, 719653, doi:10.3389/fmicb.2021.719653.
84. Dai, Y.; Chen, F.; Yue, L.; Li, T.; Jiang, Z.; Xu, Z.; Wang, Z.; Xing, B. Uptake, Transport, and Transformation of CeO2 Nanoparticles by Strawberry and Their Impact on the Rhizosphere Bacterial Community.*ACS Sustainable Chem. Eng.*  **2020**, 8, 4792-4800, doi: 10.1021/acssuschemeng.9b07422.
85. de la Osa, C.; Rodrìguez-Carvajal, M. A.; Gandullo. J.; Aranda, C.; Megìas, M; Lòpez-Baena, F. J.; Monreal, J. A. Plant Growth-Promoting Rhizobacteria Modulate the Concentration of Bioactive Compounds in Tomato Fruits. *Sepations* **2021**, *8*, doi: 10.3390/separations8110223.
86. De La Torre-Roche, R.; Cantu, J.; Tamez, C.; Zuverza-Mena, N.; Hamdi, H.; Adisa, I.O.; Elmer, W.; Gardea-Torresdey, J.; White, J.C. Seed Biofortification by Engineered Nanomaterials: A Pathway To Alleviate Malnutrition? *J. Agric. Food Chem.* **2020**, *68*, 12189–12202, doi:10.1021/acs.jafc.0c04881.
87. de Zélicourt, A.; Synek, L.; Saad, M.M.; Alzubaidy, H.; Jalal, R.; Xie, Y.; Andrés-Barrao, C.; Rolli, E.; Guerard, F.; Mariappan, K.G.; et al. Ethylene Induced Plant Stress Tolerance by Enterobacter Sp. SA187 Is Mediated by 2‐keto‐4‐methylthiobutyric Acid Production. *PLoS Genet* **2018**, *14*, e1007273, doi:10.1371/journal.pgen.1007273.
88. Delgado, C.; Mora-Poblete, F.; Ahmar, S.; Chen, J.-T.; Figueroa, C.R. Jasmonates and Plant Salt Stress: Molecular Players, Physiological Effects, and Improving Tolerance by Using Genome-Associated Tools. *IJMS* **2021**, *22*, 3082, doi:10.3390/ijms22063082.
89. Delgado-González, C.R.; Madariaga-Navarrete, A.; Rodríguez-Laguna, R.; Capulín-Grande, J.; Sharma, A.; Islas-Pelcastre, M. Microorganism Rhizosphere Interactions and Their Impact on the Bioremediation of Saline Soils: A Review. *Int. J. Environ. Sci. Technol.* **2022**, doi:10.1007/s13762-022-03930-5.
90. Deng, P.; Wan, W.; Azeem, M.; Riaz, L.; Zhang, W.; Yang, Y.; Li, C.; Yuan, W. Characterization of Biochar Derived from Bamboo and Its Application to Modulate the Toxic Effects of Chromium on Wheat Plant. *Biomass Conv. Bioref.* **2022**, doi:10.1007/s13399-022-02879-2.
91. Dhawi, F. Plant Growth Promoting Rhizobacteria (PGPR) Regulated Phyto and Microbial Beneficial Protein Interactions. *Open Life Sciences* **2020**, *15*, 68–78, doi:10.1515/biol-2020-0008.
92. Dhlamini, B.; Paumo, H. K.; Kamden, B. P.; Katata-Seru, L.; Bahadur, I. Nano-Engineering Metal-Based Fertilizers Using Biopolymers: An Innovative Strategy for a More Sustainable Agriculture. *Journal of Environmental Chemical Engineering* **2022**, *10*, 107729, doi:10.1016/j.jece.2022.107729.
93. Dif, G.; Belaouni, H.A.; Yekkour, A.; Goudjal, Y.; Djemouai, N.; Peňázová, E.; Čechová, J.; Berraf-Tebbal, A.; Eichmeier, A.; Zitouni, A. Performance of Halotolerant Bacteria Associated with Sahara-Inhabiting Halophytes Atriplex Halimus L. and Lygeum Spartum L. Ameliorate Tomato Plant Growth and Tolerance to Saline Stress: From Selective Isolation to Genomic Analysis of Potential Determinants. *World J Microbiol Biotechnol* **2022**, *38*, 16, doi:10.1007/s11274-021-03203-2.
94. Dimkpa, C.O.; McLean, J.E.; Britt, D.W.; Johnson, W.P.; Arey, B.; Lea, A.S.; Anderson, A.J. Nanospecific Inhibition of Pyoverdine Siderophore Production in *Pseudomonas Chlororaphis* O6 by CuO Nanoparticles. *Chem. Res. Toxicol.* **2012**, *25*, 1066–1074, doi:10.1021/tx3000285.
95. Dimopoulou, A.; Theologidis, I.; Varympopi, A.; Papafotis, D.; Mermigka, G.; Tzima, A.; Panopoulos, N.J.; Skandalis, N. Shifting Perspectives of Translational Research in Bio-Bactericides: Reviewing the Bacillus Amyloliquefaciens Paradigm. **2021**, *10*, 1202, doi: 10.3390/biology10111202.
96. Dixit, R.; Agrawal, L.; Gupta, S.; Kumar, M.; Yadav, S.;Chauhan, P. S., Nautiyal, C. S. Southern Blight Disease of Tomato Control by 1-Aminocyclopropane-1-Carboxylate (ACC) Deaminase Produ. *Plant signaling & Behavior* **2016**, *11*, doi: 10.1080/15/15592324.2015.1113363.
97. Du, J.; Liu, B.; Zhao, T. et al. Silica Nanoparticles Protect Rice against Biotic and Abiotic Stresses. *J Nanobiotechnol* **2022**, *20*, 197, doi: 10.1186/s12951-022-01420-x.
98. Du, N.; Shi, L.; Yuan, Y.; Li, B.; Shu, S.; Sun, J.; Guo, S. Proteomic Analysis Reveals the Positive Roles of the Plant-Growth-Promoting Rhizobacterium NSY50 in the Response of Cucumber Roots to Fusarium Oxysporum f. Sp. Cucumerinum Inoculation. *Front. Plant Sci.* **2016**, *7*, doi:10.3389/fpls.2016.01859.
99. Dubey, A.; Kumar A.; Malla, M. A. Approaches for the Amelioration of Adverse Effects of Drought Stress on Crop Plants. *Front Biosci (Landmark Ed)* **2021**, *26*, 928, doi:10.52586/4998.
100. Dubey, A.; Kumar, A.; Abd_Allah, E.F.; Hashem, A.; Khan, M.L. Growing More with Less: Breeding and Developing Drought Resilient Soybean to Improve Food Security. *Ecological Indicators* **2019**, *105*, 425–437, doi:10.1016/j.ecolind.2018.03.003.
101. Dubey, S.K., Verma, S.K., *Plant, Soil and Microbes in Tropical Ecosystems*, Eds.; Rhizosphere Biology; Springer Singapore: Singapore, 2021; ISBN 9789811633638.
102. Dutta, A.; Banerjee, S.; Dinda, S.; Chowdhury, I.; Haldar, S.; Bandyopadhyay, S. A Critical Analysis on the Roles of Exopolysaccharides and ACC Deaminase in Salinity Stress Tolerance in Crop Plants. *Biocatalysis and Agricultural Biotechnology* **2022**, *42*, 102372, doi: 10.1016/j.bcab.2022.102372.
103. Dyer, S.; Weir, R.; Cox, D.; Cheseto, X.; Torto, B.; Dalzell, J.J. Ethylene Response Factor (ERF) Genes Modulate Plant Root Exudate Composition and the Attraction of Plant Parasitic Nematodes. *International Journal for Parasitology* **2019**, *49*, 999–1003, doi:10.1016/j.ijpara.2019.09.001.
104. El Moukhtari, A.; Carol, P.; Mouradi, M.; Savoure, A.; Farissi, M. Silicon Improves Physiological, Biochemical, and Morphological Adaptations of Alfalfa (Medicago Sativa L.) during Salinity Stress. *Symbiosis* **2021**, *85*, 305–324, doi:10.1007/s13199-021-00814-z.
105. Elnahal, A.S.M.; El-Saadony, M.T.; Saad, A.M.; Desoky, E.-S.M.; El-Tahan, A.M.; Rady, M.M.; AbuQamar, S.F.; El-Tarabily, K.A. The Use of Microbial Inoculants for Biological Control, Plant Growth Promotion, and Sustainable Agriculture: A Review. *Eur J Plant Pathol* **2022**, *162*, 759–792, doi:10.1007/s10658-021-02393-7.
106. El-Saadony, M.T.; ALmoshadak, A.S.; Shafi, M.E.; Albaqami, N.M.; Saad, A.M.; El-Tahan, A.M.; Desoky, E.-S.M.; Elnahal, A.S.M.; Almakas, A.; Abd El-Mageed, T.A.; et al. Vital Roles of Sustainable Nano-Fertilizers in Improving Plant Quality and Quantity-an Updated Review. *Saudi Journal of Biological Sciences* **2021**, *28*, 7349–7359, doi:10.1016/j.sjbs.2021.08.032.
107. Escobar Diaz, P.A.; dos Santos, R.M.; Baron, N.C.; Gil, O.J.A.; Rigobelo, E.C. Effect of Aspergillus and Bacillus Concentration on Cotton Growth Promotion. *Front. Microbiol.* **2021**, *12*, 737385, doi:10.3389/fmicb.2021.737385.
108. Escobar Rodríguez, C., J. Novak, F. Buchholz, P. Uetz, L. Bragagna, M. Gumze, L. Antonielli, and B. Mitter. The Bacterial Microbiome of the Tomato Fruit is Highly Dependent on the Cultivation Approach and Correlates with Flavor Chemistry." *Frontiers in Plant Science* **2021** 12. doi:10.3389/fpls.2021.775722.
109. Eun, H.-D.; Ali, S.; Jung, H.; Kim, K.; Kim, W.-C. Profiling of ACC Synthase Gene (ACS11) Expression in Arabidopsis Induced by Abiotic Stresses. *Appl Biol Chem* **2019**, *62*, 42, doi:10.1186/s13765-019-0450-4.
110. Fahad, S., Hasanuzzaman, M., Alam, M., Ullah, H., Saeed, M., Ali Khan, I., Adnan, M. *Environment, Climate, Plant and Vegetation Growth*. Eds.; Springer International Publishing: Cham, 2020; ISBN 978-3-030-49731-6.
111. Fan, B.; Borriss, R. Bacillus Velezensis FZB42 in 2018: The Gram-Positive Model Strain for Plant Growth Promotion and Biocontrol. *Frontiers in Microbiology* **2018**, *9*, 2491, doi: 10.3389/fmicb.2018.02491.
112. Fang, Z. Formation and Comparative Analysis of Full-Length Transcriptome Sequencing and Next Generation Sequencing In Medicago Sativa L. Roots Under Abiotic Stress.
113. Farahat, M.G.; Mahmoud, M.K.; Youseif, S.H.; Saleh, S.A.; Kamel, Z. Alleviation of Salinity Stress in Wheat by ACC Deaminase-Producing Bacillus Aryabhattai EWR29 with Multifarious Plant Growth-Promoting Attributes. *Plant Archieves* **2020**, *20*, 417-429.
114. Fatima, T.; Arora, N.K. Plant Growth-Promoting Rhizospheric Microbes for Remediation of Saline Soils. *Plant Growth*. In: Arora, N., Kumar, N. Eds. Phyto and Rhizo Remediation. Microorganisms for Sustainability, 9, Springer, Singapore 2019, doi: 10.1007/978-981-31-9664-0_5.
115. Feki, K.; Tounsi, S.; Mrabet, M.; Mhadhbi, H.; Brini, F. Recent Advances in Physiological and Molecular Mechanisms of Heavy Metal Accumulation in Plants. *Environ Sci Pollut Res* **2021**, *28*, 64967–64986, doi:10.1007/s11356-021-16805-y.
116. Fernandez, J.C.; Burch-Smith, T. Chloroplasts as Mediators of Plant Biotic Interactions over Short and Long Distances. *Current Opinion in Plant Biology* **2019**, *50*, 148-155, doi: 10.1016/j.pbi.2019.06.002.
117. Ferreira, M.J.; Silva, H.; Cunha, A. Siderophore-Producing Rhizobacteria as a Promising Tool for Empowering Plants to Cope with Iron Limitation in Saline Soils: A Review. *Pedosphere* **2019**, *29*, 409–420, doi:10.1016/S1002-0160(19)60810-6.
118. Fortt, J.; González, M.; Morales, P.; Araya, N.; Remonsellez, F.; Coba de la Peña, T.; Ostria-Gallardo, E.; Stoll, A. Bacterial Modulation of the Plant Ethylene Signaling Pathway Improves Tolerance to Salt Stress in Lettuce (Lactuca Sativa L.). *Front. Sustain. Food Syst.* **2022**, *6*, 768250, doi:10.3389/fsufs.2022.768250.
119. Fortt, J.; Morales P.; Araya, N.; Remonsellez, F. et al. A. Bacterial Modulation of the Plant Ethylene Signaling Pathway Improves Tolerance to Salt Stress in Lettuce (Lactuca Sativa L.). *Frontiers in Sustainable Food Systems* **2022**, *6*, 768250, doi: 10.3389/fsufs.2022.768250.
120. Fujita, K.; Inui, H. Review: Biological Functions of Major Latex-like Proteins in Plants. *Plant Science* **2021**, *306*, 110856, doi:10.1016/j.plantsci.2021.110856.
121. Gahoi, P.; Omar, R.A.; Verma, N.; Gupta, G.S. Rhizobacteria and Acylated Homoserine Lactone-Based Nanobiofertilizer to Improve Growth and Pathogen Defense in *Cicer Arietinum* and *Triticum Aestivum* Plants. *ACS Agric. Sci. Technol.* **2021**, *1*, 240–252, doi:10.1021/acsagscitech.1c00039.
122. Garcia Teijeiro, R.; Belimov, A.A.; Dodd, I.C. Microbial Inoculum Development for Ameliorating Crop Drought Stress: A Case Study of Variovorax Paradoxus 5C-2. *New Biotechnology* **2020**, *56*, 103–113, doi:10.1016/j.nbt.2019.12.006.
123. Ghorai, P.; Ghosh, D. Ameliorating the Performance of NPK Biofertilizers to Attain Sustainable Agriculture with Special Emphasis on Bioengineering. *Bioresource Technology Reports* **2022**, *19*, 101117, doi:10.1016/j.biteb.2022.101117.
124. González-Morales, S.; Solís-Gaona, S.; Valdés-Caballero, M.V.; Juárez-Maldonado, A.; Loredo-Treviño, A.; Benavides-Mendoza, A. Transcriptomics of Biostimulation of Plants Under Abiotic Stress. *Front. Genet.* **2021**, *12*, 583888, doi:10.3389/fgene.2021.583888.
125. Gopal, M.; Gupta, A. Microbiome Selection Could Spur Next-Generation Plant Breeding Strategies. *Front. Microbiol.* **2016**, *7*, 1071, doi:10.3389/fmicb.2016.01971.
126. Grover, M.; Ali, Sk.Z.; Sandhya, V.; Rasul, A.; Venkateswarlu, B. Role of Microorganisms in Adaptation of Agriculture Crops to Abiotic Stresses. *World J Microbiol Biotechnol* **2011**, *27*, 1231–1240, doi:10.1007/s11274-010-0572-7.
127. Gruet, C.; Muller, D.; Moënne-Loccoz, Y. Significance of the Diversification of Wheat Species for the Assembly and Functioning of the Root-Associated Microbiome. *Front. Microbiol.* **2022**, *12*, 782135, doi:10.3389/fmicb.2021.782135.
128. Guarino, C.; Conte, B.; Spada, V.; Arena, S.; Sciarrillo, R.; Scaloni, A. Proteomic Analysis of Eucalyptus Leaves Unveils Putative Mechanisms Involved in the Plant Response to a Real Condition of Soil Contamination by Multiple Heavy Metals in the Presence or Absence of Mycorrhizal/Rhizobacterial Additives. *Environ. Sci. Technol.* **2014**, *19*, 11487-96, doi: 10.1021/es502070m.
129. Guarino, F., A. Cicatelli, S. Castiglione, D. R. Agius, G. E. Orhun, S. Fragkostefanakis, J. Leclercq, et al. An Epigenetic Alphabet of Crop Adaptation to Climate Change. *Frontiers in Genetics* **2022**, 13. doi:10.3389/fgene.2022.818727.
130. Gulzar, A.B.M.; Mazumder, P.B. Helping Plants to Deal with Heavy Metal Stress: The Role of Nanotechnology and Plant Growth Promoting Rhizobacteria in the Process of Phytoremediation. *Environ Sci Pollut Res* **2022**, *29*, 40319–40341, doi:10.1007/s11356-022-19756-0.
131. Gundaraniya, S.A.; Ambalam, P.S.; Tomar, R.S. Metabolomic Profiling of Drought-Tolerant and Susceptible Peanut ( *Arachis Hypogaea* L.) Genotypes in Response to Drought Stress. *ACS Omega* **2020**, *5*, 31209–31219, doi:10.1021/acsomega.0c04601.
132. Guo, A.; Pan, C.; Su, X.; Zhou, X.; Bao, Y. Combined Effects of Oxytetracycline and Microplastic on Wheat Seedling Growth and Associated Rhizosphere Bacterial Communities and Soil Metabolite Profiles. *Environmental Pollution* **2022**, *302*, 119046, doi:10.1016/j.envpol.2022.119046.
133. Guo, D.-J.; Li, D.-P.; Singh, R.K.; Singh, P.; Sharma, A.; Verma, K.K.; Qin, Y.; Khan, Q.; Lu, Z.; Malviya, M.K.; et al. Differential Protein Expression Analysis of Two Sugarcane Varieties in Response to Diazotrophic Plant Growth-Promoting Endophyte Enterobacter Roggenkampii ED5. *Front. Plant Sci.* **2021**, *12*, 727741, doi:10.3389/fpls.2021.727741.
134. Gupta, A.; Rai, S.; Bano, A.; Khanam, A.; Sharma, S.; Pathak, N. Comparative Evaluation of Different Salt-Tolerant Plant Growth-Promoting Bacterial Isolates in Mitigating the Induced Adverse Effect of Salinity in Pisum Sativum. *Biointerface Res Appl Chem* **2021**, *11*, 13141–13154, doi:10.33263/BRIAC115.1314113154.
135. Gupta, S.; Pandey, S. Unravelling the Biochemistry and Genetics of ACC Deaminase-An Enzyme Alleviating the Biotic and Abiotic Stress in Plants. *Plant Gene* **2019**, *18*, 100175, doi: 10.1026/j.plgene.2019.100175.
136. Gupta, S.; Schillaci, M.; Roessner, U. Metabolomics as an Emerging Tool to Study Plant–Microbe Interactions. *Emerging Topics in Life Sciences* **2022**, *6*, 175–183, doi:10.1042/ETLS20210262.
137. Hafez, Y.M.; Attia, K.A.; Kamel, S.; Alamery, S.F.; El-Gendy, S.; Al-Doss, A.A.; Mehiar, F.; Ghazy, A.I.; Ibrahim, E.I.; Abdelaal, K.A.A. Bacillus Subtilis as a Bio-Agent Combined with Nano Molecules Can Control Powdery Mildew Disease through Histochemical and Physiobiochemical Changes in Cucumber Plants. *Physiological and Molecular Plant Pathology* **2020**, *111*, 101489, doi:10.1016/j.pmpp.2020.101489.
138. Hakeem, K.R., Dar, G.H., Mehmood, M.A., Bhat, R.A. *Microbiota and Biofertilizers: A Sustainable Continuum for Plant and Soil Health*; Eds.; Springer International Publishing: Cham, 2021; ISBN 978-3-030-48770-6.
139. Halim, M. A., M. M. Rahman, D. Mondal, M. Megharaj, and R. Naidu. Bioaccumulation and Tolerance Indices of Cadmium in Wheat Plants Grown in Cadmium-Spiked Soil: Health Risk Assessment.*Frontiers in Environmental Science* **2021** 9. doi:10.3389/fenvs.2021.779588.
140. Halim, M.A.; Rahman, M.M.; Megharaj, M.; Naidu, R. Cadmium Immobilization in the Rhizosphere and Plant Cellular Detoxification: Role of Plant-Growth-Promoting Rhizobacteria as a Sustainable Solution. *J. Agric. Food Chem.* **2020**, *68*, 13497–13529, doi:10.1021/acs.jafc.0c04579.
141. Han, M.; Zhang, C.; Suglo, P.; Sun, S.; Wang, M.; Su, T. L-Aspartate: An Essential Metabolite for Plant Growth and Stress Acclimation. *Molecules* **2021**, *26*, 1887, doi:10.3390/molecules26071887.
142. Hanaka, A.; Ozimek, E.; Reszczyńska, E.; Jaroszuk-Ściseł, J.; Stolarz, M. Plant Tolerance to Drought Stress in the Presence of Supporting Bacteria and Fungi: An Efficient Strategy in Horticulture. *Horticulturae* **2021**, *7*, 390, doi:10.3390/horticulturae7100390.
143. *Handbook for Azospirillum: Technical Issues and Protocols*; Cassán, F.D., Okon, Y., Creus, C.M., Eds.; 1st ed. 2015.; Springer International Publishing : Imprint: Springer: Cham, 2015; ISBN 978-3-319-06542-7.
144. *Handbook for Azospirillum*; Cassán, F.D., Okon, Y., Creus, C.M., Eds.; Springer International Publishing: Cham, 2015; ISBN 978-3-319-06541-0.
145. Hao, B.; Zhang, Z.; Bao, Z.; Hao, L.; Diao, F.; Li, F. Y.; Guo, W. Claroideoglomus Etunicatum Affects the Structural and Functional Genes of the Rhizosphere Microbial Community to Help Maize Resist Cd and La Stresses. *Environmental Pollution* **2022**, *15*, 119559, doi: 10.1016/j.envpol.2022. 119559
146. Hao, H.-T.; Zhao, X.; Shang, Q.-H.; Wang, Y.; Guo, Z.-H.; Zhang, Y.-B.; Xie, Z.-K.; Wang, R.-Y. Comparative Digital Gene Expression Analysis of the Arabidopsis Response to Volatiles Emitted by Bacillus Amyloliquefaciens. *PLOS ONE* **2016**, *11*, e0158621, doi: 10.137/journal.pone.0158621.
147. Hardoim, P.R.; van Overbeek, L.S.; Elsas, J.D. van Properties of Bacterial Endophytes and Their Proposed Role in Plant Growth. *Trends in Microbiology* **2008**, *16*, 463–471, doi:10.1016/j.tim.2008.07.008.
148. Haskett, T.L; Tkacz, A.; Poole, P. S.. Engineering Rhizobacteria for Sustainable Agriculture. *ISME j*  **2021**, *15*, 949-964, doi: 10.1038/s41396-020-00835-4.
149. Heydarian, Z.; Yu, M.; Gruber, M.; Glick, B.R.; Zhou, R.; Hegedus, D.D. Inoculation of Soil with Plant Growth Promoting Bacteria Producing 1-Aminocyclopropane-1-Carboxylate Deaminase or Expression of the Corresponding AcdS Gene in Transgenic Plants Increases Salinity Tolerance in Camelina Sativa. *Front. Microbiol.* **2016**, *7*, doi:10.3389/fmicb.2016.01966.
150. Hidangmayum, A.; Dwivedi, P. Chitosan Based Nanoformulation for Sustainable Agriculture with Special Reference to Abiotic Stress: A Review. *J Polym Environ* **2022**, *30*, 1264–1283, doi:10.1007/s10924-021-02296-y.
151. Hirayama, T. Plant Hormonomics: A Key Tool for Deep Physiological Phenotyping to Improve Crop Productivity. *Plant Cell Physiol.* **2022**, *18*, pcac067, doi: 10.1093/pcp/pcac067.
152. Honeker, L.K.; Hildebrand, G.A.; Fudyma, J.D.; Daber, L.E.; Hoyt, D.; Flowers, S.E.; Gil-Loaiza, J.; Kübert, A.; Bamberger, I.; Anderton, C.R.; et al. Elucidating Drought-Tolerance Mechanisms in Plant Roots through ^1^ H NMR Metabolomics in Parallel with MALDI-MS, and NanoSIMS Imaging Techniques. *Environ. Sci. Technol.* **2022**, *56*, 2021–2032, doi:10.1021/acs.est.1c06772.
153. Howell, C.R.; Hanson, L.E.; Stipanovic, R.D.; Puckhaber, L.S. Induction of Terpenoid Synthesis in Cotton Roots and Control of *Rhizoctonia Solani* by Seed Treatment with *Trichoderma Virens*. *Phytopathology®* **2000**, *90*, 248–252, doi:10.1094/PHYTO.2000.90.3.248.
154. Huang, Z.; Wang, C.; Feng, Q.; Liou, R.-M.; Lin, Y.-F.; Qiao, J.; Lu, Y.; Chang, Y. The Mechanisms of Sodium Chloride Stress Mitigation by Salt-Tolerant Plant Growth Promoting Rhizobacteria in Wheat. *Agronomy* **2022**, *12*, 543, doi:10.3390/agronomy12030543.
155. Huang, Z.; Wang, C.; Feng, Q.; Liou, R.-M.; Lin, Y.-F.; Qiao, J.; Lu, Y.; Chang, Y. The Mechanisms of Sodium Chloride Stress Mitigation by Salt-Tolerant Plant Growth Promoting Rhizobacteria in Wheat. *Agronomy* **2022**,*12*,543. https:// doi.org/10.3390/agronomy12030543
156. Husen, A. The Harsh environment and resilient plants: an overview. *Harsh Environment and Plant Resilience*; Springer International Publishing: S.l., 2021; ISBN 978-3-030-65911-0.
157. Ilyas, M.; Nisar, M.; Khan, N.; Hazrat, A.; Khan, A.H.; Hayat, K.; Fahad, S.; Khan, A.; Ullah, A. Drought Tolerance Strategies in Plants: A Mechanistic Approach. *J Plant Growth Regul* **2021**, *40*, 926–944, doi:10.1007/s00344-020-10174-5.
158. Ilyas, N., N. Akhtar, H. Yasmin, S. Sahreen, Z. Hasnain, P. Kaushik, A. Ahmad, and P. Ahmad. Efficacy of Citric Acid Chelate and Bacillus Sp. in Amelioration of Cadmium and Chromium Toxicity in Wheat. *Chemosphere* **2022** 290. doi:10.1016/j.chemosphere.2021.133342.
159. Ishtiaq, H.; Bhardwaj, S.; Ashraf, A.; Kapoor, D. VERSATILE ROLE OF AUXIN AND ITS CROSSTALK WITH OTHER PLANT HORMONES TO REGULATE PLANT GROWTH AND DEVELOPMENT. *Plant Archives* **2021**, *21*, 1621-1627.
160. Jach, M. E., E. Sajnaga, and M. Ziaja. Utilization of Legume-Nodule Bacterial Symbiosis in Phytoremediation of Heavy Metal-Contaminated Soils." *Biology* **2022**, 11 (5). doi:10.3390/biology11050676.
161. Jafari, M.; Shahsavar, A. The Effect of Foliar Application of Melatonin on Changes in Secondary Metabolite Contents in Two Citrus Species Under Drought Stress Conditions. *Front. Plant Sci.* **2021**, *12*, 692735, doi:10.3389/fpls.2021.692735.
162. Jaiswal, A.K.; Alkan, N.; Elad, Y.; Sela, N.; Philosoph, A.M.; Graber, E.R.; Frenkel, O. Molecular Insights into Biochar-Mediated Plant Growth Promotion and Systemic Resistance in Tomato against Fusarium Crown and Root Rot Disease. *Sci Rep* **2020**, *10*, 13934, doi:10.1038/s41598-020-70882-6.
163. Jaiwal, P.K., Chhillar, A.K., Chaudhary, D., Jaiwal, R. *Nutritional Quality Improvement in Plants*; Eds.; Concepts and Strategies in Plant Sciences; Springer International Publishing: Cham, 2019; ISBN 978-3-319-95353-3.
164. Jalmi, S.K.; Sinha, A.K. Ambiguities of PGPR-Induced Plant Signaling and Stress Management. *Front. Microbiol.* **2022**, *13*, 899563, doi:10.3389/fmicb.2022.899563.
165. Jan, M., Z. Liu, C. Guo, and X. Sun. Molecular Regulation of Cotton Fiber Development: A Review.*International Journal of Molecular Sciences* **2022**, 23 (9), doi:10.3390/ijms23095004.
166. Jan, S.; Singh, B.; Bhardwaj, R.; Kapoor, D.; Kour, J.; Singh, R.; Alam, P.; Noureldeen, A.; Darwish, H. Application of Melatonin and PGPR Alleviates Thiamethoxam Induced Toxicity by Regulating the TCA Cycle in Brassica Juncea L. *Saudi Journal of Biological Sciences* **2022**, *29*, 1348–1354, doi:10.1016/j.sjbs.2022.01.039.
167. Jeong, H.; Choi, S.-K.; Ryu, C.-M.; Park, S.-H. Chronicle of a Soil Bacterium: Paenibacillus Polymyxa E681 as a Tiny Guardian of Plant and Human Health. *Front. Microbiol.* **2019**, *10*, 467, doi:10.3389/fmicb.2019.00467.
168. Jha, C.K.; Sharma, P.; Shukla, A.; Parmar, P.; Patel, R.; Goswami, D.; Saraf, M. Microbial Enzyme, 1-Aminocyclopropane-1-Carboxylic Acid (ACC) Deaminase: An Elixir for Plant under Stress. *Physiological and Molecular Plant Pathology* **2021**, *115*, 101664, doi:10.1016/j.pmpp.2021.101664.
169. Jha, Y.; Dehury, B.; Kumar, S. P. J. et al. Delineation of Molecular Interactions of Plant Growth Promoting Bacteria Induced β-1,3-Glucanases and Guanosine Triphosphate Ligand for Antifungal Response in Rice: A Molecular Dynamics Approach. *Molecular Biology Reports* **2022**, *49*, 2579-2589, doi: 10.1007/s11033-021-07059-5.
170. Jha, Y.; Mohamed, H.I. Plant Secondary Metabolites as a Tool to Investigate Biotic Stress Tolerance in Plants: A Review. *Gesunde Pflanzen* **2022**, doi:10.1007/s10343-022-00669-4.
171. Ji, S.-H.; Kim, J.-S.; Lee, C.-H.; Seo, H.-S.; Chun, S.-C.; Oh, J.; Choi, E.-H.; Park, G. Enhancement of Vitality and Activity of a Plant Growth-Promoting Bacteria (PGPB) by Atmospheric Pressure Non-Thermal Plasma. *Sci Rep* **2019**, *9*, 1044, doi:10.1038/s41598-018-38026-z.
172. Jiang, M.; Song, Y.; Kanwar, M.K.; Ahammed, G.J.; Shao, S.; Zhou, J. Phytonanotechnology Applications in Modern Agriculture. *J Nanobiotechnol* **2021**, *19*, 430, doi:10.1186/s12951-021-01176-w.
173. Jo, J.; Lee, J.; Ahn, Y.; Hwang, Y.S.; Park, J.; Lee, J.; Choi, J. Metabolome and Transcriptome Analyses of Plants Grown in Naturally Attenuated Soil after Hydrogen Fluoride Exposure. *Journal of Hazardous Materials* **2022**, *437*, 129323, doi:10.1016/j.jhazmat.2022.129323.
174. Jung, H.; Ali, S.; Kim, J.Y.; Kim, W.-C. Transgenic *Arabidopsis* Expressing *AcdS* Gene of *Pseudomonas Veronii-* KJ Alleviate the Adverse Effects of Salt and Water-Logging Stress. *Plant Breed. Biotech.* **2018**, *6*, 221–232, doi:10.9787/PBB.2018.6.3.221.
175. Juurakko, C.L.; DiCenzo, G. C.; Walker.V. K. Cold Acclimation and Prospects for Cold-Resilient Crops. *Plant Stress* **2021**, *2*, doi: 10.1016/j.stress.2021.100028.
176. Kachroo, A.; Kachroo, P. Fatty Acid–Derived Signals in Plant Defense. *Annula Rev Phytopathol.* **2009**, *47*, 153-76, doi: 10.1146/annurev-phyto-080508-081820.
177. Kafle, A.; Timilsina, A.; Gautam, A.; Adhikari, K.; Bhattarai, A.; Aryal, N. Phytoremediation: Mechanisms, Plant Selection and Enhancement by Natural and Synthetic Agents. *Environmental Advances* **2022**, *8*, 100203, doi:10.1016/j.envadv.2022.100203.
178. Kaleem, F.; Shabir, G.; Aslam, K.; Rasul, S.; Manzoor, H.; Shah, S.M.; Khan, A.R. An Overview of the Genetics of Plant Response to Salt Stress: Present Status and the Way Forward. *Appl Biochem Biotechnol* **2018**, *186*, 306–334, doi:10.1007/s12010-018-2738-y.
179. Kalia, V.C.; Gong, C.; Patel, S.K.S.; Lee, J.-K. Regulation of Plant Mineral Nutrition by Signal Molecules. *Microorganisms* ***2021***, *9*, 774, doi:10.3390/microorganisms9040774.
180. Kalwani, M.; Chakdar, H.; Srivastava, A.; Pabbi, S.; Shukla, P. Effects of Nanofertilizers on Soil and Plant-Associated Microbial Communities: Emerging Trends and Perspectives. *Chemosphere* **2022**, *287*, 132107, doi:10.1016/j.chemosphere.2021.132107.
181. Kamilova, F.; Kravchenko, L.V.; Shaposhnikov, A.I.; Azarova, T.; Makarova, N.; Lugtenberg, B. Organic Acids, Sugars, and l -Tryptophane in Exudates of Vegetables Growing on Stonewool and Their Effects on Activities of Rhizosphere Bacteria. *MPMI* **2006**, *19*, 250–256, doi:10.1094/MPMI-19-0250.
182. Kang, W.; Zhu, X.; Wang, Y.; Chen, L.; Duan, Y. Transcriptomic and Metabolomic Analyses Reveal That Bacteria Promote Plant Defense during Infection of Soybean Cyst Nematode in Soybean. *BMC Plant Biol* **2018**, *18*, 86, doi:10.1186/s12870-018-1302-9.
183. Karavidas, I.; Ntatsi, G.; Vougeleka, V.; Karkanis, A.; Ntanasi, T.; Saitanis, C.; Agathokleous, E.; Ropokis, A.; Sabatino, L.; Tran, F.; et al. Agronomic Practices to Increase the Yield and Quality of Common Bean (Phaseolus Vulgaris L.): A Systematic Review. *Agronomy* **2022**, *12*, 271, doi:10.3390/agronomy12020271.
184. Karuppiah, V.; Vallikkannu, M.; Li, T.; Chen, J. Simultaneous and Sequential Based Co-Fermentations of Trichoderma Asperellum GDFS1009 and Bacillus Amyloliquefaciens 1841: A Strategy to Enhance the Gene Expression and Metabolites to Improve the Bio-Control and Plant Growth Promoting Activity. *Microb Cell Fact* **2019**, *18*, 185, doi:10.1186/s12934-019-1233-7.
185. Kaur, R.; Kaur, S.; Kaur, G. Molecular and Physiological Manipulations in Rhizospheric Bacteria. *Acta Physiol Plant* **2021**, *43*, 77, doi:10.1007/s11738-021-03251-z.
186. Kaushal, M. Microbes in Cahoots with Plants: MIST to Hit the Jackpot of Agricultural Productivity during Drought. *IJMS* **2019**, *20*, 1769, doi:10.3390/ijms20071769.
187. Kaushal, M.; Wani, S.P. Rhizobacterial-Plant Interactions: Strategies Ensuring Plant Growth Promotion under Drought and Salinity Stress. *Agriculture, Ecosystems & Environment* **2016**, *231*, 68–78, doi:10.1016/j.agee.2016.06.031.
188. Kaushik, P.; Saini, D.K. Silicon as a Vegetable Crops Modulator—A Review. *Plants* **2019**, *8*, 148, doi:10.3390/plants8060148.
189. Khan, M.A.; Kalsoom; Imran, M.; Lubna; Shaffique, S.; Kwon, E.-H.; Kang, S.-M.; Kim, S.-H.; Hamayun, M.; Lee, I.-J. Mitigation of Commercial Food Waste-Related Salinity Stress Using Halotolerant Rhizobacteria in Chinese Cabbage Plants. *Horticulturae* **2022**, *8*, 49, doi:10.3390/horticulturae8010049.
190. Khan, M.I.R. The Intricacy of Silicon, Plant Growth Regulators and Other Signaling Molecules for Abiotic Stress Tolerance: An Entrancing Crosstalk between Stress Alleviators. *Plant Physiology and Biochemistry* **2021**, *162*, 36-47, doi: 10.1016/j.plaphy.2021.02.024.
191. Khan, N.; Ali, S.; Tariq, H.; Latif, S.; Yasmin, H.; Mehmood, A.; Shahid, M.A. Water Conservation and Plant Survival Strategies of Rhizobacteria under Drought Stress. *Agronomy* **2020**, *10*, 1683, doi:10.3390/agronomy10111683.
192. Khan, N.; Bano, A.; Rahman, M.A.; Guo, J.; Kang, Z.; Babar, Md.A. Comparative Physiological and Metabolic Analysis Reveals a Complex Mechanism Involved in Drought Tolerance in Chickpea (Cicer Arietinum L.) Induced by PGPR and PGRs. *Sci Rep* **2019**, *9*, 2097, doi:10.1038/s41598-019-38702-8.
193. Khan, S.; Shahid, M.; Khan, M.S.; Syed, A.; Bahkali, A.H.; Elgorban, A.M.; Pichtel, J. Fungicide-Tolerant Plant Growth-Promoting Rhizobacteria Mitigate Physiological Disruption of White Radish Caused by Fungicides Used in the Field Cultivation. *IJERPH* **2020**, *17*, 7251, doi:10.3390/ijerph17197251.
194. Khanna, K. Unsnarling Plausible Role of Plant Growth-Promoting Rhizobacteria for Mitigating Cd-Toxicity from Plants: An Environmental Safety Aspect. *Journal of Plant Growth Regulation* **2022**, *41*, 2514-2542, doi: 10.1007/s00344-021-10445-9.
195. Khanna, K.; Jamal. V. L.; Kohli, S. K.; Gandhi, S. G.; Ohri, P. et al. Plant Growth Promoting Rhizobacteria Induced Cd Tolerance in Lycopersicon Esculentum through Altered Antioxidative Defense Expression.*Chemosphere*  **2019**, *217*, 463-474, doi: 10.1016/j.chemosphere.2018.11.005.
196. Khanna, K.; Jamwal, V.L.; Kohli, S.K.; Gandhi, S.G.; Ohri, P.; Bhardwaj, R.; Wijaya, L.; Alyemeni, M.N.; Ahmad, P. Role of Plant Growth Promoting Bacteria (PGPRs) as Biocontrol Agents of Meloidogyne Incognita through Improved Plant Defense of Lycopersicon Esculentum. *Plant Soil* **2019**, *436*, 325–345, doi:10.1007/s11104-019-03932-2.
197. Khanna, K.; Kohli, S.K.; Handa, N.; Kaur, H.; Ohri, P.; Bhardwaj, R.; Yousaf, B.; Rinklebe, J.; Ahmad, P. Enthralling the Impact of Engineered Nanoparticles on Soil Microbiome: A Concentric Approach towards Environmental Risks and Cogitation. *Ecotoxicology and Environmental Safety* **2021**, *222*, 112459, doi:10.1016/j.ecoenv.2021.112459.
198. Khare, E.; Mishra, J.; Arora, N.K. Multifaceted Interactions Between Endophytes and Plant: Developments and Prospects. *Front. Microbiol.* **2018**, *9*, 2732, doi:10.3389/fmicb.2018.02732.
199. Khare, E.; Tyagi, S.; Patil, K.S. Language of Plant-Microbe-Microbe Interactions in Rhizospheric Ecosystems. In *Molecular Aspects of Plant Beneficial Microbes in Agriculture*; Elsevier, 2020; pp. 59–76 ISBN 978-0-12-818469-1.
200. Kisvarga, S.; Farkas, D.; Boronkay, G.; Neményi, A.; Orlóci, L. Effects of Biostimulants in Horticulture, with Emphasis on Ornamental Plant Production. *Agronomy* **2022**, *12*, 1043, doi:10.3390/agronomy12051043.
201. Kokina, I.; Plaksenkova, I.; Galek, R.; Jermaļonoka, M.; Kirilova, E.; Gerbreders, V.; Krasovska, M.; Sledevskis, E. Genotoxic Evaluation of Fe3O4 Nanoparticles in Different Three Barley (Hordeum Vulgare L.) Genotypes to Explore the Stress-Resistant Molecules. *Molecules* **2021**, *26*, 6710, doi:10.3390/molecules26216710.
202. Kong, W.-L.; Wang, W.-Y.; Zuo, S.-H.; Wu, X.-Q. Genome Sequencing of Rahnella Victoriana JZ-GX1 Provides New Insights Into Molecular and Genetic Mechanisms of Plant Growth Promotion. *Front. Microbiol.* **2022**, *13*, 828990, doi:10.3389/fmicb.2022.828990.
203. Kong, W.-L.; Wang, Y.-H.; Wu, X.-Q. Enhanced Iron Uptake in Plants by Volatile Emissions of Rahnella Aquatilis JZ-GX1. *Front. Plant Sci.* **2021**, *12*, 704000, doi:10.3389/fpls.2021.704000.
204. Kong, Z.; Glick, B.R. The Role of Plant Growth-Promoting Bacteria in Metal Phytoremediation. In *Advances in Microbial Physiology*; Elsevier, 2017; Vol. 71, pp. 97–132 ISBN 978-0-12-812385-0.
205. Korres, N.E.; Loka, D. A.; Gitsopoulos, T. K. et al. Salinity Effects on Rice, Rice Weeds, and Strategies to Secure Crop Productivity and Effective Weed Control. A Review. *Agron. Sustain. Dev.* **2022**, *42*, 58, doi: 10.1007/s13593-022-00794-4.
206. Krishnamoorthy, R.; Roy Choudhury, A.; Walitang, D.I.; Anandham, R.; Senthilkumar, M.; Sa, T. Salt Stress Tolerance-Promoting Proteins and Metabolites under Plant-Bacteria-Salt Stress Tripartite Interactions. *Applied Sciences* **2022**, *12*, 3126, doi:10.3390/app12063126.
207. Krishnatreya, D.B.; Agarwala, N.; Gill, S.S.; Bandyopadhyay, T. Understanding the Role of MiRNAs for Improvement of Tea Quality and Stress Tolerance. *Journal of Biotechnology* **2021**, *328*, 34–46, doi:10.1016/j.jbiotec.2020.12.019.
208. Kumar, M., Kumar, V., Prasad, R., Sharma, S. *Probiotics and Plant Health*; Eds.; 1st ed. 2017.; Springer Singapore : Imprint: Springer: Singapore, 2017; ISBN 978-981-10-3473-2.
209. Kumar, M.; Giri, V.P.; Pandey, S.; Gupta, A.; Patel, M.K.; Bajpai, A.B.; Jenkins, S.; Siddique, K.H.M. Plant-Growth-Promoting Rhizobacteria Emerging as an Effective Bioinoculant to Improve the Growth, Production, and Stress Tolerance of Vegetable Crops. *IJMS* **2021**, *22*, 12245, doi:10.3390/ijms222212245.
210. Kumar, S.; Choudhary, A.K.; Suyal, D.C.; Makarana, G.; Goel, R. Leveraging Arsenic Resistant Plant Growth-Promoting Rhizobacteria for Arsenic Abatement in Crops. *Journal of Hazardous Materials* **2022**, *425*, 127965, doi:10.1016/j.jhazmat.2021.127965.
211. Kumar, S.; Diksha; Sindhu, S.S.; Kumar, R. Biofertilizers: An Ecofriendly Technology for Nutrient Recycling and Environmental Sustainability. *Current Research in Microbial Sciences* **2022**, *3*, 100094, doi:10.1016/j.crmicr.2021.100094.
212. Kumari, M.; Pandey, S.; Mishra, S.K.; Giri, V.P.; Agarwal, L.; Dwivedi, S.; Pandey, A.K.; Nautiyal, C.S.; Mishra, A. Omics-Based Mechanistic Insight Into the Role of Bioengineered Nanoparticles for Biotic Stress Amelioration by Modulating Plant Metabolic Pathways. *Front. Bioeng. Biotechnol.* **2020**, *8*, 242, doi:10.3389/fbioe.2020.00242.
213. Kunert, K.J.; Vorster, B.J.; Fenta, B.A.; Kibido, T.; Dionisio, G.; Foyer, C.H. Drought Stress Responses in Soybean Roots and Nodules. *Front. Plant Sci.* **2016**, *7*, 1015, doi:10.3389/fpls.2016.01015.
214. Kuźniar; Włodarczyk; Wolińska Agricultural and Other Biotechnological Applications Resulting from Trophic Plant-Endophyte Interactions. *Agronomy* **2019**, *9*, 779, doi:10.3390/agronomy9120779.
215. Lacava, P.T.; Bogas, A.C.; Cruz, F. de P.N. Plant Growth Promotion and Biocontrol by Endophytic and Rhizospheric Microorganisms From the Tropics: A Review and Perspectives. *Front. Sustain. Food Syst.* **2022**, *6*, 796113, doi:10.3389/fsufs.2022.796113.
216. Lammerts van Bueren, E.T.; Jones, S.S.; Tamm, L.; Murphy, K.M.; Myers, J.R.; Leifert, C.; Messmer, M.M. The Need to Breed Crop Varieties Suitable for Organic Farming, Using Wheat, Tomato and Broccoli as Examples: A Review. *NJAS: Wageningen Journal of Life Sciences* **2011**, *58*, 193–205, doi:10.1016/j.njas.2010.04.001.
217. Łata, B.; Łaźny, R.; Przybyłko, S.; Wrona, D. Malus Antioxidant Metabolism Following Bacterial–Fungal Inoculation in Organic Farming: From Root to Fruit. *Applied Sciences* **2021**, *11*, 9466, doi:10.3390/app11209466.
218. Lee, S.-M.; Lee, B.; Shim, C.-K.; Chang, Y.-K.; Ryu, C.-M. Plant Anti-Aging: Delayed Flower and Leaf Senescence in *Erinus Alpinus* Treated with Cell-Free *Chlorella* Cultivation Medium. *Plant Signaling & Behavior* **2020**, *15*, 1763005, doi:10.1080/15592324.2020.1763005.
219. Li, B.; Sang, T.; He, L.; Sun, J.; Li, J.; Guo, S. Exogenous Spermidine Inhibits Ethylene Production in Leaves of Cucumber Seedlings under NaCl Stress. *J. Amer. Soc. Hort. Sci.* **2013**, *138*, 108–113, doi:10.21273/JASHS.138.2.108.
220. Li, K.; Pidatala, V.R.; Shaik, R.; Datta, R.; Ramakrishna, W. Integrated Metabolomic and Proteomic Approaches Dissect the Effect of Metal-Resistant Bacteria on Maize Biomass and Copper Uptake. *Environ. Sci. Technol.* **2014**, *48*, 1184–1193, doi:10.1021/es4047395.
221. Li, L. Effect of Piriformospora Indica-Induced Systemic Resistance and Basal Immunity Against Rhizoctonia Cerealis and Fusarium Graminearum in Wheat. *Frontiers in Plant Science* **2022**, *13*, 16.
222. Li, M.; Zhang, X.; Yang, H.; Li, X.; Cui, Z. Soil Sustainable Utilization Technology: Mechanism of Flavonols in Resistance Process of Heavy Metal. *Environ Sci Pollut Res* **2018**, *25*, 26669–26681, doi:10.1007/s11356-018-2485-1.
223. Li, N.; Wang, Z.; Wang, B.; Wang, J.; Xu, R.; Yang, T.; Huang, S.; Yu, Q.; Wang, H.; Gao, J. Identification and Characterization of Long Non-Coding RNA in Tomato Roots under Salt Stress. *Bioinformatics* **2021**, *13*, 834027, doi: 10.3389/fpls.2022. 834027.
224. Li, R.; Su, X.; Zhou, R.; Zhang, Y.; Wang, T. Molecular Mechanism of Mulberry Response to Drought Stress Revealed by Complementary Transcriptomic and ITRAQ Analyses. *BMC Plant Biol* **2022**, *22*, 36, doi:10.1186/s12870-021-03410-x.
225. Li, T.; Mann, R.; Kaur, J.; Spangenberg, G.; Sawbridge, T. Transcriptome Analyses of Barley Roots Inoculated with Novel Paenibacillus Sp. and Erwinia Gerundensis Strains Reveal Beneficial Early-Stage Plant–Bacteria Interactions. *Plants* **2021**, *10*, 1802, doi:10.3390/plants10091802.
226. Li, Y.; Tian, H.; He, Q.; Geng, Z.; Yan, S.; Tuniyazi, G.; Bai, X. Investigation of the Geographical Environment Impact on the Chemical Components of *Peganum Harmala* L. through a Combined Analytical Method. *ACS Omega* **2021**, *6*, 25497–25505, doi:10.1021/acsomega.1c03420.
227. Liang, X.; Zhang, L.; Natarajan, S.K.; Becker, D.F. Proline Mechanisms of Stress Survival. *Antioxidants & Redox Signaling* **2013**, *19*, 998–1011, doi:10.1089/ars.2012.5074.
228. Liu, C.-H.; Siew, W.; Hung, Y.-T.; Jiang, Y.-T.; Huang, C.-H. 1-Aminocyclopropane-1-Carboxylate (ACC) Deaminase Gene in *Pseudomonas Azotoformans* Is Associated with the Amelioration of Salinity Stress in Tomato. *J. Agric. Food Chem.* **2021**, *69*, 913–921, doi:10.1021/acs.jafc.0c05628.
229. Liu, H.; Timko, M.P. Jasmonic Acid Signaling and Molecular Crosstalk with Other Phytohormones. *IJMS* **2021**, *22*, 2914, doi:10.3390/ijms22062914.
230. Liu, Y.; Cao, X.; Yue, L.; Wang, C.; Tao, M.; Wang, Z.; Xing, B. Foliar-Applied Cerium Oxide Nanomaterials Improve Maize Yield under Salinity Stress: Reactive Oxygen Species Homeostasis and Rhizobacteria Regulation. *Environmental Pollution* **2022**, *299*, 118900, doi:10.1016/j.envpol.2022.118900.
231. Lubna; Asaf, S.; Hamayun, M.; Khan, A.L.; Waqas, M.; Khan, M.A.; Jan, R.; Lee, I.-J.; Hussain, A. Salt Tolerance of Glycine Max .L Induced by Endophytic Fungus Aspergillus Flavus CSH1, via Regulating Its Endogenous Hormones and Antioxidative System. *Plant Physiology and Biochemistry* **2018**, *128*, 13–23, doi:10.1016/j.plaphy.2018.05.007.
232. Lucas, J.A.; García-Villaraco, A.; Ramos-Solano, B.; Akdi, K.; Gutierrez-Mañero, F.J. Lipo-Chitooligosaccharides (LCOs) as Elicitors of the Enzymatic Activities Related to ROS Scavenging to Alleviate Oxidative Stress Generated in Tomato Plants under Stress by UV-B Radiation. *Plants* **2022**, *11*, 1246, doi:10.3390/plants11091246.
233. Luo, L., C. Zhao, E. Wang, A. Raza, and C. Yin. Bacillus Amyloliquefaciens as an Excellent Agent for Biofertilizer and Biocontrol in Agriculture: An Overview for its Mechanisms. *Microbiological Research* **2022**, 259, doi:10.1016/j.micres.2022.127016.
234. Ma, C. ; Song, W.; Yang, J., Ren, C. et al. The Role and Mechanism of Commercial Macroalgae for Soil Conditioner and Nutrient Uptake Catalyzer. *Plant Growth Regulation* **2022**, *97*, 455-476, doi:10.1007/s10725-022-00819-8.
235. Ma, Y.; Jiao, J.; Fan, X.; Sun, H.; Zhang, Y.; Jiang, J.; Liu, C. Endophytic Bacterium Pseudomonas Fluorescens RG11 May Transform Tryptophan to Melatonin and Promote Endogenous Melatonin Levels in the Roots of Four Grape Cultivars. *Front. Plant Sci.* **2017**, *7*, 2068, doi:10.3389/fpls.2016.02068.
236. Ma, Y.; Oliveira, R. S.; Freitas, H.; Zhang, C. Biochemical and Molecular Mechanisms of Plant-Microbe-Metal Interactions: Relevance for Phytoremediation. *Frontiers in Plant Science* **2016**, *7*, 918, d0i: 10.3389/fpld.2016.00918.
237. Ma, Y.; Prasad, M.N.V.; Rajkumar, M.; Freitas, H. Plant Growth Promoting Rhizobacteria and Endophytes Accelerate Phytoremediation of Metalliferous Soils. *Biotechnology Advances* **2011**, *29*, 248–258, doi:10.1016/j.biotechadv.2010.12.001.
238. Mahdi Dar, Z.; Masood, A.; Hussain Mughal, A.; Asif, M.; Ahamd Malik, M. Review on Drought Tolerance in Plants Induced by Plant Growth Promoting Rhizobacteria. *Int.J.Curr.Microbiol.App.Sci* **2018**, *7*, 412–422, doi:10.20546/ijcmas.2018.705.053.
239. Maheshwari, D.K. *Bacteria in Agrobiology: Plant Nutrient Management*; Ed.; Springer Berlin Heidelberg: Berlin, Heidelberg, 2011; ISBN 978-3-642-21060-0.
240. Maheshwari, D.K., Dheeman, S. *Field Crops: Sustainable Management by PGPR*; Eds.; Sustainable Development and Biodiversity; Springer International Publishing: Cham, 2019; Vol. 23; ISBN 978-3-030-30925-1.
241. Mangal, V.; Lal, M.K.; Tiwari, R.K.; Altaf, M.A.; Sood, S.; Kumar, D.; Bharadwaj, V.; Singh, B.; Singh, R.K.; Aftab, T. Molecular Insights into the Role of Reactive Oxygen, Nitrogen and Sulphur Species in Conferring Salinity Stress Tolerance in Plants. *J Plant Growth Regul* **2022**, doi:10.1007/s00344-022-10591-8.
242. Mansour, E., H. A. M. Mahgoub, S. A. Mahgoub, E. -S E. A. El-Sobky, M. I. Abdul-Hamid, M. M. Kamara, S. F. AbuQamar, K. A. El-Tarabily, and E. -S M. Desoky. Enhancement of Drought Tolerance in Diverse Vicia Faba Cultivars by Inoculation with Plant Growth-Promoting Rhizobacteria Under Newly Reclaimed Soil Conditions. *Scientific Reports* **2021** 11 (1). doi:10.1038/s41598-021-02847-2.
243. Martin-Rivilla, H.; Garcia-Villaraco, A.; Ramos-Solano, B.; Gutierrez-Manero, F.J.; Lucas, J.A. Improving Flavonoid Metabolism in Blackberry Leaves and Plant Fitness by Using the Bioeffector *Pseudomonas Fluorescens* N 21.4 and Its Metabolic Elicitors: A Biotechnological Approach for a More Sustainable Crop. *J. Agric. Food Chem.* **2020**, *68*, 6170–6180, doi:10.1021/acs.jafc.0c01169.
244. Maswada, H.F.; Mazrou, Y.S.A.; Elzaawely, A.A.; Alam-Eldein, S.M. Nanomaterials. Effective Tools for Field and Horticultural Crops to Cope with Drought Stress: A Review. *Span J Agric Res* **2020**, *18*, e08R01, doi:10.5424/sjar/2020182-16181.
245. Mathur, P.; Roy, S. Nanosilica Facilitates Silica Uptake, Growth and Stress Tolerance in Plants. *Plant Physiology and Biochemistry* **2020**, *157*, 114-127, doi: 10.1016/j.plaphy.2020.10.011.
246. Mbinda, W., Mukami, A. A Review of Recent Advances and Future Directions in the Management of Salinity Stress in Finger Millet. *Frontiers in Plant Science* **2021**, *12*, 734798, doi: 10.3389/fpl.2021.734798.
247. McMillan, M.; Kallenbach, C. M., Whalen, J. K. Soybean Abiotic Stress Tolerance Is Improved by Beneficial Rhizobacteria in Biosolids-Amended Soil. *Applied Soil Ecology* **2022**, *174*, 104425, doi: 10.1016/j.apsoil.2022.104425.
248. Mhlongo, M.I.; Piater, L.A.; Madala, N.E.; Labuschagne, N.; Dubery, I.A. The Chemistry of Plant–Microbe Interactions in the Rhizosphere and the Potential for Metabolomics to Reveal Signaling Related to Defense Priming and Induced Systemic Resistance. *Front. Plant Sci.* **2018**, *9*, 112, doi:10.3389/fpls.2018.00112.
249. Mir, R. A., M. Nazir, Sabreena, S. Naik, S. Mukhtar, B. A. Ganai, and S. M. Zargar. Utilizing the Underutilized Plant Resources for Development of Life Style Foods: Putting Nutrigenomics to use. *Plant Physiology and Biochemistry* **2022** 171: 128-138. doi:10.1016/j.plaphy.2021.12.038.
250. Miransari, M.; Mahdavi, S.; Smith, D. The Biological Approaches of Altering the Growth and Biochemical Properties of Medicinal Plants under Salinity Stress. *Appl Microbiol Biotechnol* **2021**, *105*, 7201–7213, doi:10.1007/s00253-021-11552-z.
251. Mishra, S.; Huang, Y.; Li, J.; Wu, X.; Zhou, Z.; Lei, Q.; Bhatt, P.; Chen, S. Biofilm-Mediated Bioremediation Is a Powerful Tool for the Removal of Environmental Pollutants. *Chemosphere* **2022**, *294*, 133609, doi:10.1016/j.chemosphere.2022.133609.
252. Mishra, S.; Upadhyay, R.S.; Nautiyal, C.S. Unravelling the Beneficial Role of Microbial Contributors in Reducing the Allelopathic Effects of Weeds. *Appl Microbiol Biotechnol* **2013**, *97*, 5659-68, doi: 10.1007/s00253-013-4885-y.
253. Misra, S.; Chauhan, P.S. ACC Deaminase-Producing Rhizosphere Competent Bacillus Spp. Mitigate Salt Stress and Promote Zea Mays Growth by Modulating Ethylene Metabolism. *3 Biotech* **2020**, *10*, 119, doi:10.1007/s13205-020-2104-y.
254. MITRA, D., R. MONDAL, B. KHOSHRU, A. SENAPATI, T. K. RADHA, B. MAHAKUR, N. UNIYAL, et al. Actinobacteria-Enhanced Plant Growth, Nutrient Acquisition, and Crop Protection: Advances in Soil, Plant, and Microbial Multifactorial Interactions. *Pedosphere* **2022** 32 (1): 149-170. doi:10.1016/S1002-0160(21)60042-5.
255. Mohamed, H.I., El-Beltagi, H.E.-D.S., Abd-Elsalam, K.A. *Plant Growth-Promoting Microbes for Sustainable Biotic and Abiotic Stress Management*; Eds.; Springer International Publishing: Cham, 2021; ISBN 978-3-030-66586-9.
256. Mokrani, S.; Nabti, E.; Cruz, C. Recent Trends in Microbial Approaches for Soil Desalination. *Applied Sciences* **2022**, *12*, 3586, doi:10.3390/app12073586.
257. Moon, Y.-S. A Fruitful Decade of Bacterial ACC Deaminase Biotechnology: A Pragmatic Approach towards Abiotic Stress Relief in Plants. *Theor. Exp. Plant Physiol.* **2022**, *34*, 109-129, doi: 10.1007/s40626-022-00237-1.
258. Moradipour, M.; Saberi-Riseh, R.; Mohammadinejad, R.; Hosseini, A. Nano-Encapsulation of Plant Growth-Promoting Rhizobacteria and Their Metabolites Using Alginate-Silica Nanoparticles and Carbon Nanotube Improves UCB1 Pistachio Micropropagation. *Journal of Microbiology and Biotechnology* **2019**, *29*, 1096–1103, doi:10.4014/jmb.1903.03022.
259. Moreno, C.A.; Castillo, F.; González, A.; Bernal, D.; Jaimes, Y.; Chaparro, M.; González, C.; Rodriguez, F.; Restrepo, S.; Cotes, A.M. Biological and Molecular Characterization of the Response of Tomato Plants Treated with Trichoderma Koningiopsis. *Physiological and Molecular Plant Pathology* **2009**, *74*, 111–120, doi:10.1016/j.pmpp.2009.10.001.
260. Moshynets, O.V.; Babenko, L.M.; Rogalsky, S.P.; Iungin, O.S.; Foster, J.; Kosakivska, I.V.; Potters, G.; Spiers, A.J. Priming Winter Wheat Seeds with the Bacterial Quorum Sensing Signal N-Hexanoyl-L-Homoserine Lactone (C6-HSL) Shows Potential to Improve Plant Growth and Seed Yield. *PLoS ONE* **2019**, *14*, e0209460, doi:10.1371/journal.pone.0209460.
261. Mubarik, M.S.; Khan, S.H.; Sajjad, M.; Raza, A.; Hafeez, M.B.; Yasmeen, T.; Rizwan, M.; Ali, S.; Arif, M.S. A Manipulative Interplay between Positive and Negative Regulators of Phytohormones: A Way Forward for Improving Drought Tolerance in Plants. *Physiologia Plantarum* **2021**, *172*, 1269-1290, doi: 10.1111/ppl-13325.
262. Mujtaba, M.; Wang, D.; Carvalho, L.B.; Oliveira, J.L.; Espirito Santo Pereira, A. do; Sharif, R.; Jogaiah, S.; Paidi, M.K.; Wang, L.; Ali, Q.; et al. Nanocarrier-Mediated Delivery of MiRNA, RNAi, and CRISPR-Cas for Plant Protection: Current Trends and Future Directions. *ACS Agric. Sci. Technol.* **2021**, *1*, 417–435, doi:10.1021/acsagscitech.1c00146.
263. Mukherjee, S.; Chatterjee, N.; Sircar, A.; Maikap, S.; Singh, A.; Acharyya, S.; Paul, S. A Comparative Analysis of Heavy Metal Effects on Medicinal Plants. *Appl Biochem Biotechnol* **2022**, doi:10.1007/s12010-022-03938-0.
264. Mulani, R. Decoding the Mojo of Plant-Growth-Promoting Microbiomes. *Physiological and Molecular Plant Pathology* **2021**, 14.
265. Murali, M.; Gowtham, H.G.; Singh, S.B.; Shilpa, N.; Aiyaz, M.; Niranjana, S.R.; Amruthesh, K.N. Bio-Prospecting of ACC Deaminase Producing Rhizobacteria towards Sustainable Agriculture: A Special Emphasis on Abiotic Stress in Plants. *Applied Soil Ecology* **2021**, *168*, 104142, doi:10.1016/j.apsoil.2021.104142.
266. Nadarajah, K.; Abdul Rahman, N. S. N. Plant–Microbe Interaction: Aboveground to Belowground, from the Good to the Bad. *Int. J. Mol. Sci.* **2021**, *22*, 10388, doi: 10.3390/ijms221910388.
267. Nadeem, S.M.; Zahir, Z.A.; Naveed, M.; Ashraf, M. Microbial ACC-Deaminase: Prospects and Applications for Inducing Salt Tolerance in Plants. *Critical Reviews in Plant Sciences* **2010**, *29*, 360–393, doi:10.1080/07352689.2010.524518.
268. Nahakpam, S.; Shah, K.; Kundu, M.; Heikham, R.S. Role of Phytohormones as Master Regulators during the Abiotic Stress. In *Stress Tolerance in Horticultural Crops*; Elsevier, 2021; pp. 347–369 ISBN 978-0-12-822849-4.
269. Naik, K.; Mishra, S.; Srichandan, H.; Singh, P.K.; Sarangi, P.K. Plant Growth Promoting Microbes: Potential Link to Sustainable Agriculture and Environment. *Biocatalysis and Agricultural Biotechnology* **2019**, *21*, 101326, doi:10.1016/j.bcab.2019.101326.
270. Naing, A.H.; Campol, J.R.; Kang, H.; Xu, J.; Chung, M.Y.; Kim, C.K. Role of Ethylene Biosynthesis Genes in the Regulation of Salt Stress and Drought Stress Tolerance in Petunia. *Front. Plant Sci.* **2022**, *13*, 844449, doi:10.3389/fpls.2022.844449.
271. Naing, A.H.; Maung, T.-T.; Kim, C.K. The ACC Deaminase‐producing Plant Growth‐promoting Bacteria: Influences of Bacterial Strains and ACC Deaminase Activities in Plant Tolerance to Abiotic Stress. *Physiologia Plantarum* **2021**, *173*, 1992-2012, doi: 10.1111/ppl.13545.
272. Namal, J.; Smith, D.L. Microbial Derived Compounds, a Step Toward Enhancing Microbial Inoculants Technology for Sustainable Agriculture. *Frontiers in Microbiology* **2021**, *12*, 634807, doi: 10.3389/fmicb.2021.634807.
273. Nasrallah, A.K.; Kheder, A.A.; Kord, M.A.; Fouad, A.S.; El-Mogy, M.M.; Atia, M.A.M. Mitigation of Salinity Stress Effects on Broad Bean Productivity Using Calcium Phosphate Nanoparticles Application. *Horticulturae* **2022**, *8*, 75, doi:10.3390/horticulturae8010075.
274. Nath, A.; Sundaram, S. Microbiome Community Interactions With Social Forestry and Agroforestry. In *Microbial Services in Restoration Ecology*; Elsevier, 2020; pp. 71–82 ISBN 978-0-12-819978-7.
275. Nievas, F.L. 12. Quorum Sensing Signaling Molecules and Their Inhibitors in Legume-Associated Bacteria. In :Abiotic Stree and Legumes 2021, 277-289, doi:10.1016/B978-012-815355-0.00014-x.
276. Nonaka, S.; Ezura, H. Plantâ€“Agrobacterium Interaction Mediated by Ethylene and Super-Agrobacterium Conferring Efficient Gene Transfer. *Front. Plant Sci.* **2014**, *5*, doi:10.3389/fpls.2014.00681.
277. Nonaka, S.; Ezura, H. Plant–Agrobacterium Interaction Mediated by Ethylene and Super-Agrobacterium Conferring Efficient Gene Transfer. *Frontiers in Plant Science* **2014**, *5*, 681, doi: 10.3389/fpls.2014.00681.
278. Noor, I.; Sohail, H.; Sun, J.; Nawaz, M.A.; Li, G.; Hasanuzzaman, M.; Liu, J. Heavy Metal and Metalloid Toxicity in Horticultural Plants: Tolerance Mechanism and Remediation Strategies. *Chemosphere* **2022**, *303*, 135196, doi:10.1016/j.chemosphere.2022.135196.
279. Notununu, I.; Moleleki, L.; Roopnarain, A.; Adeleke, R. Effects of Plant Growth-Promoting Rhizobacteria on the Molecular Responses of Maize under Drought and Heat Stresses: A Review. *Pedosphere* **2022**, *32*, 90–106, doi:10.1016/S1002-0160(21)60051-6.
280. Nowruzi, B. et al. Plant-Cyanobacteria Interactions: Beneficial and Harmful Effects of Cyanobacterial Bioactive Compounds on Soil-Plant Systems and Subsequent Risk to Animal and Human Health.*Phytochemestry*  **2021**, *192*, 112959, doi: 10.1016/j.phytochem.2021. 112959.
281. Okubara, P.A.; Paulitz, T.C. Root Defense Responses to Fungal Pathogens: A Molecular Perspective. *Plant soil* **2005**, *274*, 215-226, doi: 10.1007/s22204-004-7328-9.
282. Omomowo, O.I.; Babalola, O.O. Constraints and Prospects of Improving Cowpea Productivity to Ensure Food, Nutritional Security and Environmental Sustainability. *Front. Plant Sci.* **2021**, *12*, 751731, doi:10.3389/fpls.2021.751731.
283. Onwe, R.O. Microbial Trehalose Boosts the Ecological Fitness of Biocontrol Agents, the Viability of Probiotics during Long-Term Storage and Plants Tolerance to Environmental-Driven Abiotic Stress. *Science of the Total Environment* **2022**, *806*, 150432, doi: 10.1016/j.scitotenv.2021. 150432.
284. Ortiz-Castro, R.; López-Bucio, J. Review: Phytostimulation and Root Architectural Responses to Quorum-Sensing Signals and Related Molecules from Rhizobacteria. *Plant Science* **2019**, *284*, 135–142, doi:10.1016/j.plantsci.2019.04.010.
285. Osaki, M., Tsuji, N., Foead, N., Rieley, J. *Tropical Peatland Eco-Management*; Eds.; Springer Singapore: Singapore, 2021; ISBN 978-981-334-653-6.
286. Otlewska, A.; Migliore, M.; Dybka-Stępień, K.; Manfredini, A.; Struszczyk-Świta, K.; Napoli, R.; Białkowska, A.; Canfora, L.; Pinzari, F. When Salt Meddles Between Plant, Soil, and Microorganisms. *Front. Plant Sci.* **2020**, *11*, 553087, doi:10.3389/fpls.2020.553087.
287. Ou, T.; Zhang, M.; Huang, Y.; Wang, L.; Wang, F.; Wang, R.; Liu, X.; Zhou, Z.; Xie, J.; Xiang, Z. Role of Rhizospheric Bacillus Megaterium HGS7 in Maintaining Mulberry Growth Under Extremely Abiotic Stress in Hydro-Fluctuation Belt of Three Gorges Reservoir. *Front. Plant Sci.* **2022**, *13*, 880125, doi:10.3389/fpls.2022.880125.
288. Oubohssaine, M., L. Sbabou, and J. Aurag. Native Heavy Metal-Tolerant Plant Growth Promoting Rhizobacteria Improves Sulla Spinosissima (L.) Growth in Post-Mining Contaminated Soils.*Microorganisms* **2022** 10 (5). doi:10.3390/microorganisms10050838.
289. Padder, S.A.; Mansoor, S.; Bhat, S.A.; Baba, T.R.; Rather, R.A.; Wani, S.M.; Popescu, S.M.; Sofi, S.; Aziz, M.A.; Hefft, D.I.; et al. Bacterial Endophyte Community Dynamics in Apple (Malus Domestica Borkh.) Germplasm and Their Evaluation for Scab Management Strategies. *JoF* **2021**, *7*, 923, doi:10.3390/jof7110923.
290. Pagano, L.; Rossi, R.; Paesano, L.; Marmiroli, N.; Marmiroli, M. MiRNA Regulation and Stress Adaptation in Plants. *Environmental and Experimental Botany* **2021**, *184*, 104369, doi:10.1016/j.envexpbot.2020.104369.
291. Paparu, P.; Dubois, T.; Coyne, D.; Viljoen, A. Defense-Related Gene Expression in Susceptible and Tolerant Bananas (Musa Spp.) Following Inoculation with Non-Pathogenic Fusarium Oxysporum Endophytes and Challenge with Radopholus Similis. *Physiological and Molecular Plant Pathology* **2007**, *71*, 149–157, doi:10.1016/j.pmpp.2007.12.001.
292. Paré, P.W.; Zhang, H.; Aziz, M.; Xie, X.; Kim, M.-S.; Shen, X.; Zhang, J. Beneficial Rhizobacteria Induce Plant Growth: Mapping Signaling Networks in Arabidopsis. In *Biocommunication in Soil Microorganisms*; Witzany, G., Ed.; Soil Biology; Springer Berlin Heidelberg: Berlin, Heidelberg, 2011; Vol. 23, pp. 403–412 ISBN 978-3-642-14511-7.
293. Patel, J.; Khandwal, D.; Choudhary, B.; Ardeshana, D.; Jha, R.K.; Tanna, B.; Yadav, S.; Mishra, A.; Varshney, R.K.; Siddique, K.H.M. Differential Physio-Biochemical and Metabolic Responses of Peanut (Arachis Hypogaea L.) under Multiple Abiotic Stress Conditions. *IJMS* **2022**, *23*, 660, doi:10.3390/ijms23020660.
294. Patel, M.; Patel, K.; Al-Keridis, L.A.; Alshammari, N.; Badraoui, R.; Elasbali, A.M.; Al-Soud, W.A.; Hassan, M.I.; Yadav, D.K.; Adnan, M. Cadmium-Tolerant Plant Growth-Promoting Bacteria Curtobacterium Oceanosedimentum Improves Growth Attributes and Strengthens Antioxidant System in Chili (Capsicum Frutescens). *Sustainability* **2022**, *14*, 4335, doi:10.3390/su14074335.
295. Pathy, A.; Ray, J.; Paramasivan, B. Biochar Amendments and Its Impact on Soil Biota for Sustainable Agriculture. *Biochar* **2020**, *2*, 287–305, doi:10.1007/s42773-020-00063-1.
296. Pereira, S.I.A.; Abreu, D.; Moreira, H.; Vega, A.; Castro, P.M.L. Plant Growth-Promoting Rhizobacteria (PGPR) Improve the Growth and Nutrient Use Efficiency in Maize (Zea Mays L.) under Water Deficit Conditions. *Heliyon* **2020**, *6*, e05106, doi:10.1016/j.heliyon.2020.e05106.
297. *Plant-Microbe Interaction: An Approach to Sustainable Agriculture*; Choudhary, D.K., Varma, A., Tuteja, N., Eds.; Springer Singapore: Singapore, 2016; ISBN 978-981-10-2853-3.
298. Polko, J.K.; Kieber, J.J. 1-Aminocyclopropane 1-Carboxylic Acid and Its Emerging Role as an Ethylene-Independent Growth Regulator. *Front. Plant Sci.* **2019**, *10*, 1602, doi:10.3389/fpls.2019.01602.
299. Poupin, M.J.; Greve, M.; Carmona, V.; Pinedo, I. A Complex Molecular Interplay of Auxin and Ethylene Signaling Pathways Is Involved in Arabidopsis Growth Promotion by Burkholderia Phytofirmans PsJN. *Front. Plant Sci.* **2016**, *7*, doi:10.3389/fpls.2016.00492.
300. Prakash, V.; Peralta-Videa, J.; Tripathi, D.K.; Ma, X.; Sharma, S. Recent Insights into the Impact, Fate and Transport of Cerium Oxide Nanoparticles in the Plant-Soil Continuum. *Ecotoxicology and Environmental Safety* **2021**, *221*, 112403, doi:10.1016/j.ecoenv.2021.112403.
301. *Precision Agriculture and Sustainable Crop Production*; 2020; ISBN 978-81-7019-667-9.
302. Priyam, A.; Yadav, N.; Reddy, P.M.; Afonso, L.O.B.; Schultz, A.G.; Singh, P.P. Uptake and Benefits of Biogenic Phosphorus Nanomaterials Applied via Fertigation to Japonica Rice ( *Taipei* 309) in Low- and High-Calcareous Soil Conditions. *ACS Agric. Sci. Technol.* **2022**, *2*, 462–476, doi:10.1021/acsagscitech.1c00244.
303. Pudake, R.N., Jain, U., Kole, C. *Biosensors in Agriculture: Recent Trends and Future Perspectives*; Eds.; Concepts and Strategies in Plant Sciences; Springer International Publishing: Cham, 2021; ISBN 978-3-030-66164-9.
304. Pudake, R.N., Sahu, B.B., Kumari, M., Sharma, A.K. *Omics Science for Rhizosphere Biology*; Eds.; Rhizosphere Biology; Springer Singapore: Singapore, 2021; ISBN 9789811608889.
305. Qadir, M.; Hussain, A.; Hamayun, M.; Shah, M.; Iqbal, A.; Irshad, M.; Ahmad, A.; Lodhi, M.A.; Lee, I.-J. Phytohormones Producing Acinetobacter Bouvetii P1 Mitigates Chromate Stress in Sunflower by Provoking Host Antioxidant Response. *Antioxidants* **2021**, *10*, 1868, doi:10.3390/antiox10121868.
306. Qi, G.; Ji, B.; Zhang, Y.; Huang, L.; Wang, J.; Gao, W. Microbiome-Based Screening and Co-Fermentation of Rhizospheric Microorganisms for Highly Ginsenoside Rg3 Production. *Microbiological Research* **2022**, *261*, 127054, doi:10.1016/j.micres.2022.127054.
307. Qi, W.-Y.; Li, Q.; Chen, H.; Liu, J.; Xing, S.-F.; Xu, M.; Yan, Z.; Song, C.; Wang, S.-G. Selenium Nanoparticles Ameliorate Brassica Napus L. Cadmium Toxicity by Inhibiting the Respiratory Burst and Scavenging Reactive Oxygen Species. *Journal of Hazardous Materials* **2021**, *417*, 125900, doi:10.1016/j.jhazmat.2021.125900.
308. Rahman, M.; Rahaman, S.; Islam, R.; Hossain, E.; Mithi, F.M.; Ahmed, M.; Saldías, M.; Akkol, E.K.; Sobarzo-Sánchez, E. Multifunctional Therapeutic Potential of Phytocomplexes and Natural Extracts for Antimicrobial Properties. *Antimicrobial Properties* **2021**, *10*, 1076, doi: 10.3390/antibiotics10091076.
309. Rai, K.K.; Pandey, N.; Meena, R.P.; Rai, S.P. Biotechnological Strategies for Enhancing Heavy Metal Tolerance in Neglected and Underutilized Legume Crops: A Comprehensive Review. *Ecotoxicology and Environmental Safety* **2021**, *208*, 111750, doi:10.1016/j.ecoenv.2020.111750.
310. Rai, N.; Rai, S.P.; Sarma, B.K. Prospects for Abiotic Stress Tolerance in Crops Utilizing Phyto- and Bio-Stimulants. *Front. Sustain. Food Syst.* **2021**, *5*, 754853, doi:10.3389/fsufs.2021.754853.
311. Rajput, V.D.; Minkina, T.; Feizi, M.; Kumari, A.; Khan, M.; Mandzhieva, S.; Sushkova, S.; El-Ramady, H.; Verma, K.K.; Singh, A.; et al. Effects of Silicon and Silicon-Based Nanoparticles on Rhizosphere Microbiome, Plant Stress and Growth. *Biology* **2021**, *10*, 791, doi:10.3390/biology10080791.
312. Raklami, A., A. Meddich, K. Oufdou, and M. Baslam. Plants—Microorganisms-Based Bioremediation for Heavy Metal Cleanup: Recent Developments, Phytoremediation Techniques, Regulation Mechanisms, and Molecular Responses. *International Journal of Molecular Sciences* **2022**, 23 (9). doi:10.3390/ijms23095031.
313. Rakshit, A., Singh, S.K., Abhilash, P.C., Biswas, A. *Soil Science: Fundamentals to Recent Advances*; Eds.; Springer Singapore: Singapore, 2021; ISBN 9789811609169.
314. Ramakrishnan, M.; Satish, L.; Kalendar, R.; Narayanan, M.; Kandasamy, S.; Sharma, A.; Emamverdian, A.; Wei, Q.; Zhou, M. The Dynamism of Transposon Methylation for Plant Development and Stress Adaptation. *IJMS* **2021**, *22*, 11387, doi:10.3390/ijms222111387.
315. Rane, J.; Singh, A.K.; George, P.; Govindasamy, V.; Cukkemane, A.; Raina, S.K.; Chavan, M.P.; Aher, L.; Sunoj, V.S.J.; Singh, N.P. Effect of Cow Urine-Based Bioformulations on Growth and Physiological Responses in Mungbean Under Soil Moisture Stress Conditions. *Proc. Natl. Acad. Sci., India, Sect. B Biol. Sci.* **2020**, *90*, 123–133, doi:10.1007/s40011-019-01088-8.
316. Rani, A.; Devi, P.; Jha, U.C.; Sharma, K.D.; Siddique, K.H.M.; Nayyar, H. Developing Climate-Resilient Chickpea Involving Physiological and Molecular Approaches With a Focus on Temperature and Drought Stresses. *Front. Plant Sci.* **2020**, *10*, 1759, doi:10.3389/fpls.2019.01759.
317. Rani, A.; Tokas, J.; Punia, H. Chapter - 9 Amelioration of Abiotic Stresses Using PGPRs. In book: Research Trends in Agriculture Science, 2020, pp 163-187, Chief Editor Dr. RK Naresch.
318. Ranjan, A.; Sinha, R.; Bala, M.; Pareek, A.; Singla-Pareek, S.L.; Singh, A.K. Silicon-Mediated Abiotic and Biotic Stress Mitigation in Plants: Underlying Mechanisms and Potential for Stress Resilient Agriculture. *Plant Physiology and Biochemistry* **2021**, *163*, 15–25, doi:10.1016/j.plaphy.2021.03.044.
319. Rauf, M.; Awais, M.; Ud-Din, A.; Ali, K.; Gul, H.; Rahman, M.M.; Hamayun, M.; Arif, M. Molecular Mechanisms of the 1-Aminocyclopropane-1-Carboxylic Acid (ACC) Deaminase Producing Trichoderma Asperellum MAP1 in Enhancing Wheat Tolerance to Waterlogging Stress. *Front. Plant Sci.* **2021**, *11*, 614971, doi:10.3389/fpls.2020.614971.
320. Ravanbakhsh, M.; Sasidharan, R.; Voesenek, L.A.C.J.; Kowalchuk, G.A.; Jousset, A. Microbial Modulation of Plant Ethylene Signaling: Ecological and Evolutionary Consequences. *Microbiome* **2018**, *6*, 52, doi:10.1186/s40168-018-0436-1.
321. Raza, A.; Razzaq, A.; Mehmood, S.S.; Hussain, M.A.; Wei, S.; He, H.; Zaman, Q.U.; Xuekun, Z.; Yong, C.; Hasanuzzaman, M. Omics: The Way Forward to Enhance Abiotic Stress Tolerance in *Brassica Napus* L. *GM Crops & Food* **2021**, *12*, 251–281, doi:10.1080/21645698.2020.1859898.
322. Raziq, A.; Wang, Y.; Din, A.M.U.; Sun, J.; Shu, S.; Guo, S. A Comprehensive Evaluation of Salt Tolerance in Tomato (Var. Ailsa Craig): Responses of Physiological and Transcriptional Changes in RBOH’s and ABA Biosynthesis and Signalling Genes. *Int. J. Mol. Sci.* **2022**, *23*, 1603, doi: 10.3390/ijms23031603.
323. Reginato, M.; Sgroy, V.; Llanes, A.; Cassán, F.; Luna, V. The American Halophyte Prosopis Strombulifera, a New Potential Source to Confer Salt Tolerance to Crops. In *Crop Production for Agricultural Improvement*; Ashraf, M., Öztürk, M., Ahmad, M.S.A., Aksoy, A., Eds.; Springer Netherlands: Dordrecht, 2012; pp. 115–143 ISBN 978-94-007-4115-7.
324. Rehaman, A.; Mishra, A.K.; Ferdose, A.; Per, T.S.; Hanief, M.; Jan, A.T.; Asgher, M. Melatonin in Plant Defense against Abiotic Stress. *Forests* **2021**, *12*, 1404, doi: 10.3390/f12101404.
325. Reva, O.N.; Swanevelder, D.Z.H.; Mwita, L.A.; Mwakilili, A.D.; Muzondiwa, D.; Joubert, M.; Chan, W.Y.; Lutz, S.; Ahrens, C.H.; Avdeeva, L.V.; et al. Genetic, Epigenetic and Phenotypic Diversity of Four Bacillus Velezensis Strains Used for Plant Protection or as Probiotics. *Front. Microbiol.* **2019**, *10*, 2610, doi:10.3389/fmicb.2019.02610.
326. Rey, T.; Dumas, B. Plenty Is No Plague: Streptomyces Symbiosis with Crops. *Trends in Plant Science* **2017**, *22*, 30–37, doi:10.1016/j.tplants.2016.10.008.
327. *Rhizosphere Biology: Interactions Between Microbes and Plants*; Gupta, V.V.S.R., Sharma, A.K., Eds.; Rhizosphere Biology; Springer Singapore: Singapore, 2021; ISBN 9789811561245.
328. Riaz, U.; Murtaza, G.; Anum, W.; Samreen, T.; Sarfraz, M.; Nazir, M.Z. Plant Growth-Promoting Rhizobacteria (PGPR) as Biofertilizers and Biopesticides. In *Microbiota and Biofertilizers*; Hakeem, K.R., Dar, G.H., Mehmood, M.A., Bhat, R.A., Eds.; Springer International Publishing: Cham, 2021; pp. 181–196 ISBN 978-3-030-48770-6.
329. Ribaudo, C.M.; Soledad Riva, D.; Gori, J.I. Identification of Endophytic Bacteria and Their Characterization as Biocontrol Agents against Tomato Southern Blight Disease. *Appli Micro Open Access* **2016**, *02*, doi:10.4172/2471-9315.1000123.
330. Riseh, R.S.; Ebrahimi-Zarandi, M.; Tamanadar, E.; Pour, M.M.; Thakur, V.K. Salinity Stress: Toward Sustainable Plant Strategies and Using Plant Growth-Promoting Rhizobacteria Encapsulation for Reducing It. *Sustainability* **2021**, *13*, 12758, doi: 10.3390/su132212758.
331. Riseh, R.S.; Ebrahimi-Zarandi, M.; Vazvani, M.G.; Skorik, Y.A. Reducing Drought Stress in Plants by Encapsulating Plant Growth-Promoting Bacteria with Polysaccharides. *Int. J. Mol. Sci.* **2021**, *22*, 12979, doi: 10.3390/ijms222312979.
332. Riyazuddin, R.; Verma, R.; Singh, K.; Nisha, N.; Keisham, M.; Bhati, K.K.; Kim, S.T.; Gupta, R. Ethylene: A Master Regulator of Salinity Stress Tolerance in Plants. *Biomolecules* **2020**, *10*, 959, doi:10.3390/biom10060959.
333. Rizvi, A.; Ahmed, B.; Khan, M.S.; El-Beltagi, H.S.; Umar, S.; Lee, J. Bioprospecting Plant Growth Promoting Rhizobacteria for Enhancing the Biological Properties and Phytochemical Composition of Medicinally Important Crops. *Molecules* **2022**, *27*, 1407, doi:10.3390/molecules27041407.
334. Rizwan, M.; Ali, S.; Rehman, M.Z. ur; Riaz, M.; Adrees, M.; Hussain, A.; Zahir, Z.A.; Rinklebe, J. Effects of Nanoparticles on Trace Element Uptake and Toxicity in Plants: A Review. *Ecotoxicology and Environmental Safety* **2021**, *221*, 112437, doi:10.1016/j.ecoenv.2021.112437.
335. Roy, S., Mathur, P., Chakraborty, A.P., Saha, S.P. *Plant Stress: Challenges and Management in the New Decade*; Eds.; Advances in Science, Technology & Innovation; Springer International Publishing: Cham, 2022; ISBN 978-3-030-95364-5.
336. Ryu, C.-M., Weisskopf, L., Piechulla, B. *Bacterial Volatile Compounds as Mediators of Airborne Interactions*; Eds.; Springer Singapore: Singapore, 2020; ISBN 9789811572920.
337. Saad, M.M.; Eida, A.A.; Hirt, H. Tailoring Plant-Associated Microbial Inoculants in Agriculture: A Roadmap for Successful Application. *Journal of Experimental Botany* **2020**, *71*, 3878–3901, doi:10.1093/jxb/eraa111.
338. Saeed, Q.; Xiukang, W.; Haider, F.U.; Kučerik, J.; Mumtaz, M.Z.; Holatko, J.; Naseem, M.; Kintl, A.; Ejaz, M.; Naveed, M.; et al. Rhizosphere Bacteria in Plant Growth Promotion, Biocontrol, and Bioremediation of Contaminated Sites: A Comprehensive Review of Effects and Mechanisms. *IJMS* **2021**, *22*, 10529, doi:10.3390/ijms221910529.
339. Saghafi, D.; Asgari Lajayer, B.; Ghorbanpour, M. Engineering Bacterial ACC Deaminase for Improving Plant Productivity under Stressful Conditions. In *Molecular Aspects of Plant Beneficial Microbes in Agriculture*; Elsevier, 2020; pp. 259–277 ISBN 978-0-12-818469-1.
340. Saha, L.; Tiwari, J.; Bauddh, K.; Ma, Y. Recent Developments in Microbe–Plant-Based Bioremediation for Tackling Heavy Metal-Polluted Soils. *Front. Microbiol.* **2021**, *12*, 731723, doi:10.3389/fmicb.2021.731723.
341. Saharan, B.; Nehra, V. Plant Growth Promoting Rhizobacteria: A Critical Review. *Life Sci Med Res* **2011**, *21*, 30.
342. Saia, S.; Fragasso, M.; De Vita, P.; Beleggia, R. Metabolomics Provides Valuable Insight for the Study of Durum Wheat: A Review. *J. Agric. Food Chem.* **2019**, *67*, 3069–3085, doi:10.1021/acs.jafc.8b07097.
343. Saikia, J.; Sarma, R.K.; Dhandia, R.; Yadav, A.; Bharali, R.; Gupta, V.K.; Saikia, R. Alleviation of Drought Stress in Pulse Crops with ACC Deaminase Producing Rhizobacteria Isolated from Acidic Soil of Northeast India. *Sci Rep* **2018**, *8*, 3560, doi:10.1038/s41598-018-21921-w.
344. Samanta, S.; Roychoudhury, A. Recent Trend in Nanoparticle Research in Regulating Arsenic Bioaccumulation and Mitigating Arsenic Toxicity in Plant Species. *J. Plant Biochem. Biotechnol.* **2021**, *30*, 793–812, doi:10.1007/s13562-021-00727-4.
345. Sandhya, V.; Ali, S.Z. Quantitative MRNA Analysis of Induced Genes in Maize Inoculated with Acinetobacter Baumannii Strain MZ30V92. *CBIOT* **2019**, *7*, 438–452, doi:10.2174/2211550108666190125114821.
346. Sani, Md.N.H.; Yong, J.W.H. Harnessing Synergistic Biostimulatory Processes: A Plausible Approach for Enhanced Crop Growth and Resilience in Organic Farming. *Biology* **2021**, *11*, 41, doi:10.3390/biology11010041.
347. Saradadevi, G.P.; Das, D.; Mangrauthia, S.K.; Mohapatra, S.; Chikkaputtaiah, C.; Roorkiwal, M.; Solanki, M.; Sundaram, R.M.; Chirravuri, N.N.; Sakhare, A.S.; et al. Genetic, Epigenetic, Genomic and Microbial Approaches to Enhance Salt Tolerance of Plants: A Comprehensive Review. *Biology* **2021**, *10*, 1255, doi: 10.3390/biology10121255.
348. Sardar, R.; Ahmed S., Yasin N. A. Titanium Dioxide Nanoparticles Mitigate Cadmium Toxicity in Coriandrum Sativum L. through Modulating Antioxidant System, Stress Markers and Reducing Cadmium Uptake. *Environmental Pollution* **2022**, *292*, 118373, doi: 10.1016/j.envpol.2021. 118373.
349. Sarma, H., Joshi, S.J., Prasad, R., Jampilek, J. *Biobased Nanotechnology for Green Applications*; Eds.; Nanotechnology in the Life Sciences; Springer International Publishing: Cham, 2021; ISBN 978-3-030-61984-8.
350. Sarraf, M.; Vishwakarma, K.; Kumar, V.; Arif, N.; Das, S.; Johnson, R.; Janeeshma, E.; Puthur, J.T.; Aliniaeifard, S.; Chauhan, D.K.; et al. Metal/Metalloid-Based Nanomaterials for Plant Abiotic Stress Tolerance: An Overview of the Mechanisms. *Plants* **2022**, *11*, 316, doi:10.3390/plants11030316.
351. Sayyed, R.Z., Arora, N.K., Reddy, M.S. *Plant Growth Promoting Rhizobacteria for Sustainable Stress Management: Volume 1: Rhizobacteria in Abiotic Stress Management*; Eds.; Microorganisms for Sustainability; Springer Singapore: Singapore, 2019; Vol. 12; ISBN 9789811365355.
352. Schenk, S.T.; Schikora, A. AHL-Priming Functions via Oxylipin and Salicylic Acid. *Front. Plant Sci.* **2015**, *5*, 784, doi:10.3389/fpls.2014.00784.
353. Schiavon, M.; Nardi, S.; dalla Vecchia, F.; Ertani, A. Selenium Biofortification in the 21st Century: Status and Challenges for Healthy Human Nutrition. *Plant Soil* **2020**, *453*, 245–270, doi:10.1007/s11104-020-04635-9.
354. Scotti, R.; D’Agostino, N.; Zaccardelli, M. Gene Expression Profiling of Tomato Roots Interacting with Pseudomonas Fluorescens Unravels the Molecular Reprogramming That Occurs during the Early Phases of Colonization. *Symbiosis* **2019**, *78*, 177–192, doi:10.1007/s13199-019-00611-9.
355. Sehrawat, A.; Deswal, R. S-Nitrosylation Analysis in *Brassica Juncea* Apoplast Highlights the Importance of Nitric Oxide in Cold-Stress Signaling. *J. Proteome Res.* **2014**, *13*, 2599–2619, doi:10.1021/pr500082u.
356. Sekhohola-Dlamini, L.M.; Keshinro, O.M.; Masudi, W.L.; Cowan, A.K. Elaboration of a Phytoremediation Strategy for Successful and Sustainable Rehabilitation of Disturbed and Degraded Land. *Minerals* **2022**, *12*, 111, doi:10.3390/min12020111.
357. Selem, E.; Tuncturk, R.; Nohutcu, L.; Tuncturk, M. EFFECTS OF RHIZOBACTERIA AND ALGAL SPECIES ON PHYSIOLOGICAL AND BIOCHEMICAL PARAMETERS IN CALENDULA OFFICINALIS L. UNDER DIFFERENT IRRIGATION REGIMES. *Journal of Elementology* **2022**, *27*, 87-97, doi:10.5601/jelem.2022.27.1.2173.
358. Selim, S.; Madany, M.M.Y.; Mohamed Reyad, A.; F Alowaiesh, B.; Hagagy, N.; Al-Sanea, M.M.; Alsharari, S.S.; AbdElgawad, H. Saccharomonospora Actinobacterium Alleviates Phytotoxic Hazards of Tungsten Nanoparticles on Legumes’ Growth and Osmotic Status. *Journal of Environmental Chemical Engineering* **2021**, *9*, 106395, doi:10.1016/j.jece.2021.106395.
359. Shahid, M.A.; Sarkhosh, A.; Khan, N.; Balal, R.M.; Ali, S.; Rossi, L.; Gómez, C.; Mattson, N.; Nasim, W.; Garcia-Sanchez, F. Insights into the Physiological and Biochemical Impacts of Salt Stress on Plant Growth and Development. *Agronomy* **2020**, *10*, 938, doi:10.3390/agronomy10070938.
360. Shao, J., W. Wu, F. Rasul, H. Munir, K. Huang, M. I. Awan, T. S. Albishi. Trehalose Induced Drought Tolerance in Plants: Physiological and Molecular Responses. *Notulae Botanicae Horti Agrobotanici Cluj-Napoca* **2022** 50 (1). doi:10.15835/nbha50112584.
361. Sharma, A.; Kapoor, D.; Wang, J.; Shahzad, B.; Kumar, V.; Bali, A.S.; Jasrotia, S.; Zheng, B.; Yuan, H.; Yan, D. Chromium Bioaccumulation and Its Impacts on Plants: An Overview. *Plants* **2020**, *9*, 100, doi:10.3390/plants9010100.
362. Sharma, A.; Shahzad, B.; Rehman, A.; Bhardwaj, R.; Landi, M.; Zheng, B. Response of Phenylpropanoid Pathway and the Role of Polyphenols in Plants under Abiotic Stress. *Molecules* **2019**, *24*, 2452, doi:10.3390/molecules24132452.
363. Sharma, G.; Mathur, V. Modulation of Insect-Induced Oxidative Stress Responses by Microbial Fertilizers in Brassica Juncea. *FEMS Microbiology Ecology* **2020**, *96*, fiaa040, doi:10.1093/femsec/fiaa040.
364. Sharma, K.; Gupta, S.; Thokchom, S.D.; Jangir, P.; Kapoor, R. Arbuscular Mycorrhiza-Mediated Regulation of Polyamines and Aquaporins During Abiotic Stress: Deep Insights on the Recondite Players. *Front. Plant Sci.* **2021**, *12*, 642101, doi:10.3389/fpls.2021.642101.
365. Sharma, P. Role and Significance of Biofilm-Forming Microbes in Phytoremediation -A Review. *Environmental Technology & Innovation* **2022**, *25*, 102182, doi:10.1016/j.eti.2021.102182.
366. Sharma, P.; Chouhan, R.; Bakshi, P.; Gandhi, S.G.; Kaur, R.; Sharma, A.; Bhardwaj, R. Amelioration of Chromium-Induced Oxidative Stress by Combined Treatment of Selected Plant-Growth-Promoting Rhizobacteria and Earthworms via Modulating the Expression of Genes Related to Reactive Oxygen Species Metabolism in Brassica Juncea. *Front. Microbiol.* **2022**, *13*, 802512, doi:10.3389/fmicb.2022.802512.
367. Sharma, P.; Pandey, A.K.; Kim, S.-H.; Singh, S.P.; Chaturvedi, P.; Varjani, S. Critical Review on Microbial Community during In-Situ Bioremediation of Heavy Metals from Industrial Wastewater. *Environmental Technology & Innovation* **2021**, *24*, 101826, doi:10.1016/j.eti.2021.101826.
368. Sharma, P.; Sharma, M.M.M.; Malik, A.; Vashisth, M.; Singh, D.; Kumar, R.; Singh, B.; Patra, A.; Mehta, S.; Pandey, V. Rhizosphere, Rhizosphere Biology, and Rhizospheric Engineering. In *Plant Growth-Promoting Microbes for Sustainable Biotic and Abiotic Stress Management*; Mohamed, H.I., El-Beltagi, H.E.-D.S., Abd-Elsalam, K.A., Eds.; Springer International Publishing: Cham, 2021; pp. 577–624 ISBN 978-3-030-66586-9.
369. Sharma, R.K.; Archana, G. Cadmium Minimization in Food Crops by Cadmium Resistant Plant Growth Promoting Rhizobacteria. *Applied Soil Ecology* **2016**, *107*, 66–78, doi:10.1016/j.apsoil.2016.05.009.
370. Sharma, S.; Rana, V.S.; Pawar, R.; Lakra, J.; Racchapannavar, V. Nanofertilizers for Sustainable Fruit Production: A Review. *Environ Chem Lett* **2021**, *19*, 1693–1714, doi:10.1007/s10311-020-01125-3.
371. Shelar, A.; Singh, A.V.; Maharjan, R.S.; Laux, P.; Luch, A.; Gemmati, D.; Tisato, V.; Singh, S.P.; Santilli, M.F.; Shelar, A.; et al. Sustainable Agriculture through Multidisciplinary Seed Nanopriming: Prospects of Opportunities and Challenges. *Cells* **2021**, *10*, 2428, doi:10.3390/cells10092428.
372. Shukla, P.S.; Mantin, E.G.; Adil, M.; Bajpai, S.; Critchley, A.T.; Prithiviraj, B. Ascophyllum Nodosum-Based Biostimulants: Sustainable Applications in Agriculture for the Stimulation of Plant Growth, Stress Tolerance, and Disease Management. *Front. Plant Sci.* **2019**, *10*, 655, doi:10.3389/fpls.2019.00655.
373. Siddikee, M.A.; Zereen, M.I.; Wu, M.; Zhang, W.; Dai, C.-C. Phomopsis Liquidambaris Reduces Ethylene Biosynthesis in Rice under Salt Stress via Inhibiting the Activity of 1-Aminocyclopropane-1-Carboxylate Deaminase. *Arch Microbiol* **2021**, *203*, 6215–6229, doi:10.1007/s00203-021-02588-w.
374. Siddikee, Md.A.; Chauhan, P.S.; Sa, T. Regulation of Ethylene Biosynthesis Under Salt Stress in Red Pepper (Capsicum Annuum L.) by 1-Aminocyclopropane-1-Carboxylic Acid (ACC) Deaminase-Producing Halotolerant Bacteria. *J Plant Growth Regul* **2012**, *31*, 265–272, doi:10.1007/s00344-011-9236-6.
375. Silva, S.; Dias, M.C.; Silva, A.M.S. Titanium and Zinc Based Nanomaterials in Agriculture: A Promising Approach to Deal with (A)Biotic Stresses? *Toxics* **2022**, *10*, 172, doi:10.3390/toxics10040172.
376. Simm, S.; Scharf, K.-D.; Jegadeesan, S.; Chiusano, M.L.; Firon, N.; Schleiff, E. Survey of Genes Involved in Biosynthesis, Transport, and Signaling of Phytohormones with Focus on *Solanum Lycopersicum*. *Bioinform Biol Insights* **2016**, *10*, 185-207, doi:10.4137/BBI.S38425.
377. Singh, A.; Tiwari, S.; Pandey, J.; Lata, C.; Singh, I.K. Role of Nanoparticles in Crop Improvement and Abiotic Stress Management. *Journal of Biotechnology* **2021**, *337*, 57–70, doi:10.1016/j.jbiotec.2021.06.022.
378. Singh, C.; Tiwari, S.; Singh, J. S.; Yadav, A. N. Microbes in agriculture and environmental development. CRC Press, 2020
379. Singh, D.; Chaudhary, P.; Taunk, J.; Singh, C.K.; Singh, D.; Tomar, R.S.S.; Aski, M.; Konjengbam, N.S.; Raje, R.S.; Singh, S.; et al. Fab Advances in Fabaceae for Abiotic Stress Resilience: From ‘Omics’ to Artificial Intelligence. *IJMS* **2021**, *22*, 10535, doi:10.3390/ijms221910535.
380. Singh, D.; Thapa, S.; Mahawar, H.; Kumar, D.; Geat, N.; Singh, S.K. Prospecting Potential of Endophytes for Modulation of Biosynthesis of Therapeutic Bioactive Secondary Metabolites and Plant Growth Promotion of Medicinal and Aromatic Plants. *Antonie van Leeuwenhoek* **2022**, *115*, 699–730, doi:10.1007/s10482-022-01736-6.
381. Singh, D.P., Singh, H.B., Prabha, R. *Plant-Microbe Interactions in Agro-Ecological Perspectives*; Eds.; Springer Singapore: Singapore, 2017; ISBN 978-981-10-5812-7.
382. Singh, H. B.; Sarma, B. K.; Keswani, C. *Advances in PGPR Research*; Ed.; CABI: Boston, MA, 2017; ISBN 978-1-78639-034-9.
383. Singh, H.B., Vaishnav, A., Sayyed, R.Z. *Antioxidants in Plant-Microbe Interaction*; Eds.; Springer Singapore: Singapore, 2021; ISBN 9789811613494.
384. Singh, K.; Chandra, R.; Purchase, D. Unraveling the Secrets of Rhizobacteria Signaling in Rhizosphere. *Rhizosphere* **2022**, *21*, 100484, doi:10.1016/j.rhisph.2022.100484.
385. Singh, R.; Misra, A.N.; Sharma, P. Safe, Efficient, and Economically Beneficial Remediation of Arsenic-Contaminated Soil: Possible Strategies for Increasing Arsenic Tolerance and Accumulation in Non-Edible Economically Important Native Plants. *Environ Sci Pollut Res* **2021**, *28*, 64113-64129, doi: 10.1007/s11356-021-14507-z.
386. Singh, S.; Ramakrishna, W. Application of CRISPR–Cas9 in Plant–Plant Growth-Promoting Rhizobacteria Interactions for next Green Revolution. *3 Biotech* **2021**, *11*, 492, doi:10.1007/s13205-021-03041-x.
387. Singha, L.P.; Sinha, N.; Pandey, P. Rhizoremediation Prospects of Polyaromatic Hydrocarbon Degrading Rhizobacteria, That Facilitate Glutathione and Glutathione-S-Transferase Mediated Stress Response, and Enhance Growth of Rice Plants in Pyrene Contaminated Soil. *Ecotoxicology and Environmental Safety* **2018**, *164*, 579–588, doi:10.1016/j.ecoenv.2018.08.069.
388. SkZ, A.; Vardharajula, S.; Vurukonda, S.S.K.P. Transcriptomic Profiling of Maize (Zea Mays L.) Seedlings in Response to Pseudomonas Putida Stain FBKV2 Inoculation under Drought Stress. *Ann Microbiol* **2018**, *68*, 331–349, doi:10.1007/s13213-018-1341-3.
389. Slama, H.B.; Cherif-Silini, H.; Bouket, A.C.; Silini, A.; Alenezi, F.N.; Luptakova, L.; Vallat, A.; Belbahri, L. Biotechnology and Bioinformatics of Endophytes in Biocontrol, Bioremediation, and Plant Growth Promotion. In *Endophytes: Mineral Nutrient Management, Volume 3*; Maheshwari, D.K., Dheeman, S., Eds.; Sustainable Development and Biodiversity; Springer International Publishing: Cham, 2021; Vol. 26, pp. 181–205 ISBN 978-3-030-65446-7.
390. Sofy, M.R.; Aboseidah, A.A.; Heneidak, S.A.; Ahmed, H.R. ACC Deaminase Containing Endophytic Bacteria Ameliorate Salt Stress in Pisum Sativum through Reduced Oxidative Damage and Induction of Antioxidative Defense Systems. *Environ Sci Pollut Res* **2021**, *28*, 40971–40991, doi:10.1007/s11356-021-13585-3.
391. Sohail, M.I. Iron Bio-Fortification and Heavy Metal/(Loid)s Contamination in Cereals: Successes, Issues, and Challenges. *Crop & Pasture Science* **2021**, doi: 10.1071/CP21771
392. Soni, R.; Keharia, H. Phytostimulation and Biocontrol Potential of Gram-Positive Endospore-Forming Bacilli. *Planta* **2021**, 254, 49, doi: 10.1007/s00425-021-03695-0.
393. Souri, Z.; Karimi, N.; Farooq, M.A.; Akhtar, J. Phytohormonal Signaling under Abiotic Stress. In *Plant Life Under Changing Environment*; Elsevier, 2020; pp. 397–466 ISBN 978-0-12-818204-8.
394. Spaepen, S.; Vanderleyden, J.; Okon, Y. Chapter 7 Plant Growth-Promoting Actions of Rhizobacteria. In *Advances in Botanical Research*; Elsevier, 2009; Vol. 51, pp. 283–320 ISBN 978-0-12-374834-8.
395. Subramaniam, G., Arumugam, S., Rajendran, V. *Plant Growth Promoting Actinobacteria*; Eds.; Springer Singapore: Singapore, 2016; ISBN 978-981-10-0705-7.
396. Subramanian, P.; Krishnamoorthy, R.; Chanratana, M.; Kim, K.; Sa, T. Expression of an Exogenous 1-Aminocyclopropane-1-Carboxylate Deaminase Gene in Psychrotolerant Bacteria Modulates Ethylene Metabolism and Cold Induced Genes in Tomato under Chilling Stress. *Plant Physiology and Biochemistry* **2015**, *89*, 18–23, doi:10.1016/j.plaphy.2015.02.003.
397. Sun, C.; Ali, K.; Yan, K.; Fiaz, S.; Dormatey, R.; Bi, Z.; Bai, J. Exploration of Epigenetics for Improvement of Drought and Other Stress Resistance in Crops: A Review. *Plants* **2021**, *10*, 1226, doi:10.3390/plants10061226.
398. Sun, L.; Di, D.; Li, G.; Kronzucker, H.J.; Shi, W. Spatio-Temporal Dynamics in Global Rice Gene Expression ( Oryza Sativa L.) in Response to High Ammonium Stress. *Journal of Plant Physiology* **2017**, *212*, 94–104, doi:10.1016/j.jplph.2017.02.006.
399. Sun, L.; Lei, P.; Wang, Q.; Ma, J.; Zhan, Y.; Jiang, K.; Xu, Z.; Xu, H. The Endophyte Pantoea Alhagi NX-11 Alleviates Salt Stress Damage to Rice Seedlings by Secreting Exopolysaccharides. *Front. Microbiol.* **2020**, *10*, 3112, doi:10.3389/fmicb.2019.03112.
400. Sun, L.; Song, F.; Guo, J.; Zhu, X.; Liu, S.; Liu, F.; Li, X. Nano-ZnO-Induced Drought Tolerance Is Associated with Melatonin Synthesis and Metabolism in Maize. *IJMS* **2020**, *21*, 782, doi:10.3390/ijms21030782.
401. Sun, S.-L.; Yang, W.-L.; Fang, W.-W.; Zhao, Y.-X.; Guo, L.; Dai, Y.-J. The Plant Growth-Promoting Rhizobacterium Variovorax Boronicumulans CGMCC 4969 Regulates the Level of Indole-3-Acetic Acid Synthesized from Indole-3-Acetonitrile. *Appl Environ Microbiol* **2018**, *84*, e00298-18, doi:10.1128/AEM.00298-18.
402. Sunita, K.; Mishra, I.; Mishra, J.; Prakash, J.; Arora, N.K. Secondary Metabolites From Halotolerant Plant Growth Promoting Rhizobacteria for Ameliorating Salinity Stress in Plants. *Front. Microbiol.* **2020**, *11*, 567768, doi:10.3389/fmicb.2020.567768.
403. *Sustainable Agriculture Reviews 41: Nanotechnology for Plant Growth and Development*; Hayat, S., Pichtel, J., Faizan, M., Fariduddin, Q., Eds.; Sustainable Agriculture Reviews; Springer International Publishing: Cham, 2020; Vol. 41; ISBN 978-3-030-33995-1.
404. Svoboda, T.; Parich, A.; Güldener, U.; Schöfbeck, D.; Twaruschek, K.; Václavíková, M.; Hellinger, R.; Wiesenberger, G.; Schuhmacher, R.; Adam, G. Biochemical Characterization of the Fusarium Graminearum Candidate ACC-Deaminases and Virulence Testing of Knockout Mutant Strains. *Front. Plant Sci.* **2019**, *10*, 1072, doi:10.3389/fpls.2019.01072.
405. Sytar, O.; Kumari, P.; Yadav, S.; Brestic, M.; Rastogi, A. Phytohormone Priming: Regulator for Heavy Metal Stress in Plants. *J Plant Growth Regul* **2019**, *38*, 739–752, doi:10.1007/s00344-018-9886-8.
406. Szerement, J.; Szatanik-Kloc, A.; Mokrzycki, J.; Mierzwa-Hersztek, M. Agronomic Biofortification with Se, Zn, and Fe: An Effective Strategy to Enhance Crop Nutritional Quality and Stress Defense—A Review. *J Soil Sci Plant Nutr* **2022**, *22*, 1129–1159, doi:10.1007/s42729-021-00719-2.
407. Tan, C., M. T. Kalhoro, Y. Faqir, J. Ma, M. D. Osei, and G. Khaliq. Climate-Resilient Microbial Biotechnology: A Perspective on Sustainable Agriculture. *Sustainability (Switzerland)* **2022**, 14 (9). doi:10.3390/su14095574.
408. Tanveer, Y.; Yasmin, H.; Nosheen, A.; Ali, S.; Ahmad, A. Ameliorative Effects of Plant Growth Promoting Bacteria, Zinc Oxide Nanoparticles and Oxalic Acid on Luffa Acutangula Grown on Arsenic Enriched Soil. *Environmental Pollution* **2022**, *300*, 118889, doi:10.1016/j.envpol.2022.118889.
409. Tao, J.-J.; Chen, H.-W.; Ma, B.; Zhang, W.-K.; Chen, S.-Y.; Zhang, J.-S. The Role of Ethylene in Plants Under Salinity Stress. *Front. Plant Sci.* **2015**, *6*, 1059, doi:10.3389/fpls.2015.01059.
410. Thangaraj, K.; Li, J.; Mei, H.; Hu, S.; Han, R.; Zhao, Z.; Chen, X.; Li, X.; Kamatchi Reddiar, D. Mycorrhizal Colonization Enhanced *Sorghum Bicolor* Tolerance under Soil Water Deficit Conditions by Coordination of Proline and Reduced Glutathione (GSH). *J. Agric. Food Chem.* **2022**, *70*, 4243–4255, doi:10.1021/acs.jafc.1c07184.
411. Thangavel, P.; Anjum, N.A.; Muthukumar, T.; Sridevi, G.; Vasudhevan, P.; Maruthupandian, A. Arbuscular Mycorrhizae: Natural Modulators of Plant–Nutrient Relation and Growth in Stressful Environments. *Arch Microbiol* **2022**, *204*, 264, doi:10.1007/s00203-022-02882-1.
412. Theocharis, A.I. Physiological Responses of Burkholderia Phytofirmans Strain PsJN Colonized Plantlets of Grapevine (Vitis Vinifera L.) to Low Non-Freezing Temperatures. Doctoral Dissertation **2010**, p 226.
413. Tian, L.; Shen, J.; Sun, G.; Wang, B.; Ji, R.; Zhao, L. Foliar Application of SiO _2_ Nanoparticles Alters Soil Metabolite Profiles and Microbial Community Composition in the Pakchoi ( *Brassica Chinensis* L.) Rhizosphere Grown in Contaminated Mine Soil. *Environ. Sci. Technol.* **2020**, *54*, 13137–13146, doi:10.1021/acs.est.0c03767.
414. Tiwari, S.; Lata, C.; Chauhan, P.S.; Nautiyal, C.S. Pseudomonas Putida Attunes Morphophysiological, Biochemical and Molecular Responses in Cicer Arietinum L. during Drought Stress and Recovery. *Plant Physiology and Biochemistry* **2016**, *99*, 108–117, doi:10.1016/j.plaphy.2015.11.001.
415. Tiwari, S.; Lata, C.; Chauhan, P.S.; Prasad, V.; Prasad, M. A Functional Genomic Perspective on Drought Signalling and Its Crosstalk with Phytohormone-Mediated Signalling Pathways in Plants. *Current Genomics* **2017**, *18*, 469-482, doi:10.2174/1389202918666170605083319.
416. Toubali, S.; Ait-El-Mokhtar, M.; Boutasknit, A.; Anli, M.; Ait-Rahou, Y.; Benaffari, W.; Ben-Ahmed, H.; Mitsui, T.; Baslam, M.; Meddich, A. Root Reinforcement Improved Performance, Productivity, and Grain Bioactive Quality of Field-Droughted Quinoa (Chenopodium Quinoa). *Front. Plant Sci.* **2022**, *13*, 860484, doi:10.3389/fpls.2022.860484.
417. Tran, T.M.; Ameye, M.; Devlieghere, F.; De Saeger, S.; Eeckhout, M.; Audenaert, K. Streptomyces Strains Promote Plant Growth and Induce Resistance Against Fusarium Verticillioides via Transient Regulation of Auxin Signaling and Archetypal Defense Pathways in Maize Plants. *Front. Plant Sci.* **2021**, *12*, 755733, doi:10.3389/fpls.2021.755733.
418. Tripathi, V., Kumar, P., Tripathi, P., Kishore, A., Kamle, M. *Microbial Genomics in Sustainable Agroecosystems: Volume 2*; Eds.; Springer Singapore: Singapore, 2019; ISBN 978-981-329-859-0.
419. Tsukanova, K.A.; Сhеbоtаr, V.К.; Meyer, J.J.M.; Bibikova, T.N. Effect of Plant Growth-Promoting Rhizobacteria on Plant Hormone Homeostasis. *South African Journal of Botany* **2017**, *113*, 91–102, doi:10.1016/j.sajb.2017.07.007.
420. Tyagi, P. Comparative Study of the Pharmacological, Phytochemical and Biotechnological Aspects of Tribulusterrestris Linn. and Pedalium Murex Linn: An Overview., *Acta Ecologica Sinica* **2021**, doi: 10.1016/j.chnaes.2021.07.008. Article in press.
421. Vaishnav, A.; Kumar, R.; Singh, H.B.; Sarma, B.K. Extending the Benefits of PGPR to Bioremediation of Nitrile Pollution in Crop Lands for Enhancing Crop Productivity. *Science of The Total Environment* **2022**, *826*, 154170, doi:10.1016/j.scitotenv.2022.154170.
422. van Loon, L.C.; Geraats, B.P.J.; Linthorst, H.J.M. Ethylene as a Modulator of Disease Resistance in Plants. *Trends in Plant Science* **2006**, *11*, 184–191, doi:10.1016/j.tplants.2006.02.005.
423. Vanderstraeten, L.; Van Der Straeten, D. Accumulation and Transport of 1-Aminocyclopropane-1-Carboxylic Acid (ACC) in Plants: Current Status, Considerations for Future Research and Agronomic Applications. *Front. Plant Sci.* **2017**, *8*, 38, doi:10.3389/fpls.2017.00038.
424. Vardharajula, S.; SkZ, A.; Krishna Prasad Vurukonda, S.S.; Shrivastava, M. Plant Growth Promoting Endophytes and Their Interaction with Plants to Alleviate Abiotic Stress. *CBIOT* **2017**, *6*, 252-263, doi:10.2174/2211550106666161226154619.
425. Varma, A., Choudhary, D.K. *Mycorrhizosphere and Pedogenesis*; Eds.; Springer Singapore: Singapore, 2019; ISBN 9789811364792.
426. Varma, A., Tripathi, S., Prasad, R. *Plant Biotic Interactions: State of the Art*; Eds.; Springer International Publishing: Cham, 2019; ISBN 978-3-030-26656-1.
427. Vazquez, A.; Zawoznik, M.; Benavides, M.P.; Groppa, M.D. Azospirillum Brasilense Az39 Restricts Cadmium Entrance into Wheat Plants and Mitigates Cadmium Stress. *Plant Science* **2021**, *312*, 111056, doi:10.1016/j.plantsci.2021.111056.
428. Vilchez, S.; Manzanera, M. Biotechnological Uses of Desiccation-Tolerant Microorganisms for the Rhizoremediation of Soils Subjected to Seasonal Drought. *Appl Microbiol Biotechnol* **2011**, *91*, 1297–1304, doi:10.1007/s00253-011-3461-6.
429. Vishwakarma, K.; Kumar, N.; Shandilya, C.; Mohapatra, S.; Bhayana, S.; Varma, A. Revisiting Plant–Microbe Interactions and Microbial Consortia Application for Enhancing Sustainable Agriculture: A Review. *Front. Microbiol.* **2020**, *11*, 560406, doi:10.3389/fmicb.2020.560406.
430. Vishwakarma, K.; Singh, V.P.; Prasad, S.M.; Chauhan, D.K.; Tripathi, D.K.; Sharma, S. Silicon and Plant Growth Promoting Rhizobacteria Differentially Regulate AgNP-Induced Toxicity in Brassica Juncea: Implication of Nitric Oxide. *Journal of Hazardous Materials* **2020**, *390*, 121806, doi:10.1016/j.jhazmat.2019.121806.
431. Vo, K.T.X.; Rahman, M.M.; Rahman, M.M.; Trinh, K.T.T.; Kim, S.T.; Jeon, J.-S. Proteomics and Metabolomics Studies on the Biotic Stress Responses of Rice: An Update. *Rice* **2021**, *14*, 30, doi:10.1186/s12284-021-00461-4.
432. Vurukonda, S.S.K.P.; Vardharajula, S.; Shrivastava, M.; SkZ, A. Enhancement of Drought Stress Tolerance in Crops by Plant Growth Promoting Rhizobacteria. *Microbiological Research* **2016**, *184*, 13–24, doi:10.1016/j.micres.2015.12.003.
433. Vwioko, E.; Adinkwu, O.; El-Esawi, M.A. Comparative Physiological, Biochemical, and Genetic Responses to Prolonged Waterlogging Stress in Okra and Maize Given Exogenous Ethylene Priming. *Front. Physiol.* **2017**, *8*, 632, doi:10.3389/fphys.2017.00632.
434. Wang, A.; Hua, J.; Wang, Y.; Zhang, G.; Luo, S. Stereoisomers of Nonvolatile Acetylbutanediol Metabolites Produced by *Bacillus Velezensis* WRN031 Improved Root Elongation of Maize and Rice. *J. Agric. Food Chem.* **2020**, *68*, 6308–6315, doi:10.1021/acs.jafc.0c01352.
435. Wang, J.; Nan, Z.; Christensen, M.J.; Zhang, X.; Tian, P.; Zhang, Z.; Niu, X.; Gao, P.; Chen, T.; Ma, L. Effect of *Epichloë Gansuensis* Endophyte on the Nitrogen Metabolism, Nitrogen Use Efficiency, and Stoichiometry of *Achnatherum Inebrians* under Nitrogen Limitation. *J. Agric. Food Chem.* **2018**, *66*, 4022–4031, doi:10.1021/acs.jafc.7b06158.
436. Wang, L.; Huang, X.; Li, J.; Huang, J.; Bao, S.; He, C.; Zhang, M.; Xiang, T. Metabolites of Zearalenone and Phytohormones Secreted by Endophytic Fungus Strain TH15 Regulating the Root Development in Tetrastigma Hemsleyanum. *Plant Cell Tiss Organ Cult* **2022**, 1-12 doi:10.1007/s11240-022-02321-5.
437. Wang, X.; Zeng, X.; Luo, L.; Chen, X.; Yan, H.; Xie, Z.; Zhou, Y. Plant Growth-Promoting Activity of *Herbaspirillum Aquaticum* ZXN111 on the Zijuan Tea Plant ( *Camellia Sinensis* Var. *Assamica* ). *J. Agric. Food Chem.* **2022**, *70*, 3757–3764, doi:10.1021/acs.jafc.1c08178.
438. Wang, Z.; Chen, X.; Zhong, T.; Li, B.; Yang, Q.; Du, M.; Zalán, Z.; Kan, J. Bioeffector *Pseudomonas Fluorescens* ZX Elicits Biosynthesis and Accumulation of Functional Ingredients in Citrus Fruit Peel: A Promising Strategy for a More Sustainable Crop. *J. Agric. Food Chem.* **2021**, *69*, 13810–13820, doi:10.1021/acs.jafc.1c05709.
439. Wang, Z.; Yue, L.; Dhankher, O.P.; Xing, B. Nano-Enabled Improvements of Growth and Nutritional Quality in Food Plants Driven by Rhizosphere Processes. *Environment International* **2020**, *142*, 105831, doi:10.1016/j.envint.2020.105831.
440. Wasternack, C. Action of Jasmonates in Plant Stress Responses and Development — Applied Aspects. *Biotechnology Advances* **2014**, *32*, 31–39, doi:10.1016/j.biotechadv.2013.09.009.
441. *Wheat Production in Changing Environments: Responses, Adaptation and Tolerance*; Hasanuzzaman, M., Nahar, K., Hossain, Md.A., Eds.; Springer Singapore: Singapore, 2019; ISBN 9789811368820.
442. Wu, W.; Chen, W.; Liu, S.; Wu, J.; Zhu, Y.; Qin, L.; Zhu, B. Beneficial Relationships Between Endophytic Bacteria and Medicinal Plants. *Front. Plant Sci.* **2021**, *12*, 646146, doi:10.3389/fpls.2021.646146.
443. Xie, F.; Pathom-aree, W. Actinobacteria From Desert: Diversity and Biotechnological Applications. *Front. Microbiol.* **2021**, *12*, 765531, doi:10.3389/fmicb.2021.765531.
444. Xie, G.; Liu, N.; Song, Y.; Luo, Z.; Zhang, M. Integrated Analysis of Transcriptomics and Small RNA Reveals That 1-MCP Inhibits Lignocellulosis of Fresh Common Beans by Regulating Secondary Cell Wall Metabolism. *Postharvest Biology and Technology* **2022**, *186*, 111836, doi:10.1016/j.postharvbio.2022.111836.
445. Xie, J. Role of Rhizospheric Bacillus Megaterium HGS7 in Maintaining Mulberry Growth Under Extremely Abiotic Stress in Hydro-Fluctuation Belt of Three Gorges Reservoir. *Frontiers in Plant Science* **2022**, *13*, 880125, doi: 10.3389/fpls.2022.880125.
446. Xing, Y.-X.; Li, Y.-R. Differential Protein Expression Analysis of Two Sugarcane Varieties in Response to Diazotrophic Plant Growth-Promoting Endophyte Enterobacter Roggenkampii ED5. *Frontiers in Plant Science* **2021**, *12*, 727741, doi: 10.3389/fpls.2021.727741.
447. Xu, R.; Guo, Y.; Peng, S.; Liu, J.; Li, P.; Jia, W.; Zhao, J. Molecular Targets and Biological Functions of CAMP Signaling in Arabidopsis. *Biomolecules* **2021**, *11*, 688, doi:10.3390/biom11050688.
448. Xu, Z.-M.; Zhang, Y.-X.; Wang, L.; Liu, C.-G.; Sun, W.-M.; Wang, Y.-F.; Long, S.-X.; He, X.-T.; Lin, Z.; Liang, J.-L.; et al. Rhizobacteria Communities Reshaped by Red Mud Based Passivators Is Vital for Reducing Soil Cd Accumulation in Edible Amaranth. *Science of The Total Environment* **2022**, *826*, 154002, doi:10.1016/j.scitotenv.2022.154002.
449. Yadav, A.N., Rastegari, A.A., Yadav, N., Kour, D. *Advances in Plant Microbiome and Sustainable Agriculture: Functional Annotation and Future Challenges*; Eds.; Microorganisms for Sustainability; Springer Singapore: Singapore, 2020; Vol. 20; ISBN 9789811532030.
450. Yadav, A.N., *Soil Microbiomes for Sustainable Agriculture: Functional Annotation*; Ed.; Sustainable Development and Biodiversity; Springer International Publishing: Cham, 2021; Vol. 27; ISBN 978-3-030-73506-7.
451. Yadav, B.; Chhaya; Dubey, R.; Gnanasekaran, P.; Narayan, O.P. OMICS Approaches towards Understanding Plant’s Responses to Counterattack Heavy Metal Stress: An Insight into Molecular Mechanisms of Plant Defense. *Plant Gene* **2021**, *28*, 100333, doi:10.1016/j.plgene.2021.100333.
452. Yadav, M.R.; Choudhary, M.; Singh, J.; Lal, M.K.; Jha, P.K.; Udawat, P.; Gupta, N.K.; Rajput, V.D.; Garg, N.K.; Maheshwari, C.; et al. Impacts, Tolerance, Adaptation, and Mitigation of Heat Stress on Wheat under Changing Climates. *IJMS* **2022**, *23*, 2838, doi:10.3390/ijms23052838.
453. Yadav, V.K.; Raghav, M.; Sharma, S.K.; Bhagat, N. Rhizobacteriome: Promising Candidate for Conferring Drought Tolerance in Crops. *J. Pure Appl. Microbiol.* **2020**, *14*, 73–92, doi:10.22207/JPAM.14.1.10.
454. Yakhin, O.I.; Lubyanov, A.A.; Yakhin, I.A.; Brown, P.H. Biostimulants in Plant Science: A Global Perspective. *Front. Plant Sci.* **2017**, *7*, 2049, doi:10.3389/fpls.2016.02049.
455. Yan, S.; Ren, T.; Wan Mahari, W.A.; Feng, H.; Xu, C.; Yun, F.; Waiho, K.; Wei, Y.; Lam, S.S.; Liu, G. Soil Carbon Supplementation: Improvement of Root-Surrounding Soil Bacterial Communities, Sugar and Starch Content in Tobacco (N. Tabacum). *Science of The Total Environment* **2022**, *802*, 149835, doi:10.1016/j.scitotenv.2021.149835.
456. Yang, C.; Han, N.; Inoue, C.; Yang, Y.-L.; Nojiri, H.; Ho, Y.-N.; Chien, M.-F. Rhizospheric Plant-Microbe Synergistic Interactions Achieve Efficient Arsenic Phytoextraction by Pteris Vittata. *Journal of Hazardous Materials* **2022**, *434*, 128870, doi:10.1016/j.jhazmat.2022.128870.
457. Yang, H.; Wang, C.; Chen, F.; Yue, L.; Cao, X.; Li, J.; Zhao, X.; Wu, F.; Wang, Z.; Xing, B. Foliar Carbon Dot Amendment Modulates Carbohydrate Metabolism, Rhizospheric Properties and Drought Tolerance in Maize Seedling. *Science of The Total Environment* **2022**, *809*, 151105, doi:10.1016/j.scitotenv.2021.151105.
458. Yang, Y.; Liu, L.; Singh, R.P.; Meng, C.; Ma, S.; Jing, C.; Li, Y.; Zhang, C. Nodule and Root Zone Microbiota of Salt-Tolerant Wild Soybean in Coastal Sand and Saline-Alkali Soil. *Front. Microbiol.* **2020**, *11*, 2178, doi:10.3389/fmicb.2020.523142.
459. Yates, P.S.; Roberson, J.; Ramsue, L.K.; Song, B.-H. Bridging the Gaps between Plant and Human Health: A Systematic Review of Soyasaponins. *J. Agric. Food Chem.* **2021**, *69*, 14387–14401, doi:10.1021/acs.jafc.1c04819.
460. Yim, W.; Seshadri, S.; Kim, K.; Lee, G.; Sa, T. Ethylene Emission and PR Protein Synthesis in ACC Deaminase Producing Methylobacterium Spp. Inoculated Tomato Plants (Lycopersicon Esculentum Mill.) Challenged with Ralstonia Solanacearum under Greenhouse Conditions. *Plant Physiology and Biochemistry* **2013**, *67*, 95–104, doi:10.1016/j.plaphy.2013.03.002.
461. Yim, W.J.; Kim, K.Y.; Lee, Y.W.; Sundaram, S.P.; Lee, Y.; Sa, T.M. Real Time Expression of ACC Oxidase and PR-Protein Genes Mediated by Methylobacterium Spp. in Tomato Plants Challenged with Xanthomonas Campestris Pv. Vesicatoria. *Journal of Plant Physiology* **2014**, *171*, 1064–1075, doi:10.1016/j.jplph.2014.03.009.
462. Yue, L.; Uwaremwe, C.; Tian, Y.; Liu, Y.; Zhao, X.; Zhou, Q.; Wang, Y.; Zhang, Y.; Liu, B.; Cui, Z.; et al. Bacillus Amyloliquefaciens Rescues Glycyrrhizic Acid Loss Under Drought Stress in Glycyrrhiza Uralensis by Activating the Jasmonic Acid Pathway. *Front. Microbiol.* **2022**, *12*, 798525, doi:10.3389/fmicb.2021.798525.
463. Yue, Z.; Chen, Y.; Hao, Y.; Wang, C.; Zhang, Z.; Chen, C.; Liu, H.; Liu, Y.; Li, L.; Sun, Z. Bacillus Sp. WR12 Alleviates Iron Deficiency in Wheat via Enhancing Siderophore- and Phenol-Mediated Iron Acquisition in Roots. *Plant Soil* **2022**, *471*, 247–260, doi:10.1007/s11104-021-05218-y.
464. Zadravec, M., K. Markov, T. Lešić, J. Frece, D. Petrović, and J. Pleadin. Biocontrol Methods in Avoidance and Downsizing of Mycotoxin Contamination of Food Crops. *Processes* **2022** 10 (4). doi:10.3390/pr10040655.
465. Zaheer, M.S.; Raza, M.A.S.; Saleem, M.F.; Erinle, K.O.; Iqbal, R.; Ahmad, S. Effect of Rhizobacteria and Cytokinins Application on Wheat Growth and Yield under Normal vs Drought Conditions. *Communications in Soil Science and Plant Analysis* **2019**, *50*, 2521–2533, doi:10.1080/00103624.2019.1667376.
466. Zaib, S.; Ahmad, I.; Shakeel, S.N. MODULATION OF BARLEY (Hordeum Vulgare) DEFENSE AND HORMONAL. *Pakistan journal of Agricultural Sciences* **2020**, *57*, 1469-1481; doi: 10.21162/PAKAS/20.9373.
467. Zboralski, A.; Filion, M. Genetic Factors Involved in Rhizosphere Colonization by Phytobeneficial Pseudomonas Spp. *Computational and Structural Biotechnology Journal* **2020**, *18*, 3539–3554, doi:10.1016/j.csbj.2020.11.025.
468. Zeng, Q.; Ding, X.; Wang, J.; Han, X.; Iqbal, H.M.N.; Bilal, M. Insight into Soil Nitrogen and Phosphorus Availability and Agricultural Sustainability by Plant Growth-Promoting Rhizobacteria. *Environ Sci Pollut Res* **2022**, *29*, 45089–45106, doi:10.1007/s11356-022-20399-4.
469. Zhang, H.; Xie, J.; Fu, Y.; Cheng, J.; Qu, Z.; Zhao, Z.; Cheng, S.; Chen, T.; Li, B.; Wang, Q.; et al. A 2-Kb Mycovirus Converts a Pathogenic Fungus into a Beneficial Endophyte for Brassica Protection and Yield Enhancement. *Molecular Plant* **2020**, *13*, 1420–1433, doi:10.1016/j.molp.2020.08.016.
470. Zhang, H.; Xie, X.; Kim, M.-S.; Kornyeyev, D.A.; Holaday, S.; Paré, P.W. Soil Bacteria Augment Arabidopsis Photosynthesis by Decreasing Glucose Sensing and Abscisic Acid Levels *in Planta*: *Photosynthesis Augmentation by Soil Bacteria*. *The Plant Journal* **2008**, *56*, 264–273, doi:10.1111/j.1365-313X.2008.03593.x.
471. Zhang, J.; Sun, X. Recent Advances in Polyphenol Oxidase-Mediated Plant Stress Responses. *Phytochemistry* **2021**, *181*, 112588, doi:10.1016/j.phytochem.2020.112588.
472. Zhang, L.; Zou, D.; Zeng, N.; Li, L.; Xiao, Z. Slaked Lime Improves Growth, Antioxidant Capacity and Reduces Cd Accumulation of Peanut (Arachis Hypogaea L.) under Cd Stress. *Sci Rep* **2022**, *12*, 4388, doi:10.1038/s41598-022-08339-1.
473. Zhang, S.; Gan, Y.; Xu, B. Mechanisms of the IAA and ACC-Deaminase Producing Strain of Trichoderma Longibrachiatum T6 in Enhancing Wheat Seedling Tolerance to NaCl Stress. *BMC Plant Biol* **2019**, *19*, 22, doi:10.1186/s12870-018-1618-5.
474. Zhang, Y.; Huber, D.J.; Hu, M.; Jiang, G.; Gao, Z.; Xu, X.; Jiang, Y.; Zhang, Z. Delay of Postharvest Browning in Litchi Fruit by Melatonin via the Enhancing of Antioxidative Processes and Oxidation Repair. *J. Agric. Food Chem.* **2018**, *66*, 7475–7484, doi:10.1021/acs.jafc.8b01922.
475. Zhao, L.; Chen, S.; Tan, X.; Yan, X.; Zhang, W.; Huang, Y.; Ji, R.; White, J.C. Environmental Implications of MoS2 Nanosheets on Rice and Associated Soil Microbial Communities. *Chemosphere* **2022**, *291*, 133004, doi:10.1016/j.chemosphere.2021.133004.
476. Zhao, Y.; Cartabia, A.; Lalaymia, I.; Declerck, S. Arbuscular Mycorrhizal Fungi and Production of Secondary Metabolites in Medicinal Plants. *Mycorrhiza* **2022**, *32*, 221–256, doi:10.1007/s00572-022-01079-0.
477. Zhou, C.; Ge, N.; Guo, J.; Zhu, L.; Ma, Z.; Cheng, S.; Wang, J. *Enterobacter Asburiae* Reduces Cadmium Toxicity in Maize Plants by Repressing Iron Uptake-Associated Pathways. *J. Agric. Food Chem.* **2019**, *67*, 10126–10136, doi:10.1021/acs.jafc.9b03293.
478. Zhou, C.; Zhu, L.; Guo, J.; Xiao, X.; Ma, Z.; Wang, J. *Bacillus Subtilis* STU6 Ameliorates Iron Deficiency in Tomato by Enhancement of Polyamine-Mediated Iron Remobilization. *J. Agric. Food Chem.* **2019**, *67*, 320–330, doi:10.1021/acs.jafc.8b05851.
479. Zhu, L.; Qian, N.; Sun, Y.; Lu, X.; Duan, H.; Qian, L. Pseudomonas Fluorescens DN16 Enhances Cucumber Defense Responses Against the Necrotrophic Pathogen Botrytis Cinerea by Regulating Thermospermine Catabolism. *Front. Plant Sci.* **2021**, *12*, 645338, doi:10.3389/fpls.2021.645338.
480. Zhu, Y.-X.; Gong, H.-J.; Yin, J.-L. Role of Silicon in Mediating Salt Tolerance in Plants: A Review. *Plants* **2019**, *8*, 147, doi:10.3390/plants8060147.
481. Zubair, M.; Farzand, A.; Mumtaz, F.; Khan, A.R.; Sheikh, T.M.M.; Haider, M.S.; Yu, C.; Wang, Y.; Ayaz, M.; Gu, Q.; et al. Novel Genetic Dysregulations and Oxidative Damage in Fusarium Graminearum Induced by Plant Defense Eliciting Psychrophilic Bacillus Atrophaeus TS1. *IJMS* **2021**, *22*, 12094, doi:10.3390/ijms222212094.
482. Zulfiqar, U.; Jiang, W.; Xiukang, W.; Hussain, S.; Ahmad, M.; Maqsood, M.F.; Ali, N.; Ishfaq, M.; Kaleem, M.; Haider, F.U.; et al. Cadmium Phytotoxicity, Tolerance, and Advanced Remediation Approaches in Agricultural Soils; A Comprehensive Review. *Front. Plant Sci.* **2022**, *13*, 773815, doi:10.3389/fpls.2022.773815.
483. Zúñiga, A.; Fuente, F. de la; Federici, F.; Lionne, C.; Bônnet, J.; de Lorenzo, V.; González, B. An Engineered Device for Indoleacetic Acid Production under Quorum Sensing Signals Enables *Cupriavidus Pinatubonensis* JMP134 To Stimulate Plant Growth. *ACS Synth. Biol.* **2018**, *7*, 1519–1527, doi:10.1021/acssynbio.8b00002.
484. Zuzolo, D.; Sciarrillo, R.; Postiglione, A.; Guarino, C. The Remediation Potential for PAHs of Verbascum Sinuatum L. Combined with an Enhanced Rhizosphere Landscape: A Full-Scale Mesocosm Experiment. *Biotechnology Reports* **2021**, *31*, e00657, doi:10.1016/j.btre.2021.e00657.

***Ascorbate peroxidase (APX)***

1. [No Title Found]. *RJLBPCS* *05*.
2. Aalipour, H.; Nikbakht, A.; Etemadi, N.; Rejali, F.; Soleimani, M. Biochemical Response and Interactions between Arbuscular Mycorrhizal Fungi and Plant Growth Promoting Rhizobacteria during Establishment and Stimulating Growth of Arizona Cypress (Cupressus Arizonica G.) under Drought Stress. *Scientia Horticulturae* **2020**, *261*, 108923, doi:10.1016/j.scienta.2019.108923.
3. Aamir, M.; Rai, K.K.; Dubey, M.K.; Zehra, A.; Tripathi, Y.N.; Divyanshu, K.; Samal, S.; Upadhyay, R.S. Impact of Climate Change on Soil Carbon Exchange, Ecosystem Dynamics, and Plant–Microbe Interactions. In *Climate Change and Agricultural Ecosystems*; Elsevier, 2019; pp. 379–413 ISBN 978-0-12-816483-9.
4. Abadi, V.A.J.M.; Sepehri, M. Effect of Piriformospora Indica and Azotobacter Chroococcum on Mitigation of Zinc Deficiency Stress in Wheat (Triticum Aestivum L.). *Symbiosis* **2016**, *69*, 9–19, doi:10.1007/s13199-015-0361-z.
5. Abbasi, S.; Alipour Kafi, S.; Karimi, E.; Sadeghi, A. Streptomyces Consortium Improved Quality Attributes of Bell Pepper Fruits, Induced Plant Defense Priming, and Changed Microbial Communities of Rhizosphere under Commercial Greenhouse Conditions. *Rhizosphere* **2022**, *23*, 100570, doi:10.1016/j.rhisph.2022.100570.
6. Abbasi, S.; Sadeghi, A.; Safaie, N. Streptomyces Alleviate Drought Stress in Tomato Plants and Modulate the Expression of Transcription Factors ERF1 and WRKY70 Genes. *Scientia Horticulturae* **2020**, *265*, 109206, doi:10.1016/j.scienta.2020.109206.
7. Abbasi, S.; Zahedi, H. Effect of Plant Growth Promoting Rhizobacteria (PGPR) on Antioxidative Enzymes of Soybean Subjected to Different Irrigation Regimes. 5.
8. Abd El-Daim, I.A.; Bejai, S.; Meijer, J. Bacillus Velezensis 5113 Induced Metabolic and Molecular Reprogramming during Abiotic Stress Tolerance in Wheat. *Sci Rep* **2019**, *9*, 16282, doi:10.1038/s41598-019-52567-x.
9. Abdel Latef, A.A.H.; Abu Alhmad, M.F.; Kordrostami, M.; Abo–Baker, A.-B.A.-E.; Zakir, A. Inoculation with Azospirillum Lipoferum or Azotobacter Chroococcum Reinforces Maize Growth by Improving Physiological Activities Under Saline Conditions. *J Plant Growth Regul* **2020**, *39*, 1293–1306, doi:10.1007/s00344-020-10065-9.
10. Abdel Latef, A.A.H.; Omer, A.M.; Badawy, A.A.; Osman, M.S.; Ragaey, M.M. Strategy of Salt Tolerance and Interactive Impact of Azotobacter Chroococcum and/or Alcaligenes Faecalis Inoculation on Canola (Brassica Napus L.) Plants Grown in Saline Soil. *Plants* **2021**, *10*, 110, doi:10.3390/plants10010110.
11. Abdelkhalek, A.; El-Gendi, H.; Al-Askar, A.A.; Maresca, V.; Moawad, H.; Elsharkawy, M.M.; Younes, H.A.; Behiry, S.I. Enhancing Systemic Resistance in Faba Bean (Vicia Faba L.) to Bean Yellow Mosaic Virus via Soil Application and Foliar Spray of Nitrogen-Fixing Rhizobium Leguminosarum Bv. Viciae Strain 33504-Alex1. *Front. Plant Sci.* **2022**, *13*, 933498, doi:10.3389/fpls.2022.933498.
12. Abdelkrim, S.; Abid, G.; Chaieb, O.; Taamalli, W.; Mannai, K.; Louati, F.; Jebara, M.; Jebara, S.H. *Plant Growth Promoting Rhizobacteria Modulates the Antioxidant Defense and the Expression of Stress Responsive Genes Providing Pb Accumulation and Tolerance of Grass Pea*; In Review, 2022;
13. Abdelkrim, S.; Jebara, S.H.; Jebara, M. Antioxidant Systems Responses and the Compatible Solutes as Contributing Factors to Lead Accumulation and Tolerance in Lathyrus Sativus Inoculated by Plant Growth Promoting Rhizobacteria. *Ecotoxicology and Environmental Safety* **2018**, *166*, 427–436, doi:10.1016/j.ecoenv.2018.09.115.
14. Abeed, A.H.A.; Eissa, M.A.; Abdel-Wahab, D.A. Effect of Exogenously Applied Jasmonic Acid and Kinetin on Drought Tolerance of Wheat Cultivars Based on Morpho-Physiological Evaluation. *J Soil Sci Plant Nutr* **2021**, *21*, 131–144, doi:10.1007/s42729-020-00348-1.
15. Abou-Sreea, A.I.B.; Azzam, C.R.; Al-Taweel, S.K.; Abdel-Aziz, R.M.; Belal, H.E.E.; Rady, M.M.; Abdel-Kader, A.A.S.; Majrashi, A.; Khaled, K.A.M. Natural Biostimulant Attenuates Salinity Stress Effects in Chili Pepper by Remodeling Antioxidant, Ion, and Phytohormone Balances, and Augments Gene Expression. *Plants* **2021**, *10*, 2316, doi:10.3390/plants10112316.
16. Abulfaraj, A.A.; Jalal, R.S. Use of Plant Growth-Promoting Bacteria to Enhance Salinity Stress in Soybean (Glycine Max L.) Plants. *Saudi Journal of Biological Sciences* **2021**, *28*, 3823–3834, doi:10.1016/j.sjbs.2021.03.053.
17. Acet, T.; Kadıoğlu, A. SOS5 Gene-Abscisic Acid Crosstalk and Their Interaction with Antioxidant System in Arabidopsis Thaliana under Salt Stress. *Physiol Mol Biol Plants* **2020**, *26*, 1831–1845, doi:10.1007/s12298-020-00873-4.
18. Acosta-Motos, J.R.; Penella, C.; Hernández, J.A.; Díaz-Vivancos, P.; Sánchez-Blanco, M.J.; Navarro, J.M.; Gómez-Bellot, M.J.; Barba-Espín, G. Towards a Sustainable Agriculture: Strategies Involving Phytoprotectants against Salt Stress. *Agronomy* **2020**, *10*, 194, doi:10.3390/agronomy10020194.
19. *Advances in Plant Microbiome and Sustainable Agriculture: Functional Annotation and Future Challenges*; Yadav, A.N., Rastegari, A.A., Yadav, N., Kour, D., Eds.; Microorganisms for Sustainability; Springer Singapore: Singapore, 2020; Vol. 20; ISBN 9789811532030.
20. Afridi, M.S.; Amna; Sumaira; Mahmood, T.; Salam, A.; Mukhtar, T.; Mehmood, S.; Ali, J.; Khatoon, Z.; Bibi, M.; et al. Induction of Tolerance to Salinity in Wheat Genotypes by Plant Growth Promoting Endophytes: Involvement of ACC Deaminase and Antioxidant Enzymes. *Plant Physiology and Biochemistry* **2019**, *139*, 569–577, doi:10.1016/j.plaphy.2019.03.041.
21. Afshari, M.; Rahimmalek, M.; Sabzalian, M.R.; Bielecka, M.; Matkowski, A.; Talebi, M. Changes in Physiological, Phytochemical Traits and Gene Expression of Two Perovskia Species in Response to Water Deficit. *Scientia Horticulturae* **2022**, *293*, 110747, doi:10.1016/j.scienta.2021.110747.
22. Agarwal, P.K.; Agarwal, P.; Reddy, M.K.; Sopory, S.K. Role of DREB Transcription Factors in Abiotic and Biotic Stress Tolerance in Plants. *Plant Cell Rep* **2006**, *25*, 1263–1274, doi:10.1007/s00299-006-0204-8.
23. *Agriculturally Important Fungi for Sustainable Agriculture: Volume 1: Perspective for Diversity and Crop Productivity*; Yadav, A.N., Mishra, S., Kour, D., Yadav, N., Kumar, A., Eds.; Fungal Biology; Springer International Publishing: Cham, 2020; ISBN 978-3-030-45970-3.
24. *Agronomic Crops: Volume 3: Stress Responses and Tolerance*; Hasanuzzaman, M., Ed.; Springer Singapore: Singapore, 2020; ISBN 9789811500244.
25. Ahluwalia, O.; Singh, P.C.; Bhatia, R. A Review on Drought Stress in Plants: Implications, Mitigation and the Role of Plant Growth Promoting Rhizobacteria. *Resources, Environment and Sustainability* **2021**, *5*, 100032, doi:10.1016/j.resenv.2021.100032.
26. Ahmad, B.; Qadir, S.U.; Dar, T.A.; Alam, P.; Yousuf, P.Y.; Ahmad, P. Karrikins: Smoke-Derived Phytohormones from Stress Alleviation to Signaling. *J Plant Growth Regul* **2022**, doi:10.1007/s00344-022-10703-4.
27. Ahmad, H.M.; Fiaz, S.; Hafeez, S.; Zahra, S.; Shah, A.N.; Gul, B.; Aziz, O.; Mahmood-Ur-Rahman; Fakhar, A.; Rafique, M.; et al. Plant Growth-Promoting Rhizobacteria Eliminate the Effect of Drought Stress in Plants: A Review. *Front. Plant Sci.* **2022**, *13*, 875774, doi:10.3389/fpls.2022.875774.
28. Ahmad, I.; Zaib, S.; Alves, P.C.M.S.; Luthe, D.S.; Bano, A.; Shakeel, S.N. Molecular and Physiological Analysis of Drought Stress Responses in Zea Mays Treated with Plant Growth Promoting Rhizobacteria. *Biologia plant.* **2019**, *63*, 536–547, doi:10.32615/bp.2019.092.
29. Akbari, A.; Gharanjik, S.; Koobaz, P.; Sadeghi, A. Plant Growth Promoting Streptomyces Strains Are Selectively Interacting with the Wheat Cultivars Especially in Saline Conditions. *Heliyon* **2020**, *6*, e03445, doi:10.1016/j.heliyon.2020.e03445.
30. Alatawi, A.; Wang, X.; Saleem, M.H.; Mohsin, M.; Rehman, M.; Usman, K.; Fahad, S.; Mfarrej, M.F.B.; Hefft, D.I.; Ali, S. Individual and Synergic Effects of Phosphorus and Gibberellic Acid on Organic Acids Exudation Pattern, Ultra-Structure of Chloroplast and Stress Response Gene Expression in Cu-Stressed Jute (Corchorus Capsularis L.). *J Plant Growth Regul* **2022**, doi:10.1007/s00344-022-10622-4.
31. Alberton, D.; Valdameri, G.; Moure, V.R.; Monteiro, R.A.; Pedrosa, F. de O.; Müller-Santos, M.; de Souza, E.M. What Did We Learn From Plant Growth-Promoting Rhizobacteria (PGPR)-Grass Associations Studies Through Proteomic and Metabolomic Approaches? *Front. Sustain. Food Syst.* **2020**, *4*, 607343, doi:10.3389/fsufs.2020.607343.
32. Alen’kina, S.A.; Kupryashina, M.A. Influence Of. *Soil Res.* **2021**, *60*, 197–209, doi:10.1071/SR21092.
33. Alen’kina, S.A.; Nikitina, V.E. Effect of Azospirillum Lectins on the Ascorbate Peroxidase Activity and Ascorbic Acid Content in Wheat Seedling Roots Exposed to Abiotic Stresses. *Appl Biochem Microbiol* **2020**, *56*, 211–218, doi:10.1134/S0003683820020027.
34. Alexander, A.; Singh, V.K.; Mishra, A. Halotolerant PGPR Stenotrophomonas Maltophilia BJ01 Induces Salt Tolerance by Modulating Physiology and Biochemical Activities of Arachis Hypogaea. *Front. Microbiol.* **2020**, *11*, 568289, doi:10.3389/fmicb.2020.568289.
35. Alexander, A.; Singh, V.K.; Mishra, A. Overexpression of Differentially Expressed AhCytb6 Gene during Plant-Microbe Interaction Improves Tolerance to N2 Deficit and Salt Stress in Transgenic Tobacco. *Sci Rep* **2021**, *11*, 13435, doi:10.1038/s41598-021-92424-4.
36. Alexander, A.; Singh, V.K.; Mishra, A.; Jha, B. Plant Growth Promoting Rhizobacterium Stenotrophomonas Maltophilia BJ01 Augments Endurance against N2 Starvation by Modulating Physiology and Biochemical Activities of Arachis Hypogea. *PLoS ONE* **2019**, *14*, e0222405, doi:10.1371/journal.pone.0222405.
37. Alharby, H.F.; Al-Zahrani, H.S.; Hakeem, K.R.; Alsamadany, H.; Desoky, E.-S.M.; Rady, M.M. Silymarin-Enriched Biostimulant Foliar Application Minimizes the Toxicity of Cadmium in Maize by Suppressing Oxidative Stress and Elevating Antioxidant Gene Expression. *Biomolecules* **2021**, *11*, 465, doi:10.3390/biom11030465.
38. Ali, B.; Wang, X.; Saleem, M.H.; Azeem, M.A.; Afridi, M.S.; Nadeem, M.; Ghazal, M.; Batool, T.; Qayyum, A.; Alatawi, A.; et al. Bacillus Mycoides PM35 Reinforces Photosynthetic Efficiency, Antioxidant Defense, Expression of Stress-Responsive Genes, and Ameliorates the Effects of Salinity Stress in Maize. *Life* **2022**, *12*, 219, doi:10.3390/life12020219.
39. Ali, B.; Wang, X.; Saleem, M.H.; Sumaira; Hafeez, A.; Afridi, M.S.; Khan, S.; Zaib-Un-Nisa; Ullah, I.; Amaral Júnior, A.T. do; et al. PGPR-Mediated Salt Tolerance in Maize by Modulating Plant Physiology, Antioxidant Defense, Compatible Solutes Accumulation and Bio-Surfactant Producing Genes. *Plants* **2022**, *11*, 345, doi:10.3390/plants11030345.
40. Ali, R.; Gul, H.; Hamayun, M.; Rauf, M.; Iqbal, A.; Hussain, A.; Lee, I.-J. Endophytic Fungi Controls the PhysicochemicalStatus of Maize Crop under Salt Stress. *Pol. J. Environ. Stud.* **2022**, *31*, 561–573, doi:10.15244/pjoes/134540.
41. Ali, Shaik.Z.; Sandhya, V.; Grover, M.; Linga, V.R.; Bandi, V. Effect of Inoculation with a Thermotolerant Plant Growth Promoting *Pseudomonas Putida* Strain AKMP7 on Growth of Wheat ( *Triticum* Spp.) under Heat Stress. *Journal of Plant Interactions* **2011**, *6*, 239–246, doi:10.1080/17429145.2010.545147.
42. Alkharabsheh, H.M.; Seleiman, M.F.; Hewedy, O.A.; Battaglia, M.L.; Jalal, R.S.; Alhammad, B.A.; Schillaci, C.; Ali, N.; Al-Doss, A. Field Crop Responses and Management Strategies to Mitigate Soil Salinity in Modern Agriculture: A Review. *Agronomy* **2021**, *11*, 2299, doi:10.3390/agronomy11112299.
43. Alves, L.R.; Rodrigues dos Reis, A.; Prado, E.R.; Lavres, J.; Pompeu, G.B.; Azevedo, R.A.; Gratão, P.L. New Insights into Cadmium Stressful-Conditions: Role of Ethylene on Selenium-Mediated Antioxidant Enzymes. *Ecotoxicology and Environmental Safety* **2019**, *186*, 109747, doi:10.1016/j.ecoenv.2019.109747.
44. Ankati, S.; Rani, T.S.; Podile, A.R. Changes in Root Exudates and Root Proteins in Groundnut–Pseudomonas Sp. Interaction Contribute to Root Colonization by Bacteria and Defense Response of the Host. *J Plant Growth Regul* **2019**, *38*, 523–538, doi:10.1007/s00344-018-9868-x.
45. Anshu, A.; Agarwal, P.; Mishra, K.; Yadav, U.; Verma, I.; Chauhan, S.; Srivastava, P.K.; Singh, P.C. Synergistic Action of Trichoderma Koningiopsis and T. Asperellum Mitigates Salt Stress in Paddy. *Physiol Mol Biol Plants* **2022**, *28*, 987–1004, doi:10.1007/s12298-022-01192-6.
46. *Antioxidants in Plant-Microbe Interaction*; Singh, H.B., Vaishnav, A., Sayyed, R.Z., Eds.; Springer Singapore: Singapore, 2021; ISBN 9789811613494.
47. *Antioxidants in Plant-Microbe Interaction*; Singh, H.B., Vaishnav, A., Sayyed, R.Z., Eds.; Springer Singapore: Singapore, 2021; ISBN 9789811613494.
48. *Antioxidants in Plant-Microbe Interaction*; Singh, H.B., Vaishnav, A., Sayyed, R.Z., Eds.; Springer Singapore: Singapore, 2021; ISBN 9789811613494.
49. *Antioxidants in Plant-Microbe Interaction*; Singh, H.B., Vaishnav, A., Sayyed, R.Z., Eds.; Springer Singapore: Singapore, 2021; ISBN 9789811613494.
50. *Antioxidants in Plant-Microbe Interaction*; Singh, H.B., Vaishnav, A., Sayyed, R.Z., Eds.; Springer Singapore: Singapore, 2021; ISBN 9789811613494.
51. Anwar, K.; Joshi, R.; Dhankher, O.P.; Singla-Pareek, S.L.; Pareek, A. Elucidating the Response of Crop Plants towards Individual, Combined and Sequentially Occurring Abiotic Stresses. *IJMS* **2021**, *22*, 6119, doi:10.3390/ijms22116119.
52. *Approaches to Plant Stress and Their Management*; Gaur, R.K., Sharma, P., Eds.; Springer India: New Delhi, 2014; ISBN 978-81-322-1619-3.
53. Armada, E.; Roldán, A.; Azcon, R. Differential Activity of Autochthonous Bacteria in Controlling Drought Stress in Native Lavandula and Salvia Plants Species Under Drought Conditions in Natural Arid Soil. *Microb Ecol* **2014**, *67*, 410–420, doi:10.1007/s00248-013-0326-9.
54. Arora, M.; Saxena, P.; Abdin, M.Z.; Varma, A. Interaction between Piriformospora Indica and Azotobacter Chroococcum Diminish the Effect of Salt Stress in Artemisia Annua L. by Enhancing Enzymatic and Non-Enzymatic Antioxidants. *Symbiosis* **2020**, *80*, 61–73, doi:10.1007/s13199-019-00656-w.
55. Awan, S.A.; Ilyas, N.; Khan, I.; Raza, M.A.; Rehman, A.U.; Rizwan, M.; Rastogi, A.; Tariq, R.; Brestic, M. Bacillus Siamensis Reduces Cadmium Accumulation and Improves Growth and Antioxidant Defense System in Two Wheat (Triticum Aestivum L.) Varieties. *Plants* **2020**, *9*, 878, doi:10.3390/plants9070878.
56. Aydinoglu, F.; Iltas, O.; Akkaya, O. Inoculation of Maize Seeds with Pseudomonas Putida Leads to Enhanced Seedling Growth in Combination with Modified Regulation of MiRNAs and Antioxidant Enzymes. *Symbiosis* **2020**, *81*, 271–285, doi:10.1007/s13199-020-00703-x.
57. Azcón, R.; del Carmen Perálvarez, M.; Roldán, A.; Barea, J.-M. Arbuscular Mycorrhizal Fungi, Bacillus Cereus, and Candida Parapsilosis from a Multicontaminated Soil Alleviate Metal Toxicity in Plants. *Microb Ecol* **2010**, *59*, 668–677, doi:10.1007/s00248-009-9618-5.
58. Azeem, M.; Haider, M.Z.; Javed, S.; Saleem, M.H.; Alatawi, A. Drought Stress Amelioration in Maize (Zea Mays L.) by Inoculation of Bacillus Spp. Strains under Sterile Soil Conditions. *Agriculture* **2022**, *12*, 50, doi:10.3390/agriculture12010050.
59. Azeem, M.A.; Shah, F.H.; Ullah, A.; Ali, K.; Jones, D.A.; Khan, M.E.H.; Ashraf, A. Biochemical Characterization of Halotolerant Bacillus Safensis PM22 and Its Potential to Enhance Growth of Maize under Salinity Stress. *Plants* **2022**, *11*, 1721, doi:10.3390/plants11131721.
60. Azimychetabi, Z.; Sabokdast Nodehi, M.; Karami Moghadam, T.; Motesharezadeh, B. Cadmium Stress Alters the Essential Oil Composition and the Expression of Genes Involved in Their Synthesis in Peppermint (Mentha Piperita L.). *Industrial Crops and Products* **2021**, *168*, 113602, doi:10.1016/j.indcrop.2021.113602.
61. Azizoglu, U.; Yilmaz, N.; Simsek, O.; Ibal, J.C.; Tagele, S.B.; Shin, J.-H. The Fate of Plant Growth-Promoting Rhizobacteria in Soilless Agriculture: Future Perspectives. *3 Biotech* **2021**, *11*, 382, doi:10.1007/s13205-021-02941-2.
62. Azmat, A.; Tanveer, Y.; Yasmin, H.; Hassan, M.N.; Shahzad, A.; Reddy, M.; Ahmad, A. Coactive Role of Zinc Oxide Nanoparticles and Plant Growth Promoting Rhizobacteria for Mitigation of Synchronized Effects of Heat and Drought Stress in Wheat Plants. *Chemosphere* **2022**, *297*, 133982, doi:10.1016/j.chemosphere.2022.133982.
63. Backer, R.; Rokem, J.S.; Ilangumaran, G.; Lamont, J.; Praslickova, D.; Ricci, E.; Subramanian, S.; Smith, D.L. Plant Growth-Promoting Rhizobacteria: Context, Mechanisms of Action, and Roadmap to Commercialization of Biostimulants for Sustainable Agriculture. *Front. Plant Sci.* **2018**, *9*, 1473, doi:10.3389/fpls.2018.01473.
64. *Bacterial Volatile Compounds as Mediators of Airborne Interactions*; Ryu, C.-M., Weisskopf, L., Piechulla, B., Eds.; Springer Singapore: Singapore, 2020; ISBN 9789811572920.
65. Badawy, I.H.; Hmed, A.A.; Sofy, M.R.; Al-Mokadem, A.Z. Alleviation of Cadmium and Nickel Toxicity and Phyto-Stimulation of Tomato Plant L. by Endophytic Micrococcus Luteus and Enterobacter Cloacae. *Plants* **2022**, *11*, 2018, doi:10.3390/plants11152018.
66. Bagheri, M.; Gholami, M.; Baninasab, B. Hydrogen Peroxide-Induced Salt Tolerance in Relation to Antioxidant Systems in Pistachio Seedlings. *Scientia Horticulturae* **2019**, *243*, 207–213, doi:10.1016/j.scienta.2018.08.026.
67. Baig, M.A.; Ahmad, J.; Bagheri, R.; Ali, A.A.; Al-Huqail, A.A.; Ibrahim, M.M.; Qureshi, M.I. Proteomic and Ecophysiological Responses of Soybean (Glycine Max L.) Root Nodules to Pb and Hg Stress. *BMC Plant Biol* **2018**, *18*, 283, doi:10.1186/s12870-018-1499-7.
68. Bakshi, P.; Chouhan, R.; Sharma, P.; Mir, B.A.; Gandhi, S.G.; Landi, M.; Zheng, B.; Sharma, A.; Bhardwaj, R. Amelioration of Chlorpyrifos-Induced Toxicity in Brassica Juncea L. by Combination of 24-Epibrassinolide and Plant-Growth-Promoting Rhizobacteria. *Biomolecules* **2021**, *11*, 877, doi:10.3390/biom11060877.
69. Bakshi, P.; Chouhan, R.; Sharma, P.; Mir, B.A.; Gandhi, S.G.; Landi, M.; Zheng, B.; Sharma, A.; Bhardwaj, R. Amelioration of Chlorpyrifos-Induced Toxicity in Brassica Juncea L. by Combination of 24-Epibrassinolide and Plant-Growth-Promoting Rhizobacteria. *Biomolecules* **2021**, *11*, 877, doi:10.3390/biom11060877.
70. Balkrishna, A.; Sharma, I.P.; Arya, V.; Sharma, A.K. Biologicals and Their Plant Stress Tolerance Ability. *Symbiosis* **2022**, *86*, 243–259, doi:10.1007/s13199-022-00842-3.
71. Bandeppa, S.; Paul, S.; Thakur, J.K.; Chandrashekar, N.; Umesh, D.K.; Aggarwal, C.; Asha, A.D. Antioxidant, Physiological and Biochemical Responses of Drought Susceptible and Drought Tolerant Mustard (Brassica Juncea L) Genotypes to Rhizobacterial Inoculation under Water Deficit Stress. *Plant Physiology and Biochemistry* **2019**, *143*, 19–28, doi:10.1016/j.plaphy.2019.08.018.
72. Bandyopadhyay, P.; Yadav, B.G.; Kumar, S.G.; Kumar, R.; Kogel, K.-H.; Kumar, S. Piriformospora Indica and Azotobacter Chroococcum Consortium Facilitates Higher Acquisition of N, P with Improved Carbon Allocation and Enhanced Plant Growth in Oryza Sativa. *JoF* **2022**, *8*, 453, doi:10.3390/jof8050453.
73. Barra, P.J.; Pontigo, S.; Delgado, M.; Parra–Almuna, L.; Duran, P.; Valentine, A.J.; Jorquera, M.A.; Mora, M. de la L. Phosphobacteria Inoculation Enhances the Benefit of P–Fertilization on Lolium Perenne in Soils Contrasting in P–Availability. *Soil Biology and Biochemistry* **2019**, *136*, 107516, doi:10.1016/j.soilbio.2019.06.012.
74. Basile, B.; Rouphael, Y.; Colla, G.; Soppelsa, S.; Andreotti, C. Appraisal of Emerging Crop Management Opportunities in Fruit Trees, Grapevines and Berry Crops Facilitated by the Application of Biostimulants. *Scientia Horticulturae* **2020**, *267*, 109330, doi:10.1016/j.scienta.2020.109330.
75. Basit, F.; Liu, J.; An, J.; Chen, M.; He, C.; Zhu, X.; Li, Z.; Hu, J.; Guan, Y. Brassinosteroids as a Multidimensional Regulator of Plant Physiological and Molecular Responses under Various Environmental Stresses. *Environ Sci Pollut Res* **2021**, *28*, 44768–44779, doi:10.1007/s11356-021-15087-8.
76. Batool, M.; El-Badri, A.M.; Hassan, M.U.; Haiyun, Y.; Chunyun, W.; Zhenkun, Y.; Jie, K.; Wang, B.; Zhou, G. Drought Stress in Brassica Napus: Effects, Tolerance Mechanisms, and Management Strategies. *J Plant Growth Regul* **2022**, doi:10.1007/s00344-021-10542-9.
77. Batool, T.; Ali, S.; Seleiman, M.F.; Naveed, N.H.; Ali, A.; Ahmed, K.; Abid, M.; Rizwan, M.; Shahid, M.R.; Alotaibi, M.; et al. Plant Growth Promoting Rhizobacteria Alleviates Drought Stress in Potato in Response to Suppressive Oxidative Stress and Antioxidant Enzymes Activities. *Sci Rep* **2020**, *10*, 16975, doi:10.1038/s41598-020-73489-z.
78. Begum, N.; Wang, L.; Ahmad, H.; Akhtar, K.; Roy, R.; Khan, M.I.; Zhao, T. Co-Inoculation of Arbuscular Mycorrhizal Fungi and the Plant Growth-Promoting Rhizobacteria Improve Growth and Photosynthesis in Tobacco Under Drought Stress by Up-Regulating Antioxidant and Mineral Nutrition Metabolism. *Microb Ecol* **2022**, *83*, 971–988, doi:10.1007/s00248-021-01815-7.
79. Ben-Laouane, R.; Baslam, M.; Ait-El-Mokhtar, M.; Anli, M.; Boutasknit, A.; Ait-Rahou, Y.; Toubali, S.; Mitsui, T.; Oufdou, K.; Wahbi, S.; et al. Potential of Native Arbuscular Mycorrhizal Fungi, Rhizobia, and/or Green Compost as Alfalfa (Medicago Sativa) Enhancers under Salinity. *Microorganisms* **2020**, *8*, 1695, doi:10.3390/microorganisms8111695.
80. Berni, R.; Hausman, J.-F.; Villas-Boas, S.; Guerriero, G. Impact of Pseudomonas Sp. SVB-B33 on Stress- and Cell Wall-Related Genes in Roots and Leaves of Hemp under Salinity. *Horticulturae* **2022**, *8*, 336, doi:10.3390/horticulturae8040336.
81. Bhanse, P.; Kumar, M.; Singh, L.; Awasthi, M.K.; Qureshi, A. Role of Plant Growth-Promoting Rhizobacteria in Boosting the Phytoremediation of Stressed Soils: Opportunities, Challenges, and Prospects. *Chemosphere* **2022**, *303*, 134954, doi:10.1016/j.chemosphere.2022.134954.
82. Bhardwaj, D.; Ansari, M.W.; Sahoo, R.K.; Tuteja, N. Biofertilizers Function as Key Player in Sustainable Agriculture by Improving Soil Fertility, Plant Tolerance and Crop Productivity. *Microb Cell Fact* **2014**, *13*, 66, doi:10.1186/1475-2859-13-66.
83. Bharti, N.; Barnawal, D.; Awasthi, A.; Yadav, A.; Kalra, A. Plant Growth Promoting Rhizobacteria Alleviate Salinity Induced Negative Effects on Growth, Oil Content and Physiological Status in Mentha Arvensis. *Acta Physiol Plant* **2014**, *36*, 45–60, doi:10.1007/s11738-013-1385-8.
84. Bharti, N.; Pandey, S.S.; Barnawal, D.; Patel, V.K.; Kalra, A. Plant Growth Promoting Rhizobacteria Dietzia Natronolimnaea Modulates the Expression of Stress Responsive Genes Providing Protection of Wheat from Salinity Stress. *Sci Rep* **2016**, *6*, 34768, doi:10.1038/srep34768.
85. Bharti, N.; Yadav, D.; Barnawal, D.; Maji, D.; Kalra, A. Exiguobacterium Oxidotolerans, a Halotolerant Plant Growth Promoting Rhizobacteria, Improves Yield and Content of Secondary Metabolites in Bacopa Monnieri (L.) Pennell under Primary and Secondary Salt Stress. *World J Microbiol Biotechnol* **2013**, *29*, 379–387, doi:10.1007/s11274-012-1192-1.
86. Bhat, M.A.; Kumar, V.; Bhat, M.A.; Wani, I.A.; Dar, F.L.; Farooq, I.; Bhatti, F.; Koser, R.; Rahman, S.; Jan, A.T. Mechanistic Insights of the Interaction of Plant Growth-Promoting Rhizobacteria (PGPR) With Plant Roots Toward Enhancing Plant Productivity by Alleviating Salinity Stress. *Front. Microbiol.* **2020**, *11*, 1952, doi:10.3389/fmicb.2020.01952.
87. Bhatt, P.; Verma, A.; Verma, S.; Anwar, Md.S.; Prasher, P.; Mudila, H.; Chen, S. Understanding Phytomicrobiome: A Potential Reservoir for Better Crop Management. *Sustainability* **2020**, *12*, 5446, doi:10.3390/su12135446.
88. Bhattacharyya, C.; Banerjee, S.; Acharya, U.; Mitra, A.; Mallick, I.; Haldar, A.; Haldar, S.; Ghosh, A.; Ghosh, A. Evaluation of Plant Growth Promotion Properties and Induction of Antioxidative Defense Mechanism by Tea Rhizobacteria of Darjeeling, India. *Sci Rep* **2020**, *10*, 15536, doi:10.1038/s41598-020-72439-z.
89. Bielach, A.; Hrtyan, M.; Tognetti, V. Plants under Stress: Involvement of Auxin and Cytokinin. *IJMS* **2017**, *18*, 1427, doi:10.3390/ijms18071427.
90. *Biofertilizers for Sustainable Agriculture and Environment*; Giri, B., Prasad, R., Wu, Q.-S., Varma, A., Eds.; Soil Biology; Springer International Publishing: Cham, 2019; Vol. 55; ISBN 978-3-030-18932-7.
91. *Biology and Biotechnology of Quinoa: Super Grain for Food Security*; Varma, A., Ed.; Springer Singapore: Singapore, 2021; ISBN 9789811638312.
92. Bisht, N.; Chauhan, P.S. Comparing the Growth-Promoting Potential of Paenibacillus Lentimorbus and Bacillus Amyloliquefaciens in Oryza Sativa L. Var. Sarju-52 under Suboptimal Nutrient Conditions. *Plant Physiology and Biochemistry* **2020**, *146*, 187–197, doi:10.1016/j.plaphy.2019.11.023.
93. Bisht, N.; Mishra, S.K.; Chauhan, P.S. Bacillus Amyloliquefaciens Inoculation Alters Physiology of Rice (Oryza Sativa L. Var. IR-36) through Modulating Carbohydrate Metabolism to Mitigate Stress Induced by Nutrient Starvation. *International Journal of Biological Macromolecules* **2020**, *143*, 937–951, doi:10.1016/j.ijbiomac.2019.09.154.
94. Bisht, N.; Tiwari, S.; Singh, P.C.; Niranjan, A.; Singh Chauhan, P. A Multifaceted Rhizobacterium Paenibacillus Lentimorbus Alleviates Nutrient Deficiency-Induced Stress in Cicer Arietinum L. *Microbiological Research* **2019**, *223–225*, 110–119, doi:10.1016/j.micres.2019.04.007.
95. Biswas, S.; Nath, A.; Pal, R. A Comparison of Bacterial Variability across Biogeographic Regions Based on PGPR. *IJEAB* **2021**, *6*, 064–072, doi:10.22161/ijeab.66.8.
96. Boamah, S.; Zhang, S.; Xu, B.; Tong, L.; Inayat, R.; Calderón-Urrea, A. The Role of Trichoderma Species in Plants Response to Salt Stress. *AJRCS* **2021**, 28–43, doi:10.9734/ajrcs/2021/v6i230114.
97. Bogati, K.; Walczak, M. The Impact of Drought Stress on Soil Microbial Community, Enzyme Activities and Plants. *Agronomy* **2022**, *12*, 189, doi:10.3390/agronomy12010189.
98. Bomle, D.V.; Kiran, A.; Kumar, J.K.; Nagaraj, L.S.; Pradeep, C.K.; Ansari, M.A.; Alghamdi, S.; Kabrah, A.; Assaggaf, H.; Dablool, A.S.; et al. Plants Saline Environment in Perception with Rhizosphere Bacteria Containing 1-Aminocyclopropane-1-Carboxylate Deaminase. *IJMS* **2021**, *22*, 11461, doi:10.3390/ijms222111461.
99. Braga-Reis, I.; Neris, D.M.; Ribas, A.F.; Vieira, L.G.E.; Souza, G.M. Gamma-Aminobutyric Acid (GABA) and Acetylcholine (ACh) Alleviate Water Deficit Effects in Soybean: From Gene Expression up to Growth Performance. *Environmental and Experimental Botany* **2021**, *182*, 104303, doi:10.1016/j.envexpbot.2020.104303.
100. Bruno, L.B.; Karthik, C.; Ma, Y.; Kadirvelu, K.; Freitas, H.; Rajkumar, M. Amelioration of Chromium and Heat Stresses in Sorghum Bicolor by Cr6+ Reducing-Thermotolerant Plant Growth Promoting Bacteria. *Chemosphere* **2020**, *244*, 125521, doi:10.1016/j.chemosphere.2019.125521.
101. Bukhat, S.; Imran, A.; Javaid, S.; Shahid, M.; Majeed, A.; Naqqash, T. Communication of Plants with Microbial World: Exploring the Regulatory Networks for PGPR Mediated Defense Signaling. *Microbiological Research* **2020**, *238*, 126486, doi:10.1016/j.micres.2020.126486.
102. Cao, Y.; Yang, M.; Ma, W.; Sun, Y.; Chen, G. Overexpression of SSBXoc, a Single-Stranded DNA-Binding Protein From Xanthomonas Oryzae Pv. Oryzicola, Enhances Plant Growth and Disease and Salt Stress Tolerance in Transgenic Nicotiana Benthamiana. *Front. Plant Sci.* **2018**, *9*, 953, doi:10.3389/fpls.2018.00953.
103. Cassells, A.C.; Doyle, B.M. GENETIC ENGINEERING AND MUTATION BREEDING FOR TOLERANCE TO ABIOTIC AND BIOTIC STRESSES: SCIENCE, TECHNOLOGY AND SAFETY. 31.
104. Castiglione, S.; Oliva, G.; Vigliotta, G.; Novello, G.; Gamalero, E.; Lingua, G.; Cicatelli, A.; Guarino, F. Effects of Compost Amendment on Glycophyte and Halophyte Crops Grown on Saline Soils: Isolation and Characterization of Rhizobacteria with Plant Growth Promoting Features and High Salt Resistance. *Applied Sciences* **2021**, *11*, 2125, doi:10.3390/app11052125.
105. Cataldo, E.; Fucile, M.; Mattii, G.B. Biostimulants in Viticulture: A Sustainable Approach against Biotic and Abiotic Stresses. *Plants* **2022**, *11*, 162, doi:10.3390/plants11020162.
106. Chakraborti, S.; Bera, K.; Sadhukhan, S.; Dutta, P. Bio-Priming of Seeds: Plant Stress Management and Its Underlying Cellular, Biochemical and Molecular Mechanisms. *Plant Stress* **2022**, *3*, 100052, doi:10.1016/j.stress.2021.100052.
107. Chakraborty, N.; Chandra, S.; Acharya, K. Biochemical Basis of Improvement of Defense in Tomato Plant against Fusarium Wilt by CaCl2. *Physiol Mol Biol Plants* **2017**, *23*, 581–596, doi:10.1007/s12298-017-0450-y.
108. Chakraborty, U.; Chakraborty, B.N.; Chakraborty, A.P.; Dey, P.L. Water Stress Amelioration and Plant Growth Promotion in Wheat Plants by Osmotic Stress Tolerant Bacteria. *World J Microbiol Biotechnol* **2013**, *29*, 789–803, doi:10.1007/s11274-012-1234-8.
109. Chakraborty, U.; Chakraborty, B.N.; Dey, P.L.; Chakraborty, A.P.; Sarkar, J. Biochemical Responses of Wheat Plants Primed with Ochrobactrum Pseudogrignonense and Subjected to Salinity Stress. *Agric Res* **2019**, *8*, 427–440, doi:10.1007/s40003-018-0394-7.
110. Chakraborty, U.; Roy, S.; Chakraborty, A.P.; Dey, P.; Chakraborty, B. Plant Growth Promotion and Amelioration of Salinity Stress in Crop Plants by a Salt-Tolerant Bacterium. **2011**, 10.
111. Chandra, D.; Srivastava, R.; Gupta, V.V.S.R.; Franco, C.M.M.; Paasricha, N.; Saifi, S.K.; Tuteja, N.; Sharma, A.K. Field Performance of Bacterial Inoculants to Alleviate Water Stress Effects in Wheat (Triticum Aestivum L.). *Plant Soil* **2019**, *441*, 261–281, doi:10.1007/s11104-019-04115-9.
112. Chandran, H.; Meena, M.; Swapnil, P. Plant Growth-Promoting Rhizobacteria as a Green Alternative for Sustainable Agriculture. *Sustainability* **2021**, *13*, 10986, doi:10.3390/su131910986.
113. Chandrashekar, S.; Umesha, S. 2,6-Dichloroisonicotinic Acid Enhances the Expression of Defense Genes in Tomato Seedlings against Xanthomonas Perforans. *Physiological and Molecular Plant Pathology* **2014**, *86*, 49–56, doi:10.1016/j.pmpp.2014.03.003.
114. Chandwani, S.; Amaresan, N. Role of ACC Deaminase Producing Bacteria for Abiotic Stress Management and Sustainable Agriculture Production. *Environ Sci Pollut Res* **2022**, *29*, 22843–22859, doi:10.1007/s11356-022-18745-7.
115. Chatterjee, P.; Samaddar, S.; Anandham, R.; Kang, Y.; Kim, K.; Selvakumar, G.; Sa, T. Beneficial Soil Bacterium Pseudomonas Frederiksbergensis OS261 Augments Salt Tolerance and Promotes Red Pepper Plant Growth. *Front. Plant Sci.* **2017**, *8*, 705, doi:10.3389/fpls.2017.00705.
116. Chen, J.; Sharifi, R.; Khan, M.S.S.; Islam, F.; Bhat, J.A.; Kui, L.; Majeed, A. Wheat Microbiome: Structure, Dynamics, and Role in Improving Performance Under Stress Environments. *Front. Microbiol.* **2022**, *12*, 821546, doi:10.3389/fmicb.2021.821546.
117. Cheng, F.; Cheng, Z. Research Progress on the Use of Plant Allelopathy in Agriculture and the Physiological and Ecological Mechanisms of Allelopathy. *Front. Plant Sci.* **2015**, *6*, doi:10.3389/fpls.2015.01020.
118. Chiappero, J.; Cappellari, L. del R.; Palermo, T.B.; Giordano, W.; Khan, N.; Banchio, E. Antioxidant Status of Medicinal and Aromatic Plants under the Influence of Growth-Promoting Rhizobacteria and Osmotic Stress. *Industrial Crops and Products* **2021**, *167*, 113541, doi:10.1016/j.indcrop.2021.113541.
119. Chiboub, M.; Jebara, S.H.; Abid, G.; Jebara, M. Co-Inoculation Effects of Rhizobium Sullae and Pseudomonas Sp. on Growth, Antioxidant Status, and Expression Pattern of Genes Associated with Heavy Metal Tolerance and Accumulation of Cadmium in Sulla Coronaria. *J Plant Growth Regul* **2020**, *39*, 216–228, doi:10.1007/s00344-019-09976-z.
120. Cho, S.-M.; Kang, B.R.; Kim, Y.C. Transcriptome Analysis of Induced Systemic Drought Tolerance Elicited by Pseudomonas Chlororaphis O6 in Arabidopsis Thaliana. *The Plant Pathology Journal* **2013**, *29*, 209–220, doi:10.5423/PPJ.SI.07.2012.0103.
121. Choudhary, A.K.; Sultana, R.; Vales, M.I.; Saxena, K.B.; Kumar, R.R.; Ratnakumar, P. Integrated Physiological and Molecular Approaches to Improvement of Abiotic Stress Tolerance in Two Pulse Crops of the Semi-Arid Tropics. *The Crop Journal* **2018**, *6*, 99–114, doi:10.1016/j.cj.2017.11.002.
122. Choudhary, D.K.; Kasotia, A.; Jain, S.; Vaishnav, A.; Kumari, S.; Sharma, K.P.; Varma, A. Bacterial-Mediated Tolerance and Resistance to Plants Under Abiotic and Biotic Stresses. *J Plant Growth Regul* **2016**, *35*, 276–300, doi:10.1007/s00344-015-9521-x.
123. Chu, T.N.; Tran, B.T.H.; Van Bui, L.; Hoang, M.T.T. Plant Growth-Promoting Rhizobacterium Pseudomonas PS01 Induces Salt Tolerance in Arabidopsis Thaliana. *BMC Res Notes* **2019**, *12*, 11, doi:10.1186/s13104-019-4046-1.
124. Chung, I.-M.; Venkidasamy, B.; Upadhyaya, C.P.; Packiaraj, G.; Rajakumar, G.; Thiruvengadam, M. Alleviation of Phytophthora Infestans Mediated Necrotic Stress in the Transgenic Potato (Solanum Tuberosum L.) with Enhanced Ascorbic Acid Accumulation. *Plants* **2019**, *8*, 365, doi:10.3390/plants8100365.
125. Coba de la Peña, T.; Pueyo, J.J. Legumes in the Reclamation of Marginal Soils, from Cultivar and Inoculant Selection to Transgenic Approaches. *Agron. Sustain. Dev.* **2012**, *32*, 65–91, doi:10.1007/s13593-011-0024-2.
126. *Current Trends in Microbial Biotechnology for Sustainable Agriculture*; Yadav, A.N., Singh, J., Singh, C., Yadav, N., Eds.; Environmental and Microbial Biotechnology; Springer Singapore: Singapore, 2021; ISBN 9789811569487.
127. da Silva, M.D.; de Oliveira Silva, R.L.; Ferreira Neto, J.R.C.; Benko-Iseppon, A.M.; Kido, E.A. Genotype-Dependent Regulation of Drought-Responsive Genes in Tolerant and Sensitive Sugarcane Cultivars. *Gene* **2017**, *633*, 17–27, doi:10.1016/j.gene.2017.08.022.
128. Da, K.; Nowak, J.; Flinn, B. Potato Cytosine Methylation and Gene Expression Changes Induced by a Beneficial Bacterial Endophyte, Burkholderia Phytofirmans Strain PsJN. *Plant Physiology and Biochemistry* **2012**, *50*, 24–34, doi:10.1016/j.plaphy.2011.09.013.
129. Damalas, C.A.; Koutroubas, S.D. Exogenous Application of Salicylic Acid for Regulation of Sunflower Growth under Abiotic Stress: A Systematic Review. *Biologia* **2022**, *77*, 1685–1697, doi:10.1007/s11756-022-01020-y.
130. Dar, T.A.; Uddin, M.; Khan, M.M.A.; Hakeem, K.R.; Jaleel, H. Jasmonates Counter Plant Stress: A Review. *Environmental and Experimental Botany* **2015**, *115*, 49–57, doi:10.1016/j.envexpbot.2015.02.010.
131. de la Osa, C.; Rodríguez-Carvajal, M.Á.; Gandullo, J.; Aranda, C.; Megías, M.; Ollero, F.J.; López-Baena, F.J.; Monreal, J.A. Plant Growth-Promoting Rhizobacteria Modulate the Concentration of Bioactive Compounds in Tomato Fruits. *Separations* **2021**, *8*, 223, doi:10.3390/separations8110223.
132. Delgado, C.; Mora-Poblete, F.; Ahmar, S.; Chen, J.-T.; Figueroa, C.R. Jasmonates and Plant Salt Stress: Molecular Players, Physiological Effects, and Improving Tolerance by Using Genome-Associated Tools. *IJMS* **2021**, *22*, 3082, doi:10.3390/ijms22063082.
133. Department of Agricultural Sciences, University of Naples Federico II, Portici, Italy; De Pascale, S.; Rouphael, Y.; Department of Agricultural Sciences, University of Naples Federico II, Portici, Italy; Colla, G.; Department of Agricultural and Forestry Sciences, University of Tuscia, Viterbo, Italy Plant Biostimulants: Innovative Tool for Enhancing Plant Nutrition in Organic Farming. *Europ.J.Hortic.Sci.* **2018**, *82*, 277–285, doi:10.17660/eJHS.2017/82.6.2.
134. Dept. of Microbiology, College of Basic Sciences & Humanities, CCS HAU, Hisar, Haryana (125 004), India; Mondal, H.K.; Kaur, H.; Dept. of Microbiology, College of Basic Sciences & Humanities, CCS HAU, Hisar, Haryana (125 004), India Effect of Salt Stress on Medicinal Plants and Its Amelioration by Plant Growth Promoting Microbes. *IJBSM* **2017**, *8*, 477–487, doi:10.23910/IJBSM/2017.8.3.1772.
135. Desoky, E.-S.M.; Saad, A.M.; El-Saadony, M.T.; Merwad, A.-R.M.; Rady, M.M. Plant Growth-Promoting Rhizobacteria: Potential Improvement in Antioxidant Defense System and Suppression of Oxidative Stress for Alleviating Salinity Stress in Triticum Aestivum (L.) Plants. *Biocatalysis and Agricultural Biotechnology* **2020**, *30*, 101878, doi:10.1016/j.bcab.2020.101878.
136. Dev, K.; Choudhary, M.; Sourirajan, A. SALT TOLERANT BACTERIA FOR CROP IMPROVEMENT IN SALINE AGRICULTURE FIELDS: DEVELOPMENT, CHALLENGES AND OPPORTUNITIES. 17.
137. Devarajan, A.K.; Muthukrishanan, G.; Truu, J.; Truu, M.; Ostonen, I.; Kizhaeral S., S.; Panneerselvam, P.; Kuttalingam Gopalasubramanian, S. The Foliar Application of Rice Phyllosphere Bacteria Induces Drought-Stress Tolerance in Oryza Sativa (L.). *Plants* **2021**, *10*, 387, doi:10.3390/plants10020387.
138. Devarajan, A.K.; Muthukrishanan, G.; Truu, J.; Truu, M.; Ostonen, I.; Kizhaeral S., S.; Panneerselvam, P.; Kuttalingam Gopalasubramanian, S. The Foliar Application of Rice Phyllosphere Bacteria Induces Drought-Stress Tolerance in Oryza Sativa (L.). *Plants* **2021**, *10*, 387, doi:10.3390/plants10020387.
139. Devi, N.S.A.; Kumutha, K.; Anandham, R.; Krishnamoorthy, R. Induction of Moisture Stress Tolerance by Bacillus and Paenibacillus in Pigeon Pea (Cajanus Cajan. L). *3 Biotech* **2021**, *11*, 355, doi:10.1007/s13205-021-02901-w.
140. Dey, G.; Banerjee, P.; Sharma, R.K.; Maity, J.P.; Etesami, H.; Shaw, A.K.; Huang, Y.-H.; Huang, H.-B.; Chen, C.-Y. Management of Phosphorus in Salinity-Stressed Agriculture for Sustainable Crop Production by Salt-Tolerant Phosphate-Solubilizing Bacteria—A Review. *Agronomy* **2021**, *11*, 1552, doi:10.3390/agronomy11081552.
141. Dhanya Thomas, T.T.; Dinakar, C.; Puthur, J.T. Effect of UV-B Priming on the Abiotic Stress Tolerance of Stress-Sensitive Rice Seedlings: Priming Imprints and Cross-Tolerance. *Plant Physiology and Biochemistry* **2020**, *147*, 21–30, doi:10.1016/j.plaphy.2019.12.002.
142. Diagne, N.; Ngom, M.; Djighaly, P.I.; Fall, D.; Hocher, V.; Svistoonoff, S. Roles of Arbuscular Mycorrhizal Fungi on Plant Growth and Performance: Importance in Biotic and Abiotic Stressed Regulation. *Diversity* **2020**, *12*, 370, doi:10.3390/d12100370.
143. Dimopoulou, A.; Theologidis, I.; Liebmann, B.; Kalantidis, K.; Vassilakos, N.; Skandalis, N. Bacillus Amyloliquefaciens MBI600 Differentially Induces Tomato Defense Signaling Pathways Depending on Plant Part and Dose of Application. *Sci Rep* **2019**, *9*, 19120, doi:10.1038/s41598-019-55645-2.
144. Dixit, R.; Agrawal, L.; Gupta, S.; Kumar, M.; Yadav, S.; Chauhan, P.S.; Nautiyal, C.S. Southern Blight Disease of Tomato Control by 1-Aminocyclopropane-1-Carboxylate (ACC) Deaminase Producing *Paenibacillus Lentimorbus* B-30488. *Plant Signaling & Behavior* **2016**, *11*, e1113363, doi:10.1080/15592324.2015.1113363.
145. Dixit, R.; Agrawal, L.; Singh, S.P.; Prateeksha; Singh, P.C.; Prasad, V.; Chauhan, P.S. Paenibacillus Lentimorbus Induces Autophagy for Protecting Tomato from Sclerotium Rolfsii Infection. *Microbiological Research* **2018**, *215*, 164–174, doi:10.1016/j.micres.2018.07.008.
146. Dixit, R.; Agrawal, L.; Srivastava, S.; Chauhan, P.S. Paenibacillus Lentimorbus Enhanced Abiotic Stress Tolerance Through Lateral Root Formation and Phytohormone Regulation. *J Plant Growth Regul* **2022**, *41*, 2198–2209, doi:10.1007/s00344-021-10439-7.
147. Dong, W.; Liu, X.; Li, D.; Gao, T.; Song, Y. Transcriptional Profiling Reveals That a MYB Transcription Factor MsMYB4 Contributes to the Salinity Stress Response of Alfalfa. *PLoS ONE* **2018**, *13*, e0204033, doi:10.1371/journal.pone.0204033.
148. Du, N.; Yang, Q.; Xue, L.; Guo, H.; Lv, J.; Zhang, T.; Dong, X.; Shen, S.; Piao, F. Paenibacillus Polymyxa NSY50 Improves Defense Against Fusarium Oxysporum by Increasing Photosynthetic Efficiency, Sucrose Metabolism, and Antioxidant Capacity in Cucumber. *J Plant Growth Regul* **2022**, doi:10.1007/s00344-022-10699-x.
149. Dubey, A.; Kumar, A.; Abd_Allah, E.F.; Hashem, A.; Khan, M.L. Growing More with Less: Breeding and Developing Drought Resilient Soybean to Improve Food Security. *Ecological Indicators* **2019**, *105*, 425–437, doi:10.1016/j.ecolind.2018.03.003.
150. Dukare, A.; Mhatre, P.; Maheshwari, H.S.; Bagul, S.; Manjunatha, B.S.; Khade, Y.; Kamble, U. Delineation of Mechanistic Approaches of Rhizosphere Microorganisms Facilitated Plant Health and Resilience under Challenging Conditions. *3 Biotech* **2022**, *12*, 57, doi:10.1007/s13205-022-03115-4.
151. Durgadevi, D.; Harish, S.; Manikandan, R.; Prabhukarthikeyan, S.R.; Alice, D.; Raguchander, T. Proteomic Profiling of Defense/Resistant Genes Induced during the Tripartite Interaction of Oryza Sativa, Rhizoctonia Solani AG1-1A, and Bacillus Subtilis against Rice Sheath Blight. *Physiological and Molecular Plant Pathology* **2021**, *115*, 101669, doi:10.1016/j.pmpp.2021.101669.
152. E, Y.; Yuan, J.; Yang, F.; Wang, L.; Ma, J.; Li, J.; Pu, X.; Raza, W.; Huang, Q.; Shen, Q. PGPR Strain Paenibacillus Polymyxa SQR-21 Potentially Benefits Watermelon Growth by Re-Shaping Root Protein Expression. *AMB Expr* **2017**, *7*, 104, doi:10.1186/s13568-017-0403-4.
153. Egamberdieva, D.; Davranov, K.; Wirth, S.; Hashem, A.; Abd_Allah, E.F. Impact of Soil Salinity on the Plant-Growth – Promoting and Biological Control Abilities of Root Associated Bacteria. *Saudi Journal of Biological Sciences* **2017**, *24*, 1601–1608, doi:10.1016/j.sjbs.2017.07.004.
154. Egamberdieva, D.; Wirth, S.; Bellingrath-Kimura, S.D.; Mishra, J.; Arora, N.K. Salt-Tolerant Plant Growth Promoting Rhizobacteria for Enhancing Crop Productivity of Saline Soils. *Front. Microbiol.* **2019**, *10*, 2791, doi:10.3389/fmicb.2019.02791.
155. Egamberdieva, D.; Wirth, S.J.; Alqarawi, A.A.; Abd_Allah, E.F.; Hashem, A. Phytohormones and Beneficial Microbes: Essential Components for Plants to Balance Stress and Fitness. *Front. Microbiol.* **2017**, *8*, 2104, doi:10.3389/fmicb.2017.02104.
156. El-Dakak, R.; El-Aggan, W.; Badr, G.; Helaly, A.; Tammam, A. Positive Salt Tolerance Modulation via Vermicompost Regulation of SOS1 Gene Expression and Antioxidant Homeostasis in Viciafaba Plant. *Plants* **2021**, *10*, 2477, doi:10.3390/plants10112477.
157. El-Esawi, M.; Alaraidh, I.; Alsahli, A.; Alzahrani, S.; Ali, H.; Alayafi, A.; Ahmad, M. Serratia Liquefaciens KM4 Improves Salt Stress Tolerance in Maize by Regulating Redox Potential, Ion Homeostasis, Leaf Gas Exchange and Stress-Related Gene Expression. *IJMS* **2018**, *19*, 3310, doi:10.3390/ijms19113310.
158. El-Esawi, M.; Alayafi, A. Overexpression of StDREB2 Transcription Factor Enhances Drought Stress Tolerance in Cotton (Gossypium Barbadense L.). *Genes* **2019**, *10*, 142, doi:10.3390/genes10020142.
159. El-Esawi, M.A.; Alaraidh, I.A.; Alsahli, A.A.; Alamri, S.A.; Ali, H.M.; Alayafi, A.A. Bacillus Firmus (SW5) Augments Salt Tolerance in Soybean (Glycine Max L.) by Modulating Root System Architecture, Antioxidant Defense Systems and Stress-Responsive Genes Expression. *Plant Physiology and Biochemistry* **2018**, *132*, 375–384, doi:10.1016/j.plaphy.2018.09.026.
160. El-Esawi, M.A.; Al-Ghamdi, A.A.; Ali, H.M.; Alayafi, A.A. Azospirillum Lipoferum FK1 Confers Improved Salt Tolerance in Chickpea (Cicer Arietinum L.) by Modulating Osmolytes, Antioxidant Machinery and Stress-Related Genes Expression. *Environmental and Experimental Botany* **2019**, *159*, 55–65, doi:10.1016/j.envexpbot.2018.12.001.
161. El-Esawi, M.A.; Elansary, H.O.; El-Shanhorey, N.A.; Abdel-Hamid, A.M.E.; Ali, H.M.; Elshikh, M.S. Salicylic Acid-Regulated Antioxidant Mechanisms and Gene Expression Enhance Rosemary Performance under Saline Conditions. *Front. Physiol.* **2017**, *8*, 716, doi:10.3389/fphys.2017.00716.
162. El-Esawi, M.A.; Elkelish, A.; Soliman, M.; Elansary, H.O.; Zaid, A.; Wani, S.H. Serratia Marcescens BM1 Enhances Cadmium Stress Tolerance and Phytoremediation Potential of Soybean Through Modulation of Osmolytes, Leaf Gas Exchange, Antioxidant Machinery, and Stress-Responsive Genes Expression. *Antioxidants* **2020**, *9*, 43, doi:10.3390/antiox9010043.
163. El-Saadony, M.T.; ALmoshadak, A.S.; Shafi, M.E.; Albaqami, N.M.; Saad, A.M.; El-Tahan, A.M.; Desoky, E.-S.M.; Elnahal, A.S.M.; Almakas, A.; Abd El-Mageed, T.A.; et al. Vital Roles of Sustainable Nano-Fertilizers in Improving Plant Quality and Quantity-an Updated Review. *Saudi Journal of Biological Sciences* **2021**, *28*, 7349–7359, doi:10.1016/j.sjbs.2021.08.032.
164. Erice, G.; Ruíz-Lozano, J.M.; Zamarreño, Á.M.; García-Mina, J.M.; Aroca, R. Transcriptomic Analysis Reveals the Importance of JA-Ile Turnover in the Response of Arabidopsis Plants to Plant Growth Promoting Rhizobacteria and Salinity. *Environmental and Experimental Botany* **2017**, *143*, 10–19, doi:10.1016/j.envexpbot.2017.08.006.
165. Etesami, H. Bacterial Mediated Alleviation of Heavy Metal Stress and Decreased Accumulation of Metals in Plant Tissues: Mechanisms and Future Prospects. *Ecotoxicology and Environmental Safety* **2018**, *147*, 175–191, doi:10.1016/j.ecoenv.2017.08.032.
166. Etesami, H. Can Interaction between Silicon and Plant Growth Promoting Rhizobacteria Benefit in Alleviating Abiotic and Biotic Stresses in Crop Plants? *Agriculture, Ecosystems & Environment* **2018**, *253*, 98–112, doi:10.1016/j.agee.2017.11.007.
167. Etesami, H.; Beattie, G.A. Mining Halophytes for Plant Growth-Promoting Halotolerant Bacteria to Enhance the Salinity Tolerance of Non-Halophytic Crops. *Front. Microbiol.* **2018**, *9*, 148, doi:10.3389/fmicb.2018.00148.
168. Etesami, H.; Glick, B.R. Halotolerant Plant Growth–Promoting Bacteria: Prospects for Alleviating Salinity Stress in Plants. *Environmental and Experimental Botany* **2020**, *178*, 104124, doi:10.1016/j.envexpbot.2020.104124.
169. Etesami, H.; Maheshwari, D.K. Use of Plant Growth Promoting Rhizobacteria (PGPRs) with Multiple Plant Growth Promoting Traits in Stress Agriculture: Action Mechanisms and Future Prospects. *Ecotoxicology and Environmental Safety* **2018**, *156*, 225–246, doi:10.1016/j.ecoenv.2018.03.013.
170. Fahad, S.; Hussain, S.; Bano, A.; Saud, S.; Hassan, S.; Shan, D.; Khan, F.A.; Khan, F.; Chen, Y.; Wu, C.; et al. Potential Role of Phytohormones and Plant Growth-Promoting Rhizobacteria in Abiotic Stresses: Consequences for Changing Environment. *Environ Sci Pollut Res* **2015**, *22*, 4907–4921, doi:10.1007/s11356-014-3754-2.
171. Fahad, S.; Hussain, S.; Matloob, A.; Khan, F.A.; Khaliq, A.; Saud, S.; Hassan, S.; Shan, D.; Khan, F.; Ullah, N.; et al. Phytohormones and Plant Responses to Salinity Stress: A Review. *Plant Growth Regul* **2015**, *75*, 391–404, doi:10.1007/s10725-014-0013-y.
172. Fan, D.; Subramanian, S.; Smith, D.L. Plant Endophytes Promote Growth and Alleviate Salt Stress in Arabidopsis Thaliana. *Sci Rep* **2020**, *10*, 12740, doi:10.1038/s41598-020-69713-5.
173. Farooq, M.; Gogoi, N.; Hussain, M.; Barthakur, S.; Paul, S.; Bharadwaj, N.; Migdadi, H.M.; Alghamdi, S.S.; Siddique, K.H.M. Effects, Tolerance Mechanisms and Management of Salt Stress in Grain Legumes. *Plant Physiology and Biochemistry* **2017**, *118*, 199–217, doi:10.1016/j.plaphy.2017.06.020.
174. Farooqi, Z.U.R.; Ayub, M.A.; Zia ur Rehman, M.; Sohail, M.I.; Usman, M.; Khalid, H.; Naz, K. Regulation of Drought Stress in Plants. In *Plant Life Under Changing Environment*; Elsevier, 2020; pp. 77–104 ISBN 978-0-12-818204-8.
175. Fatima, A.; Hussain, S.; Hussain, S.; Ali, B.; Ashraf, U.; Zulfiqar, U.; Aslam, Z.; Al-Robai, S.A.; Alzahrani, F.O.; Hano, C.; et al. Differential Morphophysiological, Biochemical, and Molecular Responses of Maize Hybrids to Salinity and Alkalinity Stresses. *Agronomy* **2021**, *11*, 1150, doi:10.3390/agronomy11061150.
176. Feki, K.; Tounsi, S.; Mrabet, M.; Mhadhbi, H.; Brini, F. Recent Advances in Physiological and Molecular Mechanisms of Heavy Metal Accumulation in Plants. *Environ Sci Pollut Res* **2021**, *28*, 64967–64986, doi:10.1007/s11356-021-16805-y.
177. Feki, K.; Tounsi, S.; Mrabet, M.; Mhadhbi, H.; Brini, F. Recent Advances in Physiological and Molecular Mechanisms of Heavy Metal Accumulation in Plants. *Environ Sci Pollut Res* **2021**, *28*, 64967–64986, doi:10.1007/s11356-021-16805-y.
178. *Field Crops: Sustainable Management by PGPR*; Maheshwari, D.K., Dheeman, S., Eds.; Sustainable Development and Biodiversity; Springer International Publishing: Cham, 2019; Vol. 23; ISBN 978-3-030-30925-1.
179. Filgueiras, L.; Silva, R.; Almeida, I.; Vidal, M.; Baldani, J.I.; Meneses, C.H.S.G. Gluconacetobacter Diazotrophicus Mitigates Drought Stress in Oryza Sativa L. *Plant Soil* **2020**, *451*, 57–73, doi:10.1007/s11104-019-04163-1.
180. Fiodor, A.; Singh, S.; Pranaw, K. The Contrivance of Plant Growth Promoting Microbes to Mitigate Climate Change Impact in Agriculture. *Microorganisms* **2021**, *9*, 1841, doi:10.3390/microorganisms9091841.
181. Forni, C.; Duca, D.; Glick, B.R. Mechanisms of Plant Response to Salt and Drought Stress and Their Alteration by Rhizobacteria. *Plant Soil* **2017**, *410*, 335–356, doi:10.1007/s11104-016-3007-x.
182. Fortt, J.; González, M.; Morales, P.; Araya, N.; Remonsellez, F.; Coba de la Peña, T.; Ostria-Gallardo, E.; Stoll, A. Bacterial Modulation of the Plant Ethylene Signaling Pathway Improves Tolerance to Salt Stress in Lettuce (Lactuca Sativa L.). *Front. Sustain. Food Syst.* **2022**, *6*, 768250, doi:10.3389/fsufs.2022.768250.
183. Fujita, M.; Hasanuzzaman, M. Approaches to Enhancing Antioxidant Defense in Plants. *Antioxidants* **2022**, *11*, 925, doi:10.3390/antiox11050925.
184. Fukami, J.; Cerezini, P.; Hungria, M. Azospirillum: Benefits That Go Far beyond Biological Nitrogen Fixation. *AMB Expr* **2018**, *8*, 73, doi:10.1186/s13568-018-0608-1.
185. Fukami, J.; Ollero, F.J.; de la Osa, C.; Valderrama-Fernández, R.; Nogueira, M.A.; Megías, M.; Hungria, M. Antioxidant Activity and Induction of Mechanisms of Resistance to Stresses Related to the Inoculation with Azospirillum Brasilense. *Arch Microbiol* **2018**, *200*, 1191–1203, doi:10.1007/s00203-018-1535-x.
186. Fukami, J.; Ollero, F.J.; Megías, M.; Hungria, M. Phytohormones and Induction of Plant-Stress Tolerance and Defense Genes by Seed and Foliar Inoculation with Azospirillum Brasilense Cells and Metabolites Promote Maize Growth. *AMB Expr* **2017**, *7*, 153, doi:10.1186/s13568-017-0453-7.
187. Galicia-Campos, E.; Ramos-Solano, B.; Montero-Palmero, M.B.; Gutierrez-Mañero, F.J.; García-Villaraco, A. Management of Plant Physiology with Beneficial Bacteria to Improve Leaf Bioactive Profiles and Plant Adaptation under Saline Stress in Olea Europea L. *Foods* **2020**, *9*, 57, doi:10.3390/foods9010057.
188. Gamalero, E.; Bona, E.; Todeschini, V.; Lingua, G. Saline and Arid Soils: Impact on Bacteria, Plants, and Their Interaction. *Biology* **2020**, *9*, 116, doi:10.3390/biology9060116.
189. Gao, Y.; Zou, H.; Wang, B.; Yuan, F. Progress and Applications of Plant Growth-Promoting Bacteria in Salt Tolerance of Crops. *IJMS* **2022**, *23*, 7036, doi:10.3390/ijms23137036.
190. García-Cristobal, J.; García-Villaraco, A.; Ramos, B.; Gutierrez-Mañero, J.; Lucas, J.A. Priming of Pathogenesis Related-Proteins and Enzymes Related to Oxidative Stress by Plant Growth Promoting Rhizobacteria on Rice Plants upon Abiotic and Biotic Stress Challenge. *Journal of Plant Physiology* **2015**, *188*, 72–79, doi:10.1016/j.jplph.2015.09.011.
191. García-Villaraco, A.; Boukerma, L.; Lucas, J.A.; Gutierrez-Mañero, F.J.; Ramos-Solano, B. Tomato Bio-Protection Induced by Pseudomonas Fluorescens N21.4 Involves ROS Scavenging Enzymes and PRs, without Compromising Plant Growth. *Plants* **2021**, *10*, 331, doi:10.3390/plants10020331.
192. Ghanbarzadeh, Z.; Mohsenzadeh, S.; Rowshan, V.; Moradshahi, A. Evaluation of the Growth, Essential Oil Composition and Antioxidant Activity of Dracocephalum Moldavica under Water Deficit Stress and Symbiosis with Claroideoglomus Etunicatum and Micrococcus Yunnanensis. *Scientia Horticulturae* **2019**, *256*, 108652, doi:10.1016/j.scienta.2019.108652.
193. Ghassemi, H.R. TASOS1 AND TATM20 GENES EXPRESSION AND NUTRIENT UPTAKE IN WHEAT SEEDLINGS MAY BE ALTERED VIA EXCESS CADMIUM EXPOSURE AND INOCULATION WITH AZOSPIRILLUM BRASILENSE SP7 UNDER SALINE CONDITION. *Appl. Ecol. Env. Res.* **2018**, *16*, 1797–1817, doi:10.15666/aeer/1602_17971817.
194. Ghosh, D.; Sen, S.; Mohapatra, S. Drought-Mitigating Pseudomonas Putida GAP-P45 Modulates Proline Turnover and Oxidative Status in Arabidopsis Thaliana under Water Stress. *Ann Microbiol* **2018**, *68*, 579–594, doi:10.1007/s13213-018-1366-7.
195. González-Bosch, C. Priming Plant Resistance by Activation of Redox-Sensitive Genes. *Free Radical Biology and Medicine* **2018**, *122*, 171–180, doi:10.1016/j.freeradbiomed.2017.12.028.
196. González-Morales, S.; Solís-Gaona, S.; Valdés-Caballero, M.V.; Juárez-Maldonado, A.; Loredo-Treviño, A.; Benavides-Mendoza, A. Transcriptomics of Biostimulation of Plants Under Abiotic Stress. *Front. Genet.* **2021**, *12*, 583888, doi:10.3389/fgene.2021.583888.
197. Gopalakrishnan, S.; Sathya, A.; Vijayabharathi, R.; Varshney, R.K.; Gowda, C.L.L.; Krishnamurthy, L. Plant Growth Promoting Rhizobia: Challenges and Opportunities. *3 Biotech* **2015**, *5*, 355–377, doi:10.1007/s13205-014-0241-x.
198. Goswami, D.; Thakker, J.N.; Dhandhukia, P.C. Portraying Mechanics of Plant Growth Promoting Rhizobacteria (PGPR): A Review. *Cogent Food & Agriculture* **2016**, *2*, doi:10.1080/23311932.2015.1127500.
199. Gouda, S.; Kerry, R.G.; Das, G.; Paramithiotis, S.; Shin, H.-S.; Patra, J.K. Revitalization of Plant Growth Promoting Rhizobacteria for Sustainable Development in Agriculture. *Microbiological Research* **2018**, *206*, 131–140, doi:10.1016/j.micres.2017.08.016.
200. Gritli, T.; Boubakri, H.; Essahibi, A.; Hsouna, J.; Ilahi, H.; Didier, R.; Mnasri, B. Salt Stress Mitigation in Lathyrus Cicera by Combining Different Microbial Inocula. *Physiol Mol Biol Plants* **2022**, *28*, 1191–1206, doi:10.1007/s12298-022-01205-4.
201. Grover, M.; Bodhankar, S.; Sharma, A.; Sharma, P.; Singh, J.; Nain, L. PGPR Mediated Alterations in Root Traits: Way Toward Sustainable Crop Production. *Front. Sustain. Food Syst.* **2021**, *4*, 618230, doi:10.3389/fsufs.2020.618230.
202. Gul, Z.; Tang, Z.-H.; Arif, M.; Ye, Z. An Insight into Abiotic Stress and Influx Tolerance Mechanisms in Plants to Cope in Saline Environments. *Biology* **2022**, *11*, 597, doi:10.3390/biology11040597.
203. Gul, Z.; Tang, Z.-H.; Arif, M.; Ye, Z. An Insight into Abiotic Stress and Influx Tolerance Mechanisms in Plants to Cope in Saline Environments. *Biology* **2022**, *11*, 597, doi:10.3390/biology11040597.
204. Guo, X.; Wang, Q.; Liu, Y.; Zhang, X.; Zhang, L.; Fan, S. Screening of Salt Stress Responsive Genes in Brachypodium Distachyon (L.) Beauv. by Transcriptome Analysis. *Plants* **2020**, *9*, 1522, doi:10.3390/plants9111522.
205. Guo, Z.; Cai, L.; Liu, C.; Chen, Z.; Guan, S.; Ma, W.; Pan, G. Low-Temperature Stress Affects Reactive Oxygen Species, Osmotic Adjustment Substances, and Antioxidants in Rice (Oryza Sativa L.) at the Reproductive Stage. *Sci Rep* **2022**, *12*, 6224, doi:10.1038/s41598-022-10420-8.
206. Gupta, A.; Bano, A.; Rai, S.; Kumar, M.; Ali, J.; Sharma, S.; Pathak, N. ACC Deaminase Producing Plant Growth Promoting Rhizobacteria Enhance Salinity Stress Tolerance in Pisum Sativum. *3 Biotech* **2021**, *11*, 514, doi:10.1007/s13205-021-03047-5.
207. Gupta, A.; Bano, A.; Rai, S.; Mishra, R.; Singh, M.; Sharma, S.; Pathak, N. Mechanistic Insights of Plant-Microbe Interaction towards Drought and Salinity Stress in Plants for Enhancing the Agriculture Productivity. *Plant Stress* **2022**, *4*, 100073, doi:10.1016/j.stress.2022.100073.
208. Gupta, A.; Bano, A.; Rai, S.; Mishra, R.; Singh, M.; Sharma, S.; Pathak, N. Mechanistic Insights of Plant-Microbe Interaction towards Drought and Salinity Stress in Plants for Enhancing the Agriculture Productivity. *Plant Stress* **2022**, *4*, 100073, doi:10.1016/j.stress.2022.100073.
209. Gupta, A.; Mishra, R.; Rai, S.; Bano, A.; Pathak, N.; Fujita, M.; Kumar, M.; Hasanuzzaman, M. Mechanistic Insights of Plant Growth Promoting Bacteria Mediated Drought and Salt Stress Tolerance in Plants for Sustainable Agriculture. *IJMS* **2022**, *23*, 3741, doi:10.3390/ijms23073741.
210. Gupta, S.; Doležal, K.; Kulkarni, M.G.; Balázs, E.; Van Staden, J. Role of Non-Microbial Biostimulants in Regulation of Seed Germination and Seedling Establishment. *Plant Growth Regul* **2022**, *97*, 271–313, doi:10.1007/s10725-021-00794-6.
211. Gururani, M.A.; Ganesan, M.; Song, I.-J.; Han, Y.; Kim, J.-I.; Lee, H.-Y.; Song, P.-S. Transgenic Turfgrasses Expressing Hyperactive Ser599Ala Phytochrome A Mutant Exhibit Abiotic Stress Tolerance. *J Plant Growth Regul* **2016**, *35*, 11–21, doi:10.1007/s00344-015-9502-0.
212. Gururani, M.A.; Upadhyaya, C.P.; Baskar, V.; Venkatesh, J.; Nookaraju, A.; Park, S.W. Plant Growth-Promoting Rhizobacteria Enhance Abiotic Stress Tolerance in Solanum Tuberosum Through Inducing Changes in the Expression of ROS-Scavenging Enzymes and Improved Photosynthetic Performance. *J Plant Growth Regul* **2013**, *32*, 245–258, doi:10.1007/s00344-012-9292-6.
213. Gururani, M.A.; Upadhyaya, C.P.; Strasser, R.J.; Yu, J.W.; Park, S.W. Evaluation of Abiotic Stress Tolerance in Transgenic Potato Plants with Reduced Expression of PSII Manganese Stabilizing Protein. *Plant Science* **2013**, *198*, 7–16, doi:10.1016/j.plantsci.2012.09.014.
214. Gutierrez Albanchez, E.; García-Villaraco, A.; Lucas, J.A.; Gutierrez, F.J.; Ramos-Solano, B. Priming Fingerprint Induced by *Bacillus Amyloliquefaciens* QV15, a Common Pattern in *Arabidopsis Thaliana* and in Field-Grown Blackberry. *Journal of Plant Interactions* **2018**, *13*, 398–408, doi:10.1080/17429145.2018.1484187.
215. Gutierrez Albanchez, E.; García-Villaraco, A.; Lucas, J.A.; Gutierrez, F.J.; Ramos-Solano, B. Priming Fingerprint Induced by *Bacillus Amyloliquefaciens* QV15, a Common Pattern in *Arabidopsis Thaliana* and in Field-Grown Blackberry. *Journal of Plant Interactions* **2018**, *13*, 398–408, doi:10.1080/17429145.2018.1484187.
216. H.G., G.; S., B.S.; M., M.; N., S.; Prasad, M.; Aiyaz, M.; K.N., A.; S.R., N. Induction of Drought Tolerance in Tomato upon the Application of ACC Deaminase Producing Plant Growth Promoting Rhizobacterium Bacillus Subtilis Rhizo SF 48. *Microbiological Research* **2020**, *234*, 126422, doi:10.1016/j.micres.2020.126422.
[truncated: 438,427 more chars]
