# Supplementary material for: Meta-analysis of plant growth-promoting rhizobacteria interaction with host plants: implications for drought stress response gene expression
Source: Front Plant Sci. 2024 Jan 15;14:1282553. doi: 10.3389/fpls.2023.1282553 (PMC10823023; doi:10.3389/fpls.2023.1282553)
Supplement: Supplementary file 29 [file DataSheet_2.docx]

**Figure S1**. Graphic output of all 4 included articles in the meta-analysis regarding the ACO expression with PGPR in plants exposed to drought stress. a) The forest plot illustrates the heterogeneity and the aggregate result; b) The funnel plot detects publication bias.


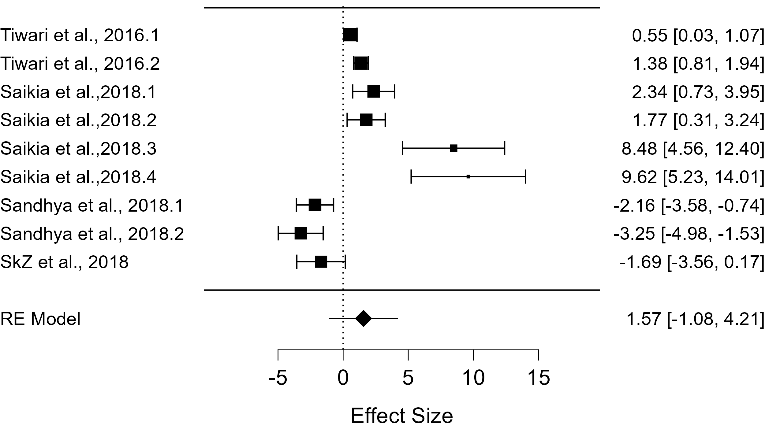

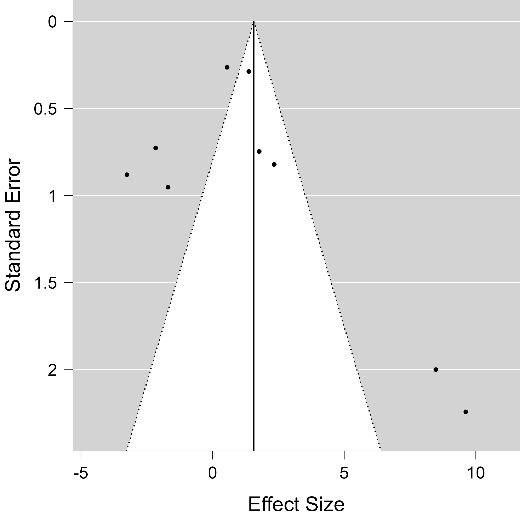


a)

b)

**Figure S2.** Graphic output of all 4 included articles in the meta-analysis regarding the ACS expression with PGPR in plants exposed to drought stress. a) The forest plot illustrates the heterogeneity and the aggregate result; b) The funnel plot detects publication bias.


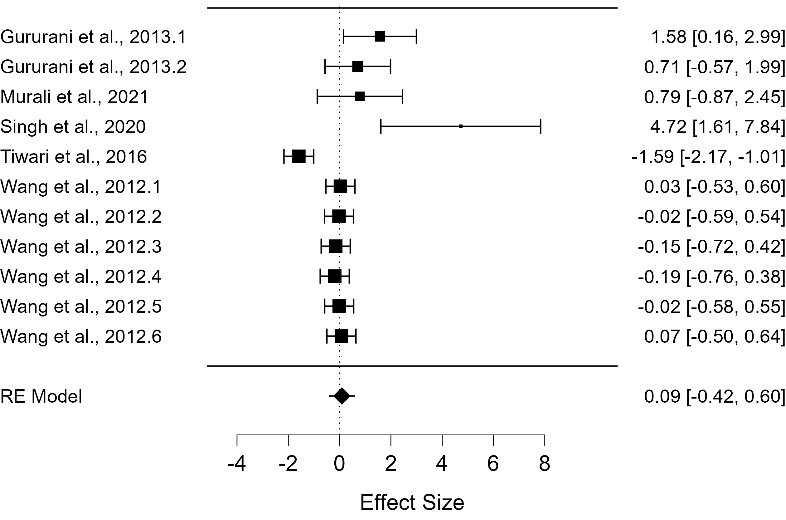

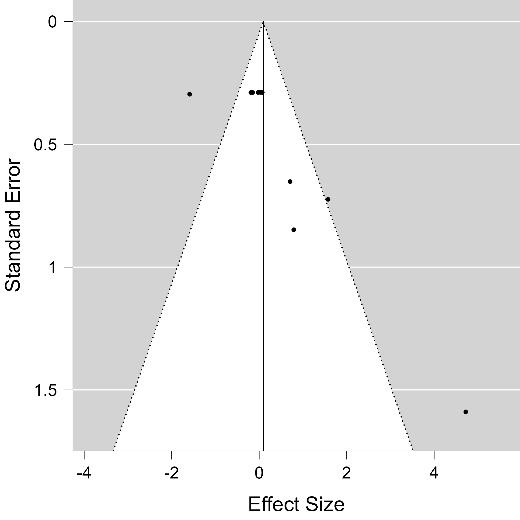


a)

b)


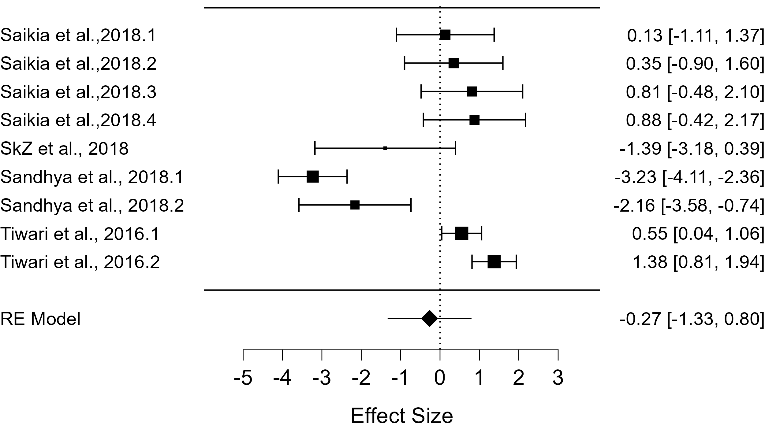

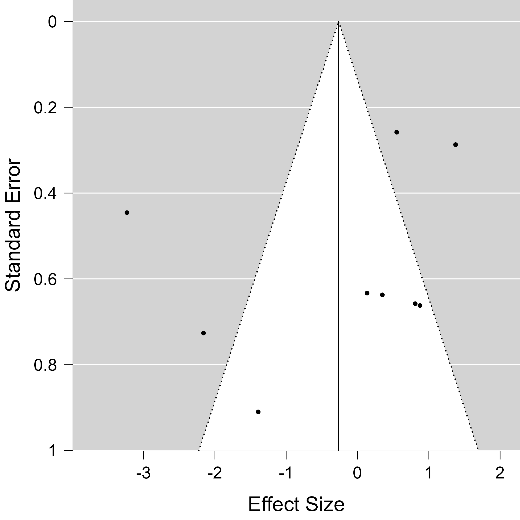


a)

b)

**Figure S3.** Graphic output of all 5 included articles in the meta-analysis regarding the APX expression with PGPR in plants exposed to drought stress. a) The forest plot illustrates the heterogeneity and the aggregate result; b) The funnel plot detects publication bias


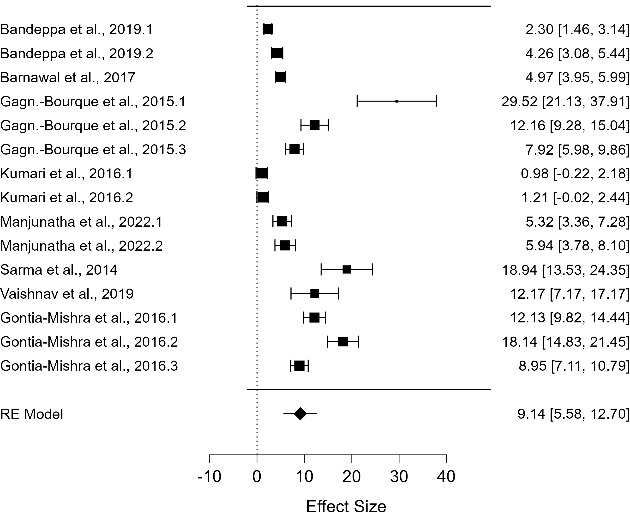

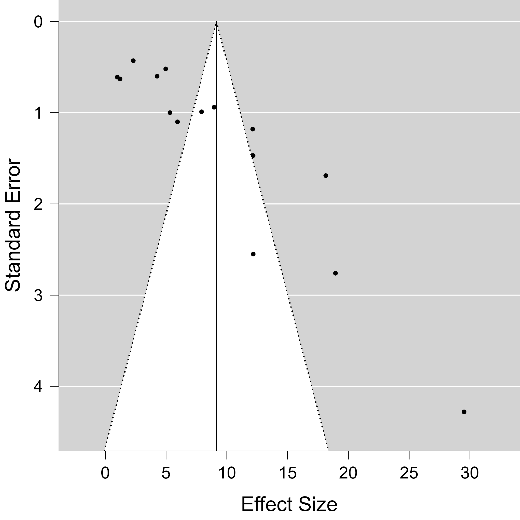


a)

b)

**Figure S4.** Graphic output of all 8 included articles in the meta-analysis regarding the DREB2 expression with PGPR in plants exposed to drought stress. a) The forest plot illustrates the heterogeneity and the aggregate result; b) The funnel plot detects publication bias


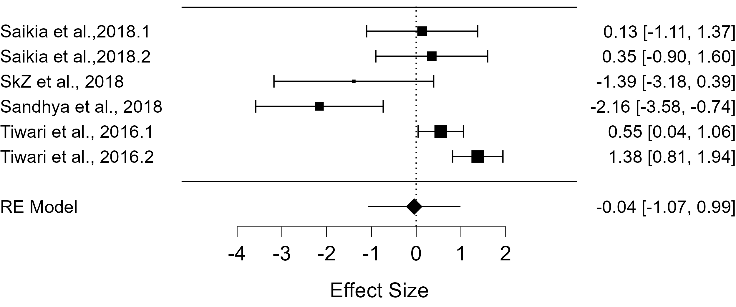

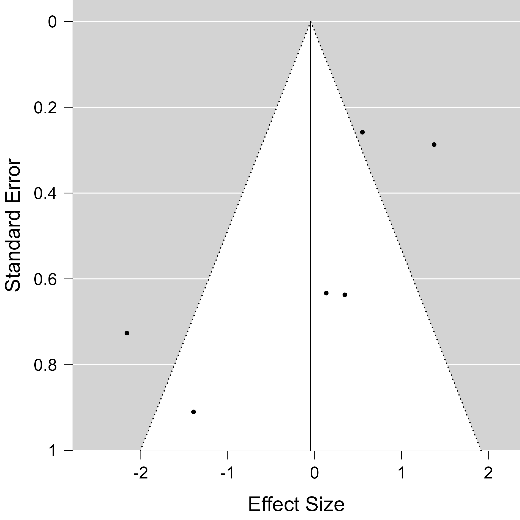


a)

b)

**Figure S5.** Forest plot and funnel plot of ACO. Subgroup analysis from 4 articles was conducted according to drought stress and leaves. a) The forest plot illustrates the heterogeneity and the aggregate result; b) The funnel plot detects publication bias.


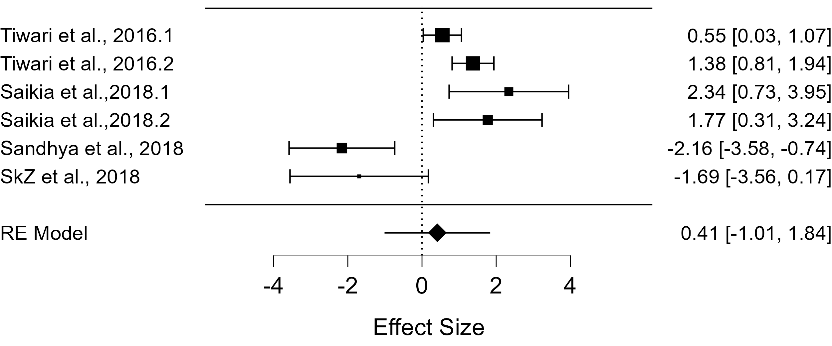

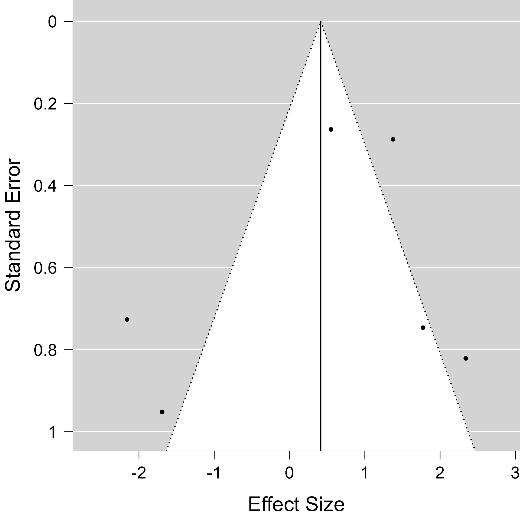


a)

b)

**Figure S6.** Forest plot and funnel plot of ACS. Subgroup analysis from 4 articles was conducted according to drought stress and leaves. a) The forest plot illustrates the heterogeneity and the aggregate result; b) The funnel plot detects publication bias.

**Figure S7.** Forest plot and funnel plot of APX. Subgroup analysis from 4 articles was conducted according to the used method to induce drought stress (dehydration) and leaves. a) The forest plot illustrates the heterogeneity and the aggregate result; b) The funnel plot detects publication bias.


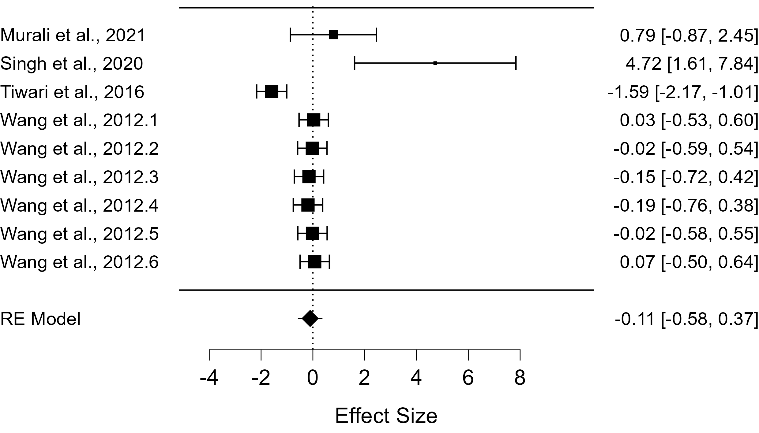

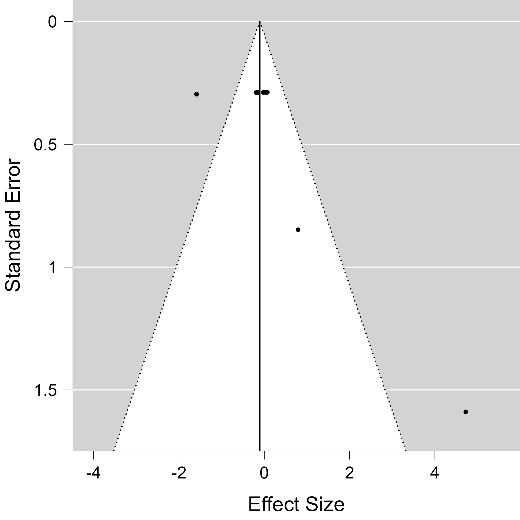


a)

b)


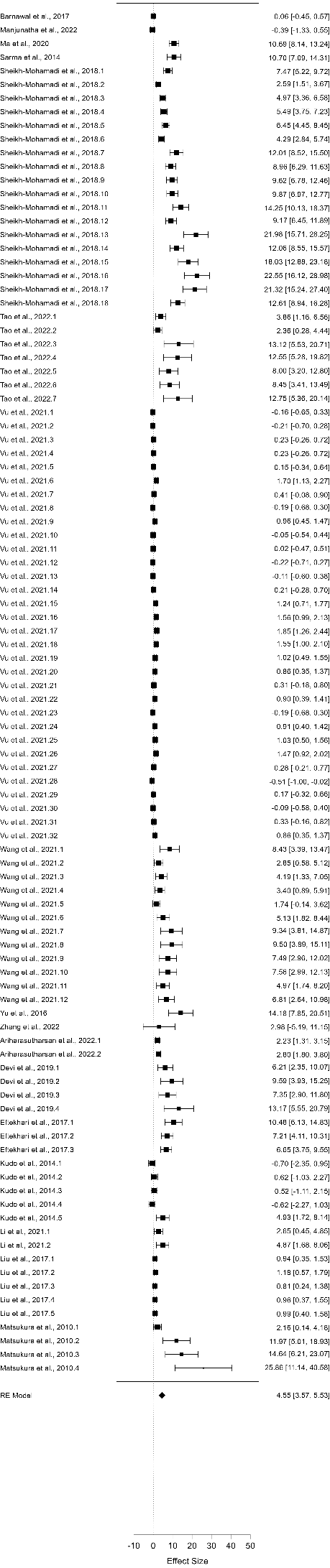

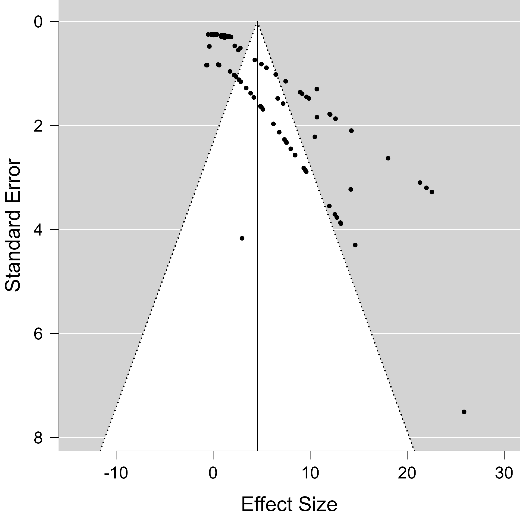


a)

b)

**Figure S8.** Graphic output of all 17 included articles in the meta-analysis regarding the DREB2 expression without PGPR in plants exposed to drought stress. a) The forest plot illustrates the heterogeneity and the aggregate result; b) The funnel plot detects publication bias.
